# Development of a Manual for Disaster Medical Support Using Korean Medicine for Disaster Survivors

**DOI:** 10.1089/jicm.2022.0561

**Published:** 2023-06-06

**Authors:** Chan-Young Kwon, Joohee Seo, Sang-Ho Kim

**Affiliations:** ^1^ Department of Oriental Neuropsychiatry, Dong-Eui University College of Korean Medicine, Busan, Republic of Korea.; ^2^ Department of Korean Neuropsychiatry, National Medical Center, Seoul, Republic of Korea.; ^3^ Department of Neuropsychiatry of Korean Medicine, Pohang Korean Medicine Hospital, Daegu Haany University, Pohang-si, Republic of Korea.

**Keywords:** disaster, trauma, integrative medicine, guidance, acupuncture, herbal medicine

## Abstract

**Objective:**

Disasters adversely affect the mental health of disaster survivors, leading to depression, anxiety, and stress-related disorders. Survivors complain of not only psychological symptoms but also physical symptoms such as insomnia, pain, and fatigue. Providing immediate and effective psychological support to all survivors is difficult because human and physical medical resources are limited. Therefore, the authors developed a manual for disaster medical support using Korean medicine (KM) for disaster survivors to provide prompt, effective, and long-term support that supplement existing psychological support.

**Methods:**

In this article, the authors introduce KM treatment protocols, which are unique elements of the manual. In addition, the authors have developed a step-by-step treatment protocol based on the stage and condition of survivors, as well as separate treatment protocols for psychological and physical symptoms.

**Results:**

The interventions include ear acupuncture, acupuncture, herbal medicine, breathing relaxation, stabilization techniques, emotional freedom technique, and self-care methods such as acupressure, exercise, and walking meditation. This manual has been certified as an official procedure of the Korean Society of Oriental Neuropsychiatry.

**Conclusions:**

The authors believe that this manual will enable KM doctors to be effectively utilized as medical resources at disaster sites. Furthermore, this manual would provide a good model that can be extended to countries that wish to use integrative medicine for disaster support to implement the commitments of the Declaration of Astana.

## Introduction

Infectious disasters such as severe acute respiratory syndrome, Middle East respiratory syndrome, swine flu (H1N1), and Ebola virus disease have had a huge impact on the world, and the coronavirus disease 2019 (COVID-19) pandemic is currently imposing a huge health, medical, and economic burden worldwide.^1,2^ Mankind has also experienced various natural disasters, including hydrometeorological, geological, and biological disasters, and the effects of these disasters still persist.^3^ Korea also suffers from natural disasters every year, including heavy rain, typhoons, and heavy snow. Between 2010 and 2019, the average annual property damage was estimated at 352.7 billion won, and the restoration amount was estimated at 823.6 billion won.^4^

Disasters cause serious and varied damage to a wide area, limiting the availability of human and material resources and making recovery difficult. As the damage caused by such disasters is not easily resolved, disaster survivors mainly live in shelters during the reconstruction period. When survivors live in these shelters for a long time, they suffer not only from disaster trauma but also from various health problems such as infectious diseases, cold, pain, insomnia, and exacerbation of existing chronic diseases.^5,6^ Because disasters adversely affect the mental health of disaster survivors, resulting in depression, anxiety, and stress-related disorders, systematic disaster support at the national level is important.^7^

In Korea, the National Trauma Center, which was installed in 2018 at the National Mental Health Center, oversees psychological support after large-scale disasters. In cooperation with relevant organizations, the National Trauma Center establishes a prompt and unified intervention system to provide disaster response services and trauma treatment programs for disaster survivors in the event of a national disaster, especially in high-risk groups with mental health problems.^8^ Currently, psychological interventions such as cognitive behavioral therapy, exposure therapy, eye movement desensitization and reprocessing, and stabilization techniques are mainly used for disaster psychological support.^9^

However, survivors complain of not only psychological symptoms but also various physical symptoms such as insomnia, pain, and fatigue.^10^ In addition, when a large number of survivors and displaced persons are present, providing immediate and effective psychological support is difficult because human and physical medical resources are limited. Moreover, long-term psychological support can lead to burnout among practitioners.^11^ Therefore, there is a need for complementary intervention methods that can provide prompt, effective, and long-term support to supplement existing psychological support at disaster sites.

Korea has a unique dual medical system, with both Western medicine (WM) and Korean medicine (KM) doctors working in the national medical system.^12^ However, despite the fact that disaster medical support must be addressed by mobilizing all available human and material resources, KM treatment support has been limited to individual voluntary medical services and has not been included in the national support system. Moreover, in this context, there is no manual that can be used by KM doctors at disaster sites. Accordingly, the research team developed a manual for disaster medical support using KM for disaster survivors.

### Ethical considerations

Clinical trial registration and the acquisition of ethics approval from an Institutional Review Board or of informed consent from patients are not applicable to this article.

## Materials and Methods

### Purpose and target population of the manual for disaster medical support using KM for disaster survivors

The purpose of this manual is to assist KM doctors in managing survivors' psychological, physical, and behavioral stress responses (hereafter referred to as “disaster trauma symptoms”) after a disaster experience. In addition, this manual has the aim to educate disaster survivors regarding self-management skills so that they can control their trauma symptoms on their own.

The target population of the manual is as below: (1) survivors (i.e., primary victims) with physical and mental symptoms of disaster trauma. (2) Disaster survivors' families, relatives, and close acquaintances; support personnel (e.g., firefighters, police officers, rescue workers, clinicians, nurses, social workers, psychological support personnel, clergy, and public officials); and community-dwelling residents who have indirectly experienced disasters. (3) Survivors who have received conventional disaster psychological support, such as cognitive behavioral therapy, exposure therapy, eye movement desensitization and reprocessing, and stabilization techniques, and psychiatric medications such as antidepressants, but their symptoms do not improve or they do not prefer the above treatments. (4) Survivors who wish to receive KM treatment in parallel with conventional disaster psychological support. (5) Survivors who do not wish to receive conventional disaster psychological support, but wish to receive KM treatment. (6) Survivors who have been referred for KM treatment by the disaster psychological support center and/or related organizations.

However, it is important to note that the person must be a survivor who does not have severe hyperarousal or psychotic symptoms or is at risk of harm, self-harm, or suicide. In these cases, psychotropic drugs and/or specialized psychotherapy should be considered first.

### Development of the manual

To develop the manual, the researchers collected and reviewed existing systematic reviews of KM modalities, other disaster medical support cases, and existing domestic and overseas disaster psychological support manuals, from January to June 2020. Three authors developed a draft manual based on this evidence and another manual from July to December 2020. The major KM modalities included in the draft manual were acupuncture (including ear acupuncture), herbal medicine, emotional freedom technique (EFT), acupressure, stabilization technique, Daoin exercise (a type of Asian traditional exercise), and meditation, based on the references above. The draft also included self-management practices that survivors could independently implement.

**FIG. 1.**
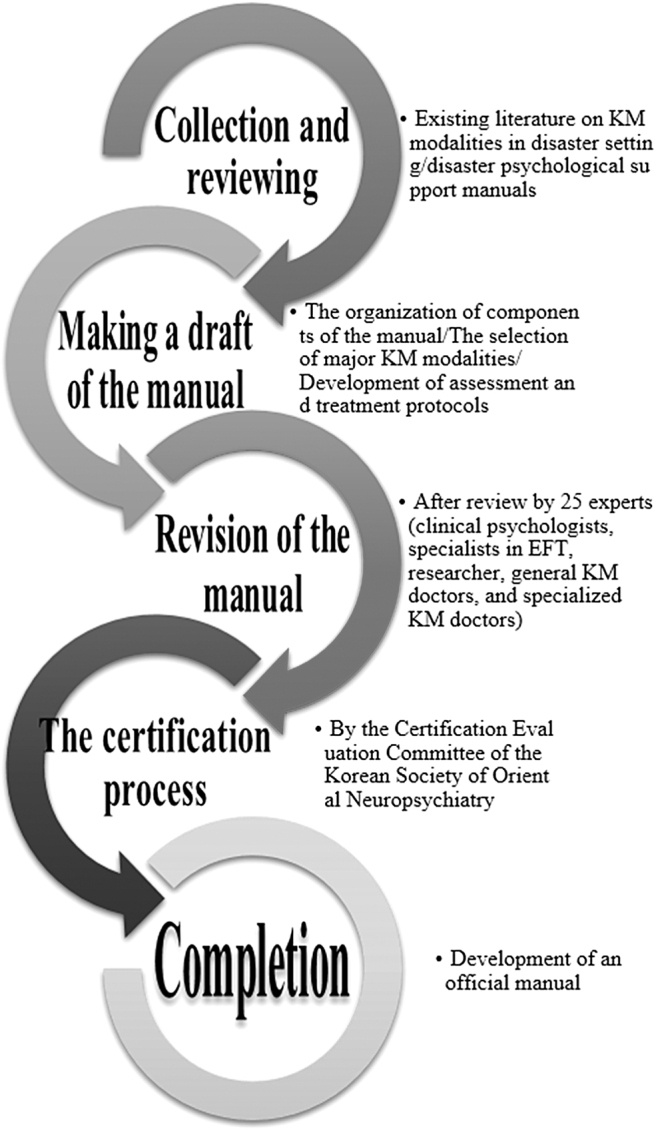
Development process of this the manual. EFT, emotional freedom technique; KM, Korean medicine.

The draft was revised after being reviewed by a total of 25 experts, including 2 clinical psychologists, 3 general KM doctors, 2 specialists in EFT, 1 senior researcher at the Korea Institute of Oriental Medicine, and 17 specialized KM doctors (neuropsychiatry, internal medicine, and acupuncture), from January to June 2021. The revised version was certified by the Certification Evaluation Committee of the Korean Society of Oriental Neuropsychiatry from July to August 2021 and was certified as an official manual on September 3, 2021. The Certification Evaluation Committee consisted of five experts who are specialists in neuropsychiatry of KM and professors at the College of KM. The authors of this article also made a summary of the manual to increase its usability (Fig. 1).

### Components of the manual

This manual consists of the following components: (I.) Overview, (II.) Treatment protocol, (III.) Treatment details, and (IV.) Appendix (Table 1). In this article, the authors introduce KM treatment protocols and details, which are unique elements of the manual.

**Table 1.** Components of the Manual

Domain	Contents
I. Overview	(1) Necessity, (2) purpose, and (3) target of the manual, (4) general reaction of survivors during a disaster, and (5) the domestic disaster psychological support system.
II. Treatment protocol	(1) Flowchart of KM management, (2) history taking and assessment manual, (3) step-by-step (acute/subacute/chronic) treatment protocol, (4) treatment protocol for each symptom, including psychological symptoms (overstrain, fear, depression, and anger) and physical symptoms (insomnia, dyspepsia, exhaustion, headache, dizziness, and pain), (5) psychological first aid, and (6) other considerations.
III. Treatment details	(1) Ear acupuncture, (2) acupuncture, (3) herbal medicine, (4) EFT, (5) stabilization technique, and (6) self-care methods, including acupressure, Daoin exercise, and meditation.
IV. Appendix	(1) The diagnostic criteria for PTSD (DSM-5), (2) medical record form, (3) agreement form of personal information collection and use, (4) validated assessment tools for survivors, (5) case of group treatment, and (6) EFT sheet.

DSM, Diagnostic and Statistical Manual of Mental Disorders; EFT, emotional freedom technique; KM, Korean medicine; PTSD, post-traumatic stress disorder.

### Treatment protocol and details of the manual

#### Flowchart of KM management

**FIG. 2.**
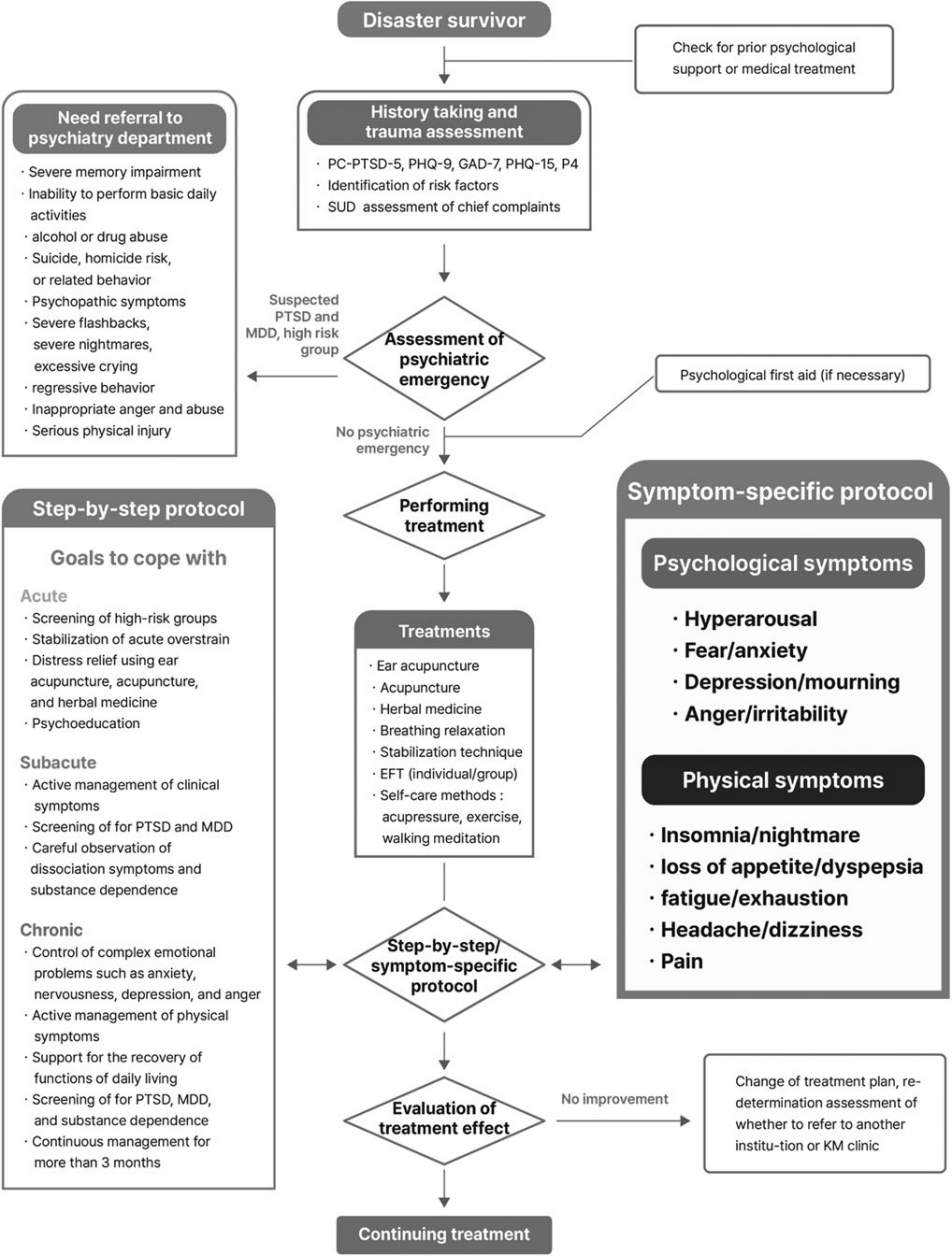
Flowchart of KM management. EFT, emotional freedom technique; GAD-7, Generalized Anxiety Disorder Assessment; KM, Korean medicine; MDD, major depressive disorder; P4, P4 Screener for assessing suicide risk; PC-PTSD-5, primary care PTSD screening according to DSM-5; PHQ, Patient Health Questionnaire; PTSD, post-traumatic stress disorder; SUD, subjective unit of distress.

The flowchart presents the basic examination after a disaster victim has visited or been referred for KM medical support, mental health evaluation, screening for psychiatric emergencies, implementation of KM treatments and self-care methods, evaluation of treatment effects, and review of treatment continuation as a series of processes (Fig. 2).

### History taking and assessment manual

In general, the history taking and assessment process are not different from other disaster mental health manuals, but it emphasizes the presence of physical symptoms as well as mental symptoms after psychological trauma, and the unique feature of this manual is that it includes KM-specific examination details (Table 2).

**Table 2.** History Taking and Assessment Process

Domain	Contents
Demographic information	Name, sex, date of birth, contact information, place of residence, marital status, occupation, visit route, and previous psychological support experience
Basic medical examination	Disaster type, trauma response stage, disaster damage level, and vital signs
Mental health screening tests	PC-PTSD-5, PHQ-9, GAD-7, PHQ-15, P4
Detailed medical examination	Chief complaints, onset, current medical history, past medical history, family medical history, medication history, and drinking and smoking
Classification of chief complaints	Predominantly psychological symptoms (overstrain, fear, depression, and anger) Predominantly physical symptoms (insomnia, anorexia, lethargy, headache/dizziness, and pain)
KM-specific examination	Appetite/digestion, stool/urine, sleep, thirst/dry mouth, chills/fever, symptoms/signs related to five viscera and six bowels, pulse examination, tongue examination, abdominal examination, inspection, and diagnosis
Risk factor assessment	Psychiatric history, past traumatic experiences, current stressful events, and weak support system
Explanation of evaluation results and treatment	The examination and evaluation results are explained. High-risk groups are referred to the psychological support center or psychiatry department. Treatment is carried out according to the step-by-step and/or symptom-specific protocols

DSM, Diagnostic and Statistical Manual of Mental Disorders; GAD-7, Generalized Anxiety Disorder Assessment; KM, Korean medicine; P4, P4 Screener for assessing suicide risk; PC-PTSD-5, primary care PTSD screening according to DSM-5; PHQ, Patient Health Questionnaire; PTSD, post-traumatic stress disorder.

### Step-by-step treatment protocol

It is desirable to provide treatment based on the stage and condition of the survivors after the disaster. The different stages after disaster, such as emergency, acute, subacute, and chronic stages, and corresponding coping goals and coping methods are presented in Table 3.

**Table 3.** Step-by-Step Treatment Protocol

Stage	Condition	Coping goal	Coping methods
Emergency stage: 1–3 days after the disaster	Psychological emergency Shock, anxiety, fear, horror	Understanding the on-site situation and preparing for treatment	Identification of disaster site situation Composition of medical support team, installation of on-site clinics, preparation of medical supplies Promotion of KM treatment implementation and referral request to related organizations
Acute stage: 3 days–1 month after the disaster	Overstrain and/or fear Acute stress response Shock, anxiety, anger, despair Emotional paralysis, poor judgment, devastation Disruption of daily life rhythm	Stabilization of overstrain/fear state Identification of high-risk groups and referral to specialized organizations	Screening test: identification of symptom severity in survivors and identification of high-risk groups; if necessary, referral to specialized organizations Immediate application of psychological first aid, if necessary Ear acupuncture, acupuncture, and herbal medicine Breathing relaxation method, concurrently with acupuncture procedure Psychoeducation EFT Education of self-care methods Treatment frequency: three times a week
Subacute stage: 1–3 months after disaster	The period when clinical symptoms and distress become more pronounced and worse High risk of developing PTSD and MDD Concerned about exacerbating anxiety and anger of the vulnerable group Occurrence of physical symptoms Disruption of daily life rhythm	Relief of psychological and physical symptoms and distress Identification of high-risk groups and referral to specialized organizations KM examination of physical symptoms Prevention of PTSD and MDD	Screening test: referral to specialized organizations, in case of PTSD, severe depression, dissociation, or suicidal risk Continuation of ear acupuncture, acupuncture, and herbal medicine Stabilization techniques: breathing meditation, grounding, I-Jeong-Byeon-Gi therapy, mindfulness and loving beingness psychotherapy EFT (individual/group) Education of self-care methods Treatment frequency: two to three times a week
Chronic stage: 3 months or later after the disaster	Complicated and persistent psychological/physical symptoms High risk of developing PTSD and MDD Occurrence of problematic dependence such as addiction Exhaustion of mind and body due to continued extreme chaos	Active treatment and management of complex mind and body symptoms Identification of high-risk groups and referral to specialized organizations Support for recovery of daily living functions	Ear acupuncture, acupuncture, and herbal medicine for persistent physical and psychological symptoms Stabilization techniques: I-Jeong-Byeon-Gi therapy, mindfulness and loving beingness psychotherapy EFT (individual/group) Education of self-care methods Treatment frequency: one to two times a week (continuous treatment for at least 3 months) If necessary, referral to nearby KM clinics and hospitals

EFT, emotional freedom technique; KM, Korean medicine; MDD, major depressive disorder; PTSD, post-traumatic stress disorder.

### Treatment protocol for each symptom

The clinical symptoms of disaster survivors were divided into psychological and physical symptoms, and intervention protocols (ear acupuncture, acupuncture, herbal medicine, and acupressure) and coping methods for each symptom were presented. Treatment protocols based on symptoms can aid in the rapid selection and introduction of interventions at disaster sites. The details of the intervention were formulated through discussion by the development team and expert advice based on previous documents, including KM clinical practice guidelines (Tables 4 and 5).

**Table 4.** Korean Medicine Treatments for Each Psychological Symptom

Psychological symptoms						
Symptoms/signs		Hypervigilance/anxiety	Fear/emotional numbing	Depression/mourning	Anger/irritability	
Treatments to be considered		Ear acupuncture, acupuncture, herbal medicine, breathing exercise, stabilization method, EFT				
Intervention	Herbal medicine	Sihosogan-san, Gamisoyo-san, Sangoin-tang, Shihogayonggolmoryo-tang, Ondam-tang, Xiaotanjieyu-fang (Modified soyo-san), Jieyu-wan (Soyo-san+Gammakdaejo-tang), Hwangryunagyo-tang		Gaejigayonggolmoryo-tang, Shihogesikungang-tang, Kwibi-tang, Chunwangbosim-dan, Woohwangchungsim-won	Shihosogan-tang, Gamisoyo-san, Banhahubak-tang, Kwibi-tang, Shihogayonggolmoryo-tang, Geijibokryungh-wan, Youkgunja-tang	Hwangryunhaedok-tang, Ukgan-san
	Acupuncture	HT7, PC6, SP6, BL15		PC8, HT7, KI1, Chong points (SP1, ST45, PC9, etc.)	GV20, EX-HN3, LI4, LR3, ST36	LR3, LR2, SP6, CV17
	Ear acupuncture	Sympathetic, Shen Men, kidney, liver, and lung points (NADA protocol)				
Self-care	Acupressure	HT7, PC6		PC8, Chong points	LI4, GV20	LR3, SP6

EFT, emotional freedom technique; NADA, National Acupuncture Detoxification Association.

**Table 5.** Korean Medicine Treatments for Each Physical Symptom

Physical symptoms						
Symptoms/signs		Insomnia/nightmare	Anorexia/dyspepsia	Lethargy/exhaustion	Headache/dizziness	Pain
Treatments to be considered		Ear acupuncture, acupuncture, herbal medicine				
Intervention	Herbal medicine	Sangoin-tang, Kwibi-tang, Chunwangbosim-dan, Ondam-tang	Hyangsapyeongwi-san, Banhasasim-tang, Bulhwangeumjeonggi-san, Samchulkunbi-tang, Banhahubak-tang, Daehwajung-eum, Naesowhajung-tang, Youkgunja-tang, Sayeok-san	Bojungykki-tang, Palmul-tang, Gamisoyo-san, Kwibi-tang, Yukmijiwhang-wan/Pamijiwhang-wan, Ssanghwa-tang, Shipjeondaebo-tang	Headache: Galgeun-tang, Chogsanggeontong-tang, Banhabaekchoolcheunma-tang, Oryung-san Dizziness: Banhabaekchoolcheunma-tang, Oryung-san, Danguijakyak-san, Cheonmagudeung-eum, Younggaechulgam-tang, Jaeumgeonbi-tang	Ojeok-san, Gumiganghwal-tang, Chogsanggeontong-tang, Jakyakgamcho-tang, Danggwisu-san, Ssanghwa-tang
	Acupuncture	Sedative: GV20, EX-HN1, EX-HN 22, HT7, SP6, KI1, BL62 Tonifying: KI6	CV12, ST25, LI4, PC6, ST36, LR3	SP3, ST36, CV4, KI1, SP1	Headache: GV20, GB20, LI4, GB41 Dizziness: LR3, LI4, GB39, GV20, GB20, EX-HN5, EX-HN3, GV23, GB8, GB21	Acupoints according to pain site, Sa-am acupuncture (removing blood stasis method)
	Ear acupuncture	Sympathetic, Shen Men, kidney, liver, and lung points (NADA protocol)				
Self-care	Acupressure	HT7, KI1, EX-HN 22	CV12, ST36, ST25	ST36, SP1, KI1	EX-HN5, GB20, LI4, GB44	Ashi points

NADA, National Acupuncture Detoxification Association.

### Psychological first aid

Various programs have been developed and distributed worldwide to promote and restore the psychological stability of disaster survivors. Psychological support for individuals exposed to disasters can be largely divided into acute phase intervention within 1 month after a disaster, subacute intervention within 1 to 3 months after a disaster, and chronic phase intervention after >3 months of a disaster. Among them, psychological first aid, a representative program for mental health support in acute disasters, is “the act of providing humanitarian help to people in pain”; it is not an intervention, but a psychosocial service that includes practical and direct assistance needed in emergency situations.^13^

### Emotional freedom technique

EFT is a psychiatric approach that controls energy by physically stimulating the meridians and acupuncture points. It was registered as a new KM technique for post-traumatic stress disorder (PTSD) in 2019.^14^ This treatment combines short-term exposure (symptom selection and evaluation) with physical interventions (acupoint tapping) and cognitive therapy (affirmations).^15^ EFT is particularly useful for post-traumatic responses, and clinical studies have shown significant improvements in pain, anxiety, depression, and post-traumatic stress disorder scales.^16^ Moreover, EFT is a self-care technique that can be used for educating disaster survivors to restore their sense of control and cope with symptoms (Table 6).

**Table 6.** Emotional Freedom Technique Process

Process	Description
Identification of the problem	Specify the problem, symptom, or discomfort to target.
Assessment	A scale of 0 to 10 determines the degree of discomfort or pain caused by the problem.
Setup	This is the process of starting acupoint tapping. With two fingers (mainly index and middle fingers) continuously tap on the opposite hand (SI3) and repeat three times a simple receptive phrase. The phrase should be about acknowledging the problem and accepting oneself nonetheless.
Sequence	Next, while repeating an associative phrase, tap the following parts of the body in the order mentioned approximately seven times: crown, beginning of the eyebrows, sides of the eyes, under the eyes, under the nose, chin, beginning of the clavicle, and under the armpits. The associative phrase may differ depending on the problem, for example, “this shoulder pain,” “an accident at that time,” “a nightmare.”
Re-assessment	A scale of 0 to 10 determines the degree of discomfort or pain caused by the problem. If the score does not decrease after performing steps 1 to 4, repeat until it decreases or the mind becomes stable. When repeating, affirmations of acceptance and associative phrases can be slightly modified to reflect the changed aspects.
Additional option: choice method	This is a choice affirmation, where the survivor recites what he or she wishes to be in the future.
Additional option: group therapy	Group therapy can provide a feeling of empathy and comfort, as well as borrowing benefits. It can be planned for 4 weeks once a week, and the duration of each episode is recommended as 1 h, but it can be set flexibly according to the situation. Self-learning audio recording files and self-care protocols are provided so that survivors can practice individually every day.
Cautions	EFT is a noninvasive method of tapping acupoints on the patient's own and it does not directly harm the subject; hence, there are no safety concerns regarding the procedure, and no side effects have been reported in previous studies.^34^ However, rather than directly recalling traumatic memories, apply EFT mainly to the physical/psychological symptoms of survivors. If an individual complains of symptoms such as severe agitation/restlessness, dissociation, delusions, hallucinations, or suicidal ideation or does not respond to EFT procedures after three to four sessions, other psychotherapy or pharmacological therapy should be considered and the patient should be referred to a mental health expert.

EFT, emotional freedom technique.

### Stabilization technique

The stabilization technique is the most basic and essential treatment method for acute disaster trauma.^17^ In a state in which the limbic system, including the amygdala, is overactivated and the prefrontal cortex function has deteriorated after experiencing a disaster,^18^ the stabilization technique may help survivors to feel more pleasurable and comfortable, to better handle recurring thoughts, and to improve psychosocial functioning.^19^ The techniques used are presented in Table 7.^20^

**Table 7.** Stabilization Techniques^20^

Techniques	Features and indications	Notes
Psychoeducation	Change in perception Knowing that one's reactions after the disaster trauma are natural, predictable, and experienced by many people reduces his/her anxiety, shame, and feeling of guilt.	It is necessary to establish a trust relationship before its implementation. Implement it in a group to proceed efficiently. Use pictures, props, and methods that can disperse hyperarousal (herbal tea, hot packs, or ice packs) together.
Breathing relaxation	Stability of hyperarousal through autonomic nervous system sedation Easy to apply	Caution in case of hypoarousal state Can be used as a group therapy
Grounding	Can be used for both hyperarousal and hypoarousal Allows the survivor's frame of mind to stay within the window of tolerance Let go of overwhelming thoughts, feelings, and memories, and let them stay in the present moment.	Utilizes appropriate senses according to the situation and the condition of survivors. Connect survivors to current supportive environments and relationships (e.g., safety factors such as solid ground or being with people who help them).
I-Jeong-Byeon-Gi therapy using orienting response	Can be used for both hyperarousal and hypoarousal Attention diverting activity	Choose an appropriate method according to the situation and the condition of survivors.
Containment exercise (in mindfulness and loving beingness psychotherapy)	When invasive memories and flashbacks are evident, this treatment allows acquiring the ability to control uncomfortable memories and emotions.	Conducted on the premise of sufficient resources and a field of psychological safety. Reinforce positive bodily sensations and emotions by processing uncomfortable memories.
Resource mindfulness (in mindfulness and loving beingness psychotherapy)	Can be used as a preparation process for exposure therapy. Can be used at the end of treatment.	Allow the patient to fully relax, through breathing before the therapy. If the survivor cannot find a field of psychological safety or if the negative memories are linked to the resource, the anxiety can be exacerbated.

### Others

**FIG. 3.**
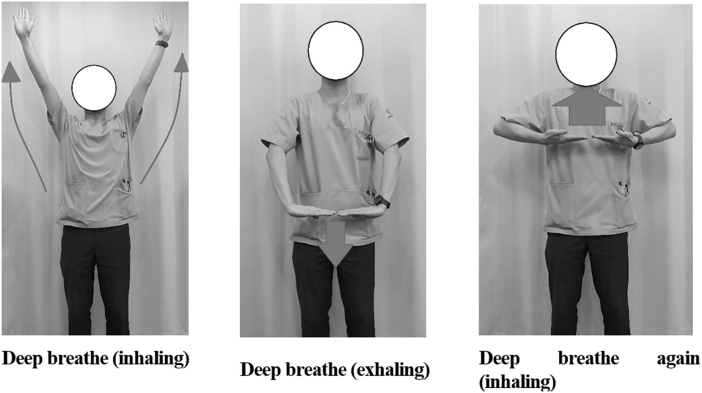
Examples of Daoin exercise for survivors after disaster.

The Daoin exercise causes movement of the spine through movement of the extremities, which in turn causes movement of the intestines, consequently correcting the structure of the musculoskeletal system and promoting smooth blood circulation in the internal organs.^21^ Moreover, because this exercise does not require much space, it is highly useful for survivors whose activities are restricted owing to physical pain and functional deterioration caused by long-term shelter life at the disaster site. It can be used as a type of self-care method or as a form of group therapy to help improve the pain and function of disaster survivors and their quality of life (Fig. 3).

Walking meditation is a dynamic meditation that focuses on all the senses that survivors feel while walking and allows them to feel a sense of stability. Moreover, as walking meditation does not require high-intensity physical activity, it can be safely performed even by elderly survivors with a physical disease or a certain level of self-gaiting ability. In addition, this method has been reported to be effective in alleviating depressive symptoms and improving health functional status and vascular reactivity; therefore, it can be usefully utilized in disaster support sites.^22^

## Discussion

This is the first manual on disaster medical support using integrative medicine for disaster survivors in South Korea (Supplementary Files S1 and S2). The authors developed this manual based on related comprehensive evidence, review by several experts, and the certification process by the Certification Evaluation Committee of the Korean Society of Oriental Neuropsychiatry. It was designed to enable the treatment of disaster survivors' physical and psychological symptoms using various KM and mind–body interventions. This manual includes a treatment protocol for each stage and symptom for ease of use at disaster sites.

In addition to existing stabilization techniques, various interventions such as meditation and EFT are mentioned in the treatment protocol. Furthermore, self-care methods, such as acupressure, EFT, Daoin exercise, and walking meditation, are also considered in this manual. These are highly useful in survivors whose activities are restricted owing to long-term shelter life at the disaster site. This manual will be revised every 5 years by a trauma research group affiliated with the Korean Society of Oriental Neuropsychiatry. The revision procedure will be re-accredited by the accreditation evaluation committee of the Korean Neuropsychiatric Association.

The KM modalities used in this manual have strengths, such as feasibility, safety, and cost-effectiveness in disaster medical support. As acupuncture (especially ear acupuncture) is a simple, effective, and safe intervention, it has been used at many disaster sites.^23,24^ Herbal medicine can aid in coping with various symptoms such as exhaustion, hypothermia, and loss of appetite, which are difficult to medically treat at disaster sites.^25^ Saikokeishikankyoto (Shiho Gyeji geongang tang) was introduced as a nonpsychological treatment in the PTSD Guidelines (2018) of the International Trauma Stress Society.^26,27^ EFT is a safe nonpharmacological treatment with wide indications. It can be used as a group treatment and can also be provided online or over the phone. Because of these advantages, EFT has been used as an effective stabilization technique at disaster sites.^28^ Meditation has already been mentioned in the KM Doctor's Mental Health Instruction Manual for telemedicine for COVID-19 patients.^29^

Therefore, the use of KM modalities may help improve access to treatment and the quality of life of survivors by supplementing current disaster medical support focused on psychological treatment. The authors have considered cooperation between WM doctors and other experts by adopting the standardized evaluation method of the National Trauma Center in this manual. It is expected that this manual will help KM doctors be effectively utilized as medical resources at disaster sites. Moreover, as there is no manual that uses integrative medicine for disaster medical support worldwide, this manual can be a good model to be extended to countries that wish to use integrative medicine for disaster support.

### Limitations and implication

For this manual to be applied to a disaster site, some limitations must be considered. First, because this manual has not been actually applied at a disaster site, research applying it to an actual site is needed. A practical research protocol with a research design, such as a prospective registry study that can be performed in the field, should be developed.^30^ Second, a well-organized educational and training program based on this manual for KM doctors should be developed and implemented to strengthen disaster preparedness and usability. An online continuing education program should be considered so that KM doctors can easily access it. Simulation cases using simulated disaster survivors may be utilized.

Third, it is necessary to develop an individualized protocol that considers vulnerable groups, such as elderly people, children, women, bereaved, patients with mental disorders, and those with severe chronic medical illnesses.^31^ Fourth, the development of a more specific cooperative manual integrating the disaster psychological support modalities of the National Trauma Center and the KM modalities of this manual is necessary for the use of KM in the national disaster management system. Fifth, large-scale disasters such as earthquakes, tsunamis, and nuclear power plant accidents have resulted in thousands of long-term evacuees.^32^ Therefore, the manual should include a strategy for the long-term care of evacuees.^33^ This manual will be developed as a continuing education program for KM doctors in the future. In addition, the manual should be continuously updated to reflect the field applications and research results.

The 2018 Declaration of Astana especially highlights the application and appropriate inclusion of traditional medicines as factors for the successful establishment of primary national health services.^34^ The Declaration of Astana makes pledges of commitment toward four key areas: (1) making bold political choices for health across all sectors; (2) building sustainable primary health care; (3) empowering individuals and communities; and (4) aligning stakeholder support to national policies, strategies, and plans. Our manual is in concordance with the commitments above and success metrics such as (1) knowledge and capacity-building and (2) human resource for health, as per the Declaration of Astana. First, KM has unique therapeutic benefits for disaster-related medical support. KM treatments emphasize not only the psychological symptoms of trauma but also the physical symptoms among survivors.

The introduction of KM into the national trauma response system provides an example of the construction of an integrated health care delivery system across all sectors. Second, KM treatment, such as acupuncture, is inexpensive and not significantly affected by a shortage of material resources in disaster situations. Furthermore, KM personnel can be employed in the national disaster management system in the context of utilizing existing support systems to increase the national capacity to respond to disasters.

**FIG. 4.**
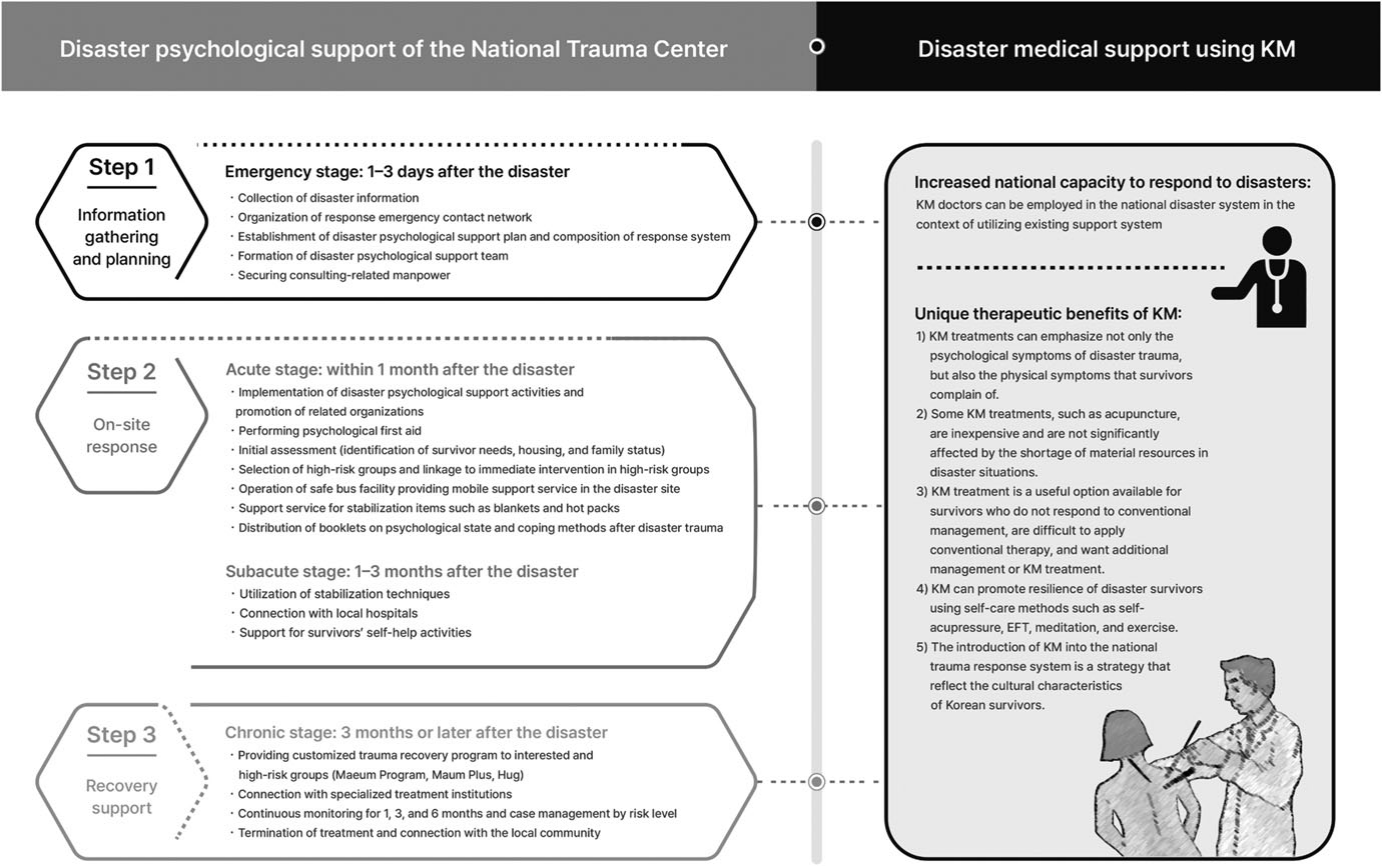
The potential benefit of introducing KM into the national trauma response system. KM, Korean medicine.

Therefore, this advantage of KM may contribute toward the construction of a sustainable national trauma response system. Third, KM treatment is a useful option for survivors who do not respond to conventional management, consider conventional therapy to be difficult to apply, and want additional management or KM treatment. KM promotes the resilience of disaster survivors using self-care methods, such as self-acupressure, EFT, meditation, and exercise. Moreover, the utilization of KM reflects the cultural characteristics of Korean survivors. Therefore, KM treatment might empower individuals and communities affected by disasters (Fig. 4).

## Conclusions

This manual provides guidance on disaster medical support using KM and integrative medicine for disaster survivors. The KM modalities mentioned in this manual have strengths such as feasibility, safety, and cost-effectiveness in disaster medical support. It is expected that this manual will help KM doctors to be effectively utilized as medical resources at disaster sites. In addition, this manual would provide a good model that can be extended to countries that wish to use integrative medicine for disaster support to implement the commitments of the Declaration of Astana.

## Supplementary Material

Supplemental data

Supplemental data
