# Supplementary material for: Development of a Manual for Disaster Medical Support Using Korean Medicine for Disaster Survivors
Source: J Integr Complement Med. 2023 Jun 6;29(6-7):395–407. doi: 10.1089/jicm.2022.0561 (PMC10280179; doi:10.1089/jicm.2022.0561)
Supplement: Supplemental data [file Supp_DataS1.pdf]

Practice guideline for the treatment of Disaster-related trauma using oriental medicine (proposal)

Sang-Ho Kim Department of Neuropsychiatry of Korean Medicine, Pohang Korean Medicine Hospital, Daegu Haany University

Chan-Young Kwon Department of Oriental Neuropsychiatric, Oriental Hospital, Dong Eui University

Joo-Hee Seo Oriental Neuropsychiatric Clinic, Department of Oriential Medicine, National Medical Center

Contents

Preface

I. Introduction
1. Purpose
2. Objective
3. Target Subjects
4. Reactions of the survivors of a disaster event
5. National Disaster Psychological Support System
6. Organizations

II. Treatment Protocol
1. Treatment algorithm
2. Manual for history taking and medical examinations
3. Treatment protocol by phases
4. Treatment protocol by symptoms
5. Psychological First Aid Treatment
6. Other considerations

III. Treatment Methods
1. Ear Acupuncture
2. Acupuncture
3. Herbal Medicine
4. Emotional Freedom Technique (EFT)
5. Stabilization Techniques
6. Self-care methods

IV. Appendix

Preface

There is a date that I am unable to forget and is familiar to me as my date of birth. 20171115... On November 15, 2017 at 14.29 p.m. a 5.5 magnitude earthquake occurred in Pohang, Korea. It feels as if this Pohang earthquake has struck just few days ago, but four years have already passed since the incidence. When I was first preparing for a project for the research foundation, I came across a newspaper article reporting that even after a year after the event, many victims of the earthquake are still living in the shelters. These victims are still suffering from insomnia, depression, pain etc. due to the trauma from the earthquake and prolonged stays at the shelters. This has drawn my attention to disaster-related trauma and led me to conduct a research in the field. We organized a medical team and provided medical support including ear acupuncture treatment to the earthquake victims with the support of the Disaster Psychological Support Center.

Disaster psychological support is mostly focused on psychological interventions. However, the disaster survivors (the term survivor is preferred over victims in disaster medicine.) report not only psychological symptoms but also multiple physiological symptoms such as insomnia, pain, fatigue etc. Moreover, when there are large number of disaster survivals after a large scale disaster, it is difficult to provide immediate disaster psychological support with limited human and physical resources. Long-term psychological support can also lead to burn-out syndrome in the psychological support providers. Hence, effective, safe and economical non-pharmacological and non-psychological interventions are required in urgent disaster sites.

Ear acupuncture is a simple non-pharmacological, oriental treatment with very little side effects, and is already used widely in various disaster sites around the world. Doctors specializing in oriental medicine can provide not only oriental treatments such as herbal medicine and acupuncture, but also give substantive help using mind-body interventions such as meditation, emotional freedom technique, stabilization technique etc. Moreover, with the use of acupressure and ascetic training, we can actively support self-care by the survivors.

Unfortunately, the current Korean disaster support system does not officially provide oriental treatment. Hence, so far the oriental treatments are only provided in forms of independent medical volunteer at national disaster sites. Also, there is a lack of systemized or organized evidences for application of oriental treatment at disaster sites, and no standardized manual for oriental medicine doctors to provide medical supports at disaster sites. Therefore, our research team has put together this manual.

We hope that this manual can serve as a guideline to help oriental medicine specialists at the disaster sites, and to become an essential foundation for the oriental medicine doctors to contribute to national disaster-related trauma responses and other fields of public health. We offer our sincere words of thanks to professor Chan-Young Kwon and Joo-Hee Seo for their support in the development of this manual, and the advisory

members for their thorough review.

Principal Investigator Sang-Ho Kim
From Pohang Office

Review Committee (Accreditation Committee of Korean Society of Oriental Neuropsychiatry)

Kim Jon g-Woo	Chairman / Head professor at Department of oriental neuropsychiatric, College of Korean Medicine, Kyung Hee University / Director of Korean Medicine Mental Heatlh Center
Seo Jin-Woo	Member / Professor at Department of oriental neuropsychiatric, College of Korean Medicine, Sangji University
Seo Hyo -Won	Administrative assistant / Research professor at College of Korean Medicine, Kyung Hee University / Oriental neuropsychiatric specialist
Lim Jun g-Hwa	Member /Professor at Department of oriental neuropsychiatric, College of Korean Medicine, Pusan University
Jung Jin-Hyun	Member /Head of medical department at Jangdeuk Korean Medicine Hospital / Oriental neuropsychiatric specialist
Choi Seong-R l	Member /Professor at Department of oriental neuropsychiatric, College of Korean Medicine, Gachon University

Advisory Committee

Kwon Ho-In	Professor at Department of Counseling Psychology, Jeonju University
Kim Keon-Hyung	Professor at Department of Acupuncture & Moxibustion Medicine, School of Korean Medicine, Pusan National University
Kim Dong-Soo	Professor at Dongshin University oriental medicine college, department of preventive medicine
Kim Ryun-Jae	Director of Nam-Ee-Kyung Clinic
Kim Sung-Tae	Professor at Daegu Hanny University, Oriental medicine college, department of Sasang constitutional medicine
Kim Yoon-Na	Clinical Professor at Kyunghee medical center, oriental medicine clinic, department of oriental neuropsychiatry
Kim Eun-Jung	Professor at Dongguk University, Oriental Medicine College, Department of acupuncture
Park Jong-Hoon	Director of Ansan Ja-Saeng Oriental Medicine clinic / Oriental neuropsychiatry specialist
Shin Cha-Seon	CEO of Peace People Organization / Doctor of Psychology
Lee Bo-Ram	Senior Researcher at Korea Institute of Oriental Medicine
Lee Seung-Hwan	Director of Sol-Sol clinic / Oriental neuropsychiatry specialist
Lee Yoon-Gyu	Professor at Daegu Hanny University, Oriental Medicine College, Department of acupuncture
Lee Jeong-Hwan	Director of Hye-Min-Seo Clinic / Head of EFT Korea & Society of Sandstone acupuncture
Lee Jin-Hee	Director of Da-Ee-Room Clinic / EFT Korean Master Trainer
Lim Jeong-Tae	Research professor at Wonkwang University / Doctor of oriental medicine
Jung Seon-Yong	Professor at Department of neuropsychiatric, College of Korean Medicine, Kyung Hee University
Cho Seong-Hoon	Professor at Department of neuropsychiatric, College of Korean Medicine, Kyung Hee University

<Contents of the Manual >

This manual is composed of **I. Introduction, II. Treatment Protocol**, **III. Treatment Method, IV. Appendix**.
In the **I. Introduction** section, 1. Purpose, 2. Objective, 3. Target Subject were proposed to define the scope of this manual. Also, in order to understand the reactions of the disaster survivors, 4. Reactions of the survivors of a disaster event was introduced and finally the 5. National Disaster Psychological Support System was introduced.

Next in the **II. Treatment Protocol,** the 1. Treatment Protocol for oriental medicine doctors to examine and approach disaster survivors, 2. Manual for history taking and medical examinations, 3. Treatment protocol by phases, 4. Treatment protocol by symptoms, 5. Psychological First Aid Treatment, 6. Other considerations were proposed.
In the manual for medical examinations, detailed approach and evaluation tools for medical examinations at disaster sites were proposed. Then different approaches for each phases of disaster (acute/sub-acute/chronic) were proposed, since the reponses differ by disaster phases. Morever, the major symptoms of complaints by disaster survivors were categorized into psychological symptoms (anxiety/fear/depression/anger) and physiological symptoms (insomnia/ dyspepsia /exhaustion/migraine and dizziness/pain), and different protocols were proposed for each category to make on site management more effective and convenient. Accupressure methods to help the self-care by the disaster survivors were also introduced. A treatment algorithm for quick understanding of the overall treatment process was proposed. This was followed by an introduction of psychological first-aid to be conducted at disaster relief site prior to the medical examination, and lastly other considerations for providing disaster medicine were explained.

In **III. Treatment Methods**, details of each treatment and management methods used during medical examinations were introduced for future references. Ear acupuncture, herbal medicine, acupuncture, emotion freedom technique and stabilization technique were proposed, and detailed training materials on different coping mechanisms by symptoms, acupressure, ascetic training, meditation etc. for self-care were explained.

I. Introduction

1. Purpose
2. Objective
3. Target Subjects
4. Reactions of the survivors of a disaster event
5. National Disaster Psychological Support System

I. Introduction

1. Purpose

Disasters occur regularly and are part of our lives. Natural disasters due to climate changes such as flood, typhoon, drought, forest fire and earthquake, and human-caused disasters such as terrorism, accidents, wars, nuclear have been taking places continuously all over the world, and even now there is an infectious disease disaster, the coronavirus disease-19, that is ongoing. Disasters cause various severe, large-scale damages over a short period of time, and are difficult to recover from. Since disasters have especially bad psychological influences on the survivors, such as as depression, posttraumatic stress disorder (PTSD) etc., a national level disaster psychological support system is necessary.

Current disaster psychological support system is focused on psychological interventions such as cognitive behavior therapy, exposure therapy, Eye Movement Desensitization and Reprocessing (EMDR) etc. However, the survivors not only report psychological symptoms but also various physiological symptoms including insomnia, pain, exhaustion etc. Moreover, immediate and effective psychological support is difficult when there are large number of survivors and refugees, and limited human and medical resources. Long-term psychological support can lead to exhaustion (burnout) of the providers. Hence, an immediate and effective intervention that can complement the existing psychological support and be performed over long-term is required.

Ear acupuncture is a representative non-pharmacological intervention in oriental medicine. It is simple to operate and has little side effects, which makes it suitable for use in disaster site, and there are many evidences on its effectiveness. Herbal medicine and acupuncture can be applied to various physiological/psychological symptoms and have less side effects and less dependency compared to psychiatric drug treatment. Emotional Freedom Technique (EFT), recently registered as the new Korean medicine health technology, can also be used effectively at disaster sites.

Even though all possible human and physical resources should be utilized in disaster medical care support, the medical support from oriental medicine doctors are limited to forms of independent volunteer activities and are yet to be included in national support system. There are no guidelines for oriental medicine doctors on the disaster trauma

sites to follow, and no cooperative treatment manual on applying oriental medicine. Hence, this research team developed a manual on oriental medicine care for disaster related trauma. We hope this manual will help the oriental medicine doctors to provide more active and systematic medical services on disaster sites. Furthermore, we hope it can lead to a more effective and improved national disaster trauma responses in the future by contributing to establishment of cooperative medical treatment system involving oriental medicine.

2. Objective

The objective of this manual is to guide and support oriental medicine doctors in their examinations and treatments of psychological, physiological and behavior stress responses of the survivors after disasters (symptoms of disaster-related trauma). Moreover, the manual involves educating the survivors on self-care techniques to manage their symptoms of trauma.

3. Target Subjects

This manual targets survivors with symptoms of disaster-related trauma (primary victims), and can also be applied to families, relatives and close acquaintances of the survivors, disaster service workers and volunteers that were involved at disaster sites, (fire fighters, police officers, rescue workers, doctors, nurses, social workers, psychological support staffs, clergy, government officials etc.), residents of the disaster area, people who have indirectly experienced trauma from mass media etc. that report secondary trauma.

Table1. Target Subjects of Manual

• When subjects report symptoms of disaster-related trauma due to psychiatric damages after experiencing disaster
• Disaster survivors, families of disaster survivors, support staffs at the disaster site
• All general citizens and residents of regional community at disaster sites.
• Disaster survivors who received psychological support such as stabilization technique, trauma-focused cognitive behavior therapy, EDMR etc. or took psychiatric drugs such as antidepressants etc., but have not shown improvements of symptoms or do not prefer those treatments.
• Survivors who are receiving psychological support and wish to receive oriental medicine treatment in parallel.
• Subjects who did not receive psychological support and wish to receive oriental treatment.

• When disaster psychological support center or associated institutes request for oriental medicine treatment.

• However, subjects with severe hyper vigilant or psychotic symptoms and risks of harming others or themselves should be excluded. For such patients, psychiatric drug interventions or professional psychological therapy should be considered first.

4. Reactions of the survivors of a disaster event

For effective treatment, understanding reactions of the survivors at the time of disaster, characteristic symptoms of disaster-related trauma and the reactions by different phases are necessary. Moreover, since many disaster survivors are already under national disaster psychological support system, understanding of the process of the support system is also required.

When experiencing life-threatening disasters or trauma directly or indirectly, most people show various reactions in physiological, cognitive, emotional and behavioral areas. Post-disaster reactions may appear immediately or after some time. However, most of the survivors are not aware that such reactions to disaster are natural and normal, and find it difficult to accept the changes. Hence, education on the types of reactions that may appear after disaster experiences is essential. Such acute stress reactions may differ based on the severity of the disaster, individual characteristics (sex, age, psychiatric history, past experiences of trauma etc.), and socio-cultural background.

1) Acute Stress Reaction

Physiological, emotional, cognitive, behavioral, spiritual changes and symptoms frequently seen in disaster survivors are as follows[12].

Table 2. Symptoms of Disaster Survivors by Category

Category	Symptoms
**Physiological change**	Increase in heart rate, increase in blood pressure, shortness of breath and perspiration.<br>Twitching lip, stomachache, increased or reduced appetite, dyspepsia, nausea, chill, insomnia or nightmares, hypersomnia and fatigue, asthenopia, migraine, pain in the upper stomach or other parts of the body.
**Emotional change**	Feels multiple overwhelming emotions such as suffering, horror, hurt, victimized, angry, guilt, unhappy, restless, fear.<br>Fear of going insane.

	Regressive behaviors such as excessive clinginess, fear, irritation, perspiration during sleep, thumb-sucking etc. may appear in children.
**Cognitive change**	Become shortsighted and rigid thinking<br>Find it hard to concentrate and feel absent-minded and confused.<br>Experience dissociation, and absence of feeling toward everything.<br>Find it hard to communicate and in worst cases orientations in person, time and spaces become impaired.<br>Images related to the event keeps coming to mind and experience flashbacks.<br>A lot of people feel their faith in the world is shaken.<br>Think there is no one having a hard time as they are
**Behavioral change**	Behaviors become rigid, easily become angry and find scapegoat or someone to blame. Difficult to communicate, feel helpless and restless.<br>Often show aversive reactions to places and people associated with the event, and may become socially isolated.<br>May take excessive alcohol, smoking, drugs, anti-anxiety medications, painkillers etc. to cope with stress.
**Spiritual change**	Some people try to find meaning in the disaster events and some become become resentful and lose hope.

However, when the following dissociative reactions continue, the risk of developing post-traumatic stress disorder is high, and a special management is required such as referral to professional mental health[3].

• Shows no emotional reactions.

• Appear absent-minded and show difficulty.

• Loss of reality. Reality does not feel real.

• Feels detachment of mind and body, and confused about self-identity (Depersonalization).

• Cannot remember important parts.

2) Phases of disaster response

After the trauma from disaster, following phases of reactions appear during the recovery process, and psychological supports for each phases should be given[4]. The periods after the diaster can be divided into acute phase (3~7days after the accident), sub-acute phase (within 1~3months), chronic phase (after 3 months) according to the time passed[5]. Such changes are normal reactions to overcome the trauma of the disaster, but in certain people these changes can become chronic and cause mental disorders.

(1) Phases of responses during recovery process

① Shock phase (0~48hours after the incidence)

This is a phase that comes immediately after the incidence, in which the survivors experience fight or flight response and despair or become petrified. Symptoms of activated sympathetic nerve system occur. Right after the incident, the survivors are in the state of shock and do not perceive what is happening around them as reality. They observe their surroundings as movies or other people's businesses. Most people act based on survival instincts and become short-sighted with decreased ability to concentrate and think logically. Behaviors become rigid and communication with other people becomes difficult. Emotions such as distrust, insensitivity, fear, confusion etc. become strong.

② Acute phase (Rescue phase)

Classified into heroic phase (within 3~7days after the incidence), honeymoon phase (within 3~7days), Disillusionment phase (7days~years).

Heroic Phase

During the rescue phase, people recover from the shock and participate in rescue activities to help others. Cooperation's and heroic acts sometimes appear among the rescue staffs, families and neighbors. The survivors feel happy to be just alive and feel confident enough to do anything.

Honeymoon Phase

As social attentions become focused on the survivors and the region of disaster, and the mass media report rescue stories and diaster experiences, the survivors feel at the center of the world. The survivors become full of energy and physiologically aroused and active.

Disillusionment Phase

As the time pass by, the attentions on the disaster site decrease and the survivors experience various stipulations and limitations in receiving the expected compensations and rewards. They feel disappointed and discouraged as the disaster damage recovery is delayed. Disillusionment phase starts 7~8 days from the time of disaster, and continues for 7~8 weeks or even for years. The survivors realize that many things they have lost through the disaster can never be recovered, and feel deep disappointment and sad. Physical energy become depleted and exhaustion surges. The aftereffect of broken daily rhythm hit and the survivors become physically depleted. They feel helpless,

overwhelmed by multiple emotions, fear going back to the incidence site and suffer from nightmares, anxiety and insomnia. Moreover, they are tense, easily surprised, stiff, shaky and keep getting flashbacks of the disaster site. Survivors become easily annoyed and depressed, wishing to be alone. Sometimes they suffer from guilt of surviving. They experience sudden changes in emotion, such as suddenly bursting into tears, and feel helpless and, loss of control and worry about other people’s negative judgment. Survivors feel enraged at the people and the world that such event took place and they were unprotected and misunderstood, and try to keep distant.

③ Working Through Grief (1~4weeks after the incidence)

Uncomfortable symptoms and denial take turn to appear. Experience stimulation of sympathetic nerve system (exaggerated startle response, hypersensitivity, insomnia, nightmares etc.) and unexpected emotions. As the recovery process nears ends, survivors deny disaster trauma and show increased hospital visits due to physiological symptoms such as fatigue, dizziness, headache, nausea etc. Anger, hypersensitivity, indifference and social withdrawal are common as well.

Stress response of rescue phase continues, but the level of reaction decreases and the survivors begin to control their painful emotions and thoughts. Interests in daily lives grow little by little and begin to plan for the future. Survivors begin to recover physically and emotionally, as the architects and roads destructed during the disaster also begin to be recovered. There are certain disappointing moments, but as the efforts to recover yield small to large success, they begin to regain the motivation for life.

④ Reconstruction phase (2weeks~2years after the incidence)

The amount of time it takes to reconstruct varies from person to person. The stress response no longer appears and the survivors accept the disaster and integrate it into their lives. This process depends on the individual's ability to cope and the social support they receive. Those who still have difficulties at this stage should be referred to a professional treatment and managed continuously.

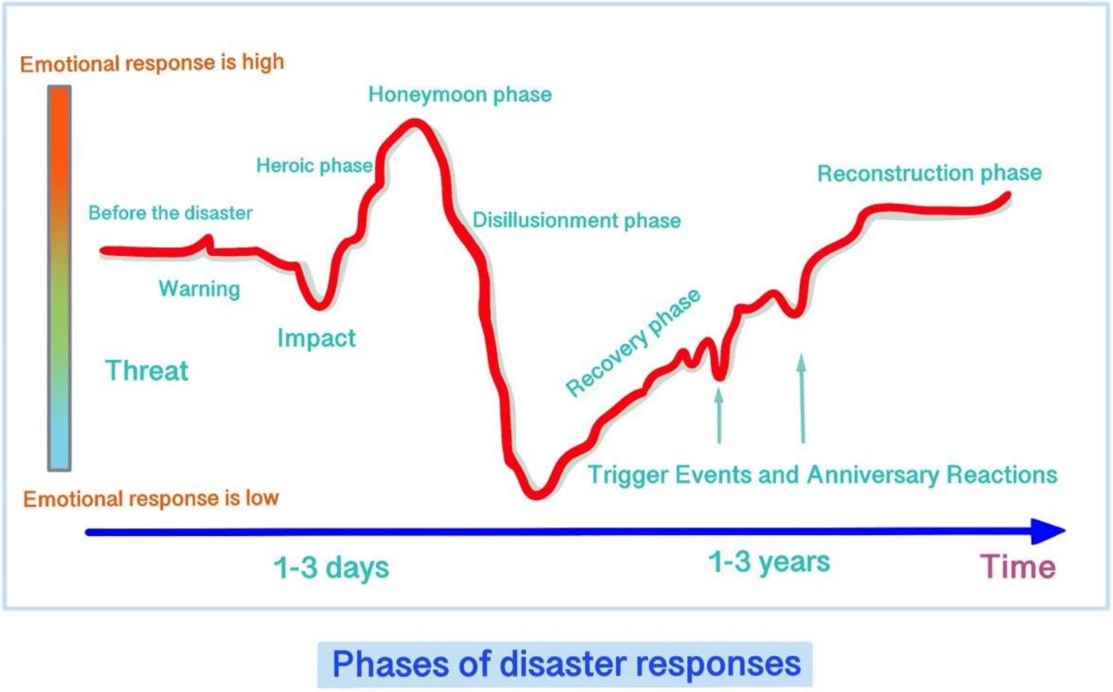

Figure 1. Phases of disaster response[6]

(2) Responses by time period after disaster

① Acute phase (Day 3~7 after the incidence)
This is the phase where the survivors are at a devastated state immediately after a disaster, and show reactions to protect themselves from the shock of severe trauma. A state of being absent-minded, poor judgment, feeling unfamiliar with the surrounding environment and people, and a state of being in a trance may appear. Such repression, denial, and dissociation immediately after the outbreak are a natural response, but if they persist, they can lead to the development of post-traumatic stress disorder.

② Sub-acute phase (Within 1~3 months)
Hyper arousal: Extreme fear and anxiety appear, and the survivors are afraid that another disaster may occur. They experience the fear of death when awake and may have recollections and nightmares about the accident. The scene of the shocking accident may appear as flashbacks. Hence, the survivors tend to avoid places or people etc. associated with the accident. They become easily startled by even the small stimulus and sensitive and get angry often. Symptoms such as tension in the neck, stiff posture, dry mouth, unstable pupils, increased heart rate, shallow and uneven breathing,

cold hands, pale skin, weakness in the knee and legs, clenched hands, abdominal discomfort, nausea, cold sweat in the hands and forehead, crying out of fear etc. may appear.

Depression/Grief/Guilt: Depression and grief appear due to the deaths of families, relative's friends, injuries from accidents, economic and social damages from the accident etc. Survivors become depressed, lose motivations, and complain of insomnia, fatigue, loss of appetite and become immersed in negative thoughts. They feel the life is meaningless and feel emotionally detached from other people, making interpersonal relationship difficult. In severe cases, they cannot feel or expression any emotions and experience mood swings. Survivors exaggerate, blame themselves, and suffer from self-criticism about their own survival and their actions and responsibilities at the time of disaster.

Sometimes they hid their guilt in fear of being criticized by the others. There is high possibility of developing PTSD as the survivors feel guilty and refuse to ask for help to others.

Distrust/Isolation: Has extreme, negative opinions about others and self, and believe no one can be trusted. Express high sense of refusal toward sympathy from families, acquaintances etc. The survivors believe that nobody can understand what they have experienced, form their own group and express anger at the others. They view the world as a dangerous place. They are unable to express their pain to others and feel isolated and alienated. The feeling of isolation may be more severe and the adjustment can be difficult if the survivors return to their home or workplace early after hospitalization or out-patient treatment.

Changes in cognitive functions: Symptoms of dissociation, damages in integrative functions such as consciousness, memory, identity, environmental perception etc., may appear. Especially, loss of memory, memory decline, perceptual disorder and decreased concentration may appear.

Physiological symptoms: The disaster survivors often complain of fatigue, migraine, loss of appetite, dyspepsia, defecations problem, insomnia, pain, decreased immunity etc. due to the stress response from the shock of disaster. The physiological pain exacerbate the psychological symptoms such as pain, anxiety, depression guilt etc., and limit the activities, delay getting back to the social life, and decrease the quality of life.

Substance Dependence: The amount of drinking, smoking, psychotropic drug use may increase to escape from physiological pain. However, continued substance abuse and

dependence can deteriorate one's health, damage family relationship and cause social issues. When accompanied by addiction, the trauma and symptoms of depression may deteriorate.

③ Chronic phase (3 Months or over)

The anxiety, nervousness and rage of the survivors continue to be uncontrolled. The survivors become nervous about their symptoms not improving, and how they will not be able to get back to their daily lives before the accident. They become skeptical about the treatments and the depression worsens. They may even lose hope in recovery and despair or attempt suicide. The mourning reactions may also continue. Isolation and distrust deteriorate as other people's understanding and attention decrease. Since the legal problems such as indemnifications etc. cannot be solved in short-term, the conflicts, anger and depression may worsen. Dependence on alcohol, painkillers and tranquilizer may become more severe.

(3) Post-disaster Mental disorders

According to the World Mental Health, the incidence rate of PTSD in the high-risk population was very high with 44.5%[7]. Acute stress disorder appears more frequently right after the disaster, and active interventions (psychological treatment, pharmacotherapy) are required to prevent progression into post-traumatic stress disorder. There are other trauma associated disorders other than post-traumatic stress disorder; major depressive disorder, substance use disorder, generalized anxiety disorder, adjustment disorder, somatization, head trauma, toxic substance, disease and secondary organic mental disorder due to dehydration.

Stress factors such as complex bereavement from death of family, indemnification issue related with disaster recovery, stress factor such as unemployment can influence the development of such mental disorders[8]. Major depressive disorder and substance abuse are often observed together with PTSD, and can increase death rate by leading to dangerous behaviors such as car accident, dangerous sexual behaviors, domestic violence etc.

PTSD and major depressive disorders are not normal reactions to disaster related trauma. They are serious diseases that need treatment. The key to detecting PTSD is definite symptoms of avoidance and insensitivity. Even after being diagnosed with PTSD, additional mental evaluation should be taken, since PTSD is normally accompanied by other mental disorders and sometimes other mental disorder may occur without PTSD. Diagnosing other mental disorders accompanied by PTSD is as important as diagnosing the PTSD for effective treatments and recovery. Women are more vulnerable to major depressive disorder whereas men are more prone to addiction problems.

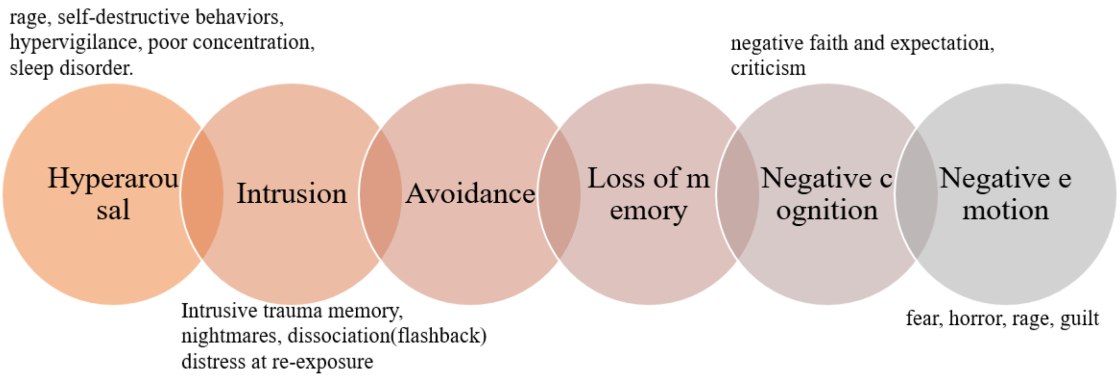

Figure 2. Diagnostic criteria for post-trauma stress disorder [9](DSM-5; Refer to the appendix)

Table 3. Post-disaster mental disorders and psychological, physiological, behavioral symptoms.

Mental disorder	• Post-trauma stress disorder, acute stress disorder, major depressive disorder, substance use disorder, generalized anxiety disorder, adjustment disorder, somatization disorder,<br>• Secondary organic mental disorder from Head trauma, Toxic substance, Disease, Dehydration
Psychological, Physiological symptoms and behavioral problems.	• Anger, irritation, distrust, sadness, anxiety, fear<br>• Sleep disturbances<br>• Increased alcohol, caffeine, smoking<br>• Family conflict<br>• Pain from economic damage due to loss of job<br>• Physiological symptoms(Insomnia, Fatigue, upper abdominal pain, Dizziness, headache, Musculoskeletal symptoms)
Bereavement and Grief process	• Prolonged grief disorder<br>• Complex bereavement disorder

(4) High risk population during disaster

Post-traumatic stress disorder mostly occurs in the primary survivals with direct experiences of the disaster, and more intense the stress of the trauma, the more mental problems occurs. People who have observed the disaster directly or experienced

physical danger are more vulnerable to trauma, and the people whose attachments with the primary survivals were clear such as the early rescue staffs, volunteers should also be cautious of trauma. People who have been psychologically susceptible even before the disaster also suffer from fear, unemployment, excessively long commuting hours, damaged interpersonal relationship, lack of social support etc.

Table 4. At-risk population during disaster

At-risk population during disaster
Direct observer of the disaster
Victims with physical injuries
Early rescue staffs
Those who have experienced bereavement
Single-parent
Children
Senior citizens
Women
People with past post-traumatic stress disorder
People with past experiences of trauma
People with mental disorders or severe chronic diseases (in the past or current)
People with absence of social support

5. National Disaster Psychological Support System

1) Guidelines on disaster response

There are 3 major keys; subject evaluation, severity assessment and treatment, that frame the core of disaster mental health response. The first job is to differentiate mental disorders such as PTSD and major depressive disorders from the general pain. Symptoms need to persist for at least 1 month to be diagnosed with PTSD. Pharmacological treatment and psychological treatment are the two effective treatments for mental disorders in disaster situation[10]. For the survivors who complain of mild to moderate symptoms and are not diagnosed with PTSD or other mental disorders, warm and practical support can be of help. One of such support is the psychological emergency service, which can be performed in various situations by not only the professionals but also by the trained general population. Psychological emergency

response was developed by the consensus among professionals, and has been used widely as a treatment during acute phase since the 9·11 incidence. However, its effects have not been sufficiently validated[11]. Many survivals show threshold symptoms following the disaster, so practical supports and helps are necessary.

When assessing the trauma of the disaster, the examinations should not diagnose the survivors based on the level of exposure to trauma alone. Moreover, it is not appropriate to diagnose the disaster survivors with PTSD only based on the self-assessment survey for PTSD. People with history of mental disorders, serious personal or social difficulties, and socially isolated people are very likely to develop chronic mental disorder and should be observed carefully.

PTSD symptoms begin immediately after the disaster and can easily become chronic, so early interventions are preferred. During the early phase of response, there is large scale of victims, so the response capacity may be temporarily paralyzed. After multiple months, the mental disorder become more permanent and the survivors require more help. Although the survivors require continuous support, the attention and the help from the others decrease over time. Moreover, although the symptoms of PTSD appear right after the disaster, many survivors seek psychological support too late or fail to receive appropriate treatments.

2) National disaster psychological support system

Disaster psychological support is managed by the Center for disaster psychological recovery support (under Ministry of the Interior and Safety) and National Center for Disaster Trauma (under National Center for Mental Health/Ministry of Health) as of October 2021. Together with the local government bodies (city, state), they select and support the centers for disaster psychological recovery support. National center for disaster trauma has been established and operated in the national center for mental health and managing the disaster psychological support at times of large-scale disasters since 2018. The National Center for Disaster trauma establish response system for disaster mental health, train and educate professionals for disaster mental health, conduct R&D to advance disaster mental health service. At the time of national disaster, they cooperate with relegate associations to quickly establish a uniform intervention platform to provide emergency response services on-site and trauma treatment program for mental health high risk group[12].

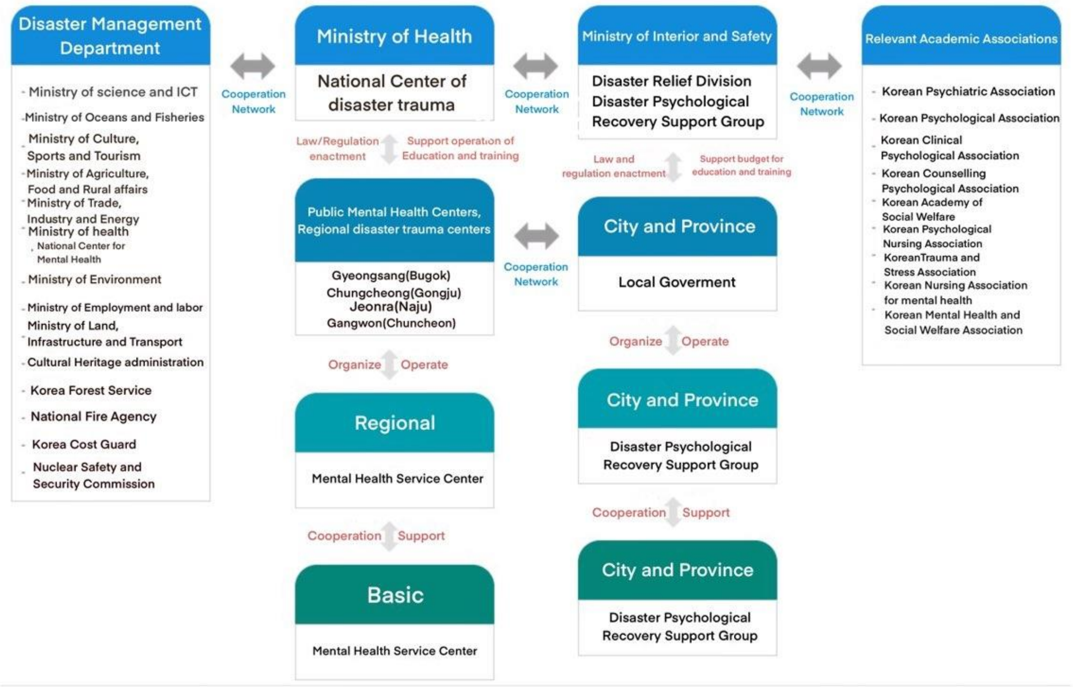

Figure 3. Disaster psychological support operation system (Plan) (National Center for Disaster Trauma Website)

3) National disaster psychological support[13][14]

Table 5. Levels of Volunteer activities for National psychological support

Disaster Response	Level 1. Data Collection	Shock phase (Right after the disaster)	• Collect information of the disaster<br>• Activate emergency contacts for response
	↓		
	Level. Planning	(Right after the disaster)	• Diaster situation monitoring<br>• Establish a plan for disaster psychological support plan and form response system (Form a psychological support team within the local disaster psychological support group)<br>• Secure relevant authorities (cooperation organization and advisory team), associated local institutions, academic society and human resource for consultations.
	↓		

	Level 3. On-site Response	Acute phase (within 1 month)	• Implement disaster psychological support activities and advertise relevant institutions.<br>• Conduct psychological emergency responses (PFA)<br>• Early evaluation (Demands from survivors, status of their residence and family)<br>• Select high risk population and involve immediate emergency response for the high risk group.<br>• Operate safe bus<br>• Provide material support such as blanket, hand warmer etc.<br>• Provide brochures on psychological status and the appropriate coping methods after trauma.
		Sub-acute phase (within 1~3months)	• Use of stabilization technique<br>• Hospital-clinic liaison.<br>• Support self-care activities of the survivors
	↓		
Recovery after the disaster	Level 4. Recovery support	Chronic phase (After 3 months)	• Provide customized trauma recovery program for caution group and high risk group (Maeum program, Maeum Plus, HUG)<br>• Liaison with professional treatment institute.<br>• Monitoring at 1,3, 6months and continued case observations for each level of risk<br>• Closing of treatment, liaison with local community.

• Level 1: Shock phase (Right after the diaster). Data Collection

- Monitor for potential disaster incidence continuously, and when there is high potention of disaster occurrence, providing relevant information on disaster psychological health and preparation activities are recommended. Level 2: Shock phase (Right after the diaster). Planning

- Provide mental health recovery guide and psychological safety items to the disaster survivors. Provide on-site response guide to the disaster psychological support

workforce.

Level 3: Acute phase (within 1 month) and Sub-acute phase (within 1~3months). On-site response

- Notify the relevant local institutes of the disaster psychological support activities, and request for spread of information to the survivors.
- Perform psychological emergency response (PFA).
- Stabilization technique: Body action program for Mental-body stability (Focus on the physiological stabilization such as massage etc.)
- Select the high risk group and refer to the professional treatment center if necessary.
- Liaison with clinics and hospitals: The survivors require various medical supports due to multiple physiological and psychological symptoms, but have difficulties finding hospitals by themselves. Hence, the case managers need to become aware of the situation and connect the subjects to appropriate hospitals.
- Operate on-site counseling centers together with safe bus. In safe bus, evaluate stress level through psychological surveys and heart rate variation analysis, and provide individual comprehensive consultations
- Support self-care activities by the survivors: Provide opportunities for the survivors to hold self-care gathering by themselves. Even for professionals, there is limit to using the external manpower, and it is essential that the local community strengthen their own professional manpower to assist self-care activities continuously and help the survivors.
- Stay together with the survivors to form trust relationship.
- Provide services to provide practical items such as blanket, hand warmer etc. and fo od
- Provide leaflets or brochures on the psychological condition after trauma
- Cooperate with PR, briefing and coverage's
- Open education sessions to train civilian assistants.
- If the survivors are staying at their own houses instead of at the shelters, outreach to meet the survivors is important.

• Level 4: Chronic (After 3months). Recovery support

- Continue to manage the survivors by visiting, calling and sending mails once a month to twice a week depending on the level of risk for each case.
- Select high risk group through early assessment and counseling.
- Establish a plan for customized trauma recovery program.
- Support period is 12 months maximum.
- Provided Programs

① Maeum program: Stabilization technique focused recovery program to help the survivors control stress level

② Maeum PLUS: A recovery program based on the Skills of psychological recovery

(SPR), a evidence-based module practice developed by the American National Center for PTSD and National Child Traumatic Stress Network, to help manage stress effectively after the disaster.

③ Hug program: Psychological treatments to induce reconstruction and reprocessing of traumatic memory (prolonged exposure therapy, eye movement desensitization and reprocessing, Narrative exposure therapy)

- After the completion of the recovery program, measure the effects through post-evaluation and conduct follow-up through monitoring every 1, 3, 6 months intervals.

II. Treatment Protocol

. Treatment algorithm
2. Manual for history taking and medical examinations
3. Treatment protocol by phases
4. Treatment protocol by symptoms
5. Psychological First Aid Treatment
6. Other considerations

1. Treatment algorithm

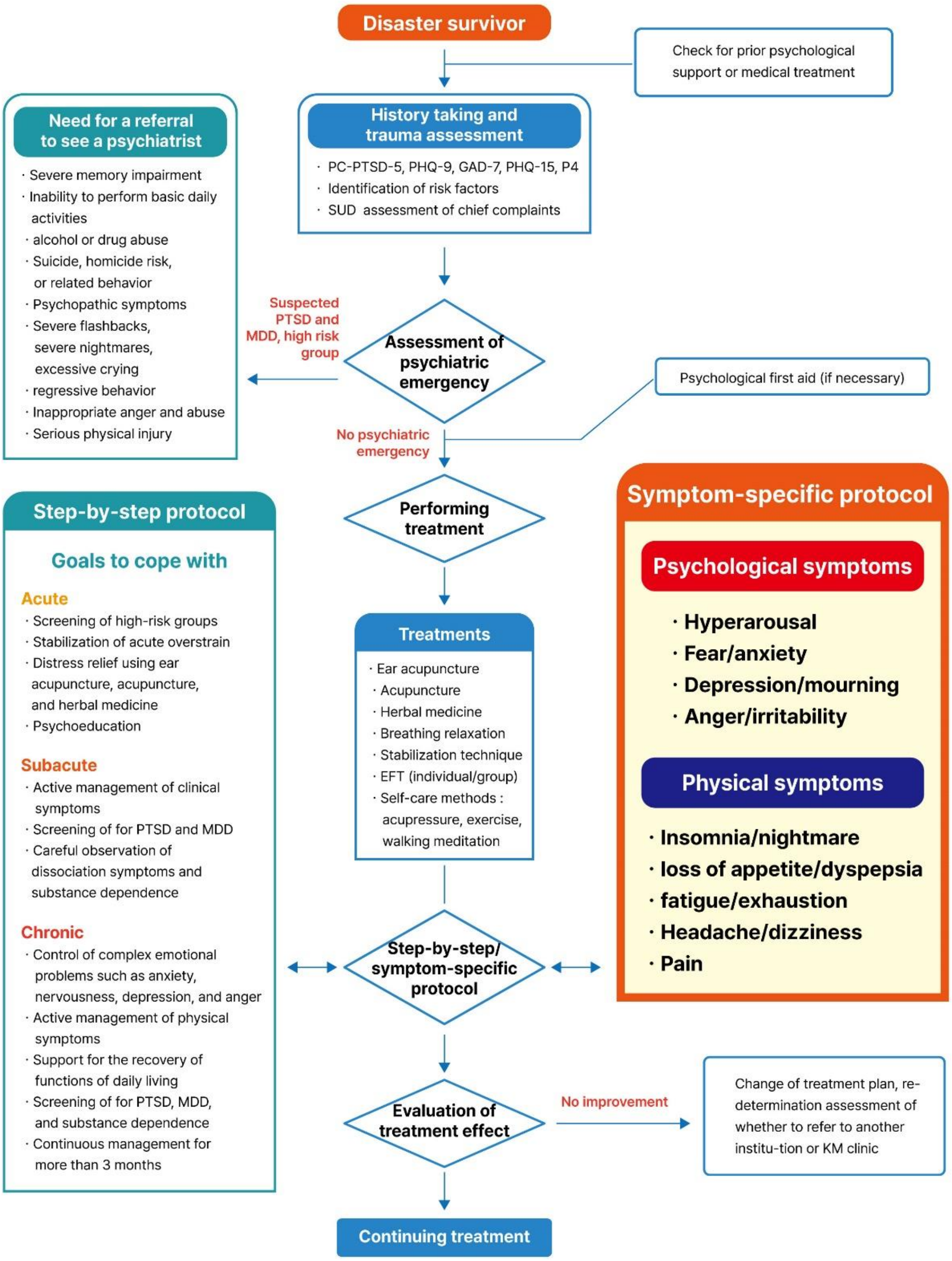

Figure 4. Oriental Treatment algorithm for disaster related trauma.

2. Manual for medical examination

1) Treatment guideline for the disaster site[15]

Behavior changes in the disaster survivors are normal responses to a sudden, unexpected situation of disaster. Hence, it is important to accept such changes as normal behavior pattern during medical examination and to not make any rash diagnosis.

○ Speak slowly. Use simple yet detailed words. Do not use technical terms.

○ Do not make assumptions about what the survivors have been through and what sort of situation they are at.

○ Do not make any rash diagnosis. Most of the acute reactions are normal responses.

○ Do not focus on the helplessness, weakness, mistake or faults of the survivors.

○ Getting detailed information about trauma is not the objective, so caution should be taken not to forcefully rein cite the memories of trauma.

○ Offer only the accurate information and in case of uncertainty, do not deduce and express uncertainty honestly.

○ Help the survivors feel safe and think that the life is predictable and they are in control, to help them recover from disaster.

- To secure physiological and mental safety, keep reminding the survivors that they are now safe.
- Explain the overall treatment process to make them understand that the treatments will be performed as predicted.
- Remind the survivors that the treatment process can be modified based on their choices

2) Basic Medical Examination

First, obtain consents from the survivors for medical examinations and then proceed. General medical examinations and trauma assessment are performed in the following orders: ① Sociodemographic inquiry, ② screening of trauma, ③ Basic medical examination, ④ Comprehensive medical examination, ⑤ Risk factor examination ⑥

Examination from oriental medicine aspect, ⑦ Classification of chief complaints. The oriental medicine doctor in charge should take into consideration the urgency of the situation and avoid excessively in-depth examinations.

※ Medical Examination Report (Example) (**Appendix 3.**)

- The medical examination report may be recorded in a Google excel form to be used as an online chart if necessary.

3) Assessment of Disaster related trauma

(1) Screening tests

The effectives of early interventions during the acute phase depend on the quick and accurate screening of the patients. Hence, the responses of the survivors should be evaluated first to start interventions and continuous observations of the responses are required to decide what level of interventions is appropriate[16].

If the early responses of the survivors are not accurately assessed, those who really require help and those who do not cannot be differentiated. Moreover, focusing on only the emotional responses can lead to mistaking the emotional stability as alleviations of symptoms and can cause premature termination of treatments[17].

This manual utilizes the simplified mental health survey, proposed by the national center of disaster trauma, as an assessment tool in an effort to establish a cooperative treatment system with the national disaster psychological support practices[18]. This survey is composed of total 40 questions that measure PTSD symptoms (5 questions)[19], Depression (9 questions)[2021], Anxiety (7 questions)[2223], Physiological symptoms (15 questions)[2425], Suicide (4 questions)[26]. Before conducting psychological examinations, explain to the patients about the collection and use of personal data, and obtain a consent form. The self-diagnosis service provided on the national center for disaster trauma website and the application (Maeum program) can be used[27]. This survey can found in Appendix (Appendix 4~8.)

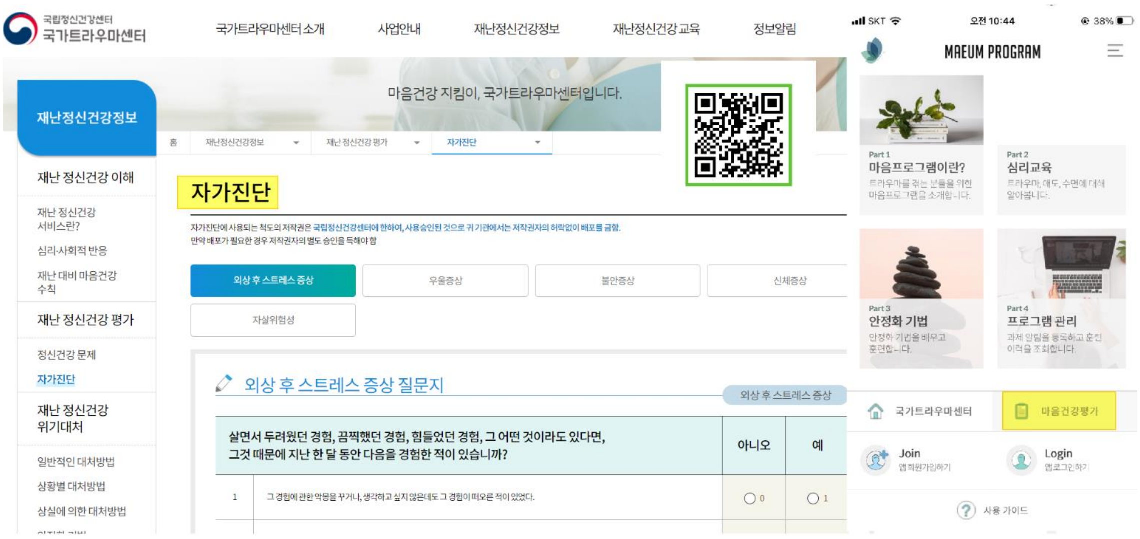

Figure 5. Application (Maeum program) provided by the national center for disaster trauma and the self-assessment service on the website.

① Cautions

﹣ For each scale, read the guide carefully and select only one that corresponds the most to your symptoms.

﹣ For every scale select based on the symptoms experienced since the ‘test period’ to the period written on the guide. (Example) For PTSD (the Primary Care PTSD Screen for DSM-5, PC-PTSD) survey, check the symptoms experienced for the past 1 month from the ‘time of test taking ‘and not at the time of disaster.

﹣ After completing the test, review again if there are any questions missing or the reverse scoring question (P4-4) has been answered properly.

② Scoring Method

﹣ For Depression (The Patient Health Questionnaire-9, PHQ-9), Anxiety (General ized Anxiety Disorder 7-item scale, GAD-7), Physiological symptoms(The Patient Health Questionnaire-15, PHQ-15), PTSD symptoms(PC-PTSD), sum up the score s written by the survivors.

- For Suicide(P4) scale, score as “low risk of suicide” if the answer to question 1 or question 2 is “yes”, and score as “high risk of suicide” if the answer to question 3 is “somewhat true” or “very true” and the answer to question 4 is “no”.

표6. Disaster related trauma assessment survey (Screening test)

Criteria	Normal	Mild-Moderate	Severe

Post-trauma stress disorder(PC-PTSD)	0~1 points	2 points	≥3 points
Depression(PHQ-9)	1~4 points	5~9 points	≥10 points
Anxiety(GAD-7)	1~4 points	5~14 points	≥15 points
Physiological symptoms(PHQ-15)	0~4 points	5~14 points	≥15 points
Suicide(P4)	No history and risk of suicide attempts	History of suicide attempts or risk of suicide attempts in the future	Risk of suicide attempts and no protective factors.

③ Interpretation

Understand the seriousness of the screening survey and select "yes" when there are relevant factors in the criteria (1.Mental health history, 2.Past trauma experience, 3.Persistent stress event, 4.Vulnerable support system) and "no" when there are no relevant factors. Select "Not sure" when there are no relevant factors confirmed.

Table 7. Criteria for high risk and caution group

Classification	Criteria
High risk group	Regardless of the other criteria, the suicide criteria(P4) is 'severe'<br>3 or more criteria are 'severe'
Caution group	1 or more criteria are 'mild' or above<br>2 or more risk factors
Normal group	All criteria are normal

④ Comprehensive examination

For a more comprehensive assessment, following psychological assessment tools for each symptom can be used[28].

Table 8. Comprehensive assessment tools by symptoms

Symptom	Assessment tool

Post-trauma stress disorder	Korean version of PTSD-5 (PCL-5-K)
Depression	Beck Depression Inventory (BDI)
Anxiety	State-Trait Anxiety Inventory (STAI
Anger	State-Trait Anger Expression Inventory (STAXI)
Insomnia	Insomnia Severity Index (ISI)
	Pittsburgh Sleep Quality Index PSQI)
Addiction	Alcohol Use Disorders Identification Test-Alcohol Consumption (AUDIT-C)
	Fagerstrom Test for Nicotine Dependence (FTND)
	Young Internet Addiction Scale (YIAS)
Grief	Inventory of Complicated Grief (ICG)

4) Classification of the chief complaint

Refer to the following classifications to discover the chief complaint of the disaster survivors. Then confirm the severity of the pain by using Subjective Units of Distress Scale (SUDs). The survivors can assess themselves using a score between 0 and 10.

Table 9. Classification of the chief complaint

Classification		
Psychological symptoms	Hypertension/Anxiety	Experience hypertension in daily lives after the disaster.<br>The hypertension in the acute phase can become chronic.<br>Too much complex thoughts and become restless.
	Fear/Emotional paralysis	Express serious anxiety and fear overwhelmed by the shock of the trauma. Emotionally paralyzed and daunted. Inadequate ability to response to the trauma. Potential dissociative symptoms may occur.
	Depression/Grief	Feeling down all the time, and nothing feels joyful. Feel helpless and suffer from guilt.
	Anger/Hypersensitivity	Become hypersensitive and aggressive. Risk of violence toward others.

Physiological symptoms	Insomnia/Nightmares	Difficulty falling asleep and staying asleep with repeated nightmares. Associated with hypersensitivity.
	Loss of appetite/Dyspepsia	Loss of appetite. Difficulty digesting and feel discomfort in the chest. Associated with depression.
	Fatigue/Burnout	Need to stay in bed all day due to fatigue and unable to carry out any activities. Chronic phase. Association with depression.
	Migraine/Dizziness	Headache and dizziness. Consider association with anger and depression.
	Pain	Due to the hypersensitivity and discomfort of living in the shelter, various muscular pain, muscle conclusions etc. may occur. Previously existing pain might worsen.

Table 10. The Subjective Units of Disturbance scale (SUDs)

0 point	No psychological pain
1~2 point	Minimal psychological pain, but do not affect daily lives.
3~6 point	Psychological pain interferes with concentration and daily activities.
7~8 point	Psychological pain is significant and limit daily activities
9~10 point	Highest level of psychological pain that can be expressed

Below is the organized process of medical examinations and treatments by the oriental doctors at disaster site.

Table 11. Process of history taking and basic medical examinations

① Demographic information	Name, Sex, Date of Birth, Contact, Residence / Marriage status, Occupation, Admission route, History of receiving psychological support
▼	
② Basic medical examination	Type of disaster, Phase, Classification based on the level of disaster damage, Vital signs
▼	
③ Screening Test	Simplified Mental Health Survey: PTSD symptoms(PC-PTSD), Depression(PHQ-9), Anxiety (GAD-7), Physiological symptoms (PHQ-15), Suicide(P4)
▼	

④ Comprehensive examination	Incidence date, Chief complaint, Present illness, Medical history, family history, Medication, Smoking and Drinking history etc.
▼	
⑤ Classification and evaluation of chief complaint	Clear psychological symptoms (Hypertonic/Fear/Depression/Anger)<br>Clear physiological symptoms (Insomnia/Loss of appetite/Fatigue/Migraine Dizziness/pain)
▼	
⑥ Oriental medicine examination	Systemic examination (pattern identification) of appetite and digestion, urine and feces, sleep, thirst and diarrhea, Chill and sob, and chief complaint related symptoms / pulse diagnosis, tongue diagnosis, abdominal diagnosis, zygoma diagnosis
▼	
⑦ Examination of risk factor	Mental illness history, past experience of trauma, persisting stress event, vulnerable support system.
▼	
⑧ Explaining assessment result and treatment.	Explain the result of examination and assessment<br>Refer the high risk group to psychological support center or mental health clinic.<br>Perform treatments considering protocol by phases (acute phase/sub-acute phase/chronic phase), protocol by symptoms((psychological symptom(hypersensitivity/fear/depression/anger), physiological symptom(insomnia/loss of appetite/fatigue/migraine and dizziness /pain))

3. Treatment Protocol by phases

It is important to consider the disaster phases and the conditions of the survivors in each phase to take responses. Response objective and method for each emergency phase, acute phase, sub-acute phase and chronic phase based on the time passed since the disaster incidence.

Table 12. Responses by the phases after disaster

Phase	Conditions	Response Objective	Response method
Emergency phase (1~3 days after the disaster)	•Psychological emergency<br>•Shock, Anxiety, fear, panic	•Understand the disaster site and prepare for treatment	•Obtain information about the disaster site<br>•Organize a medical support team, install an on-site clinic and prepare medical supplies<br>•Request for promotion about oriental medicine treatments to the relevant institutions.
Acute phase (3days ~1 month after disaster)	•Hypersensitive or, if serious, in state of panic<br>•Acute stress response<br>•panic, anxiety, Anger, grief<br>•Emotional paralysis, poor judgment, Become stunned<br>•Disturbed daily rhythm	•Hypersensitivity/Stabilization of state of shock<br>•Screen for the high risk group and refer to specialized institution	•Screening test: Find out the severity of the survivors and screen the high risk group. Refer to the professional institution if necessary<br>•Perform psychological emergency practices if required<br>•Practice acupuncture(Ear acupuncture and general acupuncture) and herbal medicine<br>•Breathing regulation: In parallel with the acupuncture treatment<br>•Psychology education on post-disaster responses<br>• Emotional Freedom Technique (EFT)<br>•Training on self-care methods<br>•Treatment frequency: 3 times a week
Sub-acute	•Sub-acute phase<br>•Symptoms	•Alleviate the psychologica	•Screening test: When PTSD, serious depression, dissociation, suicide are suspected, refer to professional

phase (1~3 months)	and pain become clear and deteriorate<br>•Risk of PTSD, Depression<br>•Anxiety and anger in the vulnerable group worsen<br>•Physiological symptoms occur<br>•Disturbed daily rhythm	l and physiological pain<br>•Screen the high risk group and connect to a specialized institution<br>•Evaluation of symptoms from oriental medicine point of view<br>•Prevent PTSD, and depression	medical institution.<br>•Continue the acupuncture(ear acupuncture or general acupuncture) and herbal medicine treatment<br>•Stabilization technique: breathing method, grounding, I-Jeong-Byeon-Gi therapy, Hang-a-ri therapy<br>•Emotional Freedom Technique(Individual or Team)<br>•Education on self-care methods<br>•Treatment frequency: 2~3 times a week

Chronic (After 3 months)	•Continued complications of psychological/ physiological symptoms<br>•Incidences of PTSD and depression in the high risk group<br>•Dependency issue such as addiction occur<br>•Reaches State of Mental-body burnout due to continued extreme confusions	•Active treatments and management of complex emotional and physiological symptoms<br>•Screen high risk group and refer to specialized centers<br>•Support Recovery of the daily lives before the disaster	•Perform active herbal medicine and acupuncture treatment to alleviate persistent physiological and psychological symptoms<br>•Stabilization technique: Consider I-Jeong-Byeon-Gi therapy, Hang-a-ri therapy, resource mindfulness etc.<br>•Emotional freedom technique: Personalized treatment and 4 or 8 weeks of group training program.<br>•Education on self-care methods<br>•Treatment frequency: 1~2times a week(Continued treatment and management for at least 3 months or longer)<br>•Refer to the nearby oriental medicine clinic or hospital if necessary

1) Acute phase (3days~1month after the disaster)

① Response objective

•Stabilize the state of hypersensitivity due to acute stress

•Alleviate the pain and symptoms of the survivors through ear acupuncture and herbal medicine etc.

•Screen high-risk group and refer to specialized institution

② Response method

•Screening of high risk group: Conduct assessment of the trauma to understand the seriousness of the survivors' conditions. Refer the survivors identified as high risk group and the survivors with other mental health emergency to the specialized centers.

•Acupuncture treatment: First consider ear acupuncture which is convenient to perform. The survivors are in a state of confusion from the shock of the event. Acupuncture, as a physiological treatment, can be used primarily for the survivors who are having difficulties with counseling. Ear acupuncture cause low level of pain with minimal invasion, and can be performed on even those who have fear of acupuncture. Use filiform needle for 20~30 minutes. When not enough treatment space is secured and filiform acupuncture cannot be performed, use intradermal acupuncture. In order to prolong the effect train the patients on the acupressure method during the intradermal acupuncture. While the acupuncture is on-going breathing method (example: abdominal respiration) can be performed together. This can also be a group activity. If general acupuncture is possible, perform acupuncture treatment based on the survivors' symptoms and the relevant acupoints and other protocol for each of the symptoms.

•Breathing method: Give training on simple breathing methods and perform them together. This can be conducted in parallel with the acupuncture.

•Herbal medicine: Prescription should be generally focused on the hypertensive state, but can also be prescribed according to the symptoms (granule/syrup).

•Psychological education: Conduct psychological education about trauma responses after disasters (refer to the "psychological therapy" section of the stabilization technique). If possible perform group training for efficiency.

•Psychological emergency practices: First conduct psychological emergency practices in necessary.

•Emotional Freedom Training (EFT): Consider EFT practices on the current psychological pains expressed by the survivors. During the acute phase, the cognitive abilities of the survivors are decreased and when they encounter EFT for the first time, they may find it hard to accept the acceptance speeches such as "Even though I am ____, but I accept myself anyway". In case it is hard to explain the concept of acceptance speech and the survivors find it hard to understand, "Selective acceptance speech" containing the most required expression such as "safety" should be applied.(Example; Even though I am very nervous and afraid, I choose to feel safe. I choose to accept my mind that I am now fine. I choose to accept and comfort myself, who is nervous and

afraid.)
•Education on self-care method: Train the survivors on the acupressure and EFT methods to help them with self-care of their symptoms. For acupressure, train them to pressure each acupoint of ear acupuncture for 20 times each time, and perform this activity for 5 times a day (morning, noon, afternoon, evening, before going to sleep).
•Treatment frequency: Consider intensive support for at least 3 times or more every week.

2) Sub-acute phase (1~3months)

① Response objective

•This is a phase during which multiple psychological and physiological symptoms and pains become more defined and worsen. Hence intensive treatments are required.
•Active management of symptoms through acupuncture, herbal medicine treatments.
•Assessment and management of physiological symptoms
•Identify patients with PTSD and depression through regular screening tests and refer them to specialized centers
•Cautious observations on any incidences of dissociative symptoms or substance dependence.

② Response method and treatment protocol

•Assessment and screening of high risk population: Conduct Trauma Assessment (Especially PC-PTSD, PHQ-9) to screen for PTSD and depression in high risk group, and refer to the specialized institution if necessary. For follow-up conduct screening test every 2 weeks.
•Acupuncture treatment: Perform acupuncture treatments on the acupoints associated with the chief complaints such as anxiety, fear, depression, anger etc. If there is no appropriate space to conduct acupuncture treatment, ear acupuncture can be applied. If filiform needle cannot be used, use intradermal needle and train the patients on acupressure. Moreover, consider adding ear acupuncture meridians customized to each physiological symptoms expressed by the individual patient
•Breathing method: Once abdominal respiration together with acupuncture treatment become familiar, start mindfulness breathing method. Use form of homework or modify the breathing method to help patients continue on.
•Stabilization technique: Select an appropriate method among various stabilization techniques such as ground method, I-Jeong-Byeon-Gi therapy, Hang-a-ri therapy etc., based on the characteristics of the symptoms expressed and preference by the symptoms. Educate the patients to continuously use the method.
•EFT: Consider EFT based on the psychological pain expressed at the time by the survivors. Generally, selective acceptance speech and acceptance speech in combination, up to repeated tapping processes are used Perform individual treatment for 1-2 times a

week, and if the survivors can organize a group by themselves, perform 1 group treatment per week. Group therapy can cause feelings of empathy and being comforted, and lead to Borrowing Benefit. Educate the patients to utilize this technique as a self-care method as well.

•Education of self-care method: Use acupressure and EFT. IF necessary, add other acupressure points (sin-mun, nae-guan, jok-sam-li, sam-eum-kyo, tae-choong, yong-chun, baek-hwae, poong-ji etc.) to ear acupuncture acupoints. Self-acupressure is to be conducted 3~5 times (morning, noon, afternoon, evening, before going to sleep) a day, and each time every acuppoints are to be pressured for 20 times.

•Treatment frequency: Consider 2~3 times a week support

3) Chronic phase (After 3months)

① Response Objective

•Control complex emotional problems such as anxiety, nervousness, depression, anger etc.

•Active management of physiological symptoms through herbal medicine and acupuncture treatments.

•Support for recovery of daily lives and activities

•Cautions observations on any incidences of PTSD, depression or substance dependence, and referral to specialized centers.

② Response method and treatment protocol

•Follow up examinations and assessment of PTSD, depression, substance dependence (10minutes): During the examination, perform observations and associated assessments (PC-PTSD, PHQ-9, AUDIT) to diagnose mental disorders and refer to specialized centers if necessary.

•Acupuncture: Continue to perform general acupuncture and ear acupuncture. Consider various symptoms and characteristics of the survivors to select target meridians. Continue to perform breathing method in parallel as well.

•Herbal medicine: Consider both the physiological and psychological symptoms to perform prescribe herbal medicines and perform acupuncture treatments.

•Stabilization technique: Conduct appropriate stabilization techniques personalized to the situation of each survivor. For follow up patients, continue to use a technique preferred by the patient among all previously attempted techniques.

•EFT: Consider EFT for the main complaint and psychological pain. Conduct individual therapy once a week in parallel with group therapy 1~2 times a week if possible. The general curriculum containing acceptance speech and selective acceptance speech can be used together. Educate the patients to use the techniques as self-care method.

•Education on self-care: Use acupressure and EFT. If necessary, add other acupressure meridians to the ear acupuncture meridians (sin-mun, nae-guan, jok-sam-li, sam-eum-kyo, tae-choong, yong-chun, baek-hwae, poong-ji etc.). Self-acupressure should be

conducted 3~5 times a day (morning, noon, afternoon, evening, before sleeping), and each time pressure should be applied for 20 times. Instruct the patients to perform acupressure and EFT by themselves when necessary. Additionally, train the patients on ascetic treatments, walking meditation etc.

•Treatment frequency: Continuous support for 1~2 times a week. Continuous feedbacks and management for minimum of 3 months or longer are required.

•If intensive oriental medicine treatment or hospitalization are required, refer to nearby oriental medicine clinics/oriental medicine hospitals/national public hospitals/public health centers.

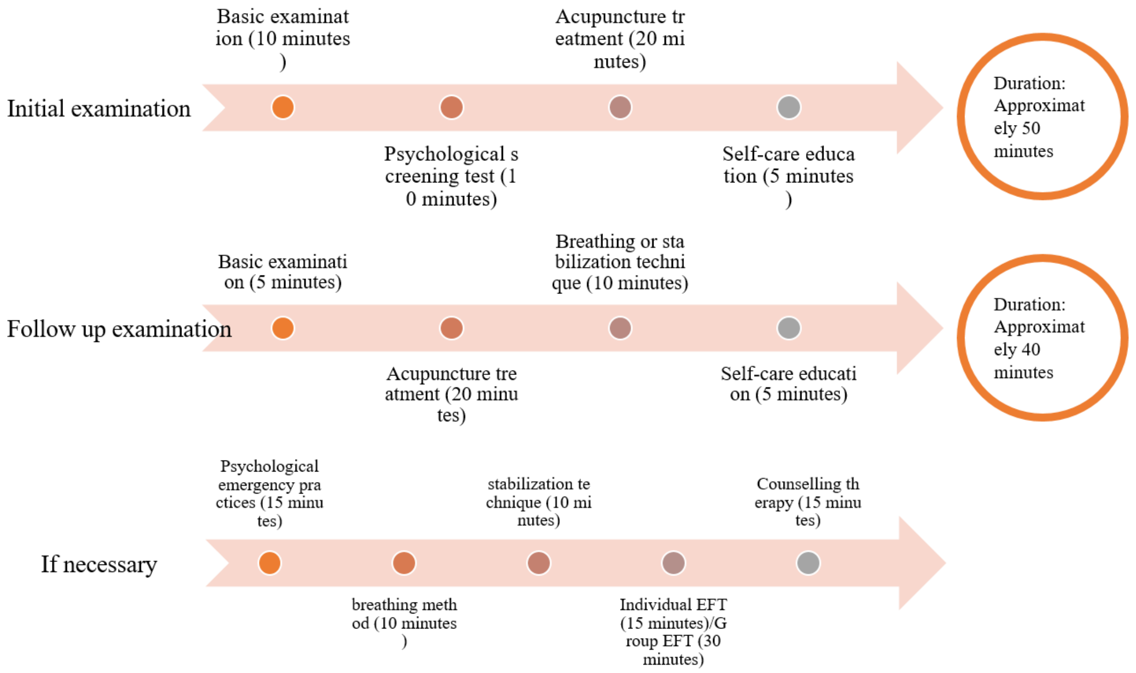

Figure 6. Examination procedure (Initial examination, Follow-up) and estimated time required

4) Assessment

• If the survivors agree, it is recommended to use SUDs or other screening tool to assess the seriousness of the symptoms every 2 weeks during the follow up.

• If the survivors' conditions do not improve or deteriorate during follow up assessments, consider more intensive treatments or refer to mental health clinics.

4. Treatment protocol by symptoms

The symptoms of the survivors were differentiated into psychological symptoms and

physiological symptoms. Response/management methods for each symptoms and intervention protocols (herbal medicine, acupuncture, acupressure points) for each chief complaint were proposed. Treatment protocol by each symptom can assist fast selection of interventions and actions on the disaster site. The detailed contents of the interventions were proposed through discussions and consultations among the development team and specialists, based on the evidences and oriental medicine clinical guideline for each symptom.

• First, secure an appropriate space for examinations and acupuncture treatment.

• During treatment on site, first refer to the protocol by symptoms, but also set treatment objective considering the characteristics of survivors by phases.

• Consider herbal medicine and acupuncture as the basic treatment, and consider performing breathing method, stabilization technique, EFT and counseling in parallel when the survivors' chief complaints are psychological symptoms.

• When complex symptoms (Example: Insomnia together with loss of appetite) appear, consider individual treatment and acupressure points.

• For ear acupuncture, consider the basic acupressure's (shin-mun, kyo-gam, liver, kidney, and lung) of the protocol of ear acupuncture treatment by National Acupuncture De-toxification Association (NADA)[29], and individual acupressure's for each patient's physiological symptoms can be added accordingly (Treatment. Refer to ear acupuncture treatment and acupressures by symptoms).

• Intradermal acupuncture has low risk of adverse event, however, program duration is recommended to be kept within 50 to 60minutess. After adhesion, inform the patient to remove the acupuncture when there are any irritations on the skin (edema, heat, pain etc.)

• When the pain around the intradermal acupressure persist, use vaccariae herba or magnet instead.

• For herbal medicine, granule formation should be primary consideration for convenience of consumption (NHI covered granule and non-covered granule).

• Other treatment: Consider Electro-acupuncture, pharmacopuncture, electronic moxibustion and simple chuna if possible.

• For acupressure, pressure each acupoints 20 times, 3 to 5 times a day.

• Asi-acupoint: The doctor should find acupressure points of the target muscles and train the patients' to perform self-acupressure.

Ex*: NHI covered granule, Ex: granule not covered by NHI, T: Herbal medicine

Table 13. Treatment protocol by symptoms

	Symptoms/ Condition	Possible Treatments	Individual treatment		Self-Care
			**Herbal medicine**	**Acupuncture**	**Acupressure**
Psychological symptoms	Hyperarousal /anxiety	**Acupuncture, Ear acupuncture, Herbal medicine, Breathing regulation, Stabilization technique, EFT**	**Ex***: Sihosogan-san, Gamisoyo-san / Ex: Sanjoin-tang, Sihogayonggolmoryeo-tang / T: Ondam-tang, Xiaotan-Jieyu Prescription (Xiaoyao Powder), Jie Yu Wan (soyo-san+Gammakdaejo-tang), Hwangryeonagyo-tang	Sin-mun, nae-guan, Sam-E un-Gyo, Sim-Su	Sin-mun, nae-guan
	Fear/ Emotional paralysis		Ex: Gyejigayonggolmoryo-tang, Shihogesikungang-tang, Gwibi0-tang, Tianwang Buxin Dan	No-Gung, Shim-Mun, Yong-Chun, stimulate Junghyeol (eunbaek, yaetae, jungchung, etc.)	No-guong, Jung-hyul
	Depression/ Grief		**Ex*:** Sihosogan-tang, gamisoyo-san, Banxia houpo decoction / Ex: gwibi-tang, shihogayonggolmoryo-tang, gyejibokryung-wan, yukgunja-tang	baekhoe, yintang, hapgok, taechung, joksamni	Hab-gok, Baek-hwae

	anger/hypersensitivity		**Ex*:** Hwangryunhaedok-tang / Ex: ukgansan,	taechung, haenggan, sameumgyo, jeongjung	Tae-Choong, San-Eun-Gyo
Physiological symptoms	Insomnia/nightmares	**Acupuncture(Ear acupuncture), Herbal medicine**	Ex: Sanjoin-tang, gwibi-tang, tianwang boxin-dan	baekhoe, sasinchong, anmyeon, sinmun, sameumgyo, yongcheon, sinmaek(sa)johhae(bo)	Shin-mun, Yong-Chun, An-Myun
	Loss of appetite/Dyspepsia		**Ex*:** Hyangsapyeongwi-san, banwhasashim-tang, bulhwangeumjeonggi-san, samchulkunbi-tang, Banxia houpo decoction / Ex: daehwajungeum, naesowhajung-tang, yukgunja-tang, sini decoction	jungwan, cheongchu, hapgok, naegwan, joksamni, taechung	Joong-wan, Jok-Sami-Li, Chun-Chu
	Fatigue/Burnout		**Ex*:** Bojungikgi-tang, palmutang, gamisoyo-san / Ex: gwibi-tang, six tastes/palmijihwang-hwan/ T: ssanghwa-tang, shipjeondaebo-tang	taebaek, joksamni, wanwon, yongcheon, eunbaek	Jok-Sam-li, Eun-baek, Yong-Chun

	Headache/Dizziness		Headache- **Ex*:** galgeun-tang, chungsanggyuntong-tang, banhabakchulchunma-tang / Ex: oryeong-san // Dizziness- **Ex*:** banhabakchulchunma-tang / Ex: oryeong, dangui-jaya-san / T: tianma gouteng decoction, youngkaechulgam-tang, jaeumkunbi-tang	Headache: baekhoe, pungji, hapgok, jokimeup<br>Dizziness: taechung, hapgok, hyeonjong, baekhoe, pungji, taeyang, yintang, sangseong, solgok, gyeonjeong	Tae-Yang, Poong-Ji, Hab-gok, Jok-Gyu-Eum
	Pain		**Ex*:** ojeok-san, kumikanghal-tang, chungsangyuntong-tang / Ex: jakyakgamcho-tang, ganggwuisu-san / T: ssanghwa-tang	Acupressure by areas of pain, Sa-am acupunture Eo-hyeol bang	Asi-acupunture

5. Psychological First Aid Treatment

1) Introduction

There are various programs developed and distributed worldwide for psychological stabilization and recovery of disaster survivors. Psychological support for individuals who were exposed to disasters can be largely categorized into acute phase intervention within 1 month immediately after disaster, sub-acute phase intervention within 1 to 3 months of disaster, and chronic phase intervention when the psychological problem persists for more than three months. Among them, psychological first aid (PFA) is representative of a program that provides mental health support for the acute phase intervention and is “the act of giving humanitarian help for those who are in pain and in need of help.” It refers to a psychological social service, not the conventional professional psychological intervention that provides realistic and practical help needed in emergency situations[30].

The purpose of psychological first aid is to provide practical help in order to reduce the initial shock of disaster survivors from the trauma incident and strengthen their adaptation and coping capabilities during the acute phase, referring to the period of time immediately after and few days after disaster occurrence[31]. Just as general first aid relieves physical pain of the injured and help stabilize their physical functions to normal, psychological first aid helps to strive for the survivor’s stability and help them to better cope with the disaster psychologically. Through psychological first aid, the aftereffects of disasters are minimized and the survivor’s normal recovery is facilitated. The target of psychological first aid covers all age and gender including children and adolescents, and is applicable regardless of cultural differences and disaster types[32]. Psychological first aid consists of simple, basic principles and anyone with sufficient education can perform it[33].

In the United States, the National Center for PTSD and National Child Traumatic Stress Network jointly developed a field guide. The original edition of this PFA field guide was first published shortly after Hurricane Katrina in the United States and the second edition was published in 2006 with additional information and guidance. Utilizing an evidence-based approach, it is designed to help children, adolescents, adults and their family following traumatic events such as terrorism or natural disasters. It can be used by workers in various mental health field related to disasters, and it is easily usable with its specific step by step examples and methods, categorized by age groups. Currently, there are five types of PFA guidelines worldwide including the guideline issued by the World Health Organization in 2011. This manual has been written with reference to the PFA guidelines issued by the United States and the World Health Organization

2) Basic Purpose of Psychological First Aid Treatment[34]

① Create an empathetic, humane relationship without judging the situation or the psychological state.
② Provide physical and emotional support to help the targets feel safe as fast as possible.
③ Soothe the disaster survivor who are overwhelmed and confused.
④ accurately identify the needs and psychological difficulties of disaster survivors.
⑤ Help connect the disaster survivor with social support such as their family and friends.
⑥ Facilitate positive coping methods and encourage active coping.
⑦ Provide psychological education so they may cope with the psychological impact of disasters.
⑧ Continue to communicate with other support systems and make sure the handover is done properly when leaving the site.

3) Targets for Psychological First Aid Treatment

Psychological first aid treatment is for those who are in distress immediately after a serious crisis situation and can be applied to both children and adults. There are often cases that cannot be resolved with psychological first aid treatment alone and such cases require a much more advanced and professional support. In these cases, one should seek help from other people such as experts, colleagues, or local residents. It is intended to reduce the initial pain immediately after terror attacks or disasters and help with short-term adaptation and long-term recovery of function and should be applied under the assumption that not all survivors will experience severe psychological problems or have long-term difficulties in recovery.

4) Main Principles of Psychological First Aid

The five main principles of PFA is sense of security, calm, connectivity, effectiveness (one’s belief and ability in coping) and optimism[35]. The application of such principles can be integrated with other treatments. PFA is associated with all areas that satisfy basic needs such as physical security, food, protection, connectivity and survival. It includes all areas of identifying the survivor’s needs, monitoring relief and recovery environment, home visits, delivering information, giving technical assistance and counseling, building resilience, assessing severity and implementing early treatments[36].

5) Do’s and Don’ts for Good Communication[37]

Communication methods are very important when providing emergency medical

assistance. Disaster survivors can be very angry, vexed and sometimes even feel guilty and tormented. It is very important to listen to those who want to talk about the painful experience they have been through. However, one must not be forceful in getting them to talk. Be scarce with your words and allow silence. A certain amount of silence allows the disaster survivors to think for themselves.

Table14. The Do's and Don'ts's when providing emergency medical assistance.

Do's	Don'ts
• Find a quiet place to talk.<br>• Respect the privacy and confidentiality of the disaster survivors.<br>• Show that you are listening by nodding or responding to their story.<br>• Be patient and talk calmly.<br>• Be clear with what you know and what you don't know (E.g. "That's something I'm not familiar with. I'll look into it.")<br>• Provide information and make it concise so that the person receiving help can understand it.<br>• Listen and be empathetic to the disaster survivors' shock and sadness. (E.g. "I'm so sorry, that must be so heartbreaking.")<br>• Identify, acknowledge and encourage disaster survivors' strengths and areas that they are coping well by themselves.<br>• Accept silence and wait.	• Don't force them to talk.<br>• Do not interrupt them mid-way, or urge during conversations. (E.g. looking at the clock, speaking quickly, etc.)<br>• Avoid physical contact.<br>• Don't judge what the other did or did not do or their emotions. (E.g. "You shouldn't feel that way.", "Consider being alive lucky.")<br>• Don't talk about things you don't know.<br>• Don't use jargons.<br>• Don't talk about others.<br>• Don't make promises or false convictions that cannot be kept.<br>• Do not think or act as if you can solve all the problems faced by the disaster survivors.<br>• Do not deprive the disaster survivors of their strength and their ability and opportunity to cope by themselves.<br>• Do not describe the disaster survivors in a negative way (E.g. "Crazy people", "insane people", etc.)

6) Key Activities of Psychological First Aid Treatment[38]

The specific content of the PFA differs slightly between guidelines. In the PFA guidelines of the World Health Organization (WHO), it was divided into ①preparation stage, ②observing, listening and connecting stage, and ③final stage. The PFA guidelines of the U.S. National Center for PTSD is as follows: ① First contact and

relationship formation, ② safety and support, ③ stabilization, ④ collecting information : identifying current needs and suffering, ⑤ practical help, ⑥ connecting with social support systems, ⑦ providing coping techniques, ⑧ guidance to affiliated organizations. In domestic manuals, either the PFA of WHO or the U.S. National Center for PTSD is selected and referred to. Since the National Trauma Center is also based on the U.S. PFA, this manual will briefly describe the U.S. PFA guidelines[39].

① First contact and relationship formation

Introduce yourself such as your name, affiliation, role, and purpose of your visit to let them know that you are on an official business. Ask for consent in advance of a conversation, and check if there are any problems that need to be resolved immediately or medical problems that require immediate treatments. Confidentiality should be guaranteed as much as possible, and legal reports of self-harm, physical harm, abuse, abandonment should be reported to the institution.

② Safety and support

Ensure physical safety and provide information on disaster response systems and activities. Pay attention to the physical well-being and facilitate social interaction. Support disaster survivors with lost family members, such as identifying children who have been separated from their guardians and give special attention to survivors who have experienced bereavement. Protect disaster survivors from situations where they have to think about their trauma. Focus on spiritual issues and help survivors who need to identify bodies.

③ Stabilization

Induce calm and psychological stability in disaster survivors. Observe the reactions that occur in disaster survivors based on your understanding of post-disaster reactions. Induce relaxation by teaching deep breathing techniques. And use grounding techniques (refer to the treatment section in “Stabilization Techniques”) to help them focus on the here and now.

④ Collecting Information

Clarify the specific needs and psychological difficulties of survivors in order to tailor support to each individual with the aim of identifying the current needs and suffering. Gather information on issues that require immediate referral, professional assistance, or additional consultation.

⑤ Practical Help

Identify the most pressing needs and articulate them. Develop an action plan and act to solve the problem.

⑥ connecting with social support systems
Connect the survivors with social support systems such as family, friends and community networks and utilize available support systems such as other survivors and support groups. Introduce small groups to build relationships.

⑦ providing coping techniques
Provide information about stress response. Teach relaxation techniques and anger control methods, and introduce positive coping methods for adaptation and dealing with negative emotions.

⑧ Guidance to affiliated organizations
In cases where there are urgent medical, psychiatric or mental health problems, a threat or concern for self-harm or harming others, problem with alcohol or drug use and any problems that persists for more than 4 weeks or the survivor requests it a referral, inform them of other affiliated organizations.

6. Other Considerations

(1) Post-care

• Post-care is important in disaster sites. Because you do not know when they will receive medical services again, it is necessary to explain the characteristics of each stage's symptoms, follow-up treatment methods, self-management education, number of in-patient visits, and connection with other medical institutions.

• Improve self-efficacy by acupressure, give homework to persistently perform stabilization techniques, introduce internet resources (National Trauma Center website[40], etc.) and mobile apps (National Trauma Center App 'Mind Program') that will help with self-management and encourage usage.

• Outreach (Treatment visits): Some residents who are unable to visit an evacuation center or have failed to evacuate do not receive adequate psychological support. In addition, disaster survivors who are in chronic phase leave shelters and return to their homes, but it is difficult for the elderly, the disabled, and other vulnerable groups to regularly visit mental health centers or public health centers to receive psychological support. Hence, an outreach or treatments visits are necessary. A specialized team management is required rather than a simple home visit, and oriental medicine treatment can be provided through connection with the local public health oriental medical doctor and the local oriental medical association.

(2) Precautions

• Keep in mind that talking about a disaster experience can be another traumatic process for a disaster survivor as it is a very painful process.

• Disaster type: Depending on the disaster type, there are some differences in the support methods. During treatment, take into consideration that the responses of survivors may differ depending on the type of disasters such as natural disaster, social disaster, and infections disasters.

• Consideration should be given to the risk of suicide of disaster survivors.

• Consideration of survivors' characteristics: Interventions should be used in consideration of the survivors' gender and age during treatment.

(3) Prevention of proxy trauma/burnout of medical staff

• You should empathize and share the suffering of the survivors but be careful not over-immerse yourself as to not exhaust the medical staff. Moreover, be cautious of the disaster survivors becoming overly dependent on the medical staff.

• Strategies for prevention of proxy trauma and burnout of oriental medicine doctors who are on-site should be included as part of the treatment system.

• Examine the following proxy trauma and burnout conditions.
  - I feel the difficult emotions that the survivor felt after treatment.
  - I also remember the horrible shock and memories of the survivors.
  - It is difficult to focus on the survivor's story.
  - I think that I am of no help to the survivor and feel incompetent.

• Self-management of medical staffs
  - Take sufficient rest before and after treatment and do physical activities.
  - Do not skip regular meals.
  - Do not see patients continuously for a long period of time. Secure appropriate days off.
  - Regularly (daily/every other day/every week) talk about your feelings with other oriental doctors at the treatment site.

(4) Ethical guidelines at disaster sites

• Put the rights and interests of disaster survivors as priority and refrain from doing the survivors any harm.

• The details of the medical treatment and counseling with survivors shall not be disclosed to third parties or the outside world without consent. Be sure to provide only accurate information, and admit what you don't know.

• If you need help that goes beyond your expertise, seek advice or refer to the

appropriate experts.

III. Treatment Methods

1. Ear Acupuncture
2. Acupuncture
3. Herbal Medicine
4. Emotional Freedom Technique (EFT)
5. Stabilization Techniques
6. Self-care methods
   (1) Dealing with each symptoms
   (2) Acupressure
   (3) Doyen Exercise
   (4) Walking meditation

1. Ear Acupuncture

1) Introduction

Today's international clinical practice guidelines do not recommend drug treatment for disaster trauma; hence psychological intervention is mainly used for disaster psychological support. However, when survivors and victims of disasters occur on a large scale, human and material medical resources will be consumed greatly, making it difficult to provide immediate, efficient and continuous support. Moreover, long-term psychological support can cause burnout for psychological support providers[41]. Hence, the use of effective and safe non-drug and non-psychological interventions that can be used at disaster sites can be helpful.

Ear acupuncture is a representative of non-pharmaceutical treatment of oriental medicine, and it is a safe and effective method that can be operated simply and its efficacy and safety have been reported not only in the field of pain and emergency medicine, but also in mental disorders. Ear acupuncture treatment is easy and fast, can improve both physical and mental symptoms and is inexpensive, hence is suitable for use in disaster treatment sites. Overseas, the NADA protocol is already being used in various disaster sites around the world[42].

Table14. Advantages of using ear acupuncture at disaster sites

Difficulties during disaster relief	Specific Problems	Advantages of Ear Acupuncture
**Cultural Competency**	• Language barrier<br>• Reluctance to provide assistance to unfamiliar outsiders<br>• Counseling treatment may prove to be difficult to apply due to cultural differences in the community and language barriers.	• Non-verbal treatment, hence no language barrier<br>• Disaster relief can be provided by training local residents in acupressure methods.
**Sustainability**	• Reliance on external aid.<br>• High cost of continuous consultation by experts	• Inexpensive<br>• It is possible to train local residents to apply it safely and effectively<br>• Ability to promote community resilience
**Application of the western mental health model**	• The DSM diagnostic approach is not appropriate<br>• Medication and	• No need for diagnosis<br>• Non-pharmaceutical treatment<br>• It is possible to supplement or

	conversation therapy are not appropriate at the disaster site.	add to existing treatment.

2) Evidence and Cases

Corrêa et al., a Brazilian research team, analyzed and reported the effects of ear acupuncture on stress, anxiety, and depression in adults and the elderly through a systematic literature review[43]. Through a comprehensive search in a total of 11 electronic medical databases, 24 related clinical studies were collected and 92% of the included studies (22 articles) reported that ear acupuncture had a positive effect on stress, anxiety and depression.

As a result of analyzing 8 randomized controlled trials reporting the effects of ear acupuncture treatment for primary insomnia, ear acupuncture treatment significantly improved the quality of sleep in insomnia patients, shortened the sleep latency, and reduced the number of nighttime awakenings compared to false ear acupuncture treatment or no treatment group[44].

The research team developing this manual conducted a systematic literature review to evaluate the effect of ear acupuncture treatment on psychological trauma-related disorders occurring in survivors of large-scale disasters[45]. According to the analysis of a total of 10 clinical studies, it was reported that ear acupuncture improved not only the overall PTSD symptoms in PTSD patients, but also improved symptoms of depression and anxiety. Regardless of the east or the west, the acupuncture points used on disaster survivors for ear acupuncture were similar. In order to systematically establish the basis for ear acupuncture treatment in the future, a standardized treatment strategy such as the NADA protocol can be used.

There has also been a case where a community of a large-scale disaster survivors and refugees relieved ear acupuncture education and implemented the treatment extensively. Acupuncturist Yarberry (2010) provided NADA protocol treatment to refugees who were suffering from PTSD due to the bloodshed in Kenya in 2007[46]. 21 refugees were trained so that those residents could directly provide the NADA protocol treatment to their neighbours and 18,000 treatments were provided over the course of 6 months. At that time, children were suffering from symptoms of severe stress, anxiety, nightmares and nocturnal enuresis. sinmun acupuncture points and kidney acupuncture points was used and after treatment, cases of nocturnal enuresis and children fighting decreased. Yarberry (2011) carried out the NADA protocol treatment and education for PTSD victims of the 2010 Haiti earthquake that caused 500,000 casualties and 1.8 million refugees[47]. 24 residents were trained with the NADA protocol treatment and over 2,000 residents were treated. After treatments, refugees reported feeling generally better and having a sense of hope, and their chest palpitations, headaches and stress were relieved.

After the 9/11 terror attacks, many hospitals in New York City provided treatment to

trauma survivors at existing addiction treatment centers that implemented NADA protocols[48]. St. Vincent’s Hospital, which was closest to ground zero where the explosion happened, opened a stress clinic that utilized NADA protocols and provided over 40,000 treatments to local residents. Other acupuncture teams were active for 24 hours immediately after the attack and provided treatments to the police and firefighters responsible for handling the disaster. Community Rebuilding and Relief through Education and Wellness (CRREW), a team providing acupuncture and massage therapy, were formed following the attacks of 9/11 and provided acupuncture treatments to the fire departments and rescue teams of New York City. In 2006, the New York City R egional Emergency Medical Response Council brought the CRREW team with cri sis relief experience to a major accident response team.

Acupuncturists Without Borders (AWB) was found in 2005 immediately after Hurricane Katrina in the United States by 75 acupuncturists and uses the NADA protocol to provide medical assistance at disaster sites in the U.S. and around the world[49]. During Hurricane Katrina, AWB provided ear acupuncture as medical support to more than 8000 people in collaboration with the disaster psychological support team, free clinics, shelters, firefighters, police and military personnel. Since then, they have provided treatments for hundreds of different disasters including earthquakes, floods, shootings, wildfires, and more recently, Covid-19.

A study in which the NADA protocol was applied to disaster trauma was also published in Korea. A treatment team at Pohang Oriental Medicine Hospital affiliated with Daegu Haany University administered ear acupuncture treatment (intradermal acupuncture) twice a week for 8 weeks to long-term evacuees following the 2017 Pohang earthquake and was reported to improve psychological trauma and depressive symptoms[50]. Moreover, the improved status was maintained even in the follow-up evaluation, four weeks post treatment. Secondary outcome indicators such as quality of sleep, anger, and quality of life also showed significant improvement after 8 weeks of treatment. No adverse reactions were found during the treatment period.

3) Treatment Mechanism

Ear acupuncture is thought to show therapeutic effect through the stimulation of the autonomic nervous system. When the external auditory meatus is stimulated, a cough reflex by the vagus nerve appears (Arnold’s Reflex), and this reflex is caused by the auricular branches of the vagus nerve (ABVN) which is an afferent nerve. And because the stimulation of ABVN can induce a response similar to that of the vagus nerve, it is related to ear acupuncture treatment[51]. ABVN delivers fibers to the nucleus of the solitary tract (NTS) and the stimulation of ABVN is thought to be able to regulate the autonomic nervous system based on the complex connection of the NTS between the brain and the intestines. Ear acupuncture increases the activities of the vagus nerves

and is thought to be able to regulate the cardiovascular system (decreasing heart rate and blood pressure, increasing heart rate variability, etc.), the respiratory system (improving olfactory perception, increasing lung capacity, etc.), the gastrointestinal system (regulating gastric motility, reducing abdominal distension, etc.) and the endocrine system (contributing to homeostasis by regulating endocrine hormones)[52]. In addition, the ear has a spot where it is controlled by the auriculotemporal nerve, a branch of the mandibular nerve which is the third branch of the trigeminal nerve, and it is thought that this pathway is able to stimulate the vagus nerve or activate certain neural structures such as the brainstem and forebrain[53].

The mechanism of ear acupuncture can also be seen within the category of acupuncture treatment. The result of comprehensively collecting previously published animal experiments and summarizing the effect and mechanisms of acupuncture treatment for PTSD are as follows[54].

(1) Acupuncture treatment reduced anxiety symptoms and fear responses and weakened fear-conditioning in PTSD animal models. As for related mechanism, increased activities of hypothalamic-pituitary-adrenal axis and increased plasticity of specific areas of the brain such as the amygdala and hippocampus have been reported.
(2) Acupuncture treatment was able to improve depressive symptoms in PTSD animal models. As for related mechanism, an increase in plasticity through the mammalian target of rapamycin, which is related to various biological functions such as aging, brain function, protein synthesis and cell growth, has been reported in the hippocampus.
(3) Acupuncture can affect sleep structure in PTSD animal models. As for related mechanism, regulation of cytokine levels in the hypothalamus has been reported.
(4) Acupuncture treatment was able to alleviate the impairment of spatial learning and memory in PTSD animal models. As for related mechanism, regulation of abnormal brain waves and cortical blood oxygen concentration has been reported.
(5) Acupuncture treatment was able to reduce excessive stress reactions in PTSD animal models. As for related mechanism, regulation of EEG activities in the hippocampus and reduction of neuronal NOS expression in the locus coeruleus have been reported.

4) Ear Acupuncture Protocol

Based on the NADA (National Acupuncture Detoxification Association) protocol, it was originally developed for the purpose of substance abuse treatment, but after it was found out to be helpful for those who have experienced severe trauma following New York’s 9/11 terror attacks and Hurricane Katrina, it was not only used for addiction but also other variety of mental health problems.

The clinical evidence of the NADA protocol for mental disorders has been

accumulated over the past 10 years on various mental disorders, with the largest number of studies being related to addiction. Besides addiction, there has been variety of subjects such as insomnia, borderline personality disorder, and burn patients complaining of anxiety, medical staff in the intensive care unit, anxiety disorder, major depression disorder, neurological loss of appetite, mothers complaining of anxiety after cesarean section, earthquake victims and more. Ear acupuncture treatment can be used along or in combination with other treatments, and depending on the study, general acupuncture treatment or acupressure can be performed in addition to the NADA protocol[55].

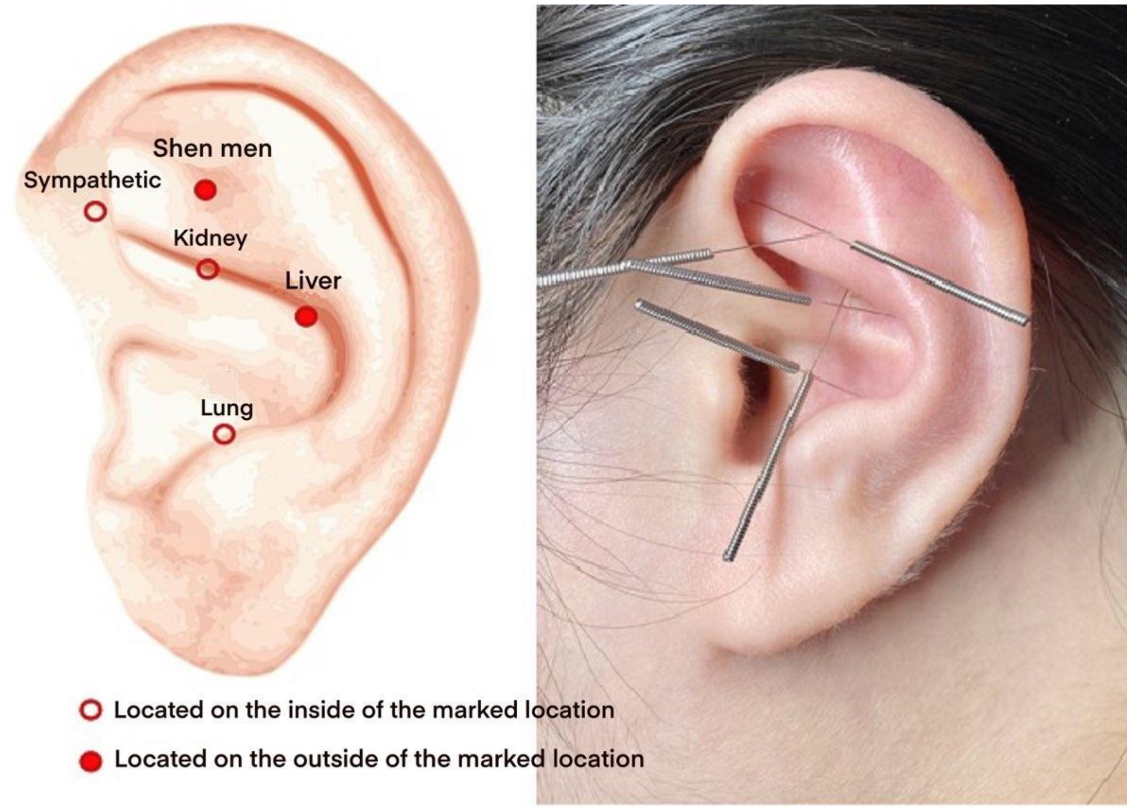

Figure7. NADA Protocol: sympathetic, shen men, kidney, liver, lungs

• **Understanding of acupuncture point in accordance to the NADA protocol**

The 'kidney' is associated to fear in oriental medicine and is the source of brain function as it stores the brain. The 'liver' is in charge of the communication with gi and when the communication becomes stagnant, we become irritable and easily angry. Liver health is important in anger control and keeping the body functioning smoothly. The 'lungs' are responsible for breathing and immunity, and are particularly associated with depression. The 'sinmun' is the pathway of the mind, is associated with the heart and controls anxiety/tension, and exhibits calmness, peace and relaxation effects.

‘Sympathetic’ regulates the balance of sympathetic/parasympathetic nerves and is associated with strong pain killing effect and is related to the spleen/stomach, promoting digestion by vasodilation of the internal organs, and reduces irritation and aggression along with the liver. When teaching acupressure therapy (acupuncture point stimulation therapy), it is thought that the patient will use it more actively if the characteristics of the acupressure points are explained as above.

- Treatment Procedure: The operator washes and dries their hands thoroughly with either soap or alcohol gel prior to the procedure. Afterwards, a disposable alcohol swab is used to disinfect the patient’s localized area of acupuncture points. Then, using clean sterilized tweezers, disposable sterile intradermal needles are attached to the patient’s acupuncture treatment points on one side. Advise the patients to apply pressure to the intradermal needles attached to each acupuncture points about 3~5 times per day (after waking up in the morning, after breakfast, lunch, dinner and before bed) and 20 times each. On the next treatment day, the operator uses tweezers to remove the intradermal needles and checks the local skin for any adverse reaction, and uses disposable alcohol swab to disinfect the local read of the patient’s acupuncture points. Then the same acupuncture treatment is performed using the same method on the acupuncture points on the opposite side.

In general, the application of the NADA protocol is no different from the existing ear acupuncture treatment, with the needle inserted approximately 0.3cm and the immersion period lasting 30~45 minutes. As in the case of this research team, instead of acupuncture needles, Melandrii Herba seeds or beads, and intradermal needles can be applied to the acupuncture points for pressure to be applied.

• Treatment frequency: Acupressure therapy is performed twice a week.

• Individual Acupuncture Points for Each Symptom [56]

When selecting ear acupuncture points, the following acupuncture points (general acupuncture points and individual acupuncture points) can be considered for each symptom of the survivor’s main complaint.

Table15. Individual Acupuncture Points for Each Symptom

Musculoskeletal Pain			
General acupuncture points1	General acupuncture points2	Area	Individual acupuncture points
Liver, kidney, sinmun	Cerebral cortex (cerebellum)	Headache	Cerebral cortex (sub cortex)
		Neck pain	Larynx, cervical vertebra, 경
		Low back pain	Lumbosacral
Internal Disease			

Liver, kidney, sinmun	Sympathetic	Digestive system	Stomach, large intestine
		Respiratory system	Lung
		Urogenital system	Bladder
		Circulatory system	Heart
	Endocrine	Obstetrics and gynecology	Female reproductive system (uterus)
		Five sensory organs	eye, ear, nose, mouth, throat
		Dermatology	Lung
Others			
		No smoking	Sinmun, hormonal secretion (endocrine), lung, throat, nose
		No drinking	larynx, cerebral cortex, secretion, hepatoid

5) Safety Procedures and Precautions

Ear acupuncture treatment is considered to be a very safe treatment. However, some side effects may occur if the safety procedure is not followed. In a systematic literature review published in 2014, as a result of comprehensively collecting and analyzing literature reporting side effects related to ear acupuncture treatment, the most frequently reported adverse reactions were pain when the needle is injected, nausea, local discomfort, minor bleeding and dizziness. In addition, adverse reactions such as local skin irritation and discomfort, mild tenderness and pain, and dizziness have also been frequently reported in acupressure without needle insertion. However, these adverse reactions were mostly temporary, mild and tolerable and no serious side effects were identified[57].

In the case of long-term intradermal needle attachment, more caution regarding infection is needed. According to a literature review published in 2020, there were case of infection, periochonitis and chondroitin due to prolonged attachment of intradermal needles. And the main cause of these side effects were presumed to be the use of contaminated tools. Accordingly, the authors suggests that the use of seeds such as Melandrii Herba seeds has clinical benefits while reducing chances of infection[58]. Therefore, safety procedures should be followed for the use of ear acupuncture treatment at disaster sites[59].

• Safety Procedure for Ear Acupuncture Treatment Protocol

- If the survivor's blood pressure is 140/90 mmHg or higher, additional treatment such as ear tip blood letting may be considered. In case of hypertensive emergency of 180/120 mmHg or higher, it I necessary to request medical treatment or transfer the survivor to an emergency medical center. Also, if there are symptoms of a respiratory infection such as fever or coughing, ear acupuncture treatments should not be used.
- Ear acupuncture treatment should not be used if the survivor's ears are damaged, has unhealed wounds, scars, frostbites or deformities.
- When applying intradermal needles, advise that survivors take 30 minutes of rest and remove the intradermal needles if there is dizziness or nausea even after rest.
- After attaching intradermal needles, if survivor experiences pain, heat or swelling due to the insertion, have them remove the intradermal needles themselves and establish a means of contact in case of discomfort or adverse reactions.
-The operator should carefully examine for signs of infection or bleeding at the treatment site at each visit, and if there are signs of infection, guide them to public health centers or hospitals to receive appropriate symptomatic treatments such as anti-inflammatory drugs or antibiotics.

• Precautions for Ear Acupuncture Treatment in Acupuncture Textbooks[60]

- Execute treatment after detailed medical consultation and accurate diagnosis.
- Consider achieving maximum effect with minimum acupuncture points. An average of 3~5 acupunctures points are used.
- Make an effort to decrease pain during needle insertion by making sure the patients understand by explaining before the procedure so that they are not nervous.
- Insufficient disinfection can cause skin infections, and sometimes even spread to cartilage, resulting in poor recuperation. In case of infection, administer anti-inflammatory treatments (e.g. antibiotics).
- Do not use needles in areas with inflammation, scars or tissue deformities in the ear.
- In case of sudden discomfort such as pain or stiffness in other parts of the body during the needle insertion, withdraw the needle.
- Thoroughly prevent vasovagal syncope and in case of vasovagal syncope, administer appropriate treatment and stabilization.
- When carrying out ear acupuncture and body acupuncture together, perform ear acupuncture treatment first, then body acupuncture treatment. This is because when symptoms are alleviated by body acupuncture, it is difficult to distinguish the tender points of ear acupuncture treatment.
- Avoid treatment on pregnant women in early stages of pregnancy (~14 weeks). Do not perform treatment on pregnant women who have experienced habitual miscarriages even after the second trimester of pregnancy. Rather than maintaining the intradermal needles, use acupuncture needles and remove after a certain period of time. Use seeds

such Melandrii Herba seeds rather than intradermal needles and shorten the maintenance period to 1~2 days, and only apply mild acupressure. In order to prevent miscarriage/prematurity, acupuncture points such as end genital, endocrine, abdominal, and pelvic cavity are contraindicated.

- Treatment is contraindicated in people with excessive fatigue, poor nutrition, extremely weak physique, and severe anemia. In cases where treatment has to be carried out, prevent vasovagal syncope in a comfortable supine position, and for elderly patients with unstable blood pressure, have them rest for 30 minutes while lying side-ways when performing body acupuncture, ear acupuncture or bloodletting. Observe while stabilizing for approximately 30 minutes after performing treatment.

6) Combining with Other Treatments

At the disaster site, ear acupuncture treatment can be used flexibly not only as a single therapy but also in combination with other treatments. Due to the nature of ear acupuncture treatment, the elements of exposure therapy, which are often used for trauma treatments, are not included, so it can be considered for patients who find it difficult to apply exposure therapy.

Moreover, because ear acupuncture treatment is simple and consumes little medical resources, it is possible to administer ear acupuncture treatment in combination to treatment patients who are already undergoing psychology treatment such as exposure therapy. Also, ear acupuncture treatment can be easily combined with acupuncture treatment, massage therapy and herbal medicine treatment.

2. Acupuncture Treatment

There are various studies and cases in which acupuncture, the main treatment me thod of oriental medicine, has been used for disaster trauma. Acupuncture can m anage both psychological and physical symptoms, such as pain, and has the adva

ntage of being a convenient treatment method to be used in combination with ot her treatments. In Japan, in the event of a major disaster, the disaster medical a ssistant team and Japan Medical Assistant Team are in charge of western medical treatment while the Japan Primary Care Association Disaster Relief Project[61], Di saster Acupuncture, Moxibustion and Massage Relief Project[62] and Association of Medical Doctors of Asia[63] participate in relief activities using acupuncture along with western medicine.

1) Evidence and Cases

The psychological relief team at Dongduk Hospital in Zhejiang Province, China, treated 91 earthquake trauma patients (mean age 35.01±19.32) diagnosed with acute stress disorder from May 20th 31st, 2008, with 24 in the cognitive behavioral therapy group and 67 receiving simultaneous treatment of cognitive behavioral therapy and acupoint motivation. Acupoint stimulation was administered to the left no gung (PC 8) at 50hz, for 30 minutes every day for a week[64]. The left no gung belongs to the pericardial meridians and is a gugeup acupoint used for heart pain, resentment, vomiting, and loss of consciousness. As the result of the treatment, the combination of cognitive behavioral therapy and acupoint stimulation significantly improved the emotional response and PTSD symptoms that occur when recalling trauma, hence the combination therapy was more effective than the single therapy. The combined treatment of acupoint stimulation showed a faster therapeutic effect than the single treatment cognitive behavioral group. This suggests that the combination of cognitive behavioral therapy and acupoint stimulation can be used as an effective treatment for acute disaster trauma.

Medical staff from the Department of Traditional Medicine at the Graduate School of Tohoku University in Japan provided medical support (acupuncture and massage) by visiting 7 shelters in Miyagi and Fukushima prefectures where the victims of the Great East Japan Earthquake lived[65]. A medical team from Tohoku University and medical staff from the Department of Traditional Medicine provided medical support and, when possible, the doctors conducted medical history listening. There were many requests for support from doctors as well as victims for acupuncture/massage treatment. A total of 553 patients were treated and the most common symptoms was stiffness of the shoulder blades. Treatment satisfaction was at 92.3%, and most responded that they felt comfortable not only physically but also mentally. When the acupuncture/massage treatment started, there was not enough tap water supplied to the shelters that the victims could not take a bath or wash their hands properly. During this period, only massage treatment was performed and acupuncture treatment was withheld due to the risk of infection. This shows that, in case of a disaster, acupuncture and massage treatment can be used effectively in combination with other medicaments because they have the effect of stabilizing both the body and the mind.

The Disaster Acupuncture, Moxibuston and Massage Relief Project Team in Japan

worked with victims of the 2011 Great East Japan Earthquake (March 27, 2011 – December 5, 2011) and the flood victims of 2015 (September 19, 2015 – October 23, 2015) and provided medical support with acupuncture and massage therapy[66]. At a retrospective analysis of the charts of 1042 victims of the Great East Japan Earthquake and 110 flood victims who received treatment, the most common complaints were shoulder pain and knee pain (67.6% among earthquake victims, 80.9% in flood victims) because pain-related problems can easily occur after living in an uncomfortable shelter long-term. Acupuncture and massage therapy significantly reduced the symptoms of victims and volunteer workers on the VAS scale (VAS 3.0 before treatment vs. VAS 1.0 after treatment, $p<.001$). Moreover, acupuncture treatment disaster medical support guidelines (published in April 25, 2016) developed by the Disaster Acupuncture, Moxibuston and Massage Relief Project Team in Japan was introduced and the following content was included: 1) Volunteer organization structure, 2) Communication with people in the affected area, 3) Data management, 4) Description of treatment procedure, 5) Cooperation with other support teams, 6) Necessary equipment and facilities, 7) Cases when acupuncture or massage therapy should not be performed, 8) Psychological counseling, 9) Coping with side effects during treatment procedure.
On August 24, 2016, magnitude 6 earthquakes struck central Italy, devastating Amatrice and several other villages, killing 300 people and displacing 30,000 people. The Lombard Association of Medical Acupuncturists and Acupuncture in the World relief teams provided acupuncture treatment for the earthquake victims from September to October 2016 for 5 weeks. Of these, the result of acupuncture treatment disaster relief activities on 41 people were analyzed and the effects acupuncture treatment on psychiatric symptoms such as anxiety, depression, and insomnia as well as pain of the survivors were reported[67]. A total of 4 treatments were performed every day for 4 consecutive days, along with moxibustion. After an evaluation of pain and general psychological state using VAS, 54% of the 41 patients included in the analysis after 3 treatments showed significant improvement in psychological symptoms and 60% in pain with no serious side effects. These results suggests that acupuncture treatment can be effective in treating PTSD and other accompanying physical symptoms in emergency situations such as disaster sites, and that it is a healthcare intervention that can be used in the early stages of mass trauma.

A review of 16 Chinese medical literature review on PTSD (4 comparative studies before and after of a single group, 12 controlled clinical studies) showed that combination therapy of not only Chinese medicine, but also general acupuncture, electro acupuncture, ear acupuncture and moxibustion as well as combination therapy of acupuncture and psychotherapy was used, in addition to western medicine and Chinese medicine being used in combination frequently on PTSD patients[68]. Electro acupuncture, body acupuncture, ear acupuncture and moxibustion were used, but electro acupuncture was the most frequently used treatment. In most studies, the Chinese medicine treatment

group showed significantly better results than the control group, and compared with western medicine treatment group, the effect was either equal or better.

According to the analysis of 7 studies (709 participants) included in a systematic literature review on the effects of acupuncture treatment on PTSD published in 2018, the acupuncture treatment group was more effective in post-treatment and follow-up investigations than the control group, and was shown to be effective in alleviating depressive symptoms. In addition, there were few side effects related to acupuncture treatment[69].

Eight studies using Korean oriental medicine treatment were confirmed in a study on the clinical research trend of oriental medicine treatment for PTSD[70]. Five cases and case groups, two comparative studies before and after of a single group, and one quasi-randomized controlled study were reported. The events that induced PTSD included traffic accidents, assaults, sexual violence, and witnessing family suicide. Various treatment methods were used, including acupuncture, herbal medicine, moxibustion, cupping, chuna manual therapy, physical therapy, gigong meditation, and oriental medicine psychotherapy (M&L trauma psychotherapy program).

2) Clinical Considerations - Acupuncture

If there is a risk of infection because bathing or hand hygiene is not being properly performed due to the life in the shelter, be careful of infection that may be associated with acupuncture treatment. If the clinic can utilize an electro acupuncture device, consider performing electro acupuncture on the acupuncture points for each symptom. Electro acupuncture is a continuous wave, hence should be performed at an intensity that the survivors can tolerate.

① Hypertension/Anxiety[71]:

Sinmun, naegwan, joksamni, baekhoe, sasinchong, sinjeong, pungji

② Fear[72]:

Nogung, sinmun, yongcheon / Stimulate shipjeong acupoints (eunbaek, yetae, jungchung, etc.) when necessary.

③ Depression/Grief[73]:

Baekhoe, yyintang, yongcheon, joksamni

* Liver-gi stagnation- taechung, yongcheon, sinmun, naegwan; Giulhwahwa- haenggan, hyeopgye, sob; * Phlegm-gi stagnation - punning, yangneungcheon, jungwan; * Heart-spleen deficiency- sameumgyo, joksamni; * Liver-kidney eum deficiency- taeyeon, sameumgyo; * Eumheohwawang- taeyeon, johhae

④ Anger/Irritability:
Taechung, haenggan, sameumgyo

⑤ Insomnia/Nightmares[74]:

Baekhoe, sasinchong, sinjeong, anmyeon(安眠: ½ between yepung, pungji acupoint), sinmun, naegwan, sinmaek, johhae, sameumgyo, joksamni, taechung, etc..

* Heart-spleen deficiency - - sinmun, joksamni；* Simsinbulgyo- johhae, nogung；* Ganulhwahwa- taechung, hyeopgye

⑤ Loss of appetite/dyspepsia [7576]:
Jungwan, cheonchu, yongcheon, naegwan, joksamni, taechung, gongson

⑥Fatigue/Burn out[77]:
Taebaek, joksamni, gwanwon

⑦ Headaches[78]:
Baekhoe, pungji, yongcheon, jokimeup
* Proximal Acupint: baekhoe, pungji, sasinchong, taeyang, yintang, solgok, hyeollo, gakson, cheonju, duyu , sajukgong, gokbin, hayeom, hyeolli, sinjeong, asi, etc.
* Distal Acupoint: yongcheon, taechung, naejeong, gollyun, jokimeupgi, gokcheon, haenggan, yeolgyeol, punning, yangneungcheon, guheo, oegwan, jungju, taeyeon, etc.

⑧ Dizziness[79]:
Baekhoe, sasinchong, pungji, taeyang, yintang, sangseong, solgok, gyeonjeong, yongcheon, taechung, hyeonjong
* Wind-plegm: punning, naegwan; * Complex syndrome of eum deficiency and yang excess - taeyeon, gansu; * Ganhwasangyeom- haenggan, taechung; * Gihyeolhyuheo-joksamni, hyeolhae

⑨ Pain: Acupoint by symptoms
Neck pain, Shoulder and back pain-gyeonjeong, pungji, daechu, gyeonoeyu, choellyo, gokwon, cheonjong, bino, geogol, jungbu, etc.
Backache[80]-yoyanggwan, myeongmun, simyu, daejangyu, sangnyo, charyo, wijung, gollyun, yangneungcheon, sameumgyo
Knee pain- yangneungcheon, eumneungcheon, joksamni, yanggu

Add acupoints in accordance with symptoms.
Sa-am acupuncture (taebaek taeyeon +, gokji oegwan -) can be taken into consideration.

3. Herbal Medicine Treatment

There are various studies and cases in which herbal medicine, the main treatment method of oriental medicine, has been used for disaster trauma. Herbal medicine has no risk of dependency and can be actively applied to various physical symptoms. Hence, herbal medicine treatment can be widely administered for acute stage survivors for whom it is difficult to prescribe psychiatric drugs. In addition, herbal medicine with the characteristics of improving bodily functions can be used for exhaustion, loss of appetite, and hypothermia which are difficult to treat with general medical treatment. In particular, herbal medicine granules are more useful in disaster sites since they are easier to prepare, transport, store and control the capacity of than general medical tablets.

1) Evidence and Cases

Researchers from the Department of Oriental Medicine at Tohoku University in Japan conducted a systematic literature review on the use of herbal medicine after large-scale disasters[81]. As a result of the review, two randomized controlled studies, 3 retrospective observational studies, and 7 case studies were confirmed, and the included studies showed the applicability of herbal medicine at disaster sites. It was shown that herbal medicine was used for PTSD and other general post-disaster physical and mental symptoms (cold, tonsillitis, lower body temperature, enteritis, rhinitis, itchy eyes, dry eyes, constipation, general weakness, abdominal pain, edema, numbness in lower extremities, dizziness, insomnia, anxiety, irritability).

Researchers at the National Sendai-Nishitaga Hospital in Japan studied 48 outpatients amongst the survivors of the Great East Japan Earthquake with chronic PTSD who had a score of 25 or higher on the Impact of Event Scale (IES-R) and administered Saiko-Keishi-Kankyoto 3 times a day for two weeks to the test group while the control group waited without treatment[82]. After two weeks of treatment, the test group had significantly improved anxiety on the trauma scale, recollection of trauma (flashback), and avoidance reaction compared to the control group, and there were neither side effects nor abnormal findings the blood tests. In other words, Saiko-Keishi-Kankyoto was effective and safe in relieving the chronic trauma symptoms of disaster survivors. Based on such evidence as this, Saiko-Keishi-Kankyoto was introduced as a non-psyc hological treatment in the Posttraumatic stress disorder prevention and treatment g uidelines (2018) of the International Society for Traumatic Stress Studies[83].

A randomized, placebo-controlled, double-blind study was conducted at the Shanghai Jangjiang Hospital in China where herbal medicine was administered to survivors diagnosed with PTSD 5 months after magnitude 8.0 Sichuan earthquake in 2008[84]. The test group (123 people) took Xiao-Tan-Jie Yu-Fang(消痰解郁方)[85] and the control group (122 people) took a placebo herbal medicine twice a day for a total of 8 weeks. Compared to the control group, the test group showed a significant improvement in the Symptom Checklist-90-Revision (SCL-90-R) scale and treatment response rate, and no serious side effects were reported. In particular, the group taking Xiao-Tan-Jie Yu-Fang improved not only anxiety, but also sleep status.

After the sinking of the Sewol ferry in 2014, the Korean Medical Association provided medical support using herbal medicine in three shifts 24 hours a day[86]. A total of 1860 patients who received oriental medicine treatment mostly complained of pain, but also complained of various symptoms such as dyspepsia, cold, headache, dizziness, exhaustion, anxiety and insomnia. Patients had specifically chosen oriental medicine treatment because of psychiatric symptoms as well as exhaustion and pain. The doctors were in charge of emergency treatment for patients, and oriental doctors treated mainly

relief workers. The most prescribed medicine were Ssangwhatang, Su He Xiang Wan and Ojeoksan. Maekmoondong-tang and Socheongryong-tang was the most effective for relief workers complaining of runny nose, stuffy nose, and dry cough.

Researchers from the Department of Oriental Medicine at Tohoku University Hospital in Japan retrospectively surveyed and analyzed the oriental medical treatment charts of 236 patients who received herbal medicine treatment for 10 weeks at an evacuation center in Onagawa, Miyagi Prefecture, which were heavily damaged after the Great East Japan Earthquake[87]. It was very cold for two weeks immediately following the earthquake but due to lack of heating facilities, the survivors showed symptoms of cold and hypothermia. Galgeun-tang and Gyeji-tang was used for cold symptoms and Ginseng-tang and Danggui-Sayuk-Ga-Osuyu-Senggang-Tang was used for hypothermia symptoms. Specifically, herbal medicine that warms the body, which is not found in westerb medicine, was effective for hypothermic elderlies with low basal metabolism. Because it was difficult to secure clean water and washing hands was not possible, gastroenteritis symptoms appeared and in cases where intestinal drugs and antidiarrheal drugs did not improve the symptoms, Oryeongsan was prescribed. From 2 to 6 weeks, hedoro (sewage and factory waste water which have been solidified on the beach) and silt dried and rose to air, worsening the air pollution and resulted in more allergies and respiratory symptoms such as severe coughing, runny nose, and pruritus in the eyes for which Socheongryong-Tang and Maekmoondong-tang was prescribed. From week 6 to 10, mental symptoms such as insomnia, restlessness, anxiety as well as various physical symptoms increased due to the stress caused by long-term shelter life for which herbal medicine with stabilizing effects such as Yeokhan-san and Gamiguibi-tang was prescribed. When an elderly uses a strong sleep inducer, it is difficult to evacuate during an aftershock as they become semiconscious and there is a risk of falling, but herbal medicine has the advantage of not lowering muscle tension while improving sleep through its mild sedative action. There were many cases of hypothermia in the elderly in the beginning phase of the disaster, and there were many people who complained of mental symptoms from the 6th week onwards. Although western medicine was already being prescribed to many disaster victims, there were many cases in which improvement in symptoms were seen due to the addition of herbal medicine.

The Association of Korean Medicine operated the ‘Covid-19 Oriental Medical Treatment Phone Consultation Center’ in Daegu, where Covid-10 occurred on a large scale at the beginning of the epidemic[88]. A volunteer oriental doctor at the phone consultation center would generally prescribe Qingfei Paid-up decoction for patients who tested positive for Covid-19, but prescribed other various herbal medicine deepening on the accompanying symptoms for patients who showed mild respiratory symptoms. Socheongryong-Tang for rhinitis and Gamiguibi-tang for anxiety and insomnia were representative prescription. Although respiratory symptoms were mild, many confirmed patients complained of various emotional problems such as anxiety,

depression, insomnia, anger and poor concentration due to anxiety about their disease worsening and isolation. To these patients, the phone consultation center provided Gamiguibi-tang along with meditation audio files (YouTube link provided).

As a result of reviewing 16 Chinese medical literature on PTSD (4 comparative studies before and after of a single group, 12 controlled clinical studies), various Chinese medicine treatment were used with not only herbal medicine but also general acupuncture, electro acupuncture, ear acupuncture and moxibustion being used in combination as well as a combination of psychotherapy and acupuncture, and western medicine and Chinese medicine were often combined [89] . The herbal prescriptions used were Tang Yu Shu granule (糖鬱疏), bailing jieyu granule(白龍解鬱颗粒), and qingxinjeiyu-tang decoction.

2) Clinical Considerations

① Hypertension/Anxiety[90]:

Gamisoyo-San: Deficiency syndrome. Blood deficiency. Consider for menopausal women.

Sihosogan-san: Excess syndrome. Liver qi depression. Tightness of the chest.

Sanjoin-Tang: Deficiency syndrome. Accompanying mental restlessness and insomnia.

Sihogayonggolmoryeo-Tang: Excess syndrome. Soothing effect. Accompanying depression.

OnDam-Tang: Excess syndrome. Acute anxiety.

Xiaotan-Jieyu Prescription (Xiaoyao Powder)[91]: Add Soyo-san and heat-clearing herbs (qingre) (Rhizoma Coptidis, Wine-Treated Rhubarb Root, etc.), Mind-tranquilizing herbal medicine(Pinellia ternata, Citrus unshiu Markovich, Acorus gramnieus), Calming medicine(Fossilia Ossis Mastodi, Ostrea gigas Thunberg).

Jie Yu Wan: Combination of Soyo-san and Gammakdaejo-Tang.

Hwangryeonagyo-Tang: Damp-heat syndrome, accompanying tightens of chest and insomnia.

② Fear[92]:

Gyejigayonggolmoryeo-Tang: Soothing effect. Sihogayonggolmoryeo-Tang rather than Deficiency syndrome. Accompanying cold constitution, abdominal pain, and poor bowel movement.[93]

Guibi-Tang: Deficiency syndrome. Heart and spleen deficiency. Accompanying insomnia, depression, fatigue, loss of appetite.

Cheon-Wang-Bo-Sim-Dan: Deficiency syndrome. Heart-Blood deficiency.

Woo-Hwang-Chung-Sim-Won: Accompanying acute hypertension, confusion / short-term use.

③ Depression/Grief[94]:
Shihosogan-Tang: Excess syndrome. Liver-Qi stagnation. Accompanying tightness of the chest.
Gamisoyo-San: Liver-qi stagnation and spleen deficiency. Blood deficiency. Menopausal women.
Banhahubak-Tang: Excess syndrome. Qi deficiency. Physical symptoms of lump in throat sensation.
Guibi-Tang: Deficiency syndrome. Heart and spleen deficiency. Accompanying insomnia, anxiety, fatigue and loss of appetite.
Sihogayonggolmoryeo-Tang: Excess syndrome. Soothing effect. Accompanying depression.
Gyeji Bokryeong-hwan: Excess syndrome, blood stasis syndrome. Menopausal women, Accompanied by burning sensation and hot flushes.
Yukgunja-tang: Deficiency syndrome, Accompanying loss of appetite and dyspepsia.

④ Anger/Irritability[95]:
Yigan-San: Excess syndrome. Accompanying headache.
Hwang-Ryeon-Hae-Dok-Tang: Damp-heat syndrome. Accompanying constipation.
BunsiGiEum: Excess syndrome. Symptoms of heat due to Liver-Qi stagnation.
Sihogayonggolmoryeo-Tang: Excess syndrome. Soothing effect. Accompanying depression.

⑤ Insomnia/Nightmare[96]:
Sanjoin-Tang: Deficiency syndrome. Palpitation, Accompanying mental restlessness
Guibi-Tang: Deficiency syndrome. Heart and spleen deficiency. Accompanying anxiety, depression, fatigue and loss of appetite.
Ondam-Tang: Excess syndrome. Acute anxiety.
Gamisoyo-san: Deficiency syndrome. Blood deficiency. Menopausal women.
Cheonwangbosi-dan: Deficiency syndrome. Heart-Blood deficiency.

⑥ Loss of Appetite/ dyspepsia [97]
Naesowhajung-Tang: Simultaneous occurrence of deficiency and excess syndrome. Frequent dyspepsia.
Banhasasim-Tang: Simultaneous occurrence of deficiency and excess syndrome. Accompaniment of epigastria fullness, heartburn, burning sensation of lower extremities.
Yukgunja-tang: Deficiency syndrome. Spleen deficiency resulting in pleghm turbidity. Stagnation of fluid retention. Accompanying full body fatigue, epigastria fullness, feeling full and frequent burps.
Soyo-san: Simultaneous occurrence of deficiency and excess syndrome. Liver-qi stagnation and spleen deficiency. Accompanying depression, anxiety and insomnia.

Sihosogan-san: Excess syndrome. Disharmony of liver and stomach. Accompanying depression, anxiety and insomnia.
Pyeongwi-san: Excess syndrome. Dampness retention in the spleen and stomach. Accompanying epigastria pain, nausea and dizziness.

⑦Fatigue /Burn out[98]
Bojungikgi-Tang: Spleen-Qi Deficiency. Deficiency of spleen and stomach-qi.
Guibi-tang: Heart and spleen deficiency. Accompanying anxiety, depression, fatigue and loss of appetite.
Ssanghwa-Tang: Qi and blood deficiency.
Palmul-Tang: Qi and blood deficiency.
Sipjeondaebo-Tang: Qi and blood deficiency.
Yukmi/Palmijihwang-hwan: Kidney Yin deficiency/ Kidney Yang deficiency Elderly.
Gamisoyo-san: Liver-qi stagnation and spleen deficiency. Blood deficiency. Menopausal women.

⑧ Headache[99]:
Chungsanggyuntong-Gagam: Damp-heat syndrome. Used for any headaches.
Banhabaekchulcheon-Matang: Spleen deficiency resulting in pleghm turbidity. Poor digestion. Accompanying dizziness.
Chungungdajosan-Gagam: Various symptoms due to pathogenic qi of wind-cold. Cold-related headaches. Beware of fever.

⑨ Dizziness[100101]:
Banhabaekchulcheon-Matang: Spleen deficiency resulting in pleghm turbidity. Poor digestion.
Cheonmagudeungem-Gagam: Surging of Liver-yang. Burning sensation, increase in blood pressure.
Younggyechulgam-Tang: Water-qi surging up to chest (水氣凌心證). Stagnation of water in stomach (Abdominal pain in the lower part of the stomach), palpitation, headaches.
Jaemgunbi-Tang: Qi and blood deficiency +pleghm-retention syndrome. Chronic development.

⑩ Pain[102]:
Ojeok-san: Cold constitution. Acconpanying dyspepsia.
Danggwuisu-san: blood stasis syndrome. Acute pain.
Ssanghwa-tang: Deficiency syndrome. Chronic pain. Accompanying muscle tension, cramping and fatigue.

4. Emotional Freedom Technique

1) Introduction

Emotional Freedom Techniques (EFT) is a psychiatric approach that controls energy by physically stimulating meridians and acupuncture points and was registered as a new medical technique for PTSD in 2019. EFT is a treatment that combines short-term exposure (symptom selection and evaluation) with physical intervention (acupoint tapping) and cognitive factors (such as affirmation). Church et al. (2017) reviewed studies applying EFT to soldiers with PTSD and found that EFT improved PTSD and its accompanying symptoms in a short-time and it has also been shown that EFT modulates stress hormones and limbic system function and improves various health indicators[103]. In addition, this study also suggested advantages of EFT such as (1) the treatment effect is clear and the indications are wide, (2) the treatment period is short, (3) there are few side effects, (4) the procedure is simple and easy to learn, (5) it can be used for both physical and psychological symptoms, (6) it has been found to be useful and effective as a large-scale group treatment, and (7) it can be applied online or over the phone.

EFT is particularly useful as a psychotherapy related to post-traumatic responses, and related clinical studies have also shown significant improvements in pain, anxiety, depression, and PTSD scales. EFT is a self-regulating technique that can be used to educate disaster survivors to restore a sense of control and cope with symptoms. EFT has a high therapeutic effect, a relatively short treatment period, low risk of side effects, small training period required and can control both physical and psychological symptoms.

Because EFT has such advantages, it can be used as a stabilization technique at disaster sites[104]. There are reports of EFT being used by individual volunteer workers or in collaboration with local and international NGOs to be used at several disaster sites (Hurricane Katrina 2004, ear quake in Pakistan in 2008, tsunami in Thailand in 2004, genocide survivors in Rwanda, earthquake in Haiti in 2010, trauma of veterans in the Iraq war, Mexican Civil War)[105].

2) Things to consider before implementation

① To avoid the patient’s reluctance to a new treatment method interfering with the treatment, briefly explain the treatment in easy-to-understand terms . (Example: “There is a certain acupoint that calms the mind. Tapping on that area will relax the mind more.”, “Reason for your heart aching is because the energy is blocked so we will trying releasing the blocked energy by tapping acupoints,”)

② Along with the treatment, proceed with education step by step. During the treatment process, use the entire procedure and in self-treatment, teach continuous tapping first so they may utilize it in daily life. As treatment session are repeated, teach other processes sequentially.

③ Examples of various affirmation and acceptance suitable for disfavor trauma during the set-up phase are presented below. An oriental doctor selects appropriate ones for the patient and presents it to the patient, and conduct EFT with the ones the patient agrees with.

3) Basic Protocol(Appendix 10. Refer to Self-healing Study sheet)

① Identification of the problem

Specify the problem, symptom or discomfort that will become the target.

②Check for discomfort before treatment

On a scale of 0 to 10, the degree of discomfort or pain caused by the problem is assigned.

③ Precatory work (SET-UP, correcting energy)

It is the process of starting to tap, and while continuing to tap the opposite acupoint (Houxi acupoint) with two fingers (mainly index and middle finger), decide on a simple phrase below and repeat it three times. The phrase is about acknowledging the problem and nevertheless accepting yourself.

‘Even though I have _____, I sincerely and fully accept myself completely.’

When targeting the current situation, the phrase is set to “I’m anxious right now, but…/I’m uncomfortable right now, but…/I want to avoid it right now, but…” and when targeting physical symptoms, the phrase is set to “Although my heart is beating…/Although I’m getting cold sweats…/Although I have a headache…”.

Explain that it is a way to correct the energy (Removal of polarity reversal).

Table16. Affirmation(Affirmation of acceptance) Example

(Past)
Even though thinking about it is painful and gets me anxious and scared
Even though I am so bewildered, anxious and I don't know what to do because it is so sudden
Even though I am angry, resentful and embarrassed that this happened to me of all people
Even though I feel so sad and upset just thinking about it

(Present, Myself)
Even though I am furious and annoyed
Even though I feel so lonely and sad
Even though it is hard and painful enough to die
Even though I get startled so easily
Even though my heart pounds and it feels scary
Even though it feels like my life is uprooted and disappearing
Even though it feels like my life is irreversibly ruined
Even though I don't want to do anything because I am depressed and lethargic
Even though it is painful because I have so many thoughts that I can't control
Even though it is scary and difficult because bad thoughts (suicidal thoughts, self-harm thoughts) keep coming up

(Present, External Stimulus)
Even though the world is resentful, and the people hateful
Even though I am flustered and scared because the world is so unfamiliar
Even though it's painful because everything feels dangerous
Even though it's painful because I care too much about other people's words and gaze
Even though it's so difficult to go about my daily life and meet people
Even though I only want to avoid the world and people

(Present, Physical Symptoms)
Even though I can't sleep because I'm so nervous and have a lot of thoughts
Even though I get nervous and get headaches so easily (even though the area of pain hurts)

(Future)
Even though I don't know how to live my life in the future and it is uncertain and far-fetched
Even though I'm scared and anxious about what will happen in the future
Even though I'm worried that something painful will happen right away
Even though it makes me more anxious to think about living this way forever

(Self-image)
Even though I feel so ashamed, pitiful and pathetic
Even though it feels sinful to live like this
Even though it is painful because of regrets and remorse
Even though I feel like an unlucky person

I sincerely accept myself as is from the bottom of my heart.

Table17. Examples of affirmation of acceptance by stage

1) Acute phase (affirmation of acceptance)

During the acute phase, the key is to "restore a sense of safety". In the acute phase, the following affirmations of acceptance are made and applied in the form of choice method for the symptoms currently experienced by survivors. It is more desirable in the acute phase to set the key of "affirmation of choice" to "the direction of recognizing that I am safe now" rather than "acceptance". At this time, the therapist does not ask about the disaster experience first, and makes an acceptance of affirmation using the following affirmation of choice form as expressed by the survivor.

(Even though) I am _______________, I choose to recognize that I am safe now.

Examples)
Even though I am dazed and restless,
Even though I am anxious and scared,
Even though something so sudden happened,
Even thought it was so shocking and surprising,
Even though my heart is pounding and I feel frightened,
Even though it feels like my life is uprooted and disappearing,
Even though I am so confused and I don't what is what,
Even though that moment keeps coming back to me unknowingly,

2) Sub acute (Affirmation of acceptance+ affirmation of choice)

EFT can be applied focusing on the ①hypervigilance ②depression/grief reaction/guilt ③distrust/isolation that occurs during the sub-acute period. As it has been more than 1 month, the EFT affirmation of acceptance or affirmation of choice can be combined to apply the affirmation of acceptance or affirmation of choice that the survivor is comfortable with.

"(Even though) I am _______________, I choose to recognize that I am safe now.

The me of now is safe.

I sincerely accept myself as is from the bottom of my heart.."

Example)

① Examples of hyper vigilant affirmation of acceptance

Even though I feel scared and afraid because I remember that time without even realizing it,
Even though I am startled at even a small stimulus,
Even though my body and neck tenses because of tension and anxiety,
Even though my heart keeps beating fast because of anxiety,
Even though I am short of breath, feel stuffy and anxious,
Even though I am afraid and scared of having nightmares,

② Examples of depression/prolonged grief disorder/guilt affirmation of acceptance

Even though my heart feels pent up with sadness and there are only tears,
Even though I am sad and helpless,
Even though I cannot accept what happened to my beloved (subject),
Even though if I had acted a little differently then that person would have been safe and I feel regret and guilt,
Even though I can't believe that I can't go back to the way things were,

▶ However, in cases of severe self-deprecation or self-blame, seek professional help.

③ Examples of distrust/isolation affirmation of acceptance

Even though no one can truly understand my mind and my situation
Even though I feel offended and angry that I am feeling pitied,
Even though I am so lonely and feel as if I have been left alone,
Even though I am scared that this will happen again,
Even though I am scared and anxious because the world seems like such a dangerous place,

3) Chronic

Even though the anxiety doesn't go away,
Even though I get anxious and worried and question why these symptoms aren't getting better,
Even though I get frustrated and angry and question how long I have to live like this,
Even though I feel pent up and frustrated and question when will I, when will this situation become better,
Even though I feel so scared and hopeless, questioning whether I can go back to the way things were,
Even though I feel that it is unfair and angry and question why it happened to me,

I sincerely accept myself as is from the bottom of my heart.

④ Contiguous Tapping (Sequence, Opening energy of 14 meridian pathways)

Next, tap each part of the body about 7 times and repeat the phrase that is the reminder phrase. The order of the body parts is the crown of the head, the beginning of the eyebrows, the side of the eyes, under the eyes, under the nose, chin, the beginning of the clavicle, and under the armpits. Reminder phrase are chosen as short phrases that can simply identify the problem. The reminder phrase will be different depending on the problem. For example, it can be 'this shoulder pain', 'that accident', 'nightmare' and so on.
Explain that it is a method of actively communicating the energy of the mind.

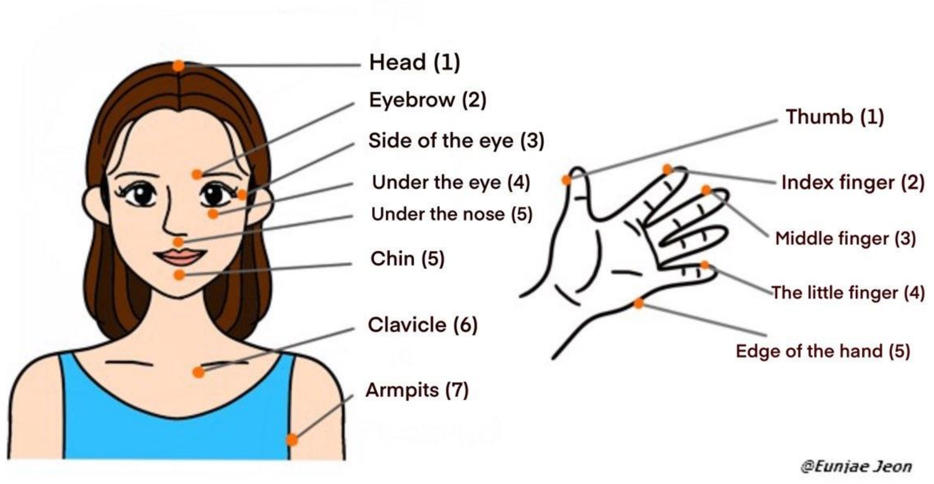

Figure8. Continuous tapping Acupoint

Table17. Examples of Reminder Phrase

Worrying, gloomy, troublesome, terrified, scary, terrified, angry, anxious, nervous, restless, jittery, fretting, uncomfortable, painful, frustrated, suffocating, sad, heartbreaking, yearning, tear-inducing, to get choked up, moody, sorrowful, bitter, heartrending, brooding, wretched, terrible, miserable, regretful, heart aching, tragic, unfair, coldhearted, dejected, lonely, empty, despondent, desolate, depressed, lethargic, melancholic, tired, bored, exhausted, excruciatingly tiresome, irksome, boring, weary, despair, disappointing, jaded, confused, embarrassed, surprised, disconcerted, shameful, angry, burning, upset, affronted, surge of resentment, feeling victimized, rising anger
Heart pounding, insatiable anger is rising within my chest, head hurts and is throbbing, shoulder and neck (area of pain) is stiff and aches, entire body aches and is tense, whole body feels so heavy, feel so frustrated, can't sleep, have no energy, body becomes unknowingly tense.

⑤ Reevaluation of discomfort

If the discomfort score set earlier does not decrease after performing step 1 to 4, repeat until it decreases or the mind becomes more stable. When repeating the steps, the affirmation of acceptance and reminder phrases should be slightly modified to reflect the changed aspects. For example, it can be expressed as "Even though there is still a little ___, remaining_____".

EFT is a stabilization technique that combines cognitive exposure, language processing, and acupoint stimulation. It can be used when anxiety or avoidance is severe due to re-experiencing during trauma recall and can also be used for physical symptoms. While general trauma psychotherapy is detailed and specifically deals with elements related to traumatic memories from various aspects, EFT deals with the overall discomfort and promotes stability while minimizing side effects such as emotional heightening and dissociation during treatment.

4) Choice Method - Additional Protocol

Explain that this is to clarify the direction of the treatment in accordance with what condition the patient wants to be in.

Table18. Examples of Choice Method

How would I feel if all my heartaches were gone?
How would I feel if all my problems were solved?

I choose to be ~~~.

Positive affirmation

I don’t hurt myself.
I don’t blame myself.
I don’t underestimate myself.
I truly understand and love myself.
I am getting healthier.
I am feeling more and more comfortable.

1) Examples of Acute Phase Choice Method

I know that I am safe now.
I am safe.
I choose to be comfortable.
I choose to restore my body and mind to health.
I choose to recover and be comfortable.
I choose to restore my body and mind healthily.
I choose to be healthy, comfortable, and happy.
I choose to feel relieved and carefree.
I choose to relax my mind and be comfortable.
I choose to sleep deeply.

2) Examples of Sub acute & Chronic Phase Choice Method

I am the protagonist of my life.
I am a deeply rooted tree.
I have the right to feel happy and comfortable.
I do not blame or hate myself.
I sincerely understand and love myself.
I am living a life of truly understanding and loving myself.
I am gradually recovering my body and mind.
I have enough wisdom and strength within me to control my emotions.
What is happening now is only a part of my life. I can go down my own path.
The past had already passed and the future is yet to come. I live my life to the fullest here and now.
I accept the past that I can do nothing about, and I do my best in the present and future that I can do something about.
Even if it's a little lacking or a little overflowing, I'm going down my own path, so it is fine as it is.
What is happening now is only a part of my life. I can go down my own path.
The past had already passed and the future is yet to come. I live my life to the fullest here and now.
I accept the past that I can do nothing about, and I do my best in the present and future that I can do something about.

5) Group Treatment

Group therapy can help participants feel empathized and comforted, and can also experience the Borriwng Benefit effect.

It can be planned for 4 weeks once a week, and the duration of each session to an hour, but it can be set flexibly according to the situation. Self-learning audio recording files and self-study papers are provided to that the survivor can practice each day individually.

6) Points to note

EFT is a noninvasive method of patients tapping their own acupoints, and since it does not directly harm the subject, there is no problem in safety according to the procedure, and no side effects have been reported in previous studies[106]. However, rather than directly recalling traumatic memories, apply EFT mainly to the physical/psychological symptoms complained by the survivors. But patients who complain of symptoms such as severe agitation/restlessness, dissociation, delusions, hallucinations, and suicidal thoughts or shows response to treatment after 3~4 sessions, other psychological or drug treatments should be considered, or refer the subject to a related expert such as a psychiatrist or a psychologist.

5. Stabilization Techniques

Stabilization technique is the most basic and essential treatment method for treating acute disaster trauma. In a state where the limbic system, including the amygdala, is over activated and the prefrontal cortex activity is degraded after experiencing a disaster, trauma survivors learn to observe and focus their emotions through stabilization techniques, activating the prefrontal function and help manage the physical sensory, emotional, and cognitive disorders related to trauma.

As a result of this study, it was shown that the stabilization technique had a high remission rate of PTSD and was very effective in reducing symptoms, as well as the flexibility safety and independence it has as a treatment method[107]. The stabilization technique used were mental education, distance technique, handling flashbacks, improvement of emotion regulation, resource activation, improving control ability, coping technique reinforcement, and enhancing sense of security. Moreover, in a study

using image guides of digital audio files for refugees who have suffered war trauma, it was shown to be helpful and well-received by the refugees, and the technique of ‘safe place visualization was particularly well-received [108].

The image stabilization technique[109] consists of three phases with the first phase consisting of conscious breathing that allows the participants to relax, focus on the present and enter a state of self-engagement. The second phase, the body scan, was original developed by Jon Kabat-Zinn[110] and described by Louise Reddemann and aims to teach the participants self-awareness, allowing them to experience their own body by focusing their minds on other body parts. The third phase uses either the ‘safe place visualization’ or the ‘tree exercise’[111]. The ‘inner safe place’ technique encourages individuals to imagine themselves in a real or imaginary places where they feel safe and comfortable. During the ‘tree exercise’, individuals imagine a tree as a symbol of power and nutrition that evokes a feeling of inner stability, comfort and energy. This is under the same context as resource mindfulness.

Pierre Janet’s evidence-based trauma approach model (1989) suggested a threep-stem trauma treatment approach[112]. Stage 1 is symptom relief and improving stabilization ability (expansion of resources), stage 2 is the response (defense, orientation, etc.) and trauma memory processing work (review and reevaluation), and stage 3 is individual integration, solidification of relationship and intimacy, and rehabilitation. In other words, stabilization stage is the foundation of trauma treatment.

1) Components of Stabilization[113][114]

Build a therapeutic alliace and promote sense of safety while normalizing and validating the patient experience. Proceed with education on post-traumatic stress and treatment while giving hope and emphasizing strength. Collaborate to great treatment goals, cope with symptoms and educate on skills to control the symptoms.

① Therapeutic alliance

Establishing the therapist-patient relationship is the basic element of any treatment. A positive therapeutic relationship is one of the most important elements of successful trauma treatment and can provide great comfort and support to trauma survivors.

② Sense of security

Safety related to trauma treatment must first be isolated from the surrounding environment as safely as possible, and it is the improvement of self-destructive thoughts and behaviors, and resolution of risks associated with self through the development and acquisition of self-care skills[115].

③ Normalization and validation

The therapist should remind the disaster survivors that the symptoms are a type of

coping mechanism and are a normal reaction. Educate the patients that the symptoms they are experiencing are not negative, but a healthy adaptation or response, or self-protection.

④ Psychological education[116]

Psychological education should provide correct information about the characteristics and effects of trauma, and enable the integration of this new information into the knowledge system of disaster survivors. When one understands that the symptoms are a natural reaction that can that can occur after experiencing a disaster, one can you reduce the feeling shame and guilt, and alleviate the traumatic reaction.

⑤ Cycle of hope and strengths

In the case of a large-scale disaster, the worldview that they had before in that the world is a safe place collapses, and they experience that there is no hope for the future. Ad this time, having hope, such as the expectation of a possibly positive future or result, and discovering positive resources are important healing elements.

⑥ Formation of treatment goals

Establish and share achievable treatment goals and focus on overall daily living and functional recovery rather than simple symptom or discomfort relief.

⑦ Training in symptom coping and control skills

The goal of technique education is to teach them how to control the powerful emotions, impulsive behaviors, and self-deprecating thoughts and behaviors that they frequently experience and interfere with their daily life, and to cope with these symptoms on their own by learning how to control them.

⑧ Understanding and application of oriental medicine

Therapeutic alliance refers to the aspect of the relationship between the patient and the therapist. In oriental medicine, the need for the therapist to cultivate themselves and examine their minds in the patient-therapist relationship is called YiDo-Therapy(以道療法) and Heosimhapdo (emptying one’s mind and looking in another’s view). By viewing disaster victims with an attitude of empathy and acceptance at the disaster site as a being of with the power of organic healing, relationships can be built and a place of safety and reassurance can be made. In oriental medicine, it is possible to explain the symptoms that appear according to each seven modes of emotion and explain the meaning behind each. Psychological education can be seen as equivalent to Sasenggong-Therpay(思勝恐) of Ojisangseung-Therapy within oriental medicine psychotherapy. Knowing your reactions to trauma and know that these are normal

reactions can help manage the feeling of fear. The cycle of hope and emphasis on strengths is similar to Jieongoronbeop in oriental medicine psychotherapy, and corresponds to the oriental medicine treatment principle that boosts energy and drives moral through the method of Bujeonggeosa.

2) Stabilization technique

① Psychological education

Psychological education is the task of explaining the psychological process and changes to disaster survivors and helping them to understand. Psychological education is an evidence-based core component of all trauma-related treatment. Education is generally conducted after basic relationship between the therapist and survivor have been established. It helps to change the perception of disaster survivors so that they can look at the negative symptoms that they are uncomfortable with from the perspective of a healthy reaction, adaptation, coping mechanism, and self-protection. Once the survivors learn that their reactions are natural, predictable and something that many people experience, their feelings of anxiety, shame and guilt will be reduced[117][118].

Even a short psychological education of children amongst the 2011 Great East Japan Earthquake survivors was able to effectively reduce the psychological impact of children[119]. Moreso, psychological education for 41 disaster survivors in Libya was able to reduce PTSD symptoms in survivors[120].

Disaster survivors who have received an unexpected shock are very nervous and their comprehension and judgment is much lower than usual. Therefore, it is important to approach them carefully and build a relationship found in trust. It is best to use simple language, pictures, or props that the survivor can easily understand.

• Goal: To explain the concept of trauma, normalize the client's reactions and help them to understand their own reactions.

• Considerations:

- If communication with the subject is possible, explain the results of the examination and treatment plan, and conduct psychological education.
- Abdominal breathing can be taught first for relaxation.
- If possible, proceed as a group.
- If face-to-face is difficult, it can be carried out online.
- For review and repeated learning, provide educational materials containing psychological education or use websites (Example: National Center for Disaster Trauma's disaster mental health information, notice of information notification)
- Using a method that can disperse hypervigilance can be helpful for an effective education. Provide herbal tea, hot packs or ice packs before education.

Table19. Psychological education(Example)

**What is a trauma?**<br>Trauma is the most shocking experience of all the various stressful experience. It is an event and a shock that goes beyond what my body and mind can handle.
**Trauma response and misunderstandings**<br>Therefore, it is very natural and reasonable to feel frightened, and feel beside yourself because of the reoccurring memories of the shock, reluctant to meet people, and feel that your life is completely ruined. Not being able to get over it quickly or cheer up is the same for others. It is not a special and unique reaction that only appears to you when everybody else is coping well.
**Checking the trauma response**<br>After experiencing disaster, various emotional, physical, cognitive, behavioral, and spiritual reactions and symptoms such as ________ will appear. This stress response is a very common adaptive and protective process.
**Understanding the trauma response**<br>First, the body and the mind are over-prepared. It is a reaction that appears to protect oneself. Traumatic memories keep coming back in order for the survivor to be cautious and cope well in the future. And being overly tense can make you anxious, sensitive and irritable.<br>Second, we avoid thinking of traumatic memories or feeling emotions in order to block out the overwhelming emotions that are hard to control and prevent memory loss due to stupefaction. In severe cases, dissociative symptoms may appear such as difficulty in perceiving the surroundings, feeling dazed or lost and as if the real world does not feel real, losing important memories or feeling as if the body and the mind are separate.
**Summary**<br>These symptoms are reactions that anyone can experience immediately after a shock. It is a coping mechanism that does its best to protect the mind and the body from an unbearable shock. It may get better over time, but it is important to seek appropriate help if symptoms are overwhelming or persistent. I hope that ______can understand this fact well, as well as be treated take good care.

• **Oriental Medicine Psychology Education**

- Disaster survivors can better understand their symptoms if they are educated on the relationship between Chiljeong of oriental medicine and related physical symptoms. Chiljeong is a change in the 7 types of emotions: Hui(喜)·Noh(怒)·Wu(憂)·Sa(思)·Bi(悲)·Gong(恐)·Gyeong(驚) [121], and is a detailed

expression of mental activities, and a response and change to external stress. Excessive chiljeong in a stressful situation can affect normal physiological changes and cause diseases[122].

-Responses related to stress are main related to Noh(怒)·Wu(憂)·Sa(思)·Bi(悲)·Gong(恐)·Gyeong(驚), and psychological education regarding the association between chiljeong and the physical symptoms can be provided as follows[123124].

Table 20. Seven modes of emotion and physical symptoms

Noh (怒)	"怒則氣上，怒則氣逆"<br>**"怒傷肝"**	When you think of a time when you were angry, what do you feel in your body?<br>Shortness of breath, fever, stiffness at the back of your neck, and tension in your body?<br>The phrase"Nojeokgisang, Nojeokgiyeok" means that when we are our angry, our body's energy surges upward. So, if you are too angry, symptoms such as fever, shortness of breath, tension, stiff eyes, and headaches may appear. Also, when the anger is overhwleming, it can harm not only the liver but also affect your mood and impair memory.	Overly sensitive enraged in everyday life.<br>When rage is excessive, it helps to understand the symptoms by explaining with various examples that it is directly involved in the manifestation of the physical symptoms.
Wusa (憂思)	"愁則氣沈，思則氣結"<br>**"憂思傷心"**<br>**"思傷脾"**	How do you feel when you see troubled people?<br>Do you have your head down, your chest bent, and you sigh deeply?<br>There is a saying "Wusuja Gipyesaekyibulhaeng" meaning that if you are worried, you cannot function because your energy is blocked. Therefore, you easily let out sighs, have difficulty relieving yourself, and with too much worry and anguish, it may damage your spleen and digestive symptoms could occur such as loss of appetite, dyspepsia and feeling of fullness. Limbs also become languid and difficult to move. It is similar to symptoms of depression.	It can be further explained that if you think too much, your heart may be damaged, resulting in symptoms such as palpitations and insomnia.

Bi (悲)	"悲則氣消." **"悲傷肺"**	How do you feel after you cry? You feel as if something is a little better, right? Dissipating qi by shedding tears can lead to catharsis, but too much can lead to energy being consumed. In traditional oriental medicine, when sadness becomes overwhelming, energy is consumed and excessive sadness damages the lungs.	Describe the state of being depressed and subdued due to the feeling of sadness from daily heartaches and suffering.
Gong (恐) Gyeong (驚)	"恐則氣下,驚則氣亂." **"恐傷腎"**	I almost lost my liver (Korean expression for expressing being very surprised or scared). My stomach churned because I was so surprised. I'm speechless. Have you heard of all these phrases? When you are too surprised, you can't think of anything and are all over the place, right? '恐則氣下,驚則氣亂' represents this.<br>It is said that surprised and fear has a great effect in various aspects, such as disturbing the qi, being surprised easily, insomnia, dizzying and even shaking of the body.	It can be observed as a state of tension and confusion in everyday life. By recognizing that fear can affect the body in various ways because the body and the mind cannot stabilize due to the qi being disturbed by fear, it is possible to understand that trauma reactions are normal and stabilization techniques can be taught.

② Deep Breath Relaxation Techniques

The deep breath relaxation technique can use for not only basic mental health management and improvement, but also for the purpose of improving the mental symptoms of disaster survivors. It is characteristic of disaster survivors to be in shock of past disasters or to worry about an unforeseen disaster in the future. Meditation using breathing can help one stay in the present and take action, freeing yourself from past regrets and future worries. In this regard, the 'Oriental Physician Mental Health Method Guidance Manual', which can be used by oriental physicians, can used by checking through published academic papers[125].

Oriental doctor's mental health instruction manual

This manual was used at the 2020 Covid-19 oriental medicine treatment phone consultation center. Symptoms caused by Covid-19 related stress are classified into persistent symptoms (hypertension, fear, anxiety, fatigue/depression) and

temporary symptoms (insomnia, dyspepsia, pain, anger/irritability), and in addition to psychological education for each symptoms, manuals and videos were provided on the mind-body therapy method so that the symptoms could be controlled.
Basic mind-body therapy includes ‘breathing training, mindful training, and walking meditation’ which is recommended to be performed regularly in daily life, once in the morning and once in the evening. Individual methods such as 1) progressive muscle relaxation and self-training for hypertonia, 2) Susikgwan (a method of counting numbers of inhalation and exhalation to focus the scattered mind) during a state of fear, 3)sitting meditation in a state of anxiety, 4)Self-love meditation and eating meditation for fatigue/depression, 5) body scene for insomnia, 6) eating meditation for dyspepsia, 7) 15-minute meditation with qi for pain, 8) sitting meditation for anger/irritability, are recommended. It is recommended that this individual method be performed whenever symptoms develop or worsen.
Implementation and education are possible through the video provided by the YouTube channel of the Korean Oriental Medicine Association (AKOM-TV) or the YouTube channel of the Department of Oriental Neuropsychiatry at Kyunghee University Hospital in Cangdong.

Figure9. Breathing meditation YouTube link

The hippocampus is responsible for traumatic memories and the amygdala is associated with threat perception. A brain imaging test showed those who meditated regularly had a larger hippocampus[126], and a smaller right amygdala[127]. This suggests that meditators can recover from trauma more quickly. Meditation helps one develop mindfulness. Mindfulness is accepting and feeling something that is happening right now. Mindfulness can help you recognized the fear response of a disaster and help you think about how to deal with it.
Meditation has been used in many disaster situations. In 2018, when the Thai youth soccer team was trapped in cave that was flooded by heavy rain and all were rescued after 17 days, the coach of Ekkapon soccer team, a former monk, told the boys to calm their minds and restore energy to their body through meditation[128]. Disaster response personnel often complain of anxiety, depression and PTSD due to their exposure to disasters. The stress response of the disaster response personnel (soldiers) were reduced

when mindfulness based stress reduction were taught in advance[129]. Transcendental meditation practice on 171 survivors of the 2011 Great East Japan Earthquake significantly reduced stress-related psychological and physical symptoms compared to 326 normal people[130]. In order to find out how mindfulness affects the aftereffects of disasters such as radiation anxiety, health anxiety and psychological stressed caused by the Fukushima Daiichi nuclear power plant accident, 416 people were surveyed 7 years after the accident and the results showed that mindfulness contributed to alleviating psychological stress[131].

According to existing studies, just controlling one's respiration to slow down can have a sedative effect on the autonomic nervous system and central nervous system, an increase in alpha brainwaves and decrease in theta waves on EEG measurements. It was also shown to activate some brain regions related to emotion regulation in an fMRI examination and decreases arousal, anxiety, depression, anger and confusion while improving comfort, relaxation, euphoria and vitality[132]. Additionally, breathing meditation that focuses on breathing may also help maintain performance during acute and chronic stress period by activating reward mechanisms that restore functional cognitive reserves and other physiological systems[133].

Introduce abdominal breathing techniques and breath techniques that can be easily used at disaster sites.
By measuring their own pulse (wrist or neck) along with breathing, the survivor can confirm that they are alive and help form a sense of stability.
In addition to the diaphragmatic breathing method of abdominal breathing, various breathing methods can be used. These include adjusting the length of breath, breathing only through the nose, breathing only through the mouth, nose-to-mouth breathing, and stopping breathing in the middle.
In an actual disaster site, start with the easy breathing. Over time, once you become accustomed to the breathing technique and your attention gets dispersed, switch to a new breathing technique. You can feel considerable amount of stability during the process of practicing new breathing. Therefore, with ways breathing (abdominal breathing), 2~3 stages of breathing techniques can be used together. In addition, various breathing methods can be used according to the difficulty and continuity of breathing.

(1) Abdominal breathing

Taking deep breaths in stressful situations has a calming effect on the sympathetic nervous system. Abdominal breathing can also be performed together while facing each other.

- Sit in a comfortable position.
- Begin breathing through your nose, focusing on the air coming in and out of

your nose (do 5~6 reps).
- Observe and see if the air goes down through your nose, down your throat, lungs and reaches the stomach.

- Let's try to breathe a little deeper using our abdominals. Place one hand on your lower abdomen, feel the inhalation and exhalation, and then proceed without your hand once you adapt to the feeling.
- Imagine that there is a aloon in your stomach and with each inhalation, imagine the air flowing and filling your stomach and lungs. In the same way, when you exhale, the air comes out of the lungs first and then out of the stomach.

- Slowly count three(inhale)/four(exhale) as you breath in and out to slow your breathing rate (do 5~6 reps).

- Practice this process at home for 5-10minute each day to make it a part of your daily routine.

(2) Breathing meditation

- Find a quiet place and sit comfortably.
If you can't find a quiet place,
put your earphones or headphones on,
and it is okay to turn on music with regular and soft melody on a low volume.

- Now, let's start the breathing meditation.
Gently close your eyes.
And take three deep breaths.

- Inhale gently, and exhale slowly,
Inhale gently, and exhale slowly,
Inhale gently, and exhale slowly.

- Now, let's continue breathing comfortably.
Make sure your breathing is regular, smooth, and uninterrupted.
Observe yourself inhaling and exhaling, and look for your own unique rhythm.

- Now, let's watch the breath move in and out.
Without any preconceived notions or judgments,
Just observe the breathing that is taking place in your body

at this moment

- Feel the air coming into your body from the tip of your nose.
Tip of your nose, mouth, throat, and chest.
At this moment, feel your body swelling
with the air that has enter your body.

- feel the air that has entered your body, leave your body again.
Chest, throat, mouth, lips.
At this moment, feel your inflated body shrink again
As the air exists the body.

- Continue to breath,
and continue to observe your breathing.
Even at this moment,
breath is constantly coming in and going out.

- It is natural for different thoughts to come into mind in midst of breathing.
If a distraction comes to mind in the midst,
notice that a distraction has arisen
and focus on your breathing again.

- Without any preconceived notions or judgments,
Just observe the breathing that is taking place in your body
at this moment.

- Continue this inhaling and exhaling for 5 to 10 minutes.

- When you think you have calmed down enough,
Decide first to ‘ I will stop the breathing meditation now’
And end the meditation by slowly opening your eyes.

③ Grounding

When disaster memories are overhwleming, and panic attacks, flashbacks, invasive negative thoughts, dissociation and such occur, grounding allows the disaster survivors to have orientation towards their current external environment and refocus on the therapist-survivor connection, hence allowing them to feel safe and control their inner awakening.

Grounding refers not only to the feet that are touching the ground, but also the ability to touch the mind as well. When grounding, it is helpful to use various senses such as tough, sight, hearing, and smell[134].

• the stabilization technique we are going to learn together is called grounding.

• .Through this practice, we can stay in the here and now.

- First, take a slow, deep breath.
- Let's take a close look at the place you are residing in now. Where are you now? What is the time? Can you describe this place? Slowly turn your head to look around and if there is something that catches your eye, take a moment to look at it and feel the emotions.
- Now, feel the soles of your feet touching the ground, the feeling of support on your back and buttocks, and the feel of the armrests.
- Let's linger in the feeling of your buttocks touching the surface. How does it feel to temporarily linger in that place?
- Put your feet on the floor and place your weight on it. How does the soles of your feet feel on the floor?
- Try touching the armrests or the sides of the chair. How does the part where your arm is touching feel?
- Try and figure out which part of these you are the most comfortable with or feel the most. And try to stay a little longer with that feeling.

④ I-Jeong-Byeon-Gi therapy and Orienting response

Simple activities are performed to cope with uncomfortable affect or intrusive thoughts. Talk to the disaster survivors to help them identify and practice activities that can distract them from their uncomfortable state. For example, listening to music, taking a walk, watching a fun program or video, drawing, or crafting by hand can be utilized.

I-Jeong-Byeon-Gi means to change Jeong (spirit) in order to change one's mood, in other words, changing one's mood is to change one's spirit[135]. I-Jeong-Byeon-Gi therapy is applied in order to provide a reliable, safe and supportive environment to escape from buried emotions and memories and to face painful memories and experiences. I-Jeong-Byeon-Gi therapy includes Jeong-Shin-Jeon-Yi (mental transfer) method using music, art, poetry, dance, plants, people, windows, tea and such as well as Jeong-Seo-Do-Yin (emotion leading) method using yoga, meditation, taichi and so on.

Recently, a technique using an orienting response has been applied in the treatment of trauma[136], in which animals, including humans, face or turn when a new stimulus is given in the direction of the new stimulus. To detect and prevent danger depending where the stimulus is coming, it is closely related to a kind of survival response.

Survivors who have experienced disasters become hyper aroused by even a trivial stimulus or become under-around and become immobile. By using orientation response, they can train to increase their sensory responses so that they can escape from their

buried emotions and memories. Orientation response causes the person to slowly turn their head and look around and to linger at the most eye catching thing while taking the moment to explore the bodily sensations and return to the present moment. Orientation response is a psychological treatment method that combines modern theory with I-Jeyong-Byeon-Gi method which comes from oriental medicine psychotherapy[137].

I-Jeong-Byeon-Gi therapy (= orienting response)

- I-Jeong-Byeon-Gi means to "change (Byeon) mood (Gi) by moving (I) the spirit (Jeong)." It is a way to free yourself from current emotions and painful memories and experience a safe and supportive environment. Let's do it together from now on.

- First, take a slow, deep breath about three times.
At this moment, slowly and move your head up, down, left and right plenty times to look around.
Take a slow look at everything and note three things that catch your eye.
Please tell me more about what they are.

- Is there anything that makes you feel good or comfortable?
If there is, take a moment to observe it and see what you feel inside.
Once you feel that feeling enough, come back slowly.

- (In case they say that they do not feel better or comfortable)
That's okay. Turn your head slowly again and look for something that catches your eyes once again.
And tell me what it is, and what you feel while you're explaining it.

⑤ Hang-ari Therapy(Containment Technique)[138]

This is a method that allows disaster survivors to effectively control invasive memories, images, and uncomfortable physiological responses that the survivors find hard to deal with through visualization in a mindful state. Containment is to teach the ability to control by imagining a storage device such as a jor, box, safe, or a sack, and containing the uncomfortable elements currently experienced by disaster survivors.

Controlling uncomfortable symptoms through visualization can give a sense of relief. This is because when uncomfortable symptoms are sealed, controllability increases and a sense of relief are felt. This therapy is performed after securing a sufficient field of safety. After the uncomfortable symptoms are sealed, you can also deal with the sealed symptoms by strengthening your positive senses and emotion.

- Bring consciousness inward and recall experiences that were painful or unpleasant.
  - When the experience comes to mind, imagine the situation and the context so that we can create a form. Instead of looking at the details, make a shape by defining the painful scenes or emotions as the boundaries.
  - Put the shaped image in the desired container and seal it tightly. And lock it with a key or tie it with a string to prevent it from opening.
  - Place the tightly sealed image wherever you want it (Example: bury it, somewhere in the corner of the room, put it in a safe, or blow it out into space).
  - You can always come back to the place where you placed the container and view it whenever you want. You don't have to look for it and you don't have to look at it. You can handle it at any time when you have the strength.

⑥ Resource Mindfulness (Safety zone visualization technique)[139]

Select a comfortable or a safe space (safe zone) among various resources and encourage them to visualize it. Be aware since in some unstable survivors, anxiety can be aggravated when a safe place cannot be found or when negative memories are connected with a resource location (safe zone). It is better to conduct it briefly at the disaster site, and if anxiety becomes severe or worsen during the implementation, stop immediately and stabilize it through orientation response or grounding. If stabilization is not possible, the patient should be referred to an expert such as an oriental neuropsychiatries or a psychotherapist.

- Sit in a comfortable position and quietly close your eyes.
- And take a slow, deep breath about three times.
- This time, you can just look inside yourself with your mind's eyes.
-. Now, if there is a place that you feel the most comfortable or safe, imagine it as it is.
  - If you do find a place, could please tell me about it slowly?
  (‘If you cannot think of a place that is okay. It can be a peaceful lake, a beach with a wide sandy coast, the blue sky you saw while lying down on a green law, or it can be a forest trail.’ As such, give examples of places that are not related to the disaster site.)
- What is the scenery like? Do you hear anything?
- What kind of sensations do you feel with your body right now?
- Can you feel the air on your face? (Guide them to use the five senses to feel it slowly.)

- And how do you feel about being there now?
  - (Give them enough time to feel the sense of comfort and reassurance that they are feeling right now. And after a little while,)
- Would you like to slowly find a place where you want to store this feeling somewhere in your body?
- (After giving them time) Have you found a place you want to use?
- Then try to save that feeling as it is in that place.
  - Feel the feeling of the saved place once again. This stored feeling can be felt again whenever you need it.
- Now, if you have felt it enough, you can come back with your eyes open.

3) Stabilization Technique Characteristics and Precautions

Method	Characteristics and Adaptability	Precautions
Psychological education	• Change in perception<br>• Feelings of anxiety, shame and guilt are reduced once one realizes that their reactions are natural, predictable and what many people experience	• Need to establish a basic trusting relationship before implementation.<br>• Efficient progress by implementing it in groups.<br>• Use together drawings, props, and other methods (oriental herbal tea, hot packs, and ice packs) to disperse hypersensitivity.
Deep Breathing Relaxation Technique	• Stability of hypertension through autonomic nervous system sedation<br>• Easily applicable	• Use caution in cases of low arousal state, can be used as a group.
Grounding	• Can be used for both hyper and hypo- arousal.<br>• Allows the survivor's condition to enter the resistance zone.<br>• Stimulate the present senses to release thoughts, emotions and memories that have been overwhelmed by the shock.	• Oriental physician use the appropriate senses according to the situation and the condition of the survivor.<br>• Connect to the current supportive environment and relationships (Example: safety factors such as a solid ground or being with people who are helping you)
I-Jeong-Byeon-Gi	• Can be used for both hyper and	• The oriental physician selects

therapy and Orienting response	hypo- arousal.<br>• Activities to divert attention	an appropriate method according to the situation and the condition of the survivor.
Containment method	• When invasive memories, flashbacks are evident.<br>• Acquire the ability to control the uncomfortable memories and emotions.	• Conduct on the premise of sufficient resources and a safe zone.<br>• Reinforce positive bodily sensations and emotions by processing uncomfortable memories.
Resource mi ndfulness	• Can be used as a preparation for exposure therapy.<br>• Can be used at the end of treatment.	• Make sure that the patient is fully relaxed through breathing before implementation.<br>• Be careful as anxiety can be exacerbated when a safe place cannot be found or when negative memories are linked to resource locations.

• When education groups on psychological education or stabilization techniques, the methods implemented by the survivors as a group can create a synergy effect by not only alleviating individual trauma symptoms through social connection, but also creating a healing atmosphere at the disaster site. Creating a healing environment through mourning, healing and solidarity is important for rapid stabilization of traumatic symptoms.

5. Self-care Methods

A large-scale disaster causes serious damage to a large area that is difficult to recover in a short period of time. When a large-scale disaster occurs and lien lines such as electricity, gas and water is destroyed, it is difficult to go to even a small clinic and a surge of refugees makes it hard to navigate. Moreover, because the number of local medical personnel is also insufficient, it is difficult to receive adequate medical support from emergency medical aid centers or evacuation centers at nearby hospitals.

Even if they are not seriously injured, victims of disaster trauma are already psychologically and physically vulnerable, but living in shelters for a long time can cause many health problems too. Refugees experience not only psychological distress, but also infectious diseases such as colds, digestive symptoms, pain, insomnia, deep vein thrombosis, exacerbation of existing chronic diseases and may even be associated with premature death[140141142143144145146]. In the early stages of a disaster, evacuation shelters may be full; hence there could be cases of death from deep vein thrombosis while taking refuge in one's care. During the 2016 Kumamoto earthquake, evacuation-related deaths were four time higher than direct deaths such as death by house collapses.[147]. Especially the elderlies, children, women, the disabled, low-income groups and vulnerable group with a history of pre-existing mental disorders may suffer even more.

In summary, 1) it is difficult to receive proper medical support in a chaotic situation where large number of survivors occur in the early stages of a disaster, 2) various symptoms occur due to long-term shelter life, 3) it is difficult to provide continuous and sufficient medical support, 4) existing medical or psychological support requires a lot of money, time and manpower, hence it is difficult to provide it continuously for a long time. Therefore, the self-management method that allows the victims to manage their own health can be effectively used.

The self-care methods have the following advantages: 1) self-efficacy and resilience can be improved if the victim actively participate in controlling their own discomfort without relying solely on external help, 2) Self-care methods can be used together with existing treatments to produce synergistic effects or alleviate the side effects of existing treatments, 3) If the symptoms are managed by the victims themselves, the medical expanses can be reduced, minimizing unnecessary hospital visits and medical use.

First, we introduce **1) Remedy for each symptom** that can be applied at the disaster

sites, **2) acupressure**, **3) Doyen exercise**, and 4) specific methods such as **walking meditation**.

1) Remedy for each symptom

① Hypertension/Anxiety

• Understanding the symptoms: Tension is a natural response to coping with the stress that occurs after the shock of a disaster. When you feel threatened, you become hyper aroused and your muscles tense. These tensions are to prepare for shock that may occur again in the future. Continuous an excessive tension can stimulate the autonomic nervous system response, causing various symptoms and avoidance behaviors such as palpitation, insomnia, dyspepsia, and headache. It is necessary to understand that this tension is a protective response, and that excessive tension will only lower the ability to react and cope.

• Breathing method: Breathing becomes a link between the body and the mind. Making a long exhale and stabilizing the sympathetic nervous system is helpful. Exhale slowly and gently, and when the anxiety has settled, return to natural breathing. For active relaxation training, progressive muscle relaxation method and autonomous training are additionally applied.

• Thought control: any concerns. It is necessary to confirm whether my concerns are

justified through conversation with the people around me or write them down my selves. When conversations clarify the thoughts that are making you nervous, it becomes easier to control the anxiety response.

• Visualization: Breath comfortably and think of a safe and comfortable place.

• Focus on bodily sensations: It is good to focus on the bodily sensations if it is difficult to control excessive worry and anxiety. Activities such as like walking, massage and stretching, playing catch, listening to music, singing, working on a coloring book, taking a bath or lower-body bath may prove to be helpful. It is more helpful to stabilize the autonomic nervous system if family members or close acquaintances are active together.

• Mindfulness: Rather than trying to force to change your thoughts, it is better to let go of your thoughts and observe and accept the tensions and worries as they are. For this, breathing relaxation or mindfulness medication can be used.

② Fear/Emotional Paralysis

• Understanding the symptoms: Fear is a state of panic caused by extreme tension and anxiety. After the shock of a disaster, our body and minds become frozen when we can't handle our excessive stress response. In addition to the hyperactivity of sympathetic nervous system, there is dizziness, memory loss, emotional paralysis and dissociative symptoms may appear. If extreme tension is anxiety about the future, fear is a reenactment of the past shock. But understand that even these fear response is to protect oneself and block the threat.

• Breathing method: Cope with anxiety attacks through the usual breathing relaxation method.

• Grounding method: Look closely at three objects seen around you, lean on a comfortable chair and feel the parts of your body that are in contact, or if you are standing, confirm the feeling of your feet on the ground.

• Exposure practice(Gyeongjapyeongji-therapy): Understand that excessive avoidance only aggravates anxiety. Observe the avoidance situation and gradually practice being exposed to them. Gyeongjaepyeongji method (systematic desensitization therapy) can be applied. First, practice in a relaxed and stable state to sense of stability, and based on this, gradual exposure to stimuli that induces a trauma response is attempted. However, it is essential to form a therapeutic relationship beforehand and should not be hasty about exposure. If the survivor's condition worsens, do not use this method and refer to a specialist.

• Refrain from using media: Be careful of too much exposure to media as reports of disaster too often can exacerbate the fear.

• Expert referral: If dissociative symptoms such as memory impairment or confusion occurs due to do excessive blocking, or if there is a risk of self-harm or other forms of harm, refer to an expert.

③ Depression/Grief

• Understanding the symptoms: If excessive tension is not released, the energy of the body and mind is consumed, resulting and fatigue and exhaustion. Feelings of guilt and self-blame for not coping well with the disaster, and depression from when disaster damage is not well-recovered and traumatic symptoms persist may occur. The rhythm of daily life is broken by the shock of the disaster which aggravates the feelings of depression.

• Suicide risk determination: Foremost, pay attention to the risk of suicide. Check the risk of suicide through periodic evaluation and observation and refer to a specialist if necessary. If there is a history of such depression in the past, be especially careful.

• Creating daily life rhythm: A new regular life rhythm must be created because normal daily life activities cannot be performed due to the impact of the disaster and life in an evacuation shelter may last for a long time. Set and practice regular wake-up time, meal time, and activity and sleep time.

• Increasing activity level: It is necessary to move the body and increase the amount of activity. Continue activities in a limited space through walking meditation and stretching or Doyen exercises.

• Sleep management:. Decreased daily life rhythm and persistent anxiety is often accompanied my insomnia. Active sleep management is required.

• Changing one’s mind: Positive thoughts and mind are needed in order to overcome depression. Seeing the meaning of the disaster, planning for the future rather than the past, and the compassion to accept the change in who you are and wanting others to be happy can bring you bright energy.

• Social relationships: Participate in gatherings and interactions with survivors of the same disaster. Social activity activates the autonomic nervous system (ventral vagus nerve) to stimulate the social engagement system and change the mood. Activities such as listening to and helping other survivors at self-help groups can help overcome depression.

• Prevention of obsession: Being caught in a vicious cycle of negative thoughts, energy is consumed and you cannot get out of depression. Help to let fo preoccupation with perfectionism or over-preparation. Encourage them to start with one very small action rather than too many thoughts.

• Grieving problem: If there is a problem of mourning or bereavement due to the death

of a family member or acquaintance, specific help an counseling are needed.

④ Anger/Hypersensitivity

• Understanding the symptom: There are many various manifestations of anger, irritability, hypersensitivity, and even violent behaviors. Physical symptoms such as burning sensation, headache, chest palpitation and tightness can appear and interpersonal problems may occur if they spread to people around them. If there is a person responsible for a disaster, resentment is a natural response. First, understand that anger is a natural reaction to the situation. If your anger is embarrassing and you deny it and repress it, it can exacerbate into another problem. Recognizing and accepting emotions that are difficult to process is necessary.

• Expression of anger: However, the expression of anger can cause various problems such as self-harm, harming others, social relationship problems and behavioral problems and can cause even further anger. Understand that anger does not help solve any problems.

• Breathing method[148]: If inhalation length is set to 3, the exhalation length is set to 5 and breath. As you inhale, count to one, two, and three. Count to five as you exhale. When you are suddenly angry, inhale and exhale like this 5 times and it will take about 1 minute. Repeat this three times and hold this pattern of deep breathing about 3 minutes. As time passes, the excitation of the sympathetic nervous system subsides and normal breathing is maintained. If the usual breathing method is maintained for around 10 minutes, the body can get out of its state of anger and feel a sense of stability. If you practice such breathing techniques on a regular basis, you can effectively deal with anger in the moment.

• Other life management[149]: Consider food (light food, bitter vegetables), tea (pettermint, chrysanthemum, goji-berry, tangerine peel tea), walking, writing, drawing, talking with people around you (expressing emotions and restoring interpersonal relationships).

⑤ Insomnia

• Understanding the symptom: It is a natural response to inability to fall asleep due to extreme tension, anxiety and depression after the initial shock of the disaster. It is caused by the disruption of the rhythm of daily life. It is important to increase the amount of activity and create a new rhythm. Appropriately evaluate and observe whether it persist for more than 1 month.

• Adequate arousal: Get enough sunlight and be active during the day to maintain proper arousal. It is helpful to get up at a set time according to the rhythm before the disaster and walk lightly for at least 30minutes by a window or outdoors in the sun. Plan

and practice regular activities to increase the amount of activity in a limited daily life.

• Restrict naps: If you can't sleep at night and feel tired, there many instances where you lie down during the day to rest or take a nap to supplement your sleep. However, this chances the day and night which lowers the effectiveness of circadian rhythm, so limit or minimize nap times.

• Relaxation method: Even when you are tired, you cannot fall asleep due to the perpetuating arousal and tension. Carry out relaxation techniques before going to bed. Create your own relaxation method such as breathing, bathing, light walks, listening to music, writing a diary, talking with your family and practice it.

• Sleep management:

- If you try too hard to sleep, your body will wake up and you will not be able to fall asleep. Paradoxical methods such as sleep restriction can help. Rather than worrying about not getting enough sleep, if you don't sleep today, it is better to make up your mind that you can sleep better tomorrow.
- If you can't fall asleep for more than 30minutes, get out of bed an do other activities. Be prepared for comfortable activities that you do during this time. Then try to sleep again when sleepiness returns.
- Even if you haven't slept at all, it is important to wake up in time for the morning to create a circadian rhythm.
- Proper exercise help you to sleep.

• Fatigue management: You can properly manage fatigue during the day through herbal medicine and acupuncture.

• Acupressure: Acupressure (ear acupuncture (sinmun)) and general acupoints (Shemen, naegwan, sameumgyo, and yongcheon) can help with insomnia and be utilized as a self-care method.

• Sleeping pills management: When taking sleeping pills, instruct them of the correct ways to take them and be careful of dependency or abuse of sleeping pills.

• Prevent dependence on sleeping pills and actively manage sleep through comprehensive sleep management including herbal medicine, acupuncture, sleep education, cognitive behavioral therapy and relaxation methods.

⑥ Loss of appetite/ dyspepsia

• Understanding the symptom: Depression reduces appetite and anxiety tension can cause dyspepsia. If the loss of appetite or dyspepsia persist, it can weaken immunity and depression and fatigue can exacerbate.

• Eating slowly: Chew your food slow and long and take time to taste it. Massage every corner of the gums with your tongue as if your brushing your teeth to stimulate salivary gland secretion.

• Visualization: Find delicious food that you normally like, and imagine the taste and the memory of the taste. Search for memories of a delicious meal with your family, friends, and close friends and try to recall them as specifically as possible

• Circadian rhythm control Appetite and gastrointestinal activity are one of the circadian rhythms. Promote appetite through regular meals and increased activity.

• Post-meal management: Take a light walk after eating to help gastrointestinal react and promote digestion.

• Acupressure: Educate to improve digestive function through appropriate acupressure (Hapgok, naegwan, etc.)

• Relaxation method: Excessive tension and anxiety inhibit gastrointestinal motility. Make your mind at east through relaxation.

• Eating disorder: If accompanied by excessive eating disorders (anorexia/bulimia), consider evaluation and referral to a specialist. You can try eating meditation.

⑦ Fatigue/Burn out

• Understanding the symptom: This can occur as a result of persistent depression or extreme tension. Disaster shocks are not easily solved and a long-term stay at an evacuation center can lead to fatigue. It is associated with the disruption of daily life rhythm. Try to have a regular wake-up time, eating, activity, sleep. Depression and insomnia leads to fatigue and fatigue can exacerbate depression and insomnia. Therefore, management of depression and sleep should be integrated.

• Increasing activity level: Increase the amount of activity. Increase the amount of activity in daily life such as light stretching and exercise, walking, walking meditation and light housework. Increased level of activity can also help improve depression and insomnia.

• Acupressure: Educate to improve fatigue through appropriate acupressure (joksamni, yongcheon, etc.)

• Social activities: Loss of activity due to fatigue can lead to isolation and exacerbate depression. Trying social activities through self-help groups can help improve fatigue and depression.

⑧ Headaches/Dizziness

• Understanding the symptoms: Headaches and dizziness may occur after disaster as it is related to muscle stiffness in the neck and shoulder due to extension, depression, reduced activity, anger and irritability. The dizziness that blacks your eyes out is often because of weak and low blood pressure and circulation to the head, so sufficient

nutrition and exercise are necessary.

• Correct posture: Posture is important. If you stay in one posture for too long, your neck and shoulders muscles are especially prone to tension. It is necessary to be careful to not overuse computers or smartphones and to take a correct posture.

• Stress management: Anxiety, tension and depression can tense the body and is associated with headaches and dizziness or can exacerbate these problems. Psychological stress should be properly managed through breathing techniques, walking, and simple recreational activities.

• Acupressure and stretching: Acupressure on the acupressure points around the neck (fetch, fungus, Cheonju, etc.) to release the stagnant qi and muscle, and your head will become clear due to improved blood circulation. In particular, the tension in the trapezius and sternocleidomastoid muscles is closely related. Stretching and acupuncture of these muscles can help.

• Steaming method: Lying down with a hot compress on the nape of the neck or the back of the head helps to relieve tension in the neck and shoulders.

• Coping with anticipatory anxiety: If fainting or panic attacks are accompanied with severe dizziness, anticipator anxiety occurs and avoidance of factors that cause dizziness will take place. Since such activity restriction can exacerbate anxiety, tension and depression, use relaxation techniques such as breathing to deal with anticipatory anxiety and tension.

• Referral to clinic: If headaches and dizziness persists all day, or it other neurological symptoms such as intracranial hypertension, double vision, and weakness in lower and upper extremities appear, consider referring to a specialist from a detailed brain examination.

⑨ Pain

• Understanding of symptom: Pain is a common physical symptom that accompanies PTSD. Muscles overstraining causes pain. It is necessary to relieve this overseen through breathing techniques. If the daily life rhythm is disrupted and the amount of other activities are lowered, depression is induced and sensitivity to pain increases. It is necessary to improve the amount of activity for active pain management.

• Dispersion of attention: If you pay attention to the area with pain, the pain will worsen. You need to practice diverting your attention elsewhere. Change of attention is required such as housework, walking, talking to people or listening to music.

• Mindfulness: Pain is a sense to protect you from threats and physical trauma. Try to chance the way we perceive pain as threatening and negative. Pain requires an attempt to feel and look at it as it is, and an attempt to observe how it changes over time.

Mindfulness meditation can help.

• Acupressure: Pain can actively be managed with acupressure on the affected area.

(2) Acupressure

1) Background

Acupressure is a treatment and management method of pressing and stimulating with a finger or a tool on a specific area (acupoint) of the body surface to improve symptoms and maintain health. Acupoints are located on meridian pathways, and meridians (12gyeongmaek and 15nakmaek) are a huge network that connects all parts of the body from the intestines to the muscle and the skin. Acupuncture points are physiological reaction points of qi (life energy) that operate meridians, pathological/diagnostic reaction points related disease occurrence and therapeutic stimulation points for acupressure. When an effective stimulus is given to acupoints, a biological response appears, which can prevent and treat diseases.

Acupressure is simple so that anyone can do it easily and it has the advantage of being able to be done alone or to a family member or a colleague without any restriction on place and time. Since acupressure is a non-pharmaceutical and non-invasive method, it can be safely applied without pain, and the patient can control his or her symptoms and improve health, thereby improving their quality of life. In the early stages of the disaster, the infrastructure was destroyed and tap water not supplied enough to the shelters, so the victims could not take a bath or wash their hands properly. Acupuncture requires additional resources such as clean water, antiseptic solution and alcohol swabs for disinfection as appropriate disinfection reduces the risk of infection, but because acupressure or massage is a noninvasive method, so it does not require resources for separate disinfection and because there is no risk of infection, it can be used safely and easily[150]. Moreover, it can be applied without any burden to survivors or children who have a fear of acupuncture.

Post-disaster victims complain of various symptoms such as pain in the shoulders, back and the joints, general fatigue, swelling and numbness, loss of appetite and constipation due to the inconvenient shelter life in which it is difficult to secure a private life as it is very limited in space as well as psychological shock. Since the meridians and acupoints are distributed through the body and are connected to the five organs, you can deal with the symptoms by selecting the meridians and acupuncture points relied to each symptom and applying acupressure.

Acupressure and ear acupressure have even applied not only to psychiatric symptoms such as anxiety and insomnia but various physical symptoms and disease such as pain, respiratory, digestive and skin diseases, female diseases, and postoperative side effects with many evidences showing their effectiveness. In a systematic literature review

conducted in 2015, self-acupressure had a significant effect on allergic diseases, nausea and vomiting in cancer patients, respiratory diseases, menstrual pain, stress and fatigue in healthy people, and insomnia[151]. Improvements in the quality of life for the elderlies can also be confirmed, such as anxiety[152], insomnia[153 154 155 156], pain[157 158], neck pain(difficulty in moving)[159], labor pain[160], post-operative pain[161], pain accompanying anxiety[162], menstrual pain[163], premenstrual syndrome[164], chemotherapy induced nausea/vomiting[165], obesity[166] myopia[167], post-operative intestinal obstruction[168], constipation[169170171], itchiness[172], allergic respiratory symptoms[173], cognitive function, behavioral and psychological symptoms of dementia[174], stress of dementia caregivers[175], insomnia·depressoin in the elderly,[176].

Although there are no studies using acupressure directly on PTSD victims or the victims of the Great East Japan Earthquake, there are reports that suggest acupuncture and massage were used in combination to relieve pain in long-term victims of the Great East Japan Earthquake.[177178]. In addition, Emotional Freedom Technique (EFT), which is used for PTSD and various psychological and physical symptoms, combines a process similar to acupressure by tapping specific acupuncture points along with cognitive and exposure therapy[179].

Acupressure is a generally safe self-care method. However, since acupressure treatment is not a treatment that fundamentally solves the cause, it cannot solve all the symptoms. Therefore, if symptoms do not improve even if acupressure treatment is continued, it is necessary to consult with an oriental physician.

2) Points to consider

• It is easy and simple, so anyone can carry it out, but if the location of the acupressure point is accurate, a better effect may appear. The users of this manual can find the exact location by referring to the standard acupuncture point DB (figure)[180] provided by the Korean Medicine Convergence Research Information Center and the oriental physician in charge should properly educate and give feedback on the acupressure.

• Before the procedure, it is desirable to disinfect or wash hands using disinfectant solution and clean water for a safe and hygienic procedure.

• Place the fingertip on the acupoint, and gently press then release. The stimulation time is about 3 seconds. When you press on the acupoint, you might feel mild tenderness, hence press around to find the tenderness points.

• The thumb which is easy to apply force to, is used the most. Use your index or middle finger to press lightly. Lightly press areas sensitive to stimulation (e.g. Face) using your index or middle finger. When stimulating a large area, use several fingers at once and lightly press both fingers on the abdomen.

• The number of pressure per acupoint is about 5 to 10 times, and the duration is about 3

minutes. The number of times of acupressure per day is 1 to 5 times, depending on the situation and individual condition[181]. If necessary, consult with an oriental physician about the appropriate number and time of stimulation.

• When acupressure is applied to acupoint, a cold, tingling, heavy and swollen feeling (sanmajunchang) may appear. This is called deukgi and serves as a criterion for judging the proper and effective stimulus.

• When stimulating a wide range, various methods such as rubbing or stroking the acupuncture points and muscles with your hands (Gyeongchal-method), holding and kneading with pressure (Yunal-method) or tapping with your fists (Gota-method)(**Figure9**) can be used. These methods are used in Chuna therapy as well. Compared to acupressure, it stimulates a wider range, so the strength of the force is weak. Rubbing the point with the back of our hand can give you a stronger stimulus. Massage improves blood circulation and is effective when hands and feet are swollen cold, or overly tight.

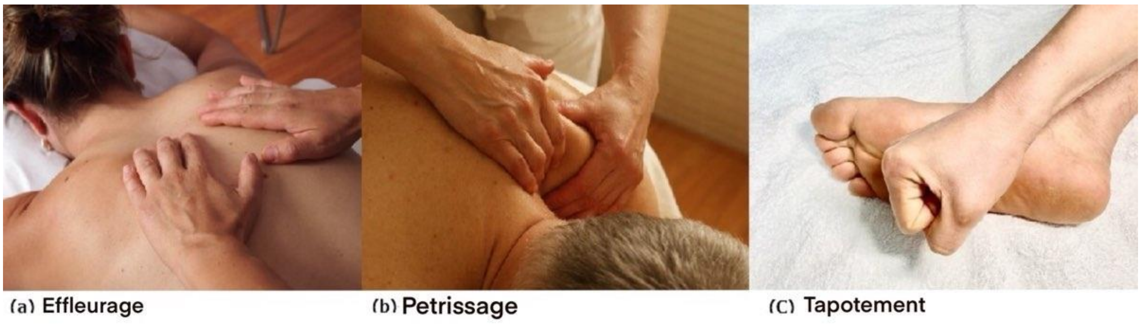

Figure9. Massage method

• There are times when the stimulation cannot continue which are cases in which the stimulation is stronger than a finger, stimulation of a hard to reach area or a wide read, or when the strength of the finger is weak. In this case, you can use a variety of everyday tools other than your fingers. Stimulate the head by tying several hairpins, ballpoint pens or toothpicks, use clothespins to give strong stimulation of fingers or toes, roll a golf ball to stimulate the soles of the feet, stroke the shoulder with a wooden bat, or stroke the hair with a comb. Hot packs, towels or hair dryers can easily give heat stimulation to a large area. Since these tools are readily available nearby, they are highly useful in situation where medical support is limited in the early stages of a disaster. Therefore, if disaster victims are provided with various acupressure tools in addition to daily necessities, it will be helpful for the victims to self-manage their health.

• Be careful of acupressure when exercising vigorously, drinking alcohol, bathing, right after eating, high fever, bleeding or excessively high blood pressure. Excessive stimulation of acupuncture points during pregnancy may lead to miscarriage or

premature birth, so it should be performed with caution after consulting with an oriental physician.

Figure 10. Korean Medicine Convergence Research Information Center Homepage Link (Standard Acupressure DB)

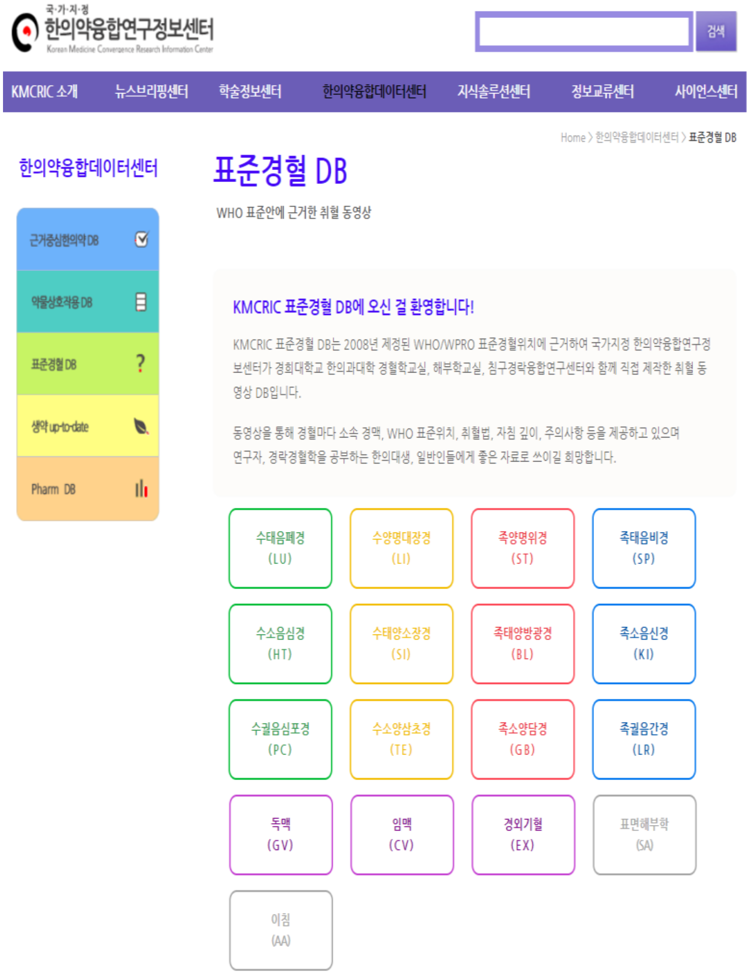

3) Acupint for each symptoms[182]

① Basic 11 Acupoints and Considerations

• Acupoint that stabilizes the body and mind: Sinmun, naegwan, yintang, sameumgyo, yongcheon

• Acupoint that circulates Qi and blood: hapgok, pungji, gyeonjeong, baekhoe, zhishi, joksamni

• Thermal stimulation: Hot pack or a hot towel can be used and if there is electricity, hair dryer can be used.

② Psychological symptoms

• Hyper arousal/Anxiety: sinmun, naegwan

• Insomnia: sinmun, yongcheon, anmyeon, sameumgyo

• Depression: baekhoe, high, taechung, joksamni

• Poor concentration/Forgetfulness: baekhoe, pungji, eunbaek

• Anger/Hypersensitivity: taechung, sameumgyo

③ Physical symptoms

• Musculoskeletal pain

Excessive tension and uncomfortable shelter life caused by disaster shocks can stiffen the muscles and cause various pains. PTSD is often accompanied by pain.

- Neck, Shoulder pain: jianzhen, pungji, houxi, Gyeonghang and gyeongjeom Of hand acupuncture(手鍼)
- Back pain: zhishi, zhongfeng, jinmen, 1,2 yaoshu of hand acunpuncture(手鍼)
- Knee pain: xiyangguan, joksamni, yangneungcheon
- Muscle cramps: joksamni, yangneungcheon

• Headache

The tension caused by the excitement of the sympathetic nervous system and the chaotic situation at the beginning of the disaster can cause headaches.

Hegu, taeyang, pungji: stimulate strongly

- proximal interphalangeal joint of the finger: Jeondu point(thumb), Dujeong point(middle finger), Pyeondu point(ring finger) and Hudu point(little finger) of hand acupuncture(手鍼).

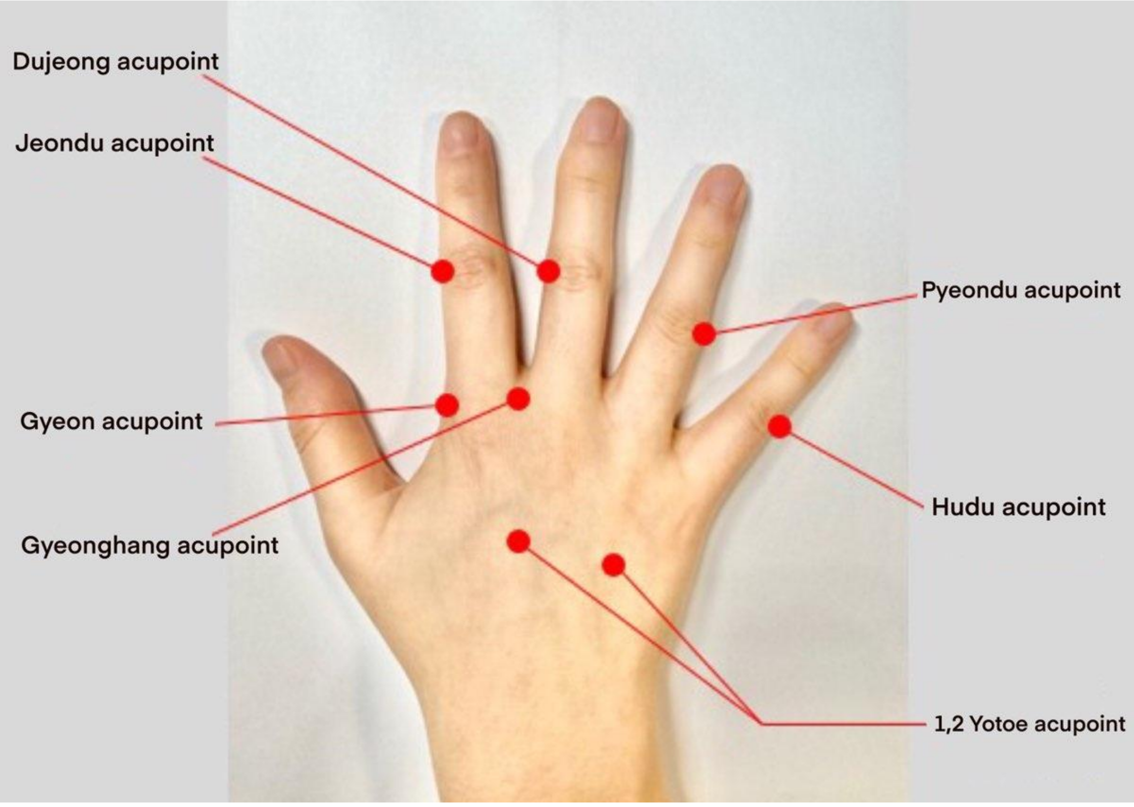

Figure 11. Hand acupunture acupoints

• Dizziness

Dizziness can often occur when experiencing natural disaster such as earthquakes that shakes the ground.

- zuqiaoyin, taeyang, pungji

• Eye fatigue/loss of sight

Disaster shocks and chaotic environment can impair sensory control, leading to narrowed or blurred vision.

- zanzhu, Eoyo, jignming, chengqi
- Jingming: Using the thumb and index finger, grasp the left and right acupoints and press them simultaneously as if wrapping them.

• Cold symptoms

Living in a shelter with a high density of people in a narrow space and poor sanitary conditions is prone to various upper respiratory infections. Especailly in winter when the shelters are very dry because they use hot air fans rather than a boiler for heating.

- Runny nose, sneezes: yingxiang, taiyuan(thermal stimulation), yongcheon
- Stuffy nose: yingxiang, daechu, yamen
- Chills, fever: fengmen, cheonju, pungji(thermal stimulation)

- Coughs: tiantu, kongzui
- Chest palpitation/shortness of breath: naegwan, jeongjung

• Loss of appetite, stomach pain
- Jungwan, joksamni, Shenmue, cheonchu (thermal stimulation)

• Defecation

Constipation can occur due to changes in lifestyle of living in the shelter, imbalance in eating habits, decreased intake, lack of exercise and stress.

- Diarrhea, stomach ache: joksamni / Shenmue, cheonchu (thermal stimulation)
- constipation: eunbaek, yetae, dadun[183]

• Fatigue/lethargy

Uncomfortable shelter life can cause fatigue and drowsiness due to reduced intake, lack of exercise, stress and depression.

- joksamni, eunbaek, yetae, yongcheon(tap 50~100 times with your fist.)

• (Women) menstrual cramps: sameumgyo

• (Children) Waking up frequently at night and cry, very irritable, a lot of night sweats: Shenzhen, myeongmun(gently stroke the entire back area)

Table22.Acupoint for each symptom

<table>
<tr><th colspan="3">Symptom</th><th>Acupoint</th></tr>
<tr><td rowspan="5">Psychologi cal sympt oms</td><td colspan="2">Anxious/nervousness</td><td>Sinmun, naegwan</td></tr>
<tr><td colspan="2">Insomnia</td><td>sinmun, yongcheon, anmyeon, sameumgy o</td></tr>
<tr><td colspan="2">Depression</td><td>baekhoe, high, joksamni</td></tr>
<tr><td colspan="2">Poor concentration/forg etfulness</td><td>baekhoe, pungji, eunbaek</td></tr>
<tr><td colspan="2">Anger/hypersensitivity</td><td>taechung, sameumgyo</td></tr>
<tr><td rowspan="3">Physical s ymptoms</td><td rowspan="3">Musculo skeletal Pain</td><td>Neck, shoul ders</td><td>jianzhen, pungji, houxi, 1 and 2 gyeonghang point of hand acupuncture</td></tr>
<tr><td>Back</td><td>zhishi, zhongfeng, jinmen, 1,2 yaoshu of hand acunpuncture</td></tr>
<tr><td>Knee</td><td>xiyangguan, joksamni, yangneungcheon</td></tr>
</table>

		Muscle cramps	joksamni, yangneungcheon / proximal interphalangeal joint of the finger: Jeondu point(thumb), Dujeong point(middle finger), Pyeondu point(ring finger) and Hudu point(little finger) of hand acupuncture(手鍼).
	Headaches		Hegu, taeyang, pungji:
	Dizziness		zuqiaoyin, taeyang, pungji
	Eye fatigue/loss of sight		zanzhu, Eoyo, jignming, chengqi
	Cold symptoms	Runny nose, sneezes	yingxiang, Taiyuan, yongcheon
		Stuffy nose	yingxiang, daechu, yamen
		chills, fever	fengmen, cheonju, pungji
		Cough	tiantu, kongzui
	Chest palpitations/shortness of breath		naegwan, jeongjung
	Loss of appetite, stomach ache		jungwan, joksamni, Shenmue, cheonchu
	Defecation	Diarrhea, stomach ache	joksamni / Shenmue, cheonchu
		Constipation	eunbaek, yetae, dadun
	Fatigue/lassitude		joksamni, eunbaek, yetae, yongcheon
	(women) menstrual cramps		sameumgyo
	(children) wakes up frequently at night and cry, irritable and night sweats		Shenzhen, myeongmun

-

(3) Doyen exercise

The origin of Doyen exercise-method, a traditional Korean medicine treatment method, can be found in “Doin Anglo” recorded in Yeongchu•Byeongjeopyeon. Doyen exercise is a therapeutic exercise used as a treatment for diseases and recuperation method that actively moves the body along with breathing[184]. Doyen implements both breathing and movement at the same time to promote qi and blood circulation in the body, excreting morale from the body[185], regulate functions of the organs, stabilize the mind, communicate meridians and strengthen the muscles and the bones[186].

Originally, the Dao(導) in Doyen means to bring in the Qi of the atmosphere through breathing and yin(引), meaning to stretch, which means that the usage of human body’s flexibility. In other words, the Doyen uses the energy drawn into the human body to activate the functions of each part of the human body hence it differs from western exercise therapy in that it includes mental exercise rather than being a simple physical exercise[187]. Studies have been published that Doyen Qigong gymnastics for adult women were effective for their shoulder pain[188] and degenerative knee arthritis[189]. Other studies have also reported that it was effective in improving the pain, function, and quality of life[190] in patients with low back pain.

Doyen exercise moves the spin through the movement of the extremities and shakes the internal organs through the movements of the spin, thereby correcting the structure of the musculoskeletal system and promoting smooth blood circulation in internal organs[191,192,193]. In particular, since it does not occupy much space, it is highly useful in situations where activities are restricted due to long-term shelter life at the disaster site and physical pain and functional deterioration occur. It can be used as a form of self-care method or as a form of group therapy to help improve pain and faction of disaster survivors and improve their quality of life.

1) Donguibogam [Massage Doyen]

• Either educates the group to follow along to the video or use videos to encourage individuals to perform consistently in their daily lives.

• Emphasis is mainly placed on action that relieve stress, relax the whole body and help with posture.

• Use the suggested YouTube video or have an oriental physician familiarize you with the movement and demonstrate it yourself.

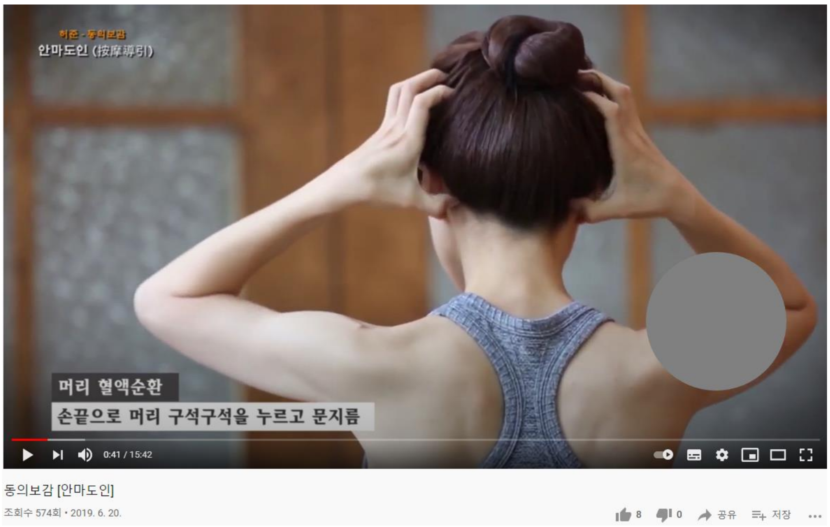

Figure12. Korean Health Promotion Institute YouTube Link(Donguibogam Massage Doyen)

Table23. Donguibogam Massage Guidelines

Number	Location	Method	Effect
1	Head	Press and rub every corner of the head with the fingertips.	Blood circulation of the head
2	Forehead	With the fingertips, sweep down from the root of the hair 10 times, rub the temples.	Sheen of the face, kidney
3	Eye	Rub the palms together to make them heat up, then rub the eyes 10 times, roll the eyes 5 times.	Clear eyesight, liver
4	Nose	Rub the left and right side of the nose up and down with the middle finger 1 times, then breath 5 times	Strengthening the nose and plunges
5	Ear	With the right hand, pull the left ear over the head 5 times and vice versa 5 times. Rub the ears 10 times with both hands.	Blindness, hearing loss prevention, strengthening the kidneys. Vagus nerve stimulation.
6	Teeth, Tongue	Clack the teeth 5 times, and rub the gums with the fingertips. Turn the tongue around in the mouth to stimulate saliva and swallow.	Strengthening the gums, spleen, and stomach
7	Neck	Pull the left neck muscles (sternocleidomastoid, quadriceps) 10 times with the right and vice versa 10 times.	Blood circulation of the neck
8	Back of the neck	Raise the head and turn it left and right 10 times with the hand placed on the waist.	Relaxation of back of the neck
9	Shoulder	Strongly move the shoulders up and down 10 times.	Shoulder tension relaxation, scapula, trapezius
10	Chest	Use the right hand like a rake and sweep from the outside to the inside from the collarbone to the ribs 10 times and vice versa 10 times.	Chest, cardiopulmonary circulation
11	Sternum	Bring the hands together and sweep from the top to the bottom 10 times (while breathing deeply through the nose).	Relieving stress

12	Abdominal	Put the hands together and sweep in a clockwise circle 10 times (in the direction of the intestine	Visceral circulation, digestion
13	Waist	With hands on waist and feet shoulder apart, move the waist forward and backward 5 times	Waist
14	Hips	Warmly rub the back and sacrum with both hands 10 times.	Strengthening the sacrum
15	Kegel exercises	Squeeze the anal muscles 5 times.	Reproductive organs
16	Twisting the waist	Interlace both hands and rotate the waist left and right 10 times.	Waist
17	Running while breathing through nose	Ruin in place while breathing through the nose for 1 minute, move the arms backwards with force.	Shoulder, full body relaxation
18	Ending breath	Breathe deeply 10 times.	Full body relaxation

2) Doyen exercise movements[194]

Doyen(導引法), or kyoyinn(撟引), is a method of stabilizing the mind and body and strengthening the body by stimulating the brain and the whole body to promote the circulation of qi and blood. Several methods are used, such as concentration, swallowing, breath control, using force, exercising, touching and rubbing method.

Doyen method is characterized by focusing on the breath and intention rather than the body movements and can help prevent diseases by promoting blood circulation, strengthening muscles and bones, relieving fatigue, and strengthening the mind and body.

① Whole body stimulation: expand shoulder rotation range, promotes blood circulation in the upper body and stimulates the brain.

- Head clapping : Raise your fingertips and lightly tap your head[195]
- Shoulder clapping : Tap the shoulder with both hands.
- Hip clapping : Tap the buttocks with both hands.
- Vertical clapping : Raise the hands vertically and clap.

② Hand exercise - clapping : stimulates peripheral nerve, promote blood circulation

- Fist claps 4 times + vertical claps 4 times
- Fingertip claps 4 times + vertical claps 4 times
- Palm claps 4회 + vertical claps 4 times
- Wrist claps 4회 + vertical claps 4 times

③Hand exercise - gripping : promotes movement capabilities

- Vertical clap : Clap strongly with both hands.
- Horizontal clap : Clap the hands while laying it flat.
- Horizontal grip : Hold your other hand tightly while keeping the hands horizontal.
- Place the hands facing each other and clasp as hard as you can.

④ Arm exercise – both arms : promotes upper body blood circulation, promotes movement capabilities

- Push both arms forward[196]
- Push up with both arms[197]
- Push both arms to the side
- Push the arms while it is crossed

⑤Arm exercise – single arm: promotes upper body blood circulation, promotes movement capabilities

- Pushing one arm at a time: starting with the right hand, push each arm forward, up, sideways and diagonally and repeat.

⑥ Making qi: activation of the occipital lobe, parietal lobe and frontal lobe

- Gathering qi

① Place one hand above and below the chest and curve your fingers inward.

② Rotate the left hand to face up.

③ When the left hand is on the upper side, slowly rotate it so that the right hand faces upward again.

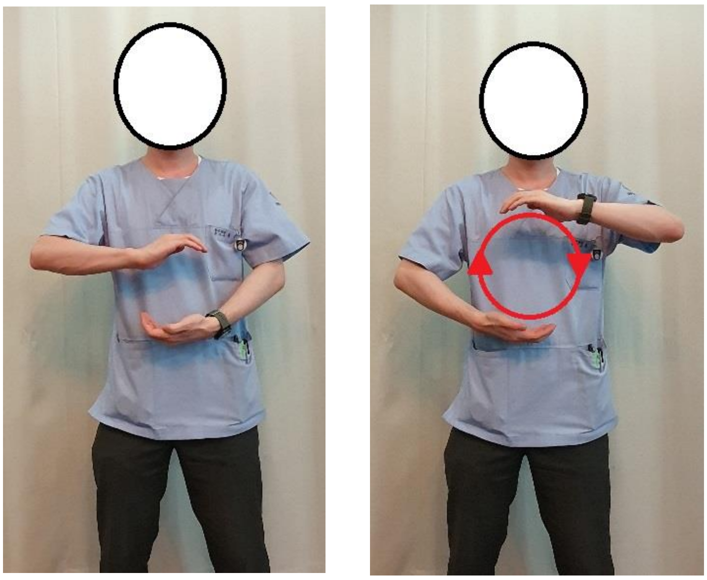

①-② ③

Figure13. Gathering energy

- Raising the energy

Extend both hands to the size of your body and rotate them slowly in the same way.

- Increase energy

Extend both arms up and down

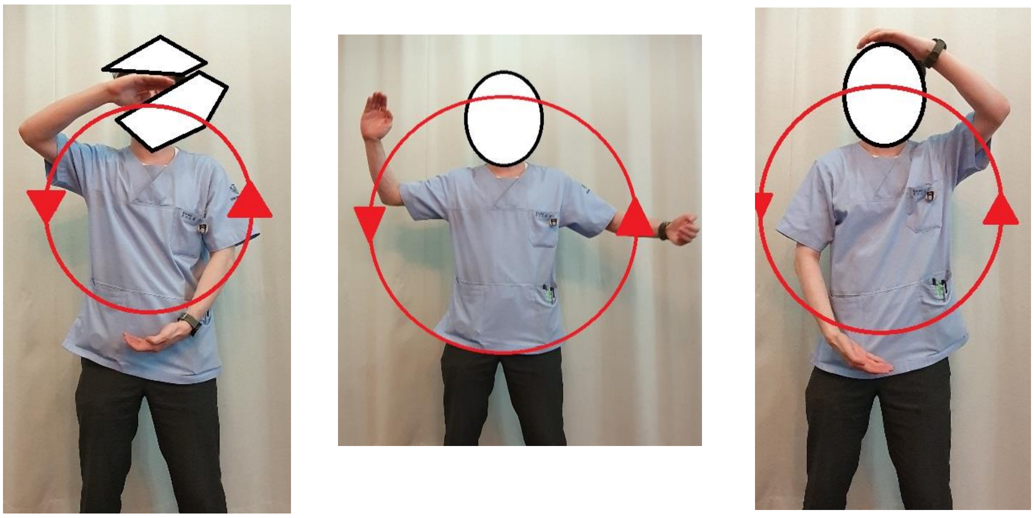

Raising energy **Increasing energy**

Figure14. Raising energy and increasing the energy.

- Spreading the energy

Extend both arms up and down and slowly rotate them with palms facing outwards.

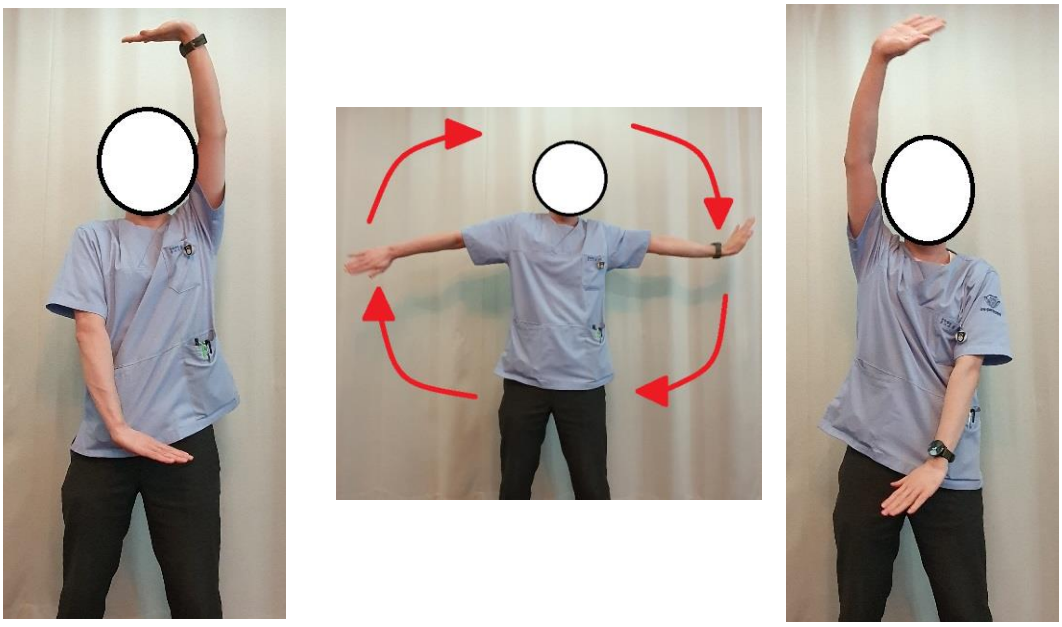

Figure15. Spreading the energy

⑦ Expanding the energy: activation of the occipital lobe, parietal lobe and frontal lobe

- Drawing the bottom circle
① Place both hands at your waist level as if you were holding a ballpoint pen.
② As the waist as the starting point, draw a circle on the bottom as if drawing a picture.
③ Draw a circle with your right hand and left hand alternately.

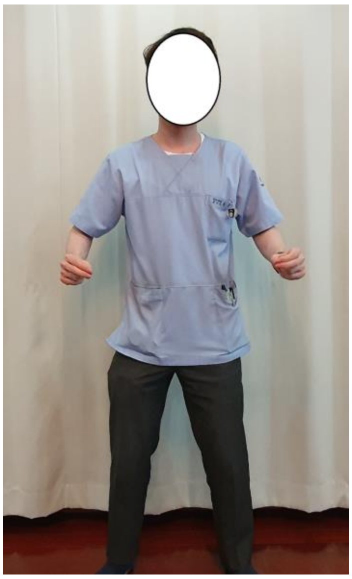

①

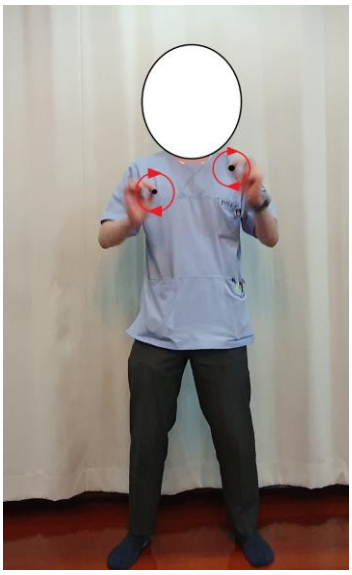

② - ③

- Drawing the front circle
① Place both hands at your waist level as if you were holding a ballpoint pen.
② As the waist as the starting point, draw a circle facing the front as if drawing a picture.
③ Draw a circle with your right hand and left hand alternately.

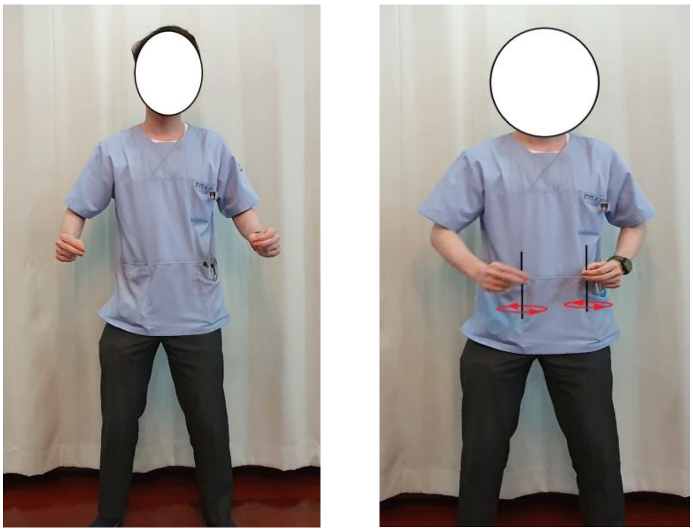

① ② - ③

Figure16. Drawing the bottom circle (above), Drawing the front circle (below)

- Drawing the front and sideway circle
① Have your hands as if holding a ballpoint pen lightly and place the left hand on the side and the right hand on the front.
② Draw a circle with both hand simultaneously.
③ Have your hands as if holding a ballpoint pen lightly and place the left hand on the front and the right hand on the side.
④ Draw a circle with both hand simultaneously.

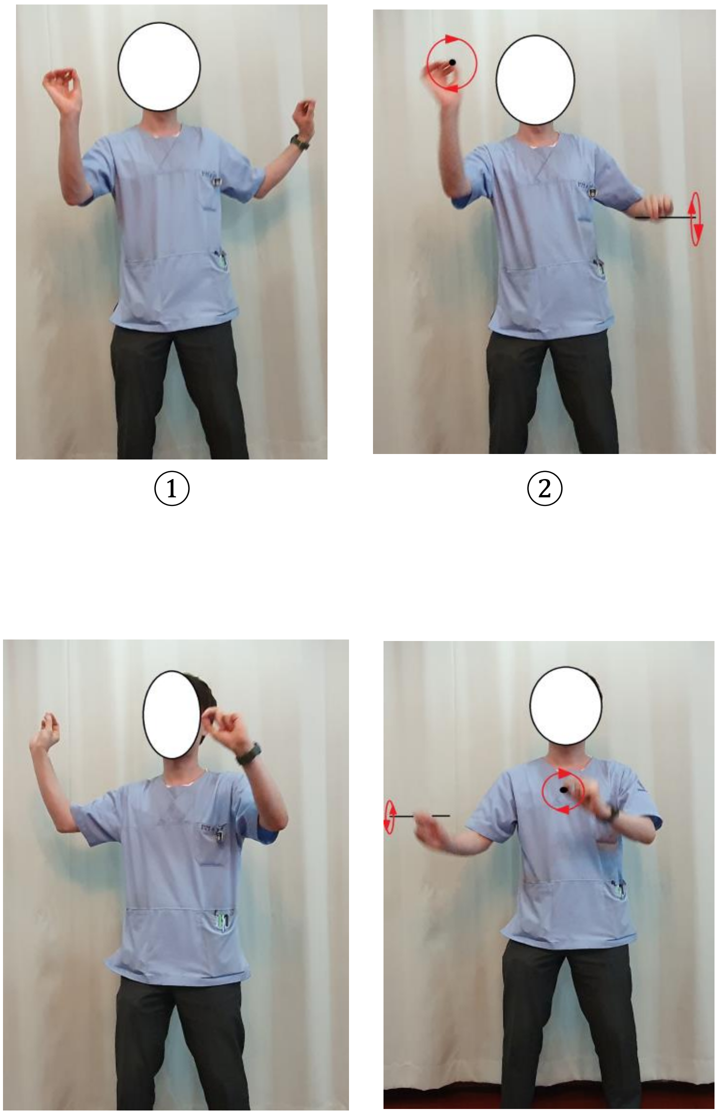

① ② ③ ④

Figure17. Drawing the front and sideway circle

⑧ Refining the whole body: relaxing the shoulder and chest muscles

- Inhaling deeply

Breath in with your chest wide open and arms raised upwards.

- Exhaling deeply

Exhale as you lower your arms over your belly

- Breathing in

With your palms facing up and your fingertips facing each other, inhale as you move your hands up towards the chest.

- Breathing out

Exhale as you put your hands facing each other, down.

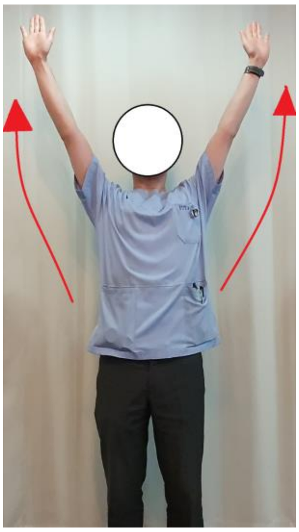

Inhaling deeply

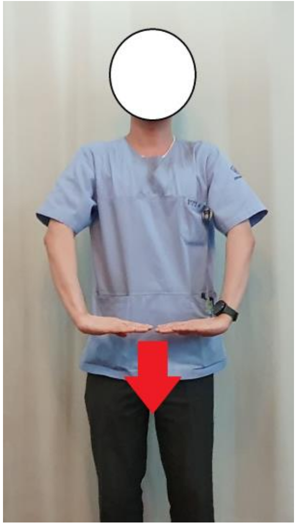

Exhaling deeply

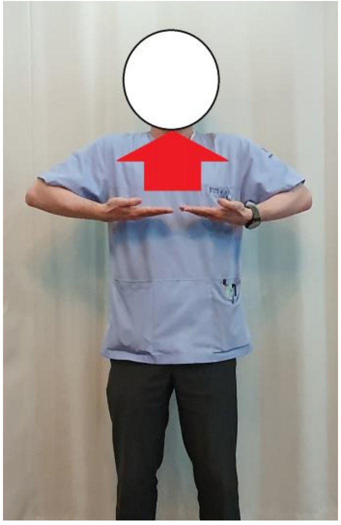

Breathing in

Figure18 Refining the whole body

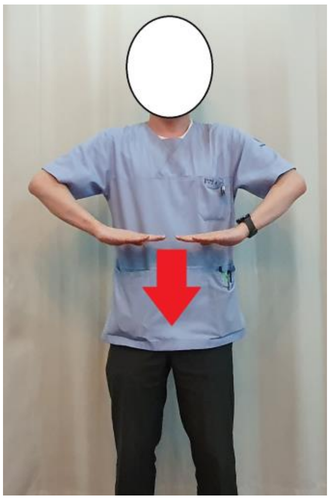

Breathing out

(4) Walking meditation method

Walking meditation is a dynamic meditation that pays attention to all the senses one feels while walking and allows one to feel all the sense of stability. It has been reported to be effective in alleviating depressive symptoms, and improving overall functional health status and vascular reactivity[198], hence can be useful at disaster support sites. In particular, this walking meditation does not require high-intensity physical activity, and can be safely performed by those with diseases and even by the elderlies who have a certain level of self-gaiting ability.

Here is a simple walking meditation (meditation performed while walking) that an oriental physician can practice with or educate the patients at disaster support sites.

Refer to the video provided by The Association of Korean Medicine on YouTube channel (AKOM_TV) or refer to the video provided by the YouTube channel of Kyunghee University Guangdong Hospital Oriental Medicine Neuropsychiatry Department. For breathing meditation, refer to the stabilization techniques of oriental medicine counseling treatment.

Figure19. Walking meditation YouTube link

Walking meditation

Now, we shall start the walking meditation.
An indoor space that is quiet or even an outdo ode space is good for walking meditation.
However, avoid areas where there are objects that could be in the way or where vehicles such as cars, motorcycles, or bicycles can pass.

Now, stand up straight with both feet on the ground.
Stand with your feet a span or two apart so that you can feel a sense of stability.
Let your arms hang comfortably on either side of your body.

Quietly close your eyes.
However, for those who have difficulty in balancing or feel dizzy,
It is okay to keep your eyes open.

Take three slow breaths.

Reaffirm that both soles of the feet feel attached to the surface of the ground.
Whether the ground hard, soft, flat, bumpy, cold or warm…
Feel yourself standing firmly with your soles attached,
Much like the roots of a tree.

Breath slowly and inspect your own movements.
Like a tree bellowing softly when the wind blows
Make sure your body is also moving little by little while keeping the balance.
Notice the slight changes in the areas where the pressure is felt
on the soles of the feet is that is in contact with the ground in order to maintain balance.

The heels, middle of the sole, outer sole, front of the sole,
toes from the big toe to the little toe
Determine where the pressure is felt onnoteoles of the feet.

Observe yourself standing securely
With the soles of your feet rooting you to the ground.

Now, we will try walking slowly.
We won't move straight into the action.
Be conscious and think that you will now have to walk,
And put those thoughts into action.

Slowly raise one foot.
Observe the moment-to-moment changes in the sensations felt in your feet and legs.
Observe the pressure build-up from the feet attached to the ground.
Your ankles may shake.
Reaffirm that there is no unneeded force in your shins or calves.

Slowly step forwarder with the one leg you lifted.
Observe the your body shaking as a whole.
Do you have any desire do put your feet on the ground quickly?
Do you have any uneasy thoughts because your body is shaking?
Observe these thoughts that come from within you.

Gradually lower the foot in front of you, starting with the hell, on to the ground.
The heels, middle of the sole, outer sole, front of the sole, the toes.
Observe the sensation of your feet gradually touching the ground.

Confirm that your body that was shaking is also stable.
Make sure the center of gravity is distributed evenly across both legs.
What kind of sensation do you feel right now in your feet, ankles, shins, calves, and thighs?

Observe the moment-to-moment chances in the sensation of your feet and legs.
Observe the pressure build-up from the feet attached to the ground.
Your ankles may shake.
Reaffirm that there is no unneeded force in your shins or calves.

Slowly step forwarder with the one leg you lifted.
Observe the your body shaking as a whole.
Do you have any desire do put your feet on the ground quickly?
Do you have any uneasy thoughts because your body is shaking?
Observe these thoughts that come from within you.

Gradually lower the foot in front of you, starting with the hell, on to the ground.
The heels, middle of the sole, outer sole, front of the sole, the toes.
Observe the sensation of your feet gradually touching the ground.
Confirm that your body that was shaking is also stable.
Make sure the center of gravity is distributed evenly across both legs.
What kind of sensation do you feel right now in your feet, ankles, shins, calves, and thighs?

Walk slowly for 5 to 10 minutes in the same way as now.
It is okay to walk around in a circle,
It is okay to walk in only one direction
And it is also okay to change directions as you walk.

Pay attention to all the sensations you feel while walking
And try walking.

…

(Wrapping up) Now, after thinking of stopping the movements,

Put those thoughts into action.

Stand up straight with your two feet on the ground just as you did in the beginning.
Stand with your feet a span or two apart so that you can feel a sense of stability.
Let your arms hang comfortably on either side of your body.

Make sure that both soles of the feet feel attached to the ground.
Feel yourself standing still, firmly.

You can feel your movements as you breath slowly.
Shift the center of gravity between your feet and legs,
And reaffirm sure your body is in balance.
For a while, feel the sense of security until you feel is enough.

Now, take 3 slow breaths and finish the walking meditation.
Thank you for your effort.

IV. Appendices

Appendix1. Diagnosis Criteria for Post-traumatic stress Disorder (DSM-5)
Appendix2. Medical record (Example)
Appendix3. Consent for personal information collection and usage (Example)
Appendix4. Screening test for PTSD (The Korean Version of the Primary Care PTSD Screen for DSM-5, K-PC-PTSD-5)
Appendix5. Screening test for depression (Patient Health Questionnaire, PHQ-9)
Appendix6. Anxiety questionnaire (Generalized Anxiety Disorder 7-item scale, GAD-7)
Appendix7. Health questionnaire-15 (Patient Health Questionnaire, PHQ-15)
Appendix8. Suicide risk questionnaire
Appendix9. Group therapy example
Appendix10. Emotional freedom technique self-care study sheet

Appendix1. Diagnosis Criteria for PTSD (DSM-5)

Diagnosis Criteria for PTSD (DSM-5)
The detailed diagnostic criteria differ by era, as do the standard set by the American Psychiatric Association and the World Health Organization. However, it can usually be diagnosed if it includes the following symptoms and the symptoms persist for more than a month.
**A. Exposed to actual death or threatened death, serious injury or sexual violence**<br>1) Experience the traumatic event firsthand<br>2) Witnessing the traumatic event happen to another person<br>3) Finding out that an incident occurred to a close family member or friend (if a family member or a friend actually died or experienced a similar situation, the incident must be violent or sudden)<br>4) Experiencing repeated or severe exposure to the repulsive details of the traumatic event (E.g.: first responders working on collecting corpses, police officers repeatedly exposed to detailed of child abuse)<br>Note: 4) Item for is not occupation-related and does not apply to exposure through electronic media, television, film or photography.
**B. One or more traumatic event-related invasive symptoms**<br>1) Repetitive, involuntary and invasive memories of the painful traumatic event<br>2) Reoccurrence of distressing dreams related to the event<br>3) Dissociative reactions (e.g. flashbacks) in which the Peron feels or acts as if the event is reoccurring (these reactions vary, and in severe cases may be completely unaware of the present situation).<br>4) Strong or persistent psychological distress when exposed to stimuli that are similar or a symbolic part of the event.<br>5) Significant psychological effect that occur when exposed to stimuli that are similar to or a symbolic part of the vent.
**C. Onset of persistent avoidance of related stimuli, with one or both of the following symptoms**<br>1) Avoiding or trying to avoid painful memories, thoughts or feelings related to the traumatic event.<br>2) Avoiding or trying to avoid traumatic events and external factors that recall painful memories, thoughts or emotions (E.g.: people, places, conversations, activities, objects, situations).

D. Negative alteration in cognition and mood related to the event that started or worsened after the traumatic event occurred, with two or more of the following:

1) Inability to remember important aspects of the traumatic event
   - Typically due to dissociative amnesia
   - Excludes other factors such as head injuries, alcohol or drugs

2) Persistent and excessively negative beliefs or expectations about self, others or the world;
   - e.g.) "I am bad."
   - "No one can be trusted."
   - "Everything in the world is dangerous."
   - "All my nervous systems are completely broken."

3) Persistent and distorted cognition that leads to blaming oneself or others for the cause or effect of the traumatic event.

4) Persistent negative emotions (e.g.: fear, horror, anger, guilt or shame)

5) Severe decrease in interest or participation in meaningful activities

6) Feeling isolated or alienated from others

7) Inability to consistently experience positive emotions (e.g.: inability to experience feelings of happiness, satisfaction or love)

E. Severe changes in arousal and response associated with the traumatic event that began or worsened after two or more of the following events have occurred:

1) Irritable behaviors and outburst of anger, typically expressed as verbal or physical aggression towards others or objects (with little to no trigger)

2) Reckless and self-destructive behavior

3) Hyper arousal

4) Excessively surprised reaction

5) Difficulty in concentrating

6) Sleep disturbances(e.g.: having difficulty falling asleep, staying asleep or not getting a good night's sleep)

※Acute stress disorder

• The symptoms are the same as those of PTSD but symptoms appear within 4 weeks after the traumatic experience and the symptoms last within 4 weeks.

Appendix2. Medical record (Example)

Demographic Survey and Basic Questionnaire					
Name/ Gender/ Age		☐ Male ☐ Female	Years of age	Birth Date	Year Month Date
Marriage Status	☐ Married ☐ Single □Divorced □Bereavement □Other			Height /Weight	cm kg
Occupation				Date of Onset	Year Month Date
Special note	□Infant □Elderly □Disabled □Mentally disabled □Pregnant □Others( )			Trauma Type	□Threats to life □Physical injury □Witness(death/physical injury) □Cognitive(death of family/friend)
Vital Signs	Systolic/diastolic blood pressure (left): / mmHg Pulse(left) :/min Temperature:. ℃ Respiratory rate: /min				
Chief Complaint	(SUDs: ___Unit)				
Present	Physical	Emotional	Cognitive	Behavioral	

Symptoms	□ Headache<br>□ Dizziness<br>□ Insomnia<br>□ Loss of appetite<br>□ Dyspepsia<br>□ Tightness of the chest<br>□ Heart palpitation<br>□ Fatigue<br>□ Pain	□ Anxiety<br>□ Fear<br>□ Sadness<br>□ Depression<br>□ Despair<br>□ Anger<br>□ Shame<br>□ Guilt	□ Loss of concentration<br>□ Memory loss<br>□ Nightmares<br>□ Terrible memories that keeps coming back<br>□ Inability to accept the death of someone close<br>□ Preoccupation with death	□ Stupor<br>□ Avoidance/Denial<br>□ Social avoidance<br>□ Self-harm<br>□ Regression<br>□ Violence/abusive language<br>□ Excessive drinking/smoking<br>□ Decreased coping ability	
	Other symptoms:				
Past History	□ High blood pressure □ Diabetes □ Hyperlipidemia □ Liver disease □ Heart disease □ Stroke(cerebral palsy) □ Cancer □ Thyroid disease □ Degenerative arthritis □ Hernia of intervertebral discs/spinal canal stenosis □ Other( )				
Psychiatric Treatment Experience	□ Yes: Diagnostic name( ) Medication taken( ) □ No				
Medication taken					
Smoking	___pack/day * ___Year	Drinking	__times/week * Alcohol tolerance______ * __ Year		
Name of Diseases					
Screening Tests	PTSD(PC-PTSD-5): ___points	Depression (PHQ-9): ___ points	Anxiety(GAD-7): ___ points	Physical Health(PHQ-15): ___ points	Suicide(P4): ___ points
Risk Factors	Psychiatric past ( )	Past traumatic experiences ( )	Current stressful event( )	Weak support system ( )	

Oriental physical name: (sign/seal)

Submission Date: Year Month Date

Appendix3. Consent for personal information collection and usage (Example)

We would like to collect and use personal information as follows for the treatment of oriental medicine. Please read the contents carefully and decide whether to agree or not.

『Personal information』 · 『Sensitive information and unique identification information』 are retained and used only for the period of providing disaster mental health services for the 'purpose of collecting and using information' and the information collected for service provision is retained in within the provisions of the law and in accordance with the Personal Information Protection Act, Medical Act, Mental Health Promotion and Welfare Service Support for Mental Illness Act. However, even if the purpose of collection and provisions has been achieved, personal information may be retained if there is a need to retain it according to other laws and regulations.

□ **Personal information collection • usage history**

☞

Do you agree to the collection of personal information as above?

Agree □ Disagree □

**Collection and usage items**	**Purpose of collection and usage**	**Retention period**	**Basis for retention**
•Name<br>•Gender<br>•Birth Date<br>•Contact information<br>• Address	• Data needed for **oriental medicine treatment** for the recovery of those who have experienced disasters<br>• Used as a smooth communication path to **deliver notices**, etc. **(phone, SMS)**<br>• Analysis data needed for **education and research**	**5 years**	『Standard Personal Information Protection Guidelines』 Article 60, [Attached Table No.1] Criteria for setting personal information file retention period

☞ Do you agree to the usage of personal information as above? Agree □ Disagree □

□ **Sensitive information collection • usage history**

**Collection and usage items**	**Purpose of collection and usage**	**Retention period**	**Basis for retention**

**<u>Psychological and physical symptoms, medical history and personal health information deemed necessary by the services provide due to the disaster experience</u>**	•Data needed for oriental medical treatment, investigation and research for victims of disasters	**<u>5years</u>**	『Standard Personal Information Protection Guidelines』 Article 60, [Attached Table No.1] Criteria for setting personal information file retention period

☞ Do you agree to the collection of sensitive information as above? Agree □ Disagree □

☞ Do you agree to the usage of sensitive information as above? Agree □ Disagree □

□ **Details of provision of personal and sensitive information to third parties**

Receiving institution	Purpose of provision	Items provided	Retention period
**<u>Disaster psychological support related organization and medical institutions</u>**	**<u>Linking with institutions or treatment institution necessary for recovery of those who have experienced disasters</u>**	Name, gender, date of birth, contact information, place of residence, health information related to disaster experience	**<u>5 years</u>**

☞ Do you agree to the provision of personal and sensitive information to third parties as above? Agree □ Disagree □

□ **Information on refusal of consent**

※ Personal and sensitive information collected and used will not done so for any other purpose. You have the right to refuse the provision and use of personal information, in which case you may be restricted from using oriental medical services.

20 Year Month Date

Person : __________________________(Sign)

Legal representative :

___________________________(Sign)

Appendix4. Korean version of the screening test for PTSD (The Korean Version of the Primary Care PTSD Screen for DSM-5, K-PC-PTSD-5)[199200]

Sometimes unusually frightening, terrifying or shocking **things can happen someti mes. For example:**

• Serious accidents or fires<br>• Physical violence, sexual violence, physical abuse or sexual abuse<br>• Earthquakes or floods<br>• War<br>• Witnessing someone else dead or seriously injured<br>• A loved one dies by murder or suicide

Have you ever experienced this kind of event before?<br>□ Yes □ No

In the past month, have you had any of the following experiences due to a scary, terrifying, difficult, or any other experiences in your life?

1. There have been times when I had nightmares about the experience or even thought about even though I didn’t want to.	0) No 1) Yes
2. I tried not to think about the experience, or made special efforts to avoid situations that would remind me of the experience.	0) No 1) Yes
3. There have been instances when I constantly alert, unable to relax or easily startled.	0) No 1) Yes
4. There have been times when I had no feelings about other people, daily activities or surroundings, or felt isolated from others.	0) No 1) Yes
5. I felt guilty about the event or the problems it caused, or could not stop resenting myself or others.	0) No 1) Yes

Appendix5. Screening test for depression (Patient Health Questionnaire, PHQ-9)[201202]

In the past two weeks, please indicate (V) how often you have been disturbed by the following problems:

	Never	For several days	For more than a week	Almost daily
1) Lack of interest or pleasure in work or leisure activities.	0	1	2	3
2) Feeling down, depressed, or hopeless	0	1	2	3
3) Difficulty falling asleep or staying asleep, or sleeping too much.	0	1	2	3
4) Feeling tired or lethargic	0	1	2	3
5) Lack of appetite or overeating	0	1	2	3
6) Sees oneself negatively. Or feels like a failure and disappoints self or family.	0	1	2	3
7) Difficulty concentrating on tasks such as reading the newspapers or watching TV	0	1	2	3
8) Moving or speaking so slowly enough that it is noticed by others. Or, conversely, moving more than usual, being too restless or excited.	0	1	2	3
9) Thinking that you would be better off dead or that you would harm yourself in anyway.	0	1	2	3

If you have checked any of the symptoms with a score of 1 or higher, how much difficulty did you have at work, household chores or social relationships because of the symptoms?
① Not at all ② Somewhat difficult ③ Very difficult ④ Extremely difficult

Appendix6. Anxiety questionnaire (Generalized Anxiety Disorder 7-item scale, GAD-7)[203]

the past two weeks, how often have you been disturbed by the following problems?

	Never	For s evera l day s	For more than a w eek	Almo st dai ly
1) Feeling anxious, nervous or impatient.	0	1	2	3
2) Not able to control your worries.	0	1	2	3
3) Worrying too much about many times.	0	1	2	3
4) It is difficult to be comfortable.	0	1	2	3
5) Easily irritable or quick to anger.	0	1	2	3
6) Feeling so restless that it is difficult to stay still.	0	1	2	3
7) Feeling afraid as if something terrible is about to happen.	0	1	2	3

Appendix7. Health questionnaire-15 (Patient Health Questionnaire, PHQ-15)[204205]

In the past 4 weeks, how often have you suffered from the symptoms listed below?

	Never afflicted	Slightly afflicted	Very afflicted
Stomach ache	0	1	2
Back pain	0	1	2
Pain in the arms, legs, or joints (knee, hip, etc.)	0	1	2
Problems such as menstrual cramps during menstruation [Women only]	0	1	2
Headache	0	1	2
Chest pain, pleurodynia	0	1	2
Dizziness	0	1	2
Feeling faint	0	1	2
Heart beating fast	0	1	2
Being out of breath	0	1	2
Problems such as pain during inter course	0	1	2
Constipation, loose stools or diarrh ea	0	1	2
Nausea, gas, dyspepsia	0	1	2
Fatigue, lethargy	0	1	2
Difficulty sleeping	0	1	2

Appendix8. Suicide risk questionnaire[206207]

Have you ever thought of really hurting yourself?			Yes	No
Please answer questions 1 to 4-1 below only if you have checked ‘yes’. Those who have checked ‘no’ do not need to answer.			Yes	No
1	Have you ever done anything that put you at risk before?		Yes	No
2	Are you still thinking about the methods of really hurting yourself?			
2-1	If so, in what ways? ( )			
3	There is a big difference between thinking about it and putting it into action. At ay point within the month, are you likely to act on the thought of harming yourself or ending your life?	Not at all	Slightly	Very much so
4	Is there anything that stops or prevents you from doing something to harm yourself?		Yes	No
4-1	If so, what is it? ( )			

Appendix9. Group therapy example

Session	Content	Notes

1	An overview of anxiety<br>EFT Introduction<br>Learning and practicing the predatory work and continuous tapping during clinical EFT	√ Overview of anxiety symptoms<br>√ Effects of stress on health and life<br>√ Learning and practicing the predatory work of clinical EFT<br>√ Practice with practice topics that focus on general anxiety symptoms and general negative beliefs that cause anxiety.<br>√ Distribute EFT voice files and practice checklists (practice at least once a day)<br>√ Provide self-practice papers for situation that make you anxious in daily life as assignments and check changes in SUD (subjective pain index)
2	Review of first session<br>Learning and practicing affirmation techniques during clinical EFT	√ Used for changing perception about memory beliefs by connecting with the predatory works in clinical EFT<br>√ Practice with practice topics that focus on general anxiety symptoms and general negative beliefs that cause anxiety.<br>√ Distribute EFT voice files and practice checklists (practice at least once a day)<br>√ Provide self-practice papers for situation that make you anxious in daily life as assignments and check changes in SUD
3	Review sessions 1~2<br>Learning and practicing the brain tuning process of EFT Basic recipe<br>Group practice on the subject’s individual situation	√ Learning and practicing brain tuning in EFT<br>√ Practice to cultivate the ability to self-care according to each situation<br>√ Distribute EFT voice files and practice checklists (practice at least once a day)<br>√ Provide self-practice papers for situation that make you anxious in daily life as assignments and check changes in SUD
4	Review sessions 1~3<br>Group practice on the subject’s individual situation	√ Completed clinical EFT predatory work, continuous tapping and choice method<br>√ Practice to cultivate the ability to self-care according to each situation<br>√ Distribute EFT voice files and practice checklists (practice at least once a day)<br>√ Provide self-practice papers for situation that make you anxious in daily life as assignments and check changes in SUD

Appendix10. Emotional freedom technique self-care study sheet

Emotional freedom technique (EFT) self-care study sheet

1. Close your eyes and take one deep breath.

2. Choose a person or a situation in your daily life that makes you anxious, angry, irritable or uncomfortable. What happened?

3. What emotions or symptoms do you get when you think these thoughts?

(Example) angry, irritated, unmotivated, annoyed, anxious, nervous, sad, resentful, disappointed, sorrowful, regretful, pathetic, headaches, dizzy, indigested, frustrated, heart palpitation, chest burns.

4. How do you rate that feeling on a scale of 0 to 10?
(*Write in the column next to the feelings you wrote down.)

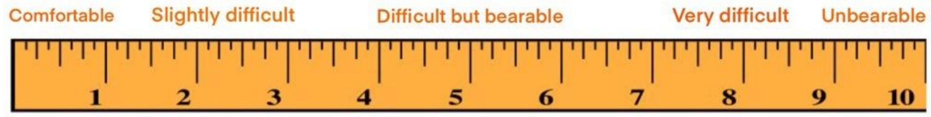

1. Precatory work(Set-up)
Apply the affirmation that are right for you by different periods.
In ____, you can put the situation/emotions you want to solve as it is.

*Repeat the above sentence 3 times.

By period	Affirmation choice school
Acute (~1month)	Even though I ______, I choose to realize that I am now safe.
Sub-acute (1~3mont)	Even though I am ______, I am safe now. I sincerely and fully accept myself completely.”
Chronic (3months)	Even though I may be _______, I sincerely accept myself as is from the bottom of my heart.

2. Continuous tapping (Sequence)
*Repeated tapping with uncomfortable emotional symptoms

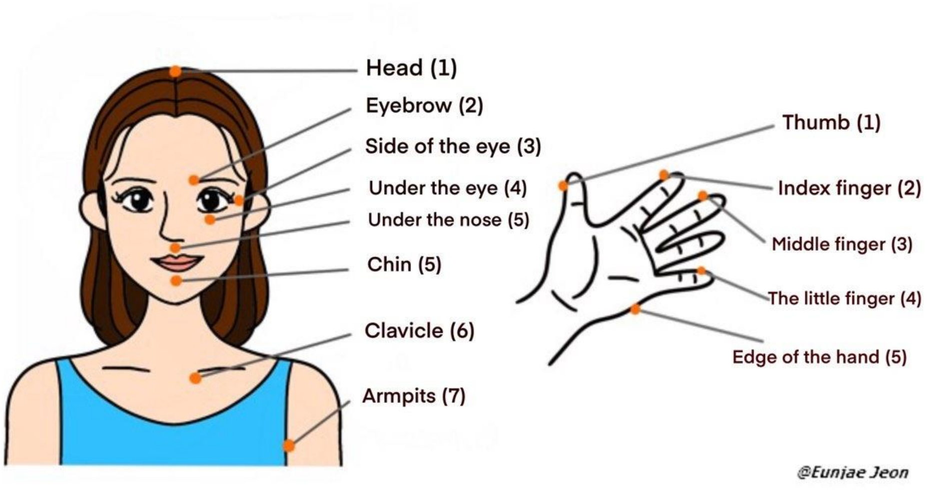

3. Affirmation of choice (Choice method)

In spite of the bad memories, the uncertain future and the present situation that is hard on me,

I will not harm myself.

I will not criticize myself.

I will not underestimate myself.

I live as the protagonist of my life.

I truly understand, accept and love myself.

I live a life of understanding and loving myself more and more.

I feel more and more comfortable.

I am getting healthier.

I am getting more pleasant.

I am getting happier.

Academic Certification for Practice guideline for the treatment of Disaster-related trauma using korean medicine

Practice guideline for the treatment of Disaster-related trauma using oriental medicine Academic Certificate

The certification evaluation committee of The Society of Korean Oriental Neuropsychiatry have reviewed the 'Practice guideline for the treatment of Disaster-related trauma using oriental medicine' developed by Professor Kim Sangho's research team in the Department of Oriental Medicine at Daegu Haany University and the content is hereby accredited by the society.

September 3rd, 2021

President of the Korean Oriental Neuropsychiatry Society

[1] The American Psychiatric Association (APA). Coping After Disaster. [Cited Dec 24, 2020] Av

ailable:https://www.psychiatry.org/patients-families/coping-after-disaster-trauma#:-:text=Common%20reactions%20in%20adults%20after%20a%20disaster%3A&text=Sadness%2C%20depression%2C%20hyperactivity%2C%20irritability,at%20all%20or%20feeling%20numb.

[2] Kwon Jung-Hae, Ahn Hyun-Eu, Choi Yoon-Gyung, Joo Hae-Seon. Psychological Emergency Practices for Disaster and Trauma Volume 2. Seoul: Hakjisa (2014). p30-31.

[3] Kwon Jung-Hae, Ahn Hyun-Eu, Choi Yoon-Gyung, Joo Hae-Seon. Psychological Emergency Practices for Disaster and Trauma Volume 2. Seoul: Hakjisa (2014). p32.

[4] Kwon Jung-Hae, Ahn Hyun-Eu, Choi Yoon-Gyung, Joo Hae-Seon. Psychological Emergency Practices for Disaster and Trauma Volume 2. Seoul: Hakjisa (2014). p34-37

[5] Korean Disaster Mental Heatlh Committee. Disaster and Mental Health. Seoul:Hakjisa(2015). p111-118

[6] Zunin & Myers as cited in DeWolfe, D. J., 2000. Training manual for mental health and human service workers in major disasters (2nd ed., HHS Publication No. ADM 90-538)

[7] Bromet EJ, Atwoli L, Kawakami N, Navarro-Mateu F, Piotrowski P, King AJ, Aguilar-Gaxiola S, Alonso J, Bunting B, Demyttenaere K, Florescu S, de Girolamo G, Gluzman S, Haro JM, de Jonge P, Karam EG, Lee S, Kovess-Masfety V, Medina-Mora ME, Mneimneh Z, Pennell BE, Posada-Villa J, Salmerón D, Takeshima T, Kessler RC. Post-traumatic stress disorder associated with natural and human-made disasters in the World Mental Health Surveys. Psychol Med. 2017 Jan;47(2):227-241.

[8] Kessler RC, McLaughlin KA, Koenen KC, Petukhova M, Hill ED, World Health Organization(WHO) World Mental Health Survey Consortium. The importance of secondary trauma exposure for post-disaster mental disorder. Epidemiology and psychiatric sciences. 2012 Mar;21(1):35.

[9] American Psychiatric Association. Diagnostic and Statistical Manual of Mental Disorders (The 5th Edition)(Kwon Jun-Soo, Kim Jae-Jin, Nam Goong-Ki, Park Won-Myung, Shin Min-Sub, Yoo Bum-Hee. Lim Hyo-Duk). Seoul: Hakjisa. p289-292.

[10] North CS, Pfefferbaum B. Mental health response to community disasters: a systematic review. Jama. 2013 Aug 7;310(5):507-18.

[11] Shultz JM, Forbes D. Psychological first aid: Rapid proliferation and the search for evidence. Disaster Health. 2014 Jan 1;2(1):3-12.

[12] National Center of Disaster Trauma. Standardized manual for disaster mental health workers (Draft). Seoul: National Center of Mental Health ;2019. p13-17.

[13] National Center of Disaster Trauma. Standardized manual for disaster mental health workers (Draft). Seoul: National Center of Mental Health ;2019. p31-52.

[14] Koh Young-Hoon. Ansan Mental Health Trauma Center Disaster psychological support guide. Geyonggi:Ansan Mental Health Trauma Center;2017. p14-31.

[15] Ministry of Interior and Safety. Revised version of Diaster psychological recovery support manual. Ministry of Interior and Safety, Division of Disaster Relief;2017. 83p.

[16] Hoff, L. A. People in crisis: Understanding and helping 4th ed. Redwood city, CA:Addison-Wesley;1995. 105-132p.

[17] Myer RA, Conte C. Assessment for crisis intervention. J Clin Psychol. 2006;62(8):959-970.

[18] National Center of Disaster Trauma. Standardized manual for disaster mental health workers

(Draft) Amendment. Seoul: National Center of Mental Health ;2019.2019, 121-125p

[19] Jung YE, Kim D, Kim WH, Roh D, Chae JH, Park JE. A Brief Screening Tool for PTSD: Validation of the Korean Version of the Primary Care PTSD Screen for DSM-5(K-PC-PTSD-5). Journal of Korean Medical Science 2018;33(52):e338.

[20] Spitzer RL., Kroenke K., Williams JBW. Validation and utility of a self-report version of PRIME-MD: the PHQ primary care study. The Journal of the American Medical Association. 1999;282(18);1737-1744.

[21] Ahn Jae-Yong, Seo Eun-Ran, Lim Gyung-Hee, Shin Jae-Hyun, Kim Jung-Bum. Standardization of the Korean version of Screening Tool for Depression(Patient Health Questionnaire-9, PHQ-9) 2013 Jun;19(1):47-56.

[22] Pfizer Inc. The Korean Version of the GAD-7. (2018). Available online at: http://www.phqscreeners.com (Accessed April 1, 2018).

[23] Ahn JK, Kim Y, Choi KH. The psychometric properties and clinical utility of the Korean version of GAD-7 and GAD-2. Frontiers in psychiatry. 2019 Mar 18;10:127.

[24] Kroenke K., Spitzer RL., Williams JB. The PHQ–15: validity of a new measure for evaluating the severity of somatic symptoms. Psychosomatic Medicine. 2002;64:258–266.

[25] Han C, Pae CU, Patkar AA, Masand PS, Kim KW, Joe SH, Jung IK. Psychometric properties of the Patient Health Questionnaire-15(PHQ-15) for measuring the somatic symptoms of psychiatric outpatients. Psychosomatics. 2009;50(6):580-585.

[26] Park Joo-Eon, Kim Won-Hyung, Noh Dae-Young, Won Seong-Doo, Kim Ha-Kyung, Kang Seok-Hoon, Hong Na-Rae, Park Seong-Yong, Kim Dae-Ho, Chae Jeong-Ho. Disaster Trauma Mental Health Assessment Workbook. Korean Society of Anxiety, Korea Mental Health R&D Project. 2016.

[27] National Center of Disaster Trauma/Disaster Mental Health Information/Self-Diagnosishttps://nct.go.kr/distMental/rating/rating02_1.do

[28] Park Joo-Eon, Kim Won-Hyung, Noh Dae-Young, Won Seong-Doo, Kim Ha-Kyung, Kang Seok-Hoon, Hong Na-Rae, Park Seong-Yong, Kim Dae-Ho, Chae Jeong-Ho. Disaster Trauma Mental Health Assessment Workbook. Korean Society of Anxiety, Korea Mental Health R&D Project. 2016.

[29] The Ear acupuncture treatment protocol was first developed by the National Acupuncture Detoxification Association (NADA) as an adjuvant treatment for Heroin addiction, and since the 9.11 terrorism in 2001 and Hurricane Katrina, has been used for people suffering various mental disorders and disaster trauma. NADA is developing disaster response manpower through systematic training programs.

[30] Park HI et al. Effect of Korean Version of Psychological First Aid Training Program on Training Disaster Mental Health Service Provider. J Korean Neuropsychiatr Assoc. 2020;59(2): 123-135.

[31] Brymer M, Jacobs A, Layne C, Pynoos R, Ruzek J, Steinberg A, Vernberg E, Watson P. Psychological first aid: field operations guide. 2nd ed. Los Angeles, CA: National Child Traumatic Stress Network and National Center for PTSD; 2006.

[32] Pynoos RS, Nader K. Psychological first aid and treatment approach to children exposed t

o community violence: research implications. J Trauma Stress 1988;1(4):445-473

[33] Dieltjens T, Moonens I, Van Praet K, De Buck E, Vandekerckhove P. A systematic literature search on psychological first aid: lack of evidence to develop guidelines. PLoS One. 2014;9:e114714

[34] Brymer et al. Psychological first aid: field operations guide (2nc edition). National Child Traumatic Stress Network & National Center for PTSD; 2006.

[35] Hobfoll SE, Watson P, Bell CC, Bryant RA, Brymer MJ, Friedman MJ, Friedman M, Gersons BP, De Jong JT, Layne CM, Maguen S. Five essential elements of immediate and mid–term mass trauma intervention: Empirical evidence. Psychiatry: Interpersonal and Biological Processes. 2007;70(4):283-315.

[36] The National Child Traumatic Stress Network. PSYCHOLOGICAL FIRST AID (PFA) FIELD OPERATIONS GUIDE: 2ND EDITION. [Cited 21 Dec, 2020]. Available:https://www.nctsn.org/resources/psychological-first-aid-pfa-field-operations-guide-2nd-edition

[37] World Health Organization, War Trauma Foundation & World Vision International. Psychological first aid: Guide for field workers. World Health Organization; 2011

[38] National Center of Disaster Trauma. Standardized manual for Disaster Mental Health Workers. Seoul: National Center for Mental Health; 2019. 58-63p.

[39] The National Child Traumatic Stress Network. PSYCHOLOGICAL FIRST AID (PFA) FIELD OPERATIONS GUIDE: 2ND EDITION. [Cited 21 Dec, 2020]. Available:https://www.nctsn.org/resources/psychological-first-aid-pfa-field-operations-guide-2nd-edition

[40] https://nct.go.kr/

[41] Mattei A, Fiasca F, Mazzei M, Necozione S, Bianchini V. Stress and Burnout in Health-Care Workers after the 2009 L'Aquila Earthquake: A Cross-Sectional Observational Study. Front Psychiatry. 2017 Jun 12;8:98.

[42] Bemis R./NADA Literature Clearinghouse. Ear Acupuncture and Humanitarian Aid: History, application, and improvement of the NADA model. [Cited 10 August 2020] Available from: https://acudetox.com/ear-acupuncture-and-humanitarian-aid/

[43] Corrêa HP, Moura CC, Azevedo C, Bernardes MFVG, Mata LRFPD, Chianca TCM. Effects of auriculotherapy on stress, anxiety and depression in adults and older adults: a systematic review. Rev Esc Enferm USP. 2020 Oct 26;54:e03626.

[44] Tan HJ, Lan Y, Wu FS, Zhang HD, Wu L, Wu X, Liang FR. Auricular acupuncture for primary insomnia: a systematic review based on GRADE system. Zhongguo Zhen Jiu. 2014 Jul;34(7):726-30.

[45] Kwon CY, Lee B, Kim SH. Effectiveness and safety of ear acupuncture for trauma-related mental disorders after large-scale disasters: A PRISMA-compliant systematic review. Medicine (Baltimore). 2020 Feb;99(8):e19342.

[46] Yarberry, M. The use of the NADA protocol for Post-Traumatic Stress-Disorder in Kenya. German Journal of Acupuncture & Related Techniques. 2010 Oct 53(4):6-11.

[47] Yarberry, M. NADA Training Provides Post-Traumatic Stress Disorder Relief in Haiti. German Journal of Acupuncture & Related Techniques. 2011 Jan 54(1):21-24.

[48] Claudia Voyles, Rachel Toomim and Libby Stuyt. Acupuncture Detoxification Specialist Training Resource Manua(Fifth Edition)l: A handbook for individuals training in the National Acupuncture Detoxification Association's Five-needle Acudetox Protocol. National Acupuncture Detoxification Association;2017. 104-105p.

[49] Acupuncture Without Borders. National(USA) Programs. Available from: https://acuwithoutborders.org/international-work/ [Cited 25 Jan 2021]

[50] Kim SH, Kwon CY, Kim ST, Han SY. Ear acupuncture for posttraumatic symptoms among long-term evacuees following the 2017 Pohang earthquake: a retrospective case series study. Integr Med Res. 2020 Dec;9(4):100415.

[51] Hou PW, Hsu HC, Lin YW, Tang NY, Cheng CY, Hsieh CL. The History, Mechanism, and Clinical Application of Auricular Therapy in Traditional Chinese Medicine. Evid Based Complement Alternat Med. 2015;2015:495684.

[52] He W, Wang X, Shi H, Shang H, Li L, Jing X, Zhu B. Auricular acupuncture and vagal regulation. Evid Based Complement Alternat Med. 2012;2012:786839.

[53] Mercante B, Deriu F, Rangon CM. Auricular Neuromodulation: The Emerging Concept beyond the Stimulation of Vagus and Trigeminal Nerves. Medicines (Basel). 2018 Jan 21;5(1):10.

[54] Kwon C-Y, Lee B, Kim S-H. Efficacy and Underlying Mechanism of Acupuncture in the Treatment of Posttraumatic Stress Disorder: A Systematic Review of Animal Studies. Journal of Clinical Medicine. 2021; 10(8):1575.

[55] Kim Da-Eun, Kim Sang-Ho. Use of ear treatment for Disaster trauma– Introduction of NADA Protocol. Dongui Neuropsychiatric Association. 2020;31(3):157-168.

[56] Korean Acupuncture Society Textbook Compilation Committee. Acupuncture medicine. Seoul(Korean Medicine):2020.

[57] Tan JY, Molassiotis A, Wang T, Suen LK. Adverse events of auricular therapy: a systematic review. Evid Based Complement Alternat Med. 2014;2014:506758.

[58] Nielsen A, Gereau S, Tick H. Risks and Safety of Extended Auricular Therapy: A Review of Reviews and Case Reports of Adverse Events. Pain Med. 2020 Jun 1;21(6):1276-1293.

[59] Kim SH, Kwon CY, Kim ST, Han SY. Ear acupuncture for posttraumatic symptoms among long-term evacuees following the 2017 Pohang earthquake: a retrospective case series study. Integr Med Res. 2020 Dec;9(4):100415.

[60] Korean Acupuncture Society Textbook Compilation Committee. Acupuncture medicine. Seoul(Korean Medicine) 2020;249-250p.

[61] Japan Primary Case Association-Primary Care for All Team[Internet]. [cited 2020 Dec 17]. Available from https://primary-care.or.jp/jpca_eng/activities.html

[62] Disaster Acupuncture and Massage Project[Internet]. [cited 2020 Dec 17]. Available from http://sinkyu-sos.jimdo.com/

[63] Association of Medical Doctors of Asia[Internet]. [cited 2020 Dec 17]. Available from http://amda.or.jp/

[64] Zhang Y, Feng B, Xie JP, Xu FZ, Chen J. Clinical study on treatment of the earthquake-caused post-traumatic stress disorder by cognitive-behavior therapy and acupoint stimulation. J Tradit Chin Med. 2011 Mar;31(1):60-3.

[65] Takayama S, Kamiya T, Watanabe M, Hirano A, Matsuda A, Monma Y, Numata T, Kusuya

ma H, Yaegashi N. Report on disaster medical operations with acupuncture/massage ther apy after the great East Japan earthquake. Integr Med Insights. 2012;7:1-5.

66 Miwa M, Takayama S, Kaneko S. Medical support with acupuncture and massage therapies for disaster victims. J Gen Fam Med. 2017 Dec 16;19(1):15-19.

67 Moiraghi C, Poli P, Piscitelli A. An Observational Study on Acupuncture for Earthquake-Re lated Post-Traumatic Stress Disorder: The Experience of the Lombard Association of Medi cal Acupuncturists/Acupuncture in the World, in Amatrice, Central Italy. Med Acupunct. 2019 Apr 1;31(2):116-122.

68 Choi Yoo-Jin, Kwon Chan-Young, Jang Jae-Soon, Jung Ha-Young, Kim Yun-Na, Jung Seon-Yong. A Review on Clinical Research Trends in the Treatment of Posttraumatic Stress Disorder in Traditional Chinese Medicine
. Dongeui Neuropsychiatric Society. 2016;27(3):197-206.

69 Grant S, Colaiaco B, Motala A, Shanman R, Sorbero M, Hempel S. Acupuncture for the tre atment of adults with posttraumatic stress disorder: A systematic review and meta-analysi s. Journal of Trauma & Dissociation. 2018 Jan 1;19(1):39-58.

70 Joo Seong-Jun, Kwon Jeong-Eun, Kwon Chan-Young, Lee Bo-Ram, Kim Sang-Ho. A Review on Clinical Research Trends in the Treatment of Posttraumatic Stress Disorder in Traditional Korean Medicine
. Dongeui Neuropsychiatric Society. 2019;30(3):251–63.

71 Kim Keun-wu. Korean Medicine Clinical Practice Guideline for Anxiety Disorder. Guideline center for Korean Medicine. . Korean Society of Oriental Neuropsychiatry; 2017. 1-57p.

72 Kim Keun-wu. Korean Medicine Clinical Practice Guideline for Anxiety Disorder. Guideline center for Korean Medicine. . Korean Soceity of Oriental Neuropsychiatry; 2017. 1-57p.

73 Kim Jong-Woo. Korean Medicine Clinical Practice Guideline for Depression. Institute of Oriental Medicine. Korean Soceity of Oriental Neuropsychiatry; 2016. 168p.

74 Kim Bo-Kyung. Korean Medicine Clinical Practice Guideline for Insomnia. Guideline center for Korean Medicine. Korean Society of Oriental Neuropsychiatry; 2017. 1-50p.

75 Kim Jin-sung. Korean Medicine Clinical Practice Guideline for Funtional Dyspepsia. Guideline center for Korean Medicine. The Society of Internal Korean Medicine; 2020. 3-6p.

76 Kim Jin-sung. Korean Medicine Clinical Practice Guideline for Functional Dyspepsia. Guideline center f or Korean Medicine. The Society of Internal Korean MEDICINE; 2017. 1-53p.

77 Zhang Q, Gong J, Dong H, Xu S, Wang W, Huang G. Acupuncture for chronic fatigue synd rome: a systematic review and meta-analysis. Acupunct Med. 2019 Aug;37(4):211-222.

78 Lee Sang-kwan. Korean Medicine Clinical Practice Guideline for Migraines. Guideline center for Korean Medicine. The Society of Stroke on Korean Medicine; 2017. 1-28p.

79 Lee Ui-joo. Korean Medicine Clinical Practice Guideline for Dizziness. Guideline center for Korean Medicine. The Society of Sasang Constitutional Medicine; 2021. 68p.

80 Nam Dong-woo. Chronic Low Back Pain. Korean Medicine Clinical Practice Guideline for. Guideline center for Korean Medicine. Korean Acupuncture and Moxibustion Medicine Society; 2020. 65p.

81 Takayama S, Kaneko S, Numata T, Kamiya T, Arita R, Saito N, Kikuchi A, Ohsawa M, Koh

ayagawa Y, Ishii T. Literature Review: Herbal Medicine Treatment after Large-Scale Disasters. Am J Chin Med. 2017;45(7):1345-1364.

[82] Numata T, Gunfan S, Takayama S, Takahashi S, Monma Y, Kaneko S, Kuroda H, Tanaka J, Kanemura S, Nara M, Kagaya Y, Ishii T, Yaegashi N, Kohzuki M, Iwasaki K. Treatment of posttraumatic stress disorder using the traditional Japanese herbal medicine saikokeishikankyoto: a randomized, observer-blinded, controlled trial in survivors of the great East Japan earthquake and tsunami. Evid Based Complement Alternat Med. 2014;2014:683293.

[83] ISTSS Guidelines Committee. Posttraumatic stress disorder prevention and treatment guidelines methodology and recommendations. International Society for Traumatic Stress Studies (ISTSS).[Google Scholar]. 2018:p28.

[84] Meng XZ, Wu F, Wei PK, Xiu LJ, Shi J, Pang B, Sun DZ, Qin ZF, Huang Y, Lao L. A chinese herbal formula to improve general psychological status in posttraumatic stress disorder: a randomized placebo-controlled trial on sichuan earthquakesurvivors. Evid Based Complement Alternat Med. 2012;2012:691258.

[85] Soyosangagambang. From Soyo-san(Siho, danggwi, china root, baekchul, paeonia japonica, menthe arvensis L., licorice root, geinseng)exclude ginseng, and add 7 herbal medicine(coptis chinesis, pinellia ternata, chenpi, yonggol, ostrea gigas, daehwang jujung, acorus gramineus).

[86] Kim KH, Jang S, Lee JA, Jang BH, Go HY, Park S, Jo HG, Lee MS, Ko SG. Experiences Providing Medical Assistance during the Sewol Ferry Disaster Using Traditional Korean Medicine. Evid Based Complement Alternat Med. 2017;2017:3203768.

[87] Takayama, S., et al. The Role of Oriental Medicine in the Great East Japan Earthquake Disaster(東日本大震災における東洋医学による医療活動. Kampo medicine 2011;62(5):621-626.

[88] Jang S, Kim D, Yi E, Choi G, Song M, Lee EK. Telemedicine and the Use of Korean Medicine for Patients With COVID-19 in South Korea: Observational Study. JMIR Public Health and Surveillance. 2021 Jan 19;7(1):e20236.

[89] Choi Yu-Jin, Kwon Chan-Young, Jang Jae-soon, Jung Ha-Jang Jae-soon, Jung Ha-young, Kim Yun-Na, Chung Sun-Young. A Review on Clinical Research Trends in the Treatment of Posttraumatic Stress Disorder in Traditional Chinese Medicine. The Korean Society of Oriental Neuropsychiatry. 2016;27(3):197-206.

[90] Kim Keun-wu. Korean Medicine Clinical Practice Guideline for Anxiety Disorder. Guideline center for Korean Medicine. . Korean Society of Oriental Neuropsychiatry; 2017. 1-57p.

[91] From Soyo-san(Siho, danggwi, china root, baekchul, paeonia japonica, menthe arvensis L., licorice root, geinseng)exclude ginseng, and add 7 herbal medicine(coptis chinesis, pinellia ternata, chenpi, yonggol, ostrea gigas, daehwang jujung, acorus gramineus).

[92] State of being immobile with fear and state of low-arousal are both understood to be caused by excessive hyperactivity. Hence, it can be interpreted as lack of internal energy against the trauma, and as weakness instead of hyperarousal. Therefore, prescription for weakness among the clinical guideline for anxiety disorder was propose.

[93]

[94] Kim Jong-Woo. Korean Medicine Clinical Practice Guideline for Depression. Institute of Oriental Medicine. Korean Soceity of Oriental Neuropsychiatry; 2016. 1-10p.

[95] Jung Seon-Yong. Korean Medicine Clinical Practice Guideline for Hwa-Byung. Korean Meidicine

Clinical Practice Guideline R&D Group. Korean Soceity of Oriental Neuropsychiatry; 2016. 49p.
[96] Kim Bo-Kyung. Korean Medicine Clinical Practice Guideline for Insomnia. Korean Meidicine Clinical Practice Guideline R&D Group. Korean Soceity of Oriental Neuropsychiatry; 2017. 1-50p.
[97] Kim Jin-Sung. Korean Medicine Clinical Practice Guideline for Functional Dyspesisa. Korean Meidicine Clinical Practice Guideline R&D Group. The Soceity of Internal Korean MEdicine; 2020. 3-6p.
[98] Nam Dong-Hyun. Korean Medicine Clinical Practice Guideline for Chronic fatigue. Korean Meidicine Clinical Practice Guideline R&D Group. The Society of Korean Medicine Diagnostics, The Soceity of Saang Constitutional Medicine; 2020. 3-6p.
[99] Lee Sang-Kwan. Korean Medicine Clinical Practice Guideline for Migraine. Korean Meidicine Clinical Practice Guideline R&D Group. The Society of Strok on Korean Medicine; 2017. 1-28p.
[100] Lee Eu-Joo. Korean Medicine Clinical Practice Guideline for Scotodinia(Dizziness). Korean Meidicine Clinical Practice Guideline R&D Group. The Soceity of Saang Constitutional Medicine; 2021. 3-6p.
[101] Lee Jae-Hui, Shin Hyun-Soo, Kim Dong-Hyun, Cho Chang-Hwan, Lim Seung-Min, Ahn Jung-Jo, Cho Hyung-Kyung, Kim Yun-Sik, Seol In-Chan, Yoo Ho-Ryong. Statistical Study in 70 Cases for Dizziness Patients on the Effect of Jaeumgeonbi-tang Gamibang. Korean Journal of Oriental Physiology & Pathology. 2010 Feb;24(1):171-6.
[102] NEO Handbook Editing Committee. NEO Intern Handbook 2nd Edition. Paju: Koonja Publication Group; 2017. 661-75p.
[103] Church D, Feinstein D. The manual stimulation of acupuncture points in the treatment of post-traumatic stress disorder: A review of clinical emotional freedom techniques. Medica l acupuncture. 2017 Aug 1;29(4):194-205.
[104] Feinstein D. Energy psychology in disaster relief. Traumatology. 2008 Mar;14(1):127-39.
[105] Emotional Freedom Technique Volume 4. Response to Large scale Disaster. Seoul:Medibook;2020. pp345-367.
[106] Flint, GA, Lammers W, Mitnick DG. Emotional Freedom Techniques: A safe treatment inte rvention for many trauma based issues. Journal of aggression, maltreatment & trauma. 20 06;12(1-2):125-150.
[107] Mattheß C, Farrell D, Mattheß M, Bumke P, Sodemann U, Mattheß H. The therapeutic valu e of trauma stabilisation in the treatment of post-traumatic stress disorder–a Southeast A sian study. Asian journal of psychiatry. 2019;41:45-49.
[108] Zehetmair C, Nagy E, Leetz C, Cranz A, Kindermann D, Reddemann L, Nikendei C. Self-P ractice of Stabilizing and Guided Imagery Techniques for Traumatized Refugees via Digital Audio Files: Qualitative Study. Journal of medical Internet research. 2020;22(9):e17906.
[109] Zehetmair C, Kaufmann C, Tegeler I, Kindermann, D, Junne F, Zipfel S, Herpertz SC, Herz og W, Nikendei C. Psychotherapeutic Group Intervention for Traumatized Male Refugees U sing Imaginative Stabilization Techniques—A Pilot Study in a German Reception Center. Fr ontiers in psychiatry. 2018;9:533.
[110] Kabat-Zinn J, Hanh TN. Full catastrophe living: Using the wisdom of your body and mind to face stress, pain, and illness. NY; A Division of Random House, Inc.: 2009.

[111] Reddemann, L. Imagination als heilsame Kraft. Klett-Cotta, Stuttgart; 2005.

[112] Van der Hart O, Brown P, Van der Kolk BA. Pierre Janet's treatment of post-traumatic stress. Journal of traumatic stress. 1989 Oct 1;2(4):379-95.

[113] Disaster and Mental Health Committee. Disaster and Mental Health. Seoul: Hakjisa; 2015. 268-271p.

[114] Haskell L. First stage trauma treatment: A guide for mental health professionals working with women. Toronto, ON: Centre for Addiction and Mental Health; 2003. 45p.

[115] Baranowsky A, Gentry JE. Trauma practice: Tools for stabilization and recovery. Boston, Massachusetts: Hogrefe Publishing; 2014.

[116] Fisher J. The work of stabilization in trauma treatment. Boston, Massachusetts: Trauma Center Lecture Series; 1999.

[117] Hisli SN, Yilmaz B, Batigun A. Psychoeducation for children and adults after the Marmara earthquake: an evaluation study. Traumatology. 2011;17(1):41-49.

[118] Fisher J. The work of stabilization in trauma treatment. Boston, Massachusetts: Trauma Center Lecture Series; 1999.

[119] Fukuchi N. Psychoeducation for children in a psychiatric ward in the immediate aftermath of the 2011 earthquake and tsunami in Japan. Intervention. 2020;18(1):85.

[120] Mughairbi FA, Abdulaziz AA, Hamid A. Effects of psychoeducation and stress coping techniques on posttraumatic stress disorder symptoms. Psychological reports. 2020;123(3):710-724.

[121] College of Oriental Medicine, Neuropsychiatry Division, Textbook Compilation Committee Edition. Oriental Neuropsychiatry Science. Seoul:Jibmundang;2014. 33-7p.

[122] Lee Gyung-Woo. Huangjenaegyeongsomun 上. Seoul:Yeogang Publishing Group;2007. 589-92p.

123 Kim Ha-Na, Kim Kyung-Ok. Statistical Considerations of Pathological Symptoms Derived from Chiljeong. The Korean Society of Oriental Neuropsychiatry. 2015;26(1):11-22.

[124] Lee Go-Eun, Yoo Young-Soo, Kang Hyung-Won. Study of Pathologic Mechanism Related Chiljeong-Based on a Literature Review of Donguibogam. The Korean Society of Oriental Neuropsychiatry. 2014;25(1):85-108.

[125] Kwon CY, Kwak HY, Kim JW. Using Mind-Body Modalities via Telemedicine during the COVID-19 Crisis: Cases in the Republic of Korea. Int J Environ Res Public Health. 2020 Jun 22;17(12):4477.

[126] Luders E, Toga AW, Lepore N, Gaser C. The underlying anatomical correlates of long-term meditation: larger hippocampal and frontal volumes of gray matter. Neuroimage. 2009 Apr 15;45(3):672-8.

[127] Gotink RA, Vernooij MW, Ikram MA, Niessen WJ, Krestin GP, Hofman A, Tiemeier H, Hunink MM. Meditation and yoga practice are associated with smaller right amygdala volume: the Rotterdam study. Brain imaging and behavior. 2018 Dec 1;12(6):1631-9.

[128] Chosun-Ilbo. 4 Factors that led to "miraculous survivor" at the dark cave of Thailand [Cited: March 29, 2021] Available:https://www.chosun.com/site/data/html_dir/2018/07/13/2018071301090.html

[129] Atti SK, Alfalasi RB, Mahon SE, Sarin RR, Voskanyan A, Molloy MS, Ciottone GR. Mindfuln

ess in Disaster Response. Prehospital and disaster medicine. 2017 Apr;32(S1):S181.
[130] Yoshimura M, Kurokawa E, Noda T, Tanaka Y, Hineno K, Kawai Y, Dillbeck MC. Disaster relief for the Japanese earthquake–tsunami of 2011: Stress reduction through the transcen dental meditation® technique. Psychological reports. 2015 Aug;117(1):206-16.
[131] Kashiwazaki Y, Takebayashi Y, Murakami M. Relationships between radiation risk percepti on and health anxiety, and contribution of mindfulness to alleviating psychological distres s after the Fukushima accident: Cross-sectional study using a path model. PloS one. 2020 Jul 6;15(7):e0235517.
[132] Zaccaro A, Piarulli A, Laurino M, Garbella E, Menicucci D, Neri B, Gemignani A. How Bre ath-Control Can Change Your Life: A Systematic Review on Psycho-Physiological Correlat es of Slow Breathing. Front Hum Neurosci. 2018 Sep 7;12:353.
[133] Carter KS, Carter R 3rd. Breath-based meditation: A mechanism to restore the physiologi cal and cognitive reserves for optimal human performance. World J Clin Cases. 2016 Apr 16;4(4):99-102.
[134] Fisher J. The work of stabilization in trauma treatment. Boston, Massachusetts: Trauma C enter Lecture Series; 1999.
[135] Yang YK. Hwangjaenaekyung Haeseok (Somoon). Seoul:Seongbosa;1980. 35, 108-12, 304, 6 64p.
[136] Pat O. Kekuni M, Clare P. Trauma and the Body: A sensorimotor approach to psychother apy. New York: WW Norton & Company; 2006. 27,69p.
[137] Park Jong-Min, Lee Go-Eun, Seo Joo-Hee, Bae Dal-Bit, Choi Sung-Yeol, Kang Hyung-Won. Case Study on the effect of Maeum Chaeng-gim meditation and LiGyeungByunQi therapy to treat PTSD. The Korean Society of Oriental Neuropsychiatry. 2014:25(1):73-84.
[138] Korean M&L Psychological Therapy Resaerch Center. M&L Psychological Therapy Pro-skill training course compilations for Professionals.
[139] Lee Hui-Jung, Kang Da-Hyun, Lim Myung-Ah, Kim Doo-ri, & Seo Joo-Hee. Case Reports: Effect of M&L Psychotherapy and Korean Medical Treatment on Psychiatric & Physical Symptoms of North Korean Defectors. The Korean Society of Oriental Neuropsychiatry. 2016:2 7(3), 185-196.
[140] Kunii Y, Suzuki Y, Shiga T, et al. Severe Psychological Distress of Evacuees in Evacuatio n Zone Caused by the Fukushima Daiichi Nuclear Power Plant Accident: The Fukushima Health Management Survey. PLoS One. 2016;11(7):e0158821
[141] Uemura M, Ohira T, Yasumura S, Otsuru A, Maeda M, Harigane M, Horikoshi N, Suzuki Y, Yabe H, Takahashi H, Nagai M, Nakano H, Zhang W, Hirosaki M, Abe M; Fukushima H ealth Management Survey Group. Association between psychological distress and dietary i ntake among evacuees after the Great East Japan Earthquake in a cross-sectional study: t he Fukushima Health Management Survey. BMJ Open. 2016 Jul 5;6(7):e011534.
[142] Takahashi T, Goto M, Yoshida H, Sumino H, Matsui H. Infectious Diseases after the 2011 Great East Japan Earthquake. J Exp Clin Med. 2012;4(1):20-23.
[143] Inoue T, Nakao A, Kuboyama K, Hashimoto A, Masutani M, Ueda T, Kotani J. Gastrointesti nal symptoms and food/nutrition concerns after the great East Japan earthquake in Marc h 2011: survey of evacuees in a temporary shelter. Prehosp Disaster Med. 2014 Jun;29(3):3

03-6.

[144] Yabuki S, Ouchi K, Kikuchi S, Konno S. Pain, quality of life and activity in aged evacuees living in temporary housing after the Great East Japan earthquake of 11 March 2011: a cross-sectional study in Minamisoma City, Fukushima prefecture. BMC Musculoskelet Disord. 2015;16:246.

[145] Ueda S, Hanzawa K, Shibata M. One-year overview of deep vein thrombosis prevalence in the ishinomaki area since the great East Japan earthquake. Ann Vasc Dis. 2014;7(4):365-368.

[146] Sawano T, Nishikawa Y, Ozaki A, et al. Premature death associated with long-term evacuation among a vulnerable population after the Fukushima nuclear disaster: A case report. Medicine (Baltimore). 2019;98(27):e16162.

[147] "What happens to medical care in the event of a disaster? What can be doen with acupuncture and moxibustion in the field: Interview with Dr. MAsataka Miwa" Health and Beuty Information Media "Carecle". May 2, 2018 [Cited: Jan 7, 2021] Available: https://media.carecle.com/articles/n5Ivb#i31602

[148] Korean Society of Oriental Medicine and Psychiatry, Hwa-Byung R&D Center. 100 Q&As about Hwa-byung. Seoul: Jipmundang;2013. p.257-8.

[149] Korean Society of Oriental Medicine and Psychiatry, Hwa-Byung R&D Center. 100 Q&As about Hwa-byung. Seoul: Jipmundang;2013. p.259-60, 261-3, 274-5.

[150] Takayama S, Kamiya T, Watanabe M, Hirano A, Matsuda A, Monma Y, Numata T, Kusuyama H, Yaegashi N. Report on disaster medical operations with acupuncture/massage therapy after the great East Japan earthquake. Integr Med Insights. 2012;7:1-5.

[151] Song HJ, Seo HJ, Lee H, Son H, Choi SM, Lee S. Effect of self-acupressure for symptom management: a systematic review. Complementary therapies in medicine. 2015 Feb 1;23(1):68-78.

[152] Kwon CY, Lee B. Acupuncture or acupressure on yintang (EX-HN 3) for anxiety: a preliminary review. Medical acupuncture. 2018 Apr 1;30(2):73-9.

[153] Waits A, Tang YR, Cheng HM, Tai CJ, Chien LY. Acupressure effect on sleep quality: a systematic review and meta-analysis. Sleep medicine reviews. 2018 Feb 1;37:24-34.

[154] Zhao ZH, Zhou Y, Li WH, Tang ZH, Xia TW. Auricular acupressure in patients with hypertension and insomnia: a systematic review and meta-analysis. Evidence-Based Complementary and Alternative Medicine. 2020 Jun 17;2020.

[155] Jang M, Park H. The Effects of Auricular Acupressure on Sleep Disorder in The Elderly: A Systematic Review and Meta-analysis. Journal of the Korea Academia-Industrial cooperation Society. 2020;21(6):116-26.

[156] Gong Z, Yu W. Effectiveness and Characteristics of Acupressure for Elderly with Insomnia: A Systematical Review. Global Journal of Intellectual & Developmental Disabilities. 2017;2(2):58-66.

[157] You E, Kim D, Harris R, D'Alonzo K. Effects of auricular acupressure on pain management: A systematic review. Pain Management Nursing. 2019 Feb 1;20(1):17-24.

[158] Liu M, Tong Y, Chai L, Chen S, Xue Z, Chen Y, Li X. Effects of auricular point acupressure on pain relief: a systematic review. Pain Management Nursing. 2020 Sep 17.

[159] Kwon CY, Lee B. Clinical effects of acupressure on neck pain syndrome (nakchim): a systematic review. Integrative medicine research. 2018 Sep 1;7(3):219-30.

[160] Chen Y, Xiang XY, Chin KH, Gao J, Wu J, Lao L, Chen H. Acupressure for labor pain management: a systematic review and meta-analysis of randomized controlled trials. Acupuncture in Medicine. 2020 Aug 18:0964528420946044.

[161] Zhong Q, Wang D, Bai YM, Du SZ, Song YL, Zhu J. Effectiveness of auricular acupressure for acute postoperative pain after surgery: a systematic review and meta-analysis. Chinese journal of integrative medicine. 2019 Mar 1;25(3):225-32.

[162] Monson E, Arney D, Benham B, Bird R, Elias E, Linden K, McCord K, Miller C, Miller T, Ritter L, Waggy D. Beyond pills: Acupressure impact on self-rated pain and anxiety scores. The Journal of Alternative and Complementary Medicine. 2019 May 1;25(5):517-21.

[163] Ghiasi A, Keramat A, Mollaahmadi L, Hashemzadeah M, Bagheri L. The effect of acupressure at the Sameumgyo (SP6) point on relief of primary dysmenorrhea: a systematic review of clinical trials. The Iranian Journal of Obstetrics, Gynecology and Infertility. 2017;19(40):55-68.

[164] Armour M, Ee CC, Hao J, Wilson TM, Yao SS, Smith CA. Acupuncture and acupressure for premenstrual syndrome. Cochrane Database of Systematic Reviews. 2018(8).

[165] Miao J, Liu X, Wu C, Kong H, Xie W, Liu K. Effects of acupressure on chemotherapy-induced nausea and vomiting-a systematic review with meta-analyses and trial sequential analysis of randomized controlled trials. International journal of nursing studies. 2017 May 1;70:27-37.

[166] Huang CF, Guo SE, Chou FH. Auricular acupressure for overweight and obese individuals: a systematic review and meta-analysis. Medicine. 2019 Jun;98(26).

[167] Gao H, Zhang L, Liu J. Auricular acupressure for myopia in children and adolescents: A systematic review. Complementary therapies in clinical practice. 2020 Feb 1;38:101067.

[168] Liu Y, Tang WP, Gong S, Chan CW. A systematic review and meta-analysis of acupressure for postoperative gastrointestinal symptoms among abdominal surgery patients. The American Journal of Chinese Medicine. 2017 Aug 22;45(06):1127-45.

[169] Zheng SH, Yan M, Field T, Xu X. Beneficial effects of auricular acupressure on preventing constipation in breast cancer patients undergoing chemotherapy: evidence from systematic review and meta-analysis. Frontiers of Nursing. 2018 Oct 25;5(3):227-34.

[170] Sahan S. The Effect of Acupressure on Constipation: A Systematical Review. Int J Clin Med Info. 2020;3(1):28-34.

[171] Wang PM, Hsu CW, Liu CT, Lai TY, Tzeng FL, Huang CF. Effect of acupressure on constipation in patients with advanced cancer. Supportive Care in Cancer. 2019 Sep 1;27(9):3473-8.

[172] Aval SB, Ravanshad Y, Azarfar A, Mehrad-Majd H, Torabi S, Ravanshad S. A systematic review and meta-analysis of using acupuncture and acupressure for uremic pruritus. Iranian journal of kidney diseases. 2018 Mar 1;12(2):78.

[173] Liang Y, Lenon GB, Yang AW. Acupressure for respiratory allergic diseases: a systematic review of randomised controlled trials. Acupuncture in Medicine. 2017 Dec;35(6):413-20.

[174] Harris ML, Titler MG, Struble LM. Acupuncture and acupressure for dementia behavioral

and psychological symptoms: a scoping review. Western journal of nursing research. 2020 Oct;42(10):867-80.

[175] Almeida Sr JR, Almeida Sr MC, Cunha Sr ME, Silva Sr AG. The effectiveness of a self-administered acupressure intervention in caregivers of Alzheimer's disease patients: A systematic review: Psychosocial factors and environmental design/Living with dementia and quality of life. Alzheimer's & Dementia. 2020 Dec;16:e037536.

[176] Hmwe NT, Browne G, Mollart L, Allanson V, Chan SW. An integrative review of acupressure interventions for older people: A focus on sleep quality, depression, anxiety, and agitation. International Journal of Geriatric Psychiatry. 2019 Mar;34(3):381-96.

[177] Takayama S, Kamiya T, Watanabe M, Hirano A, Matsuda A, Monma Y, Numata T, Kusuyama H, Yaegashi N. Report on disaster medical operations with acupuncture/massage therapy after the great East Japan earthquake. Integr Med Insights. 2012;7:1-5.

[178] Takayama S, Kamiya T, Watanabe M, Hirano A, Matsuda A, Monma Y, Numata T, Kusuyama H, Yaegashi N. Report on disaster medical operations with acupuncture/massage therapy after the great East Japan earthquake. Integr Med Insights. 2012;7:1-5.

[179] Church D, Stapleton P, Yang A, Gallo F. Is tapping on acupuncture points an active ingredient in Emotional Freedom Techniques? A systematic review and meta-analysis of comparative studies. The Journal of nervous and mental disease. 2018 Oct 1;206(10):783-93.

[180] Korean Medicine onvergence Research Information Center. Standard Acupuncture DB.[Cited: Jan 12, 2021] Available:https://www.kmcric.com/database/acupoint

[181] Takenouchi Mitsushi. Easy to lear Acupressure Vitamin100 with pictures. Choon-Ang Life Cycle (Seoul):2008. pp31.

[182] Korean Acupuncture Society Textbook Compilation Committee. Acupuncture medicine. Chapter2 Detailed Episodes. Seoul(Korean Medicine);2020.

[183] Korean Acupuncture Society Textbook Compilation Committee. Acupuncture medicine. Seoul(Korean Medicine):2020. p715

[184] The society of korean medicine rehabilitation. Oriental rehabilitation medicine. 3rd ed. Seoul: Koonja
publishing company; 2011. p.306.

[185] Hong WS, Jing-Jiao-Hung-Di-Nei-Jing, Seoul:Research of Oriental Medicine. 1985:198.

[186] Kim HT. A Documentational Study of Doinqigong in The Oriental Medicine Classics. Journal of Korean Medical classics. 2009:22(3):9-18.

[187] Lee EJ, Park IS, Oh MS. Effects of the Daoyin and Corrective Exercise Program on Musculoskeletal Pain -Based on the Acute Sprain Patients due to Traffic Accidents-. Journal of Korean Medicine Rehabilitation. 2011;21(4):205-17.

[188] Youn HM, Kim MY, Kim YS, Lim JS. Effects of the Doin Gigong Exercise on the Shoulder-Arm Pain in Women. The Journal of Korean Acupuncture & Moxibustion Society, 2005;22(1):243-8.

[189] Lee JW. Effect of exercise treatment through Dong Bang Hwal Bup and dao in yang seng gong in degenerative knee arthritis. Official Journal of the Korean Association of Certified Exercise Professionals. 2006;8(2):129-36.

[190] Choi BS, Lee EJ, Li YC, Lee JM, Kim ES, Song GC, Jung IC, Oh MS. Effect of The Daoyin

Exercise Therapy Combined with Complex Korean Medicine Treatment on Pain and Function Improvement of Low Back Pain Patients : A Retrospective Observational Study. J Physiol & Pathol Korean Med 2018;32(1):88-97.

[191] Hwang Eu-Hyung, Yang Chang-Sub. Medical Engineering. Pusan, Pusan National University, Publishing Department, 2013, pp21-99,

[192] Koo Dae-Keun, Park Jong-Hakr, Han Chang-Hee, Um Ki-Young, Kang Soo-Won. Ko Sik Hwa Tao Geum Hee**.** Seoul, Bal-Geun-Bit Publishing group, 2010, pp 12-20.

[193] Hwang EH, Kwon YK, Heo KH, Cho HW, Lee HY, Sung WY. Comparative Review of Qigong and Daoyin as a Therapeutic Exercise of Traditional Korean Medicine. Korean J. Oriental Physiology & Pathology 2013;27(5):594-601,

[194] National Center of Dementia. 2018 Guide to Dementia. National Center of Dementia.. Ministy of Health. 2018:19-31p

[195] Jebyungwonhuron, Ascetic Training Method. Movements resembling Dumyunpunghu (頭面風候) 令髮不白．以手復捋頭五，通脈也

[196] Jebyungwonhuron Ascetic Training Method. Movements resembling Heorohu (虛勞候) 兩手抱兩乳，急努，前後振搖，極勢二七

[197] Jebyungwonhuron Ascetic Training Method. Movements resembling Heorohu (虛勞候) 兩手長舒，掌相向腦項之後

[198] Prakhinkit S, Suppapitiporn S, Tanaka H, Suksom D. Effects of Buddhism walking meditation on depression, functional fitness, and endothelium-dependent vasodilation in depressed elderly. J Altern Complement Med. 2014 May;20(5):411-6.

[199] Prins A., Bovin MJ., Kimerling R., Kaloupek DG., Marx BP., Pless Kaiser A., Schnurr PP. The Primary Care PTSD Screen for DSM-5 (PC-PTSD-5). National Center for PTSD. 2015.(Available from https://www.ptsd.va.gov)

[200] Jung YE, Kim D, Kim WH, Roh D, Chae JH, Park JE. A Brief Screening Tool for PTSD: Validation of the Korean Version of the Primary Care PTSD Screen for DSM-5(K-PC-PTSD-5). Journal of Korean Medical Science 2018;33(52):e338.

[201] Spitzer RL., Kroenke K., Williams JBW. Validation and utility of a self-report version of PRIME-MD: the PHQ primary care study. The Journal of the American Medical Association. 1999:282(18);1737-1744.

[202] Park Joo-Eon, Kim Won-Hyung, Noh Dae-Young, Won Seong-Doo, Kim Ha-Kyung, Kang Seok-Hoon, Hong Na-Rae, Park Seong-Yong, Kim Dae-Ho, Chae Jeong-Ho. Disaster Trauma Mental Health Assessment Workbook. 2016.

[203] Spitzer RL., Kroenke K., Williams JB., Lowe B. A brief measure for assessing generalized anxiety disorder: the GAD-7. Archives of internal medicine. 2006;166(10):1092-1097.

[204] Kroenke K., Spitzer RL., Williams JB. The PHQ–15: validity of a new measure for evaluating the severity of somatic symptoms. Psychosomatic Medicine. 2002;64:258–266.

[205] Han C, Pae CU, Patkar AA, Masand PS, Kim KW, Joe SH, Jung IK. Psychometric properties of the Patient Health Questionnaire-15(PHQ-15) for measuring the somatic symptoms of psychiatric outpatients. Psychosomatics. 2009;50(6):580-585.

[206] Dube P, Kurt K, Bair MJ, Theobald D, Williams LS. The p4 screener: evaluation of a brief measure for assessing potential suicide risk in 2 randomized effectiveness trials of primar

y care and oncology patients. Primary care companion to the Journal of clinical psychiatry. 2010;12(6).

[207] Park Joo-Eon, Kim Won-Hyung, Noh Dae-Young, Won Seong-Doo, Kim Ha-Kyung, Kang Seok-Hoon, Hong Na-Rae, Park Seong-Yong, Kim Dae-Ho, Chae Jeong-Ho. Disaster Trauma Mental Health Assessment Workbook. 2016.
