# Supplementary material for: Development of a Manual for Disaster Medical Support Using Korean Medicine for Disaster Survivors
Source: J Integr Complement Med. 2023 Jun 6;29(6-7):395–407. doi: 10.1089/jicm.2022.0561 (PMC10280179; doi:10.1089/jicm.2022.0561)
Supplement: Supplemental data [file Supp_DataS2.pdf]

재난트라우마의 한의사 진료 매뉴얼(안)

김상호
대구한의대 부속포항한방병원 한방신경정신과

권찬영
동의대학교 한방병원 한방신경정신과

서주희
국립중앙의료원 한방진료부 한방신경정신과

CONTENTS
목차

머리말 4

Ⅰ 매뉴얼 개요

1. 필요성 8
2. 목적 8
3. 적용 대상 9
4. 재난 시 생존자의 반응 9
5. 국내 재난심리지원 체계 17

Ⅱ 진료 프로토콜

1. 진료흐름도 22
2. 병력청취 및 문진 매뉴얼 23
3. 단계별 진료 프로토콜 29
4. 증상별 진료 프로토콜 34
5. 심리적 응급처치 36
6. 기타 고려사항 40

본 매뉴얼은 정부(과학기술정보통신부)의 재원으로 한국연구재단의 지원을 받아 수행된 연구 성과임(No.2019R1G1A100591513).

Ⅲ 치료법 소개

1. 이침치료	44
2. 침치료	52
3. 한약치료	56
4. 감정자유기법	63
5. 안정화기법	70
6. 자가 관리법	85

Ⅳ 부록

머리말

포항 진료실에서
연구책임자
김상호

생년월일만큼 제게 익숙한 그리고 잊지 못하는 숫자가 있습니다. 20171115... 2017년 11월 15일 14시 29분에 포항에서 규모 5.5의 지진이 발생했습니다. 포항지진이 발생한 지가 엊그제 같은데 어느덧 4년이 넘는 시간이 지났습니다. 처음 연구재단 연구과제를 준비하다가 신문 기사를 통해 지진이 발생한 지 1년이 훨씬 넘었는데도 아직도 많은 이재민이 대피소에서 지내고 있다는 소식을 접했습니다. 이재민들은 여전히 지진 트라우마와 장기 대피소 생활로 인한 불면, 우울, 통증 등을 겪고 있었습니다. 이를 계기로 비로소 재난트라우마에 관심을 가지게 되었고 연구를 진행하게 되었습니다. 실제 진료팀을 꾸려 흥해 보건지소 재난심리지원센터의 협조하에 지진 이재민들을 대상으로 이침치료를 활용한 의료지원 활동을 펼쳤습니다

재난 심리지원에는 주로 심리적 중재가 활용됩니다. 하지만 재난 생존자 (재난의학에서는 피해자가 아니라 생존자라는 단어를 주로 사용합니다.)는 심리적 증상뿐만 아니라, 불면, 통증, 피로 등 다양한 신체 증상을 호소합니다. 하지만 재난 후 대규모 재난 생존자가 발생하면 제한된 인적·물적 의료자원으로는 즉각적인 재난 심리지원을 제공하기가 어렵습니다. 그리고 장기적인 심리지원은 심리지원 제공자의 번아웃 증후군을 유발할 수 있습니다. 그러므로 급박한 재난 현장에서 활용 가능한 효과적이며 안전하고 경제적인 비약물·비심리적 중재법이 필요합니다.

이침 치료는 부작용이 적고 시술이 간편한 비약물 치료법으로 이미 전 세계 재난 현장에서 활용되고 있는 대표적 한의 치료법입니다. 또한 한의사는 한약, 침과 같은 전통적 한의학치료법뿐 아니라 명상, 감정자유기법, 안정화기법 등 다양한 심신의학 중재를 통해 재난 현장에서 따뜻한 실질적인 도움을 줄 수 있습니다. 또한 지압이나 도인운동법 등을 활용하여 재난 생존자들의 자가 관리를 적극적으로 도울 수 있습니다.

안타깝게도 현재 국내 재난 현장에서 한의 치료는 개별적인 의료봉사 형태로만 제공되고 있을 뿐 국가 지원체계 내에서 공식적으로 활용되고 있지 않습니다. 또한 재난 현장에서 적용될 수 있는 한의 치료의 근거에 대한 정리 및 체계화가 미흡하며, 한의사가 재난 현장에서 어떻게 의료지원을 시행할지에 대한 표준화된 매뉴얼도 없습니다. 이에 본 연구팀은 매뉴얼을 개발하였습니다.

본 매뉴얼이 재난현장에서 한의사의 진료를 돕는 가이드북이자, 향후 국가 재난트라우마 대응 및 공공의료영역에서 한의사가 기여할 수 있는 소중한 밑거름이 되기를 희망합니다. 함께 매뉴얼 개발에 힘써주신 권찬영 교수님, 서주희 과장님, 그리고 매뉴얼의 방향을 지도해 주신 김종우 교수님께 감사드립니다. 검토해 주신 많은 자문위원분들에게도 진심으로 감사의 말씀을 드리며, 사랑하는 저희 가족과 하나님께 감사드립니다.

검토위원 대한한방신경정신과학회 인증평가위원회

김종우	위원장 / 경희대학교 한의과대학 한방신경정신과 주임교수 / 한의학정신건강센터 센터장
서진우	위원 / 상지대학교 한의과대학 한방신경정신과 교수
서효원	간사 / 경희대학교 한의학과 학술연구교수 / 한방신경정신과 전문의
임정화	위원 /부산대학교 한의학전문대학원 한방신경정신과 교수
정진형	위원 /장덕한방병원 진료과장 / 한방신경정신과 전문의
최성열	위원 /가천대학교 한의과대학 한방신경정신과 교수

자문위원

권호인	전주대학교 상담심리학과 교수
김건형	부산대학교 한의학전문대학원 침구의학과 교수
김동수	동신대학교 한의과대학 예방의학교실 교수
김륜재	남이경희한의원장
김성태	대구한의대학교 한의과대학 사상체질의학과 교수
김윤나	경희대학교 한방병원 한방신경정신과 임상교수
김은정	동국대학교 한의과대학 침구의학과 교수
박종훈	안산자생한방병원장 / 한방신경정신과 전문의
서영호	서영호한의원장 / 한방내과 전문의
신차선	NGO 사람들에게 평화를 심리사회지원 교육원. 대표 / 심리치료 박사
한선용	금손한의원장
이보람	한국한의학연구원 선임연구원
이승환	솔솔한의원장 / 한방신경정신과 전문의
이윤규	대구한의대학교 한의과대학 침구의학과 교수
이정환	혜민서한의원장 / 한국EFT협회 & 사암침법학회장
이진희	다이룸한의원장 / EFT 코리아 마스터 트레이너
임정태	원광대학교 연구교수 / 한의학박사
정선용	경희대학교 한의과대학 한방신경정신과 교수
조성훈	경희대학교 한의과대학 한방신경정신과 교수

편집위원

김다운	대구한의대학교 부속포항한방병원 한방신경정신과 / 전문수련의
이도은	국립의료원 한방진료부 한방신경정신과 / 전문수련의

본 매뉴얼의 구성

본 매뉴얼은 I. 개요, II. 진료 프로토콜, III. 치료법, IV. 부록으로 구성되었다.
먼저 I. 개요에서 매뉴얼의 1. 필요성, 2. 목적, 3. 대상을 제시하여 본 매뉴얼의 범위를 명확히 하였다. 또한 재난 생존자의 반응을 이해하기 위해 4. 재난 시 생존자의 반응을 소개하였고 마지막으로 5. 국내 재난심리지원 체계에 대해 소개하였다.

다음으로 II. 진료 프로토콜에서는 먼저 전체 진료 과정을 한눈에 파악할 수 있는 1. 진료 흐름도를 제시하였고, 2. 병력청취 및 문진 매뉴얼, 3. 단계별 진료 프로토콜, 4. 증상별 진료 프로토콜, 5. 심리적 응급처치, 6. 기타 고려 사항이 제시되었다. 병력청취 및 문진 매뉴얼에서 재난 현장 진료를 위해 구체적인 접근 방법 및 평가도구를 제시하였다. 다음으로 재난 단계에 따라 다른 반응이 나타나므로 재난 단계별(급성기/아급성기/만성기) 접근 방법을 제시하였다. 또한 현장에서 실질적인 대처에 용이하도록 재난 생존자의 주호소 증상을 심리적 증상(과긴장/공포/우울/분노)과 신체적 증상(불면/소화불량/탈진/두통·어지럼증/통증)으로 구분한 증상별 프로토콜을 제시하였다. 증상별 프로토콜에는 재난 생존자의 자가 관리를 돕는 경혈지압도 제시하였다. 마지막으로 재난구호 현장에서 진료에 앞서 우선 시행해야 할 심리적 응급처치와 기타 재난의료지원 시 고려 사항을 설명하였다.

III. 치료법에서는 진찰 시 활용되는 개별 치료 및 관리법에 대해 구체적으로 제시하여 진료에 참고하도록 하였다. 치료법으로는 이침, 한약, 침치료, 감정자유기법 및 안정화기법을 제시하였고, 증상별 대처법, 경혈지압, 도인운동, 명상과 같은 자가 관리법의 구체적인 교육 자료를 제시하였다.

I

매뉴얼 개요

01 필요성

02 목적

03 적용 대상

04 재난 시 생존자의 반응

05 국내 재난심리지원 체계

Ⅰ. 매뉴얼 개요

1. 필요성

재난은 정기적으로 발생하는 우리 삶의 일부이다. 전 세계에서 기후변화로 인한 홍수, 태풍, 가뭄, 산불 및 지진과 같은 자연재난과 테러, 사고, 전쟁, 핵 재난 같은 사회재난이 지속적으로 발생해왔고 현재 코로나바이러스 감염증-19(코로나19)로 인한 감염재난이 지속 중이다. 재난은 짧은 시간 동안 광범위한 지역에 심각하고 다양한 피해를 발생시키며 복구와 회복이 쉽지 않다. 특히 재난은 우울, 외상후스트레스장애 등 재난 생존자의 정신건강에도 악영향을 미치므로 국가 차원의 체계적 재난심리지원이 필요하다.

현재 재난심리지원은 인지행동치료나 노출치료, 안구운동 민감소실 및 재처리(Eye Movement Desensitization and Reprocessing, EMDR), 안정화기법 등 심리적 중재가 주로 활용된다. 하지만 생존자는 심리적 증상뿐만 아니라 불면, 통증, 피로 등 다양한 신체증상을 호소한다. 또한 대규모 생존자와 이재민이 발생하면 인적 및 물적 의료자원은 제한적이므로 즉각적이고 효율적인 심리지원이 어렵다. 그리고 장기 심리지원은 제공자의 소진(번아웃, burnout)을 유발할 수 있다. 그러므로 재난현장에서 기존 심리지원을 보완하는 신속하고 효과적이며 장기 지원이 가능한 보완 중재법이 필요하다.

이침치료는 한의학의 대표적인 비약물요법으로 시술이 간단하고 부작용이 적어 재난 진료 현장에서 활용하기에 적합하며 그 효과에 대한 많은 근거들이 있다. 한약이나 침치료 역시 다양한 신체/정신증상에 적용 가능하며 정신과 약물치료에 비해 부작용이 적고 의존성이 없다. 최근 한의 신의료기술로 등재된 감정자유기법(Emotional Freedom Technique, EFT) 또한 재난 현장에서 효과적으로 활용 가능하다.

재난 의료지원에는 가용한 인적, 물적 자원을 총동원하여 대처해야 함에도 불구하고 현재까지 한의사 인력을 활용한 한의 진료지원은 개별 의료봉사 형태에 국한되어 국가 재난지원체계 내에 포함되지 못한 상황이다. 또한 현재까지 재난트라우마 현장에서 한의사가 활용 가능한 진료 매뉴얼과 한의진료를 적용할 수 있는 협진 매뉴얼은 없다. 이에 본 연구팀은 재난트라우마에 대한 한의의료지원 매뉴얼을 개발하였으며, 이 매뉴얼은 한의사 인력이 재난현장에서 체계적이고 적극적인 의료지원을 제공하도록 돕고, 향후 한의진료를 활용한 협진체계 구축에 활용되어 보다 적극적이고 효율적인 국가 재난 트라우마 대응에 기여할 것으로 기대된다.

2. 목적

본 매뉴얼의 목적은 재난 경험 후 발생한 생존자의 심리, 신체 및 행동적 스트레스 반응(이하 재난트라우마 증상)을 완화할 수 있도록 한의사의 진료를 돕는 것이다. 또한 진료와 병용하여 재난 생존자 스스로 트라우마 증상을 조절할 수 있도록 자가 관리법을 교육하는 것이다.

3. 적용 대상

본 매뉴얼은 재난트라우마 증상을 호소하는 생존자(1차 피해자)를 대상으로 하며, 이 외에 재난 생존자의 가족, 친척 및 가까운 지인, 재난 현장에서 활동한 재난업무종사자 및 지원인력(소방관, 경찰관, 구조 대원, 의사, 간호사, 사회복지사, 심리지원요원, 성직자, 공무원 등), 재난 발생 지역사회 주민, 대중매체를 통해 간접적으로 재난 충격을 경험한 시민 등 2차적 트라우마를 호소하는 경우에도 적용할 수 있다.

표 1 매뉴얼 적용 대상

- 재난 경험의 충격으로 재난트라우마 증상을 호소하는 경우
- 재난 생존자, 재난 생존자의 가족, 재난현장 지원인력
- 재난 발생 지역사회 주민 및 대중매체를 통해 간접적으로 재난 충격을 경험한 시민
- 재난 생존자가 안정화기법, 트라우마중심 인지행동치료나 안구운동 민감소실 및 재처리(EMDR) 등의 심리지원 및 항우울제 등 정신과 약물치료를 받았으나 증상이 개선되지 않거나 위의 치료를 선호하지 않는 경우
- 심리지원을 받는 생존자가 한의 치료의 병용을 원할 경우
- 심리지원을 받지 못했고 한의 치료를 원하는 경우
- 재난심리지원센터 및 관련 기관에서 한의 진료를 의뢰하는 경우
- 단, 심각한 과각성 또는 정신병적 증상이 나타나거나 타해나 자해의 위험이 없는 자여야 한다. 이런 경우 정신과 약물치료나 전문 심리치료를 우선 고려한다.

4. 재난 시 생존자의 반응

먼저 효과적인 진료를 위해 재난 시 생존자의 반응과 재난트라우마의 특징적 증상, 단계적 반응을 이해하는 것이 필요하다. 재난 생존자들은 이미 재난심리지원 등을 받고 있는 경우가 많으므로 국가 재난심리지원의 시행과정에 대한 이해도 필요하다.

생명을 위협하는 재난이나 트라우마 사건을 직·간접적으로 경험하면 대부분 생리적·인지적·정서적·행동적 영역에서 다양한 반응이 나타난다. 재난 후 반응은 즉시 나타나거나 얼마 동안 시간이 지난 후 나타날 수도 있다. 하지만 대부분의 생존자들은 이런 반응이 재난 충격에 따른 자연스럽고 정상적인 반응이라는 것을 알지 못하고 자신의 변화를 받아들이지 못한다. 따라서 재난 경험 후 어떤 반응이 나타날 수 있는지 교육하는 것이 필요하다. 이런 급성 스트레스 반응은 재난의 심각성, 개인별 특성(성, 연령, 기존의 정신과적 병력, 과거 트라우마 경험 등) 및 사회문화적 배경에 따라 달라질 수 있다.

1) 급성 스트레스 반응

재난 생존자들에서 흔히 나타나는 신체적, 정서적, 인지적, 행동적, 영적 변화 및 증상은 다음과 같다[1) 2)].

표 2 재난 생존자의 영역별 증상

영 역	증 상
**신체적 변화**	심박수가 빨라지며, 혈압이 올라가고, 숨이 가빠지며, 땀을 흘린다.<br>입술 떨림, 위통, 식욕저하 혹은 식욕항진, 소화불량, 구역, 오한, 불면증 또는 악몽, 수면과다증 및 전신피로, 눈의 피로, 두통, 상복부 통증 및 다른 부위 통증 등을 호소한다.
**정서적 변화**	괴롭고, 끔찍하고, 마음이 상하고, 억울하며, 화나고, 죄책감, 불행감, 안절부절못함,<br>두려움 같은 압도적인 다양한 감정을 느낀다. 자신이 미치지 않을까 두려워한다.<br>소아에서는 과도한 매달림, 공포, 짜증, 자다가 땀을 흘림, 엄지손가락을 빠는 퇴행 행동이 나타날 수 있다.
**인지적 변화**	시야가 좁아지고 생각이 경직된다.<br>멍하고 혼란스러워 집중하기 어렵다.<br>모든 것에 아무 것도 느끼지 못하는 듯한 무감각상태(해리)가 될 수 있다.<br>의사소통하기 어렵고 심하면 사람·시간·장소에 대한 지남력이 손상될 수 있다.<br>사건 관련된 이미지 등이 떠오르고 플래시백을 경험할 수 있다.<br>세상에 대한 믿음이 흔들리는 사람도 많다.<br>나처럼 힘든 사람은 없을 거라는 생각을 한다.
**행동적 변화**	행동이 경직되어 화를 잘 내고 비난할 책임자나 희생양을 찾는다. 의사소통이 어렵고 무력하고 안절부절못한다. 사건 관련된 장소나 사람들을 피하려는 회피 반응이 자주 일어나 사회적 고립 상태가 될 수 있다. 스트레스에 대처하기 위해 알코올, 담배, 마약 및 항불안제, 진통제 등 약물을 과다 복용할 수 있다.
**영적 변화**	재난 사건에서 의미를 찾으려고 노력하는 사람도 있고, 원망하며 희망을 잃어버리는 사람도 있다.

단, 다음의 해리 반응이 지속되면 추후 외상후스트레스장애 발병 위험이 높으므로 정신건강 전문가 진료를 의뢰하는 등 특별히 관리해야 한다[3)].

- 정서적 반응이 없음
- 멍하거나 넋이 나간 듯 주변 인식에 어려움을 겪음
- 현실 세계가 실제같이 느껴지지 않음
- 자신의 몸과 정신이 분리된 것 같고 자기 정체성에 대한 혼란(이인증)
- 중요한 부분을 기억하지 못함

1)The American Psychiatric Association (APA). Coping After Disaster. [Cited Dec 24, 2020] Available:https://www.psychiatry.org/patients-families/coping-after-disaster-trauma#:-:text=Common%20reactions%20in%20adults%20after%20a%20disaster%3A&text=Sadness%2C%20depression%2C%20hyperactivity%2C%20irritability,at%20all%20or%20feeling%20numb.

2)권정혜, 안현의, 최윤경, 주혜선. 재난과 트라우마의 심리적 응급처치 2판. 서울: 학지사(2014). p30-31.

3)권정혜, 안현의, 최윤경, 주혜선. 재난과 트라우마의 심리적 응급처치 2판. 서울: 학지사(2014). p32.

2) 재난 후 단계별 반응

재난 사건의 충격 후 적응 과정에 따라 다음과 같은 반응 단계가 나타나며 각 단계별 심리 지원이 이뤄져야 한다[4]. 재난 후 시기에 따라 급성기(사고 후 3~7일), 아급성기(1~3개월 이내), 만성기(3개월 이상)로 구분하기도 한다[5]. 이런 변화들은 재난 충격을 이겨내기 위한 정상적 반응이지만, 일부 사람들에게는 만성화되어 정신장애가 발생할 수 있다.

(1) 적응 과정에 따른 반응 단계

❶ 충격기(사고 후 0~48시간)

사건 직후 단계로 투쟁/도피 반응이 일어나 얼어붙거나 자포자기하는 행동을 보인다. 교감신경계 항진 증상이 나타난다. 사건 직후 생존자들은 일종의 쇼크 상태로 주위에서 일어나는 모든 일이 현실인지 믿지 못하고 영화나 다른 사람의 일처럼 멍하니 바라본다. 대부분 생존을 위한 행동을 하며, 시야가 협소해져서 이성적으로 생각할 수 있는 능력이 저하되고 집중하지 못한다. 행동이 경직되고 다른 사람들과 의사소통이 잘 안 된다. 불신감, 불감증, 두려움, 혼란을 포함한 감정들이 강하게 나타난다.

❷ 급성기(구출기)

영웅기(사고 후 3~7일 이내), 밀월기(3~7일 이내), 환멸기(7일~수년 이내)로 구분한다.

영웅기

구출 단계에서 사람들은 충격에서 벗어나 다른 사람을 돕고 구조활동에 참여한다. 구조 대원, 가족, 그리고 이웃들 사이에서 서로 협조하고 영웅적 행위들이 나타나기도 한다. 생존자들은 살아 있다는 것만으로도 기쁨을 느끼고 무엇이든 할 수 있을 것 같은 전능감을 경험한다.

밀월기

그 후 재난 지역과 생존자들에게 사회적 관심이 집중되고 대중매체가 구출담이나 재난 경험을 보도함으로써 마치 자신이 세상 중심에 서 있는 것 같이 느낀다. 생존자들은 에너지가 넘치고 생리적으로 각성되어 신체적 활동을 활발히 한다. 그러나 인지 기능은 떨어져 집중하기 어렵고 이해력과 문제해결력이 떨어지며 우선순위를 잘 정하지 못해 효율성은 전반적으로 낮다.

---

4)권정혜, 안현의, 최윤경, 주혜선. 재난과 트라우마의 심리적 응급처치 2판. 서울: 학지사(2014). p34-37
5)재난정신건강위원회 편. 재난과 정신건강. 서울:학지사(2015). p111-118

환멸기

시간이 지나면서 재난 현장에 쏟아졌던 관심을 줄어들고, 생존자들은 기대했던 보상이나 필요한 지원을 받는 데 많은 규정과 제한을 경험하며, 피해 복구가 지연되는 것에 실망과 환멸을 느낀다. 환멸기는 재난 직후 7~8일 후 시작되어 7~8주, 때로 수년 동안 계속될 수 있다. 생존자들은 재난을 통해 잃은 많은 것들을 결코 되찾을 수 없음을 깨닫고 깊은 상실감과 슬픔을 경험한다. 신체적 에너지도 고갈되어 피로감이 몰려오고 일상 리듬이 깨졌던 후유증이 몰려와 신체적으로 소진된다. 여러 감정에 압도되어 무력감을 느끼고, 사건 현장에 돌아가는 것을 두려워하며, 악몽, 불안, 불면증에 시달린다. 또한 긴장되어 잘 놀라고 몸이 뻣뻣해지고 떨리며 재난 현장에서 겪었던 일들이 플래시백으로 나타난다. 쉽게 짜증을 내고 혼자 있고 싶어 하며 우울해한다. 살아난 것에 대해 갑자기 죄책감에 시달리기도 한다. 멀쩡하게 있다가 갑자기 눈물을 흘리는 등 기분이 자주 바뀌어 당황하고 무능감과 통제 불능감을 느끼며 다른 사람들의 부정적 평가를 염려한다. 그런 일이 일어났다는 것과 보호받지 못하고 이해받지 못했다는 것에 대해 사람들과 세상에 대해 분노를 느끼며 거리감을 둔다.

❸ 회복기(사고 후 1~4주)

불편한 증상들과 부인(denial)이 번갈아 나타난다. 교감신경계 항진(과잉 놀람 반응, 신경과민, 불면, 악몽 등) 및 예상하지 못한 감정과 생각들이 나타난다. 적응이 끝날 무렵 재난 충격에 대해 흔히 부인하며 피로, 현기증, 두통, 구토 등 신체 증상이 나타나 병원 방문도 잦아진다. 분노, 과민, 무관심, 사회적 위축도 흔히 나타난다.

구출 단계의 스트레스 반응이 지속되지만 반응 강도는 점차 약해져서 괴로워하던 감정과 생각을 추스르기 시작한다. 일상생활에 대한 관심이 조금씩 살아나며 미래를 계획하기 시작한다. 생존자들은 신체적, 정서적으로 회복되기 시작하며 재난으로 파괴되었던 건물이나 도로도 조금씩 복구되기 시작한다. 실망하는 일도 있지만 회복하고자 노력했던 일들이 작거나 크게 성과를 거두며 삶의 의욕을 되찾는다.

❹ 재통합기(사고 후 2주~2년)

개인에 따라 재통합에 걸리는 시간은 달라진다. 스트레스 반응이 더 이상 나타나지 않으며 재난 사건을 받아들이고 자신의 삶에 통합한다. 이 과정은 개인의 대처 역량과 사회적 지지에 따라 달라진다. 이 단계에서 여전히 어려움을 겪는 사람은 전문적인 치료에 의뢰하고 지속적으로 관리해야 한다.

그림 1 재난 후 반응 단계[6)]

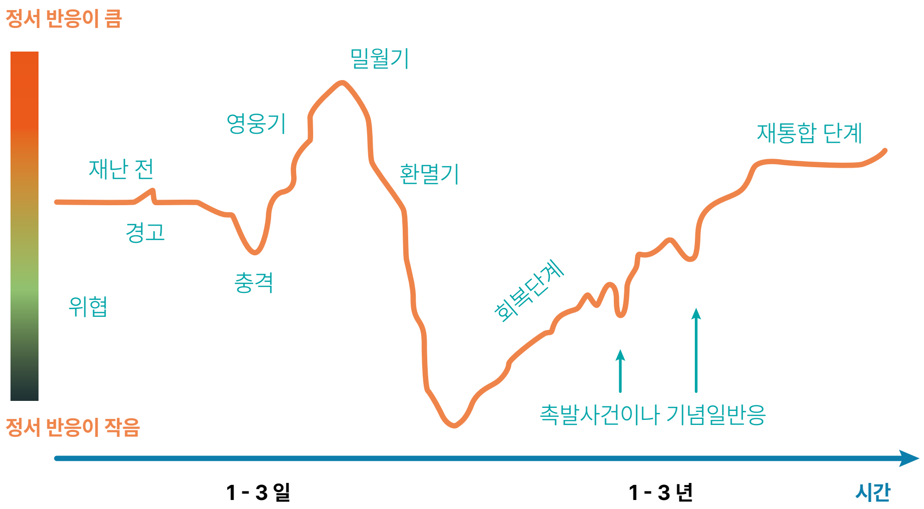

(2) 재난 후 시기별 반응

❶ 급성기(사고 후 3~7일)

재난 발생 직후 망연자실한 상태로 심한 트라우마의 충격에서 자신을 보호하는 반응이다. 정신이 멍해지고, 판단력이 떨어지고, 주변 환경과 사람을 낯설게 느끼며, 꿈꾸는 것 같은 상태가 나타날 수 있다. 발생 직후 이 같은 억압, 부정, 해리는 자연스런 반응이지만 지속되면 외상후스트레스장애 발생으로 이어질 수 있다.

❷ 아급성기(1~3개월 이내)

- **과각성 :** 재난 사실에 극도의 공포와 불안이 나타나며 또다시 재난이 발생할까 두려워한다. 깨어 있을 때 죽음에 대한 공포도 경험한다. 사고 장면을 회상하고 악몽을 꿀 수 있다. 충격적인 사고 장면이 플래시백으로 나타날 수 있다. 그러므로 사고와 연관되는 장소나 사람 등을 회피한다. 작은 자극에도 잘 놀라고 예민해져 자주 화를 내기도 한다. 목부위 긴장, 자세 경직, 입마름, 이리저리 움직이는 눈동자, 심박수 증가, 얕고 고르지 못한 호흡, 손이 차가움, 창백한 피부, 무릎과 다리에 힘이 빠짐, 손을 꽉 쥠, 복부 불편감, 메스꺼움, 손과 이마에 식은땀, 겁에 질려 우는 것과 같은 증상이 나타날 수 있다.

6)Zunin & Myers as cited in DeWolfe, D. J., 2000. Training manual for mental health and human service workers in major disasters (2nd ed., HHS Publication No. ADM 90-538)

- **우울/애도반응/죄책감 :** 같은 재난 사건으로 사망한 가족이나 친척, 친구, 사고로 인한 부상, 사고로 인한 경제적·사회적 손실 등으로 우울과 애도반응이 나타난다. 기분이 저하되고 의욕이 없으며 불면과 식욕부진, 무기력을 호소하여 부정적 생각에 집착한다. 인생을 의미없게 여기며 사람들과 정서적으로 멀게 느끼며 대인관계가 어려워진다. 심하면 어떤 기분도 느끼거나 표현하지 못하며 감정기복을 호소할 수도 있다. 자신이 살아남은 것과 재난 시 자신의 행동과 책임에 대해 과대 해석하고 자책하고 자기 비하에 시달린다. 다른 사람들에게 비난받을지도 모른다는 두려움에 죄책감을 숨기기도 한다. 죄책감을 느끼면 타인의 도움을 구하지 않는 경향이 크므로 외상후스트레스장애가 발생한 가능성이 높다.

- **불신/고립감 :** 자신이나 타인을 극단적으로 부정적으로 보고 아무도 믿을 수 없다고 생각한다. 가족, 지인 등의 동정에 큰 거부감을 나타낸다. 자기 경험을 아무도 이해하지 못할 것이라고 생각하며 생존자들끼리만 집단을 형성하고 타인에 대해 분노한다. 세상을 매우 위험한 곳으로 바라본다. 자신의 고통을 누구에게도 말하지 못하고 고립감과 소외감을 느낄 수 있다. 신체 부상으로 인한 입원이나 외래 치료가 끝나고 집이나 직장에 빨리 복귀하면 고립감이 심해지고 적응이 어려워질 수 있다.

- **인지기능 변화 :** 의식, 기억, 주체성, 환경 지각 등 통합적 기능이 손상되는 해리 증상이 발생할 수 있다. 특히 기억상실 및 기억력 저하, 지각장애, 집중력 저하가 나타날 수 있다.

- **신체증상** : 재난 생존자는 재난 충격으로 인한 스트레스 반응으로 피로, 두통, 식욕 저하, 소화불량, 배변 문제, 불면, 통증, 면역기능 저하 등의 증상을 흔히 호소한다. 신체적 고통은 불안, 우울, 죄책감 등 심리 증상을 악화시키며 활동을 제한하여 사회 복귀를 지연시키고 삶의 질을 저하시킨다.

- **물질의존** : 불안과 신체적 고통에서 벗어나고자 재난 경험 후 생존자의 음주, 흡연, 향정신성 약물 사용량이 증가할 수 있다. 하지만 지속적으로 사용하면 물질 남용과 의존을 발생시켜 개인의 건강을 악화시키고 가족관계를 손상시키며 사회적 문제가 발생할 수 있다. 중독이 동반될 경우 트라우마 및 우울 증상은 더욱 악화될 수 있다.

❸ 만성기(3개월 이상)

생존자의 불안, 초조, 분노 상태가 잘 조절되지 않고 지속된다. 본인의 증상이 회복되지 않는 것과 사고 전 일상생활로 돌아갈 수 없음을 초조하게 생각하고 치료에 회의적이 되며 우울증이 심해진다. 이전으로의 회복의 희망을 잃고 절망감에 빠지거나 자포자기하거나 자살을 시도할 수 있다. 애도반응도 지속 될 수 있다. 타인의 이해와 관심이 줄어들면서 고립감과 불신감이 심해진다. 피해 보상 등 법적 문제는 단기간에 해결되지 않으므로 갈등과 분노, 우울이 악화될 수 있다. 알코올, 진통제, 신경안정제 등 물질의존 문제가 심각해질 수 있다.

(3) 재난 후 정신장애

세계정신건강조사(The World Mental Health)에 따르면 재난 상황에서 외상후스트레스장애 유병률은 고위험군에서 44.5%로 매우 높았다[7]. 재난 직후 급성스트레스장애는 더 흔히 나타나는데, 외상후스트레스장애로 지속되지 않도록 적극적인 치료(심리치료, 약물치료)가 필요하다. 트라우마와 연관되는 장애로는 외상후스트레스장애 외에도 주요우울장애, 물질사용장애, 범불안장애, 적응장애, 신체화, 그리고 두부 손상, 독성물질, 질환, 탈수에 의한 이차적 기질성 정신장애가 있다.

가족 사망으로 인한 복합 애도, 재난회복과 관련된 보상 문제, 실직 같은 스트레스 요인은 이와 같은 정신장애의 발병에 영향을 줄 수 있다[8]. 주요우울장애와 물질 중독은 외상후스트레스장애와 흔히 함께 발생하며, 물질 중독은 자동차 사고, 위험한 성행위, 가정폭력 등 위험행동으로 이어져 사망률이 증가할 수 있다.

외상후스트레스장애와 주요우울장애는 재난트라우마에 대한 정상 반응이 아니라 치료해야 할 심각한 질환이다. 외상후스트레스장애를 발견하는 실마리는 뚜렷한 회피와 무감각 증상이다. 외상후스트레스장애로 진단되더라도 추가적인 정신과적 평가가 필요하다. 보통 다른 정신장애가 동반되거나 외상후스트레스장애 없이 다른 정신장애만 단독으로 발생할 수 있기 때문이다. 효과적인 치료와 회복을 위해 동반된 다른 정신장애를 평가하는 것이 외상후스트레스장애의 평가만큼 중요하다. 여성은 외상후스트레스장애와 주요우울장애 발병에 더욱 취약한 반면 남성은 중독 문제가 발생하기 쉽다.

그림 2 외상후스트레스장애 진단기준[9](DSM-5; 부록 참고)

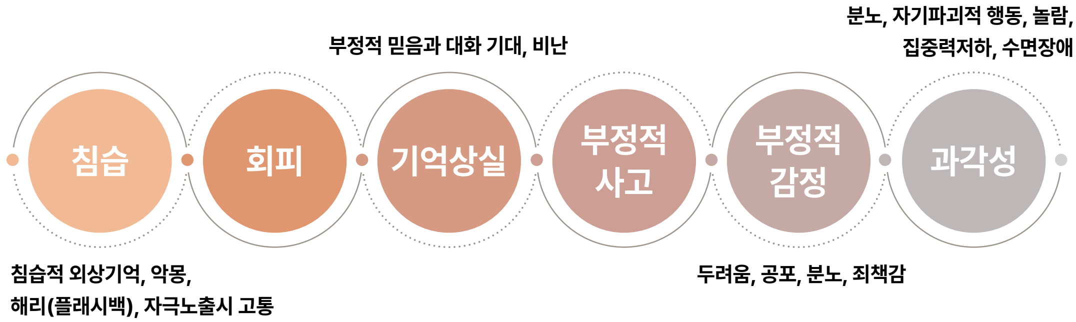

7)Bromet EJ, Atwoli L, Kawakami N, Navarro-Mateu F, Piotrowski P, King AJ, Aguilar-Gaxiola S, Alonso J, Bunting B, Demyttenaere K, Florescu S, de Girolamo G, Gluzman S, Haro JM, de Jonge P, Karam EG, Lee S, Kovess-Masfety V, Medina-Mora ME, Mneimneh Z, Pennell BE, Posada-Villa J, Salmerón D, Takeshima T, Kessler RC. Post-traumatic stress disorder associated with natural and human-made disasters in the World Mental Health Surveys. Psychol Med. 2017 Jan;47(2):227-241.

8)Kessler RC, McLaughlin KA, Koenen KC, Petukhova M, Hill ED, 국제보건기구(WHO) World Mental Health Survey Consortium. The importance of secondary trauma exposure for post-disaster mental disorder. Epidemiology and psychiatric sciences. 2012 Mar;21(1):35.

9)American Psychiatric Association. 정신장애의 진단 및 통계 편람 (제5판)(권준수, 김재진, 남궁기, 박원명, 신민섭, 유범희. 임효덕 역.). 서울: 학지사. p289-292.

표 3 재난 후 정신장애와 심리·신체·행동적 증상

**정신장애**	• 외상후스트레스장애, 급성스트레스장애, 주요우울장애, 물질사용장애, 범불안장애, 적응장애, 신체화장애, 두부손상<br>• 독성물질, 질환, 탈수에 의한 이차적 기질성 정신장애
**심리적, 신체적 증상 및 행동 문제**	• 분노, 짜증, 불신, 슬픔, 불안, 공포<br>• 수면장애<br>• 알코올, 카페인, 담배 사용량 증가<br>• 가족갈등<br>• 실직으로 인한 경제적 피해로 인한 고통<br>• 신체증상(불면, 피로, 상복부통증, 어지럼증, 두통, 근골격계 증상)
**사별과 애도반응**	• 지속성 애도장애<br>• 복합 사별장애

(4) 재난 발생 시 고위험군

트라우마 후 정신장애는 재난을 직접 경험한 1차 생존자에서 주로 발생하며, 트라우마 스트레스의 강도가 높을수록 정신과적 문제가 많이 발생한다. 특히 신체적 위험을 경험하거나 재난을 직접 목격한 사람이 트라우마에 취약하며, 1차 생존자와 애착관계가 뚜렷했던 사람이나 초기 대응요원, 자원봉사자 또한 트라우마 발생에 주의해야한다. 재난 경험 전에 심리적으로 취약한 사람들도 공포, 실직의 현실, 지나치게 긴 출퇴근 시간, 망가진 대인관계, 사회적 지지의 부족 등으로 고통받는다.

표 4 재난 발생 시 고위험군

재난 발생 시 고위험군	
• 재난을 직접 목격한 자	• 노인
• 신체 부상자	• 여성
• 초기 대응요원	• 과거 외상후스트레스장애를 가진 자
• 사별한 자	• 과거 트라우마 경험이 있는 자
• 한부모	• 정신장애 및 심각한 만성질환이 있는 자(과거나 현재)
• 소아	• 사회적 지지가 부족한 자

5. 국내 재난심리지원 체계

1) 재난대응 지침

재난정신건강 대응의 핵심적 틀에는 세 가지 기본 요소, 즉 1) 대상자 평가, 2) 중증도 파악, 3) 치료가 포함된다. 첫 번째 작업은 외상후스트레스장애나 주요우울장애 같은 정신장애를 일반적 고통과 구분하여 확인하는 것이다. 외상후스트레스장애 진단에는 최소 1달 이상 증상이 지속되는 기준이 충족되어야 한다. 재난 상황 속 정신장애에 효과적인 치료법으로 약물치료와 심리치료가 있다[10]. 외상후스트레스장애 등 정신장애로 진단되지 않는 경증 및 중등도 증상을 호소하는 생존자에는 실질적이고 따뜻한 지원이 도움이 될 수 있다. 그 한 가지 방법이 바로 심리적 응급처치이며 다양한 상황에서 전문가뿐 아니라 적절한 교육을 받은 일반인들도 시행할 수 있다. 심리적 응급처치는 전문가 합의에 의해 개발되었는데 9·11 이후 재난 급성기 치료로 널리 활용되고 있지만 그 효과는 충분히 검증되지 않았다[11]. 하지만 재난 직후에는 역치하 증상을 가진 생존자가 많기 때문에 이를 위한 실질적 지원과 도움이 필요하다.

재난의 충격을 평가할 때 트라우마 노출 수준만으로 생존자를 일방적으로 진단하면 안 된다. 또한 재난을 경험한 생존자의 경우 자가보고형 외상후스트레스장애 설문만으로 외상후스트레스장애를 평가하는 것은 적절하지 않다. 정신과적 병력이 있거나 사회적으로 고립된 사람들, 심각한 개인적·사회적 어려움을 가진 사람들은 만성 정신장애가 발생할 위험이 높으므로 주의 깊게 관리해야 한다.

외상후스트레스장애 증상은 재난 후 바로 시작되고 만성화되기 쉽기 때문에 조기에 치료하는 것이 바람직하다. 초기 대응 단계에서는 대규모 피해자가 발생하므로 대응능력이 마비될 수 있다. 수개월이 지나면 생존자의 정신장애는 고착화되어 더 많은 도움을 필요로 한다. 지속적 지원이 필요하지만 시간이 흐르면서 외부의 도움과 생존자에 대한 관심은 줄어든다. 또한 재난 직후 외상후스트레스장애 증상이 나타나지만 생존자들은 심리지원을 뒤늦게 찾거나 많은 사람들이 적절히 치료를 받지 못하고 있다.

---

10) North CS, Pfefferbaum B. Mental health response to community disasters: a systematic review. Jama. 2013 Aug 7;310(5):507-18.

11) Shultz JM, Forbes D. Psychological first aid: Rapid proliferation and the search for evidence. Disaster Health. 2014 Jan 1;2(1):3-12.

2) 국내 재난심리지원 체계

2021년 10월 현재 국가 재난심리지원은 재난심리회복지원센터(행정안전부 산하)와 국가트라우마센터(국립정신건강센터/보건복지부 산하)에서 담당하며, 지방자치단체(시, 도)와 함께 재난심리회복지원센터를 지정, 지원하고 있다. 2018년 국립정신건강센터에 국가트라우마센터를 설치·운영하면서 국가트라우마센터가 대규모 재난 시 재난심리지원을 총괄하게 되었다. 국가트라우마센터에서는 평소 재난 정신건강 대응체계를 구축하고, 재난 정신건강 전문 인력 양성 및 교육 훈련, 재난 정신건강 서비스 고도화를 위한 연구 개발을 하며 국가적 재난 발생 시 관계 기관과 협력하여 신속하고 일원화된 개입체계를 구축하여 재난 현장 위기대응 서비스를 제공하고, 정신건강 고위험군을 선별하여 트라우마 치료 프로그램을 제공한다[12].

그림 3 재난심리지원 운영 체계(안)(국립트라우마센터 홈페이지)

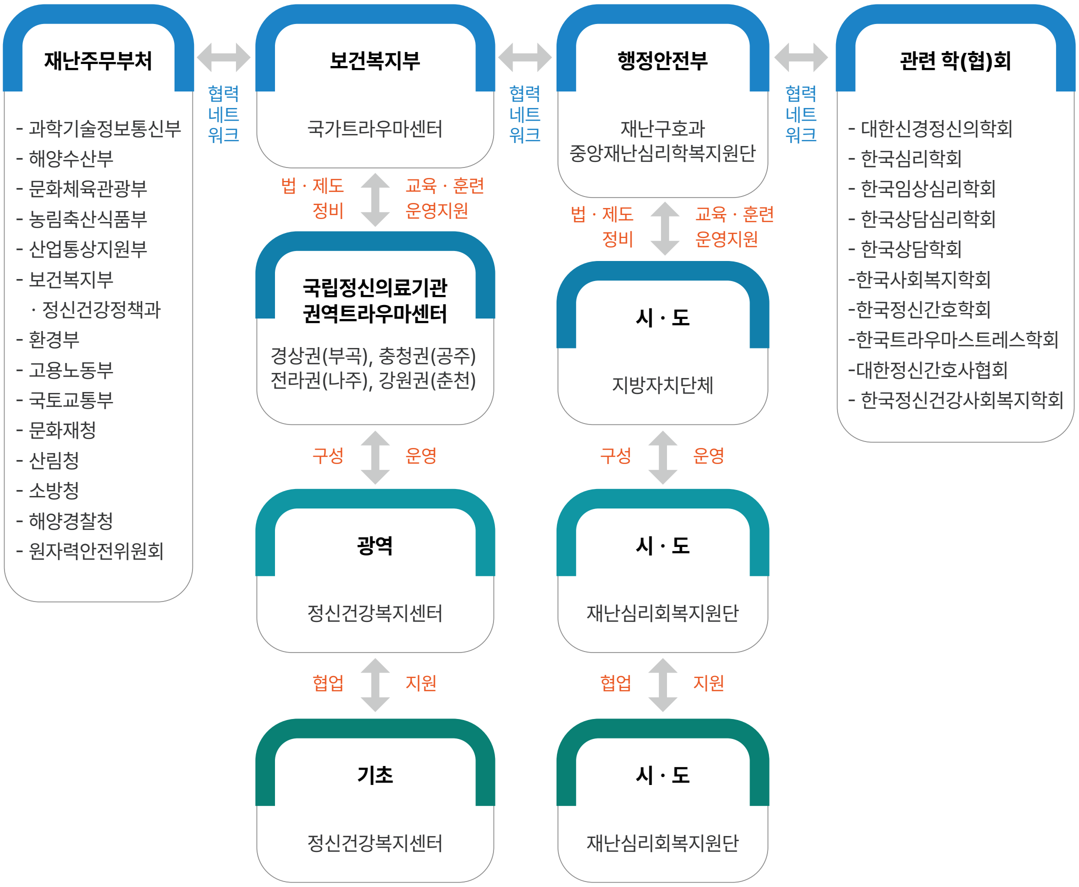

12)국가트라우마센터. 재난 정신건강 실무자를 위한 표준매뉴얼(안). 서울:국립정신건강센터;2019. p13-17.

3) 국가 재난심리지원 내용[13)14)]

표 5 국가 재난심리지원 단계별 지원활동

재난시 대응	1단계 정보수집	충격기 재난 직후	· 재난발생 정보 수집<br>· 대응 비상연락망 가동
재난시 대응	2단계 계획수립	충격기 재난 직후	· 재난상황 모니터링<br>· 재난심리지원 계획 수립 및 대응체계 구성(발생지역 통합재난심리지원단내 심리지원팀 구성)<br>· 관계부처(협력기관 및 통합자문단) 및 지차체 유관기관, 학회, 상담 관련 인력 확보
재난시 대응	3단계 현장대응	급성기 1개월 이내	· 재난심리지원 활동 시행 및 유관기관 홍보<br>· 심리적 응급처치(PFA) 수행<br>· 초기 평가(생존자 요구, 거처 및 가족 상태 파악)<br>· 고위험군 선별 및 연계, 고위험군에 즉각적 위기 개입<br>· 안심버스 운영<br>· 담요, 핫팩 등 안정화 물품 지원 서비스<br>· 트라우마 후 심리상태와 대처법에 대한 소책자 제공
재난시 대응	3단계 현장대응	아급성기 1~3개월 이내	· 안정화 기법 활용<br>· 병의원 연계<br>· 생존자 자조활동 지원
재난후 회복	4단계 회복지원	만성기 3개월 후	· 관심군 및 고위험군에 맞춤형 트라우마 회복프로그램 제공 (마음프로그램, 마음플러스, 허그)<br>· 전문치료기관 연계<br>· 1, 3, 6개월 모니터링 및 위험도별 사례관리 지속<br>· 치료 종결 및 지역사회 연계

13)국가트라우마센터. 재난 정신건강 실무자를 위한 표준매뉴얼(안). 서울:국립정신건강센터;2019. p31-52.
14)고영훈. 안산정신건강트라우마센터 재난심리지원 안내서. 경기:안산정신건강트라우마센터;2017. p14-31.

1단계

충격기(재난 직후)
정보수집

- 주기적 재난 발생여부를 모니터링하고 재난 발생 가능성이 높은 경우, 관련 재난 정신건강에 대한 정보 제공 및 대비 활동을 권장한다.

2단계

충격기(재난 직후)
계획수립

- 재난 생존자에게 마음건강 회복 안내서 및 심리 안정 용품을 제공하고, 재난심리지원 인력에게 현장대응지침서를 제공한다.

3단계

급성기(1개월 이내) 및 아급성기 (1~3개월 이내)
현장대응

- 재난 발생지역 내 관련 기관에 재난심리지원 활동이 이뤄짐을 알리고 생존자에게 정보 전달을 요청한다.
- 심리적 응급처치(PFA)를 수행한다.
- **안정화 기법 :** 심신안정 신체요법 프로그램(마사지 활용 등 신체적 안정화 집중)
- 고위험군을 선별하여 필요시 전문 치료기관으로 연계한다.
- **병의원 연계 :** 생존자들은 다양한 신체적, 심리적 문제가 발생하여 여러 가지 의료지원이 필요하나 스스로 병원을 찾는 것이 어렵다. 그러므로 사례관리자가 상황을 파악하여 대상자와 병원을 적절히 연계해주는 것이 필요하다.
- 재난 현장 상담소와 함께 안심버스를 운영한다. 안심버스에서는 심리설문지 및 심박변이도 검사를 통해 스트레스를 평가하고, 심리교육 및 안정화 프로그램, 개별 심층상담을 제공한다.
- 생존자들의 자조활동 지원: 생존자 스스로 자조모임을 형성하는 장을 마련해야 한다. 전문가라도 임시로 외부 인력을 활용하는 것은 한계가 있으며 지역사회 인력이 지속적으로 자조활동 및 도움을 줄 수 있도록 지역사회 인력의 전문성을 강화하는 것이 필요하다.
- 신뢰관계를 형성하기 위해 생존자들이 활동하는 곳에 함께한다.
- 실질적으로 필요한 담요, 핫팩 등 안정화 물품 및 반찬지원 서비스를 진행한다.
- 트라우마 후 심리상태와 대처법에 대한 소책자를 제공한다.
- 홍보, 브리핑, 취재 등에 협조한다.
- 조력자 역할이 가능한 시민치유자 양성과정 교육을 개설한다.
- 생존자가 대피소가 아닌 집에 있다면 직접 생존자를 만나는 아웃리치가 중요하다.

4단계

만성기(3개월 후)
회복지원

- 사례별 위험도에 따라 월1회~주2회 방문 및 전화, 우편발송을 통해 관리를 지속한다.
- 초기 평가, 상담을 통해 고위험군을 선별한다.
- 맞춤형 트라우마 회복프로그램 계획을 수립한다.
- 지원기간은 최대 12개월이다.
- 제공 프로그램
  ❶ **마음프로그램 :** 생존자의 스트레스 조절을 돕는 안정화 기법 중심의 회복 프로그램
  ❷ **마음플러스 :** 미국 국립 외상후스트레스장애 센터와 국립 아동 트라우마 스트레스 네트워크에서 개발한 근거기반 모듈 방식의 마음건강 회복 기술 훈련(Skills for Psychological Recovery, SPR)을 활용하여 재난 후 스트레스에 효율적인 대처를 돕는 회복 프로그램
  ❸ **허그프로그램 :** 트라우마 기억 재처리와 통합을 유도하는 트라우마 심리치료(지속노출치료, 안구운동 민감소실 및 재처리, 내러티브 노출치료)를 활용
- 회복프로그램 종결 후 사후 평가를 통해 그 효과를 측정하고, 1, 3, 6개월 간격 모니터링 사후관리를 시행한다.

II

진료 프로토콜

01 진료흐름도

02 병력청취 및 문진 매뉴얼

03 단계별 진료 프로토콜

04 증상별 진료 프로토콜

05 심리적 응급처치

06 기타 고려사항

Ⅱ. 진료 프로토콜

1. 진료흐름도

그림 4 재난트라우마에 대한 한의 진료흐름도

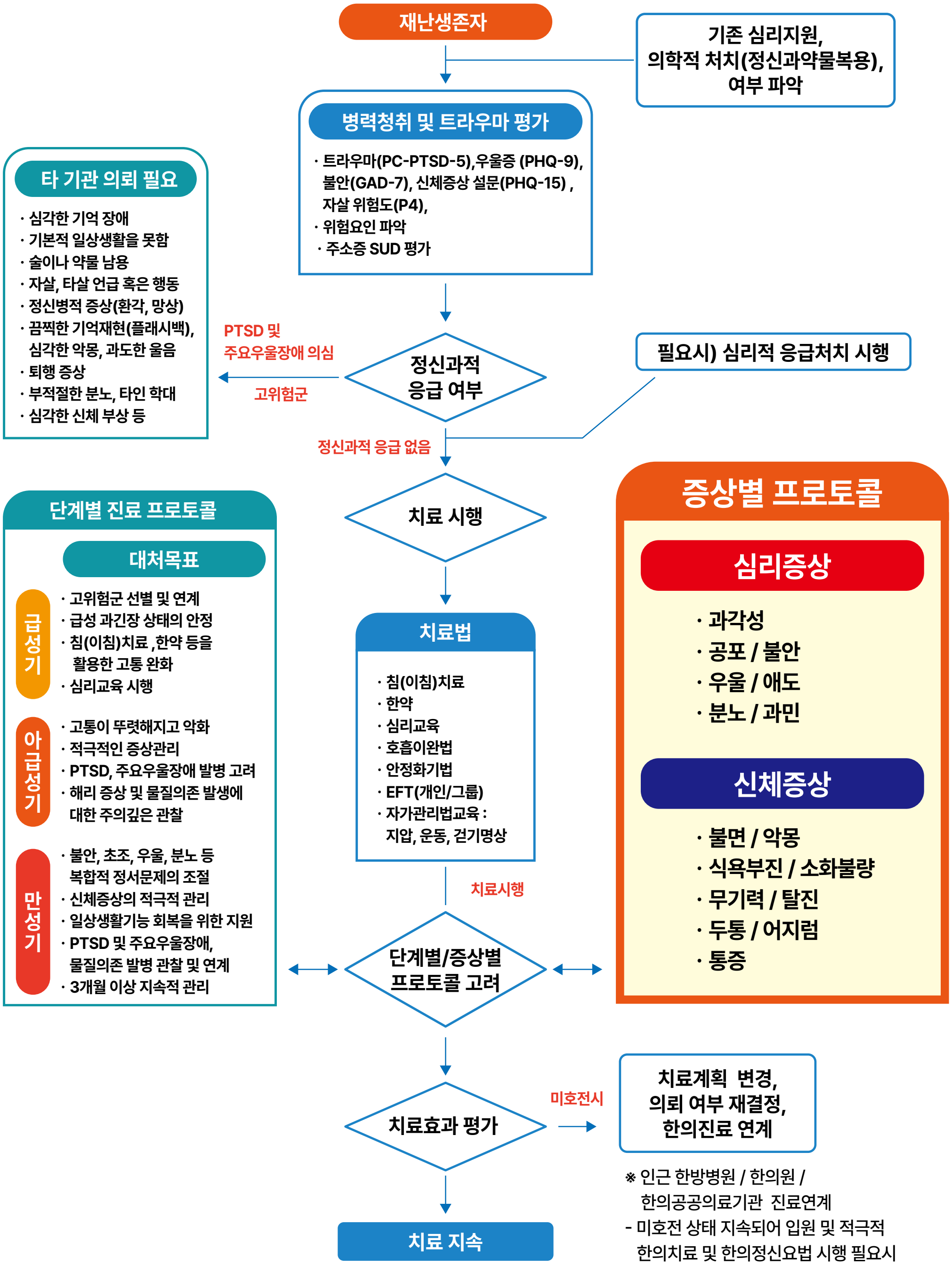

2. 병력청취 및 문진 매뉴얼

1) 재난현장에서의 진료지침[15)]

재난 생존자들의 행동 변화는 재난이라는 급격하고 예상치 못한 상황에서 보일 수 있는 정상 반응이다. 따라서 이를 섣불리 질병으로 규정하지 않고 정상적인 행동양식임을 이해하고 진찰한다.

- 천천히 말한다. 단순하고도 구체적인 용어를 사용한다. 전문용어를 사용하지 않는다.
- 생존자가 어떤 일을 겪었는지, 어떤 처지에 있는지 섣부른 가정을 하지 않는다.
- 섣불리 질병으로 규정하지 않는다. 대부분 급성 반응은 이해할 만한 정상 반응이다.
- 생존자의 무력감, 약점, 실수, 결함에 초점을 맞추지 않는다.
- 트라우마에 대한 상세한 내용을 듣는 것은 목표가 아니며 억지로 트라우마 상황을 떠올리게 하지 않도록 주의한다.
- 정확한 정보만 제공하며 모르는 경우에는 추론하지 말고, 모른다고 솔직하게 대답한다.
- 재난 후 회복을 위해 재난생존자가 안전하고, 삶을 예측할 수 있으며, 통제력을 가지고 있다고 느끼는 데 도움이 되는 것들을 찾아야 한다.
  - 신체적, 정신적 안전 확보를 위해 지금은 안전하다는 것을 상기시킨다
  - 전반적인 진료 과정을 설명하여 예측 가능한 대로 시행할 것임을 인지시킨다.
  - 재난생존자들의 선택에 따라 진료과정이 이루어짐을 주지시킨다.

2) 기본 문진

먼저, 진료에 대해 생존자의 동의를 얻은 후 진료를 시행한다. ① 인구학적 조사, ② 트라우마 선별검사, ③ 기초 문진, ④ 상세 문진, ⑤ 위험요인 문진, ⑥ 한의학적 진찰, ⑦ 주소증별 유형 구분의 순으로 기본 문진 및 트라우마 평가를 시행한다. 단, 담당 한의사는 급박한 현장 상황을 고려하여 지나치게 자세한 문진은 주의한다.

※ 진료기록부(예시)(부록2.)

- 필요시 본 진료기록부를 구글엑셀로 작성하여 온라인차트로 이용할 수 있다.

15)행정안전부. 재난심리회복지원 매뉴얼 개정본. 행정안전부 재난구호과;2017. 83p.

3) 재난 트라우마 평가

(1) 선별검사

급성기 중재의 효과는 빠르고 정확한 선별 평가에 좌우되기 때문에, 재난 생존자들의 반응을 평가해서 중재를 시작해야 하고, 재난 생존자의 반응을 지속적으로 관찰하며 어떤 수준의 중재가 필요한지 결정해야 한다[16].

재난 생존자의 초기 반응을 정확하게 평가하지 못할 경우 정말로 도움이 필요한 사람과 그렇지 않은 사람을 구별하지 못할 수 있다. 또한 정서적 반응에만 초점을 맞추는 경우 정서가 일단 안정되면 증상이 사라진 것처럼 보여 치료가 중단될 수 있으므로 주의한다[17].

본 매뉴얼에서는 국가트라우마 심리지원 과정과 원활한 협진체계 구축을 위해 트라우마 선별 평가도구로서 국가트라우마센터에서 제시하는 단축형 마음건강 검사지를 활용한다[18]. 이 검사지는 외상후스트레스 증상(5문항)[19], 우울(9문항)[20][21], 불안(7문항)[22][23], 신체증상(15문항)[24][25], 자살사고(4문항)[26]를 측정하는 단축형 척도로 구성되어있으며 총 문항은 40문항이다. 심리검사 실시 전 개인정보 수집 및 이용 동의서에 대해 설명한 후 동의서를 받는다. 국가트라우마센터 홈페이지 및 어플리케이션(마음프로그램)에서 제공하는 자가진단 서비스를 활용할 수 있다[27]. 본 설문지는 부록(부록4~8.)에 제시되어있다.

16)Hoff, L. A. People in crisis: Understanding and helping 4th ed. Redwood city, CA:Addison-Wesley;1995. 105-132p.

17)Myer RA, Conte C. Assessment for crisis intervention. J Clin Psychol. 2006;62(8):959-970.

18)국가트라우마센터. 재난 정신건강 실무자를 위한 표준매뉴얼(안) 개정본. 2019, 121-125p

19)Jung YE, Kim D, Kim WH, Roh D, Chae JH, Park JE. A Brief Screening Tool for PTSD: Validation of the Korean Version of the Primary Care PTSD Screen for DSM-5(K-PC-PTSD-5). Journal of Korean Medical Science 2018;33(52):e338.

20)Spitzer RL., Kroenke K., Williams JBW. Validation and utility of a self-report version of PRIME-MD: the PHQ primary care study. The Journal of the American Medical Association. 1999:282(18);1737-1744.

21)안제용, 서은란, 임경희, 신재현, 김정범. 한국어판 우울증 선별도구 (Patient Health Questionnaire-9, PHQ-9) 의 표준화 연구. 생물치료정신의학. 2013 Jun;19(1):47-56.

22)Pfizer Inc. The Korean Version of the GAD-7. (2018). Available online at: http://www.phqscreeners.com (Accessed April 1, 2018).

23)Ahn JK, Kim Y, Choi KH. The psychometric properties and clinical utility of the Korean version of GAD-7 and GAD-2. Frontiers in psychiatry. 2019 Mar 18;10:127.

24)Kroenke K., Spitzer RL., Williams JB. The PHQ–15: validity of a new measure for evaluating the severity of somatic symptoms. Psychosomatic Medicine. 2002;64:258–266.

25)Han C, Pae CU, Patkar AA, Masand PS, Kim KW, Joe SH, Jung IK. Psychometric properties of the Patient Health Questionnaire-15(PHQ-15) for measuring the somatic symptoms of psychiatric outpatients. Psychosomatics. 2009;50(6):580-585.

26)박주언, 김원형, 노대영, 원성두, 김하경, 강석훈, 홍나래, 박성용, 김대호, 채정호. 재난정신건강평가 워크북. 대한불안의학회, 정신건강기술개발사업단. 2016.

27)국가트라우마센터/재난정신건강정보/자가진단https://nct.go.kr/distMental/rating/rating02_1.do

그림 5 국가트라우마센터 제공 어플(마음프로그램) 및 홈페이지의 자가진단 서비스

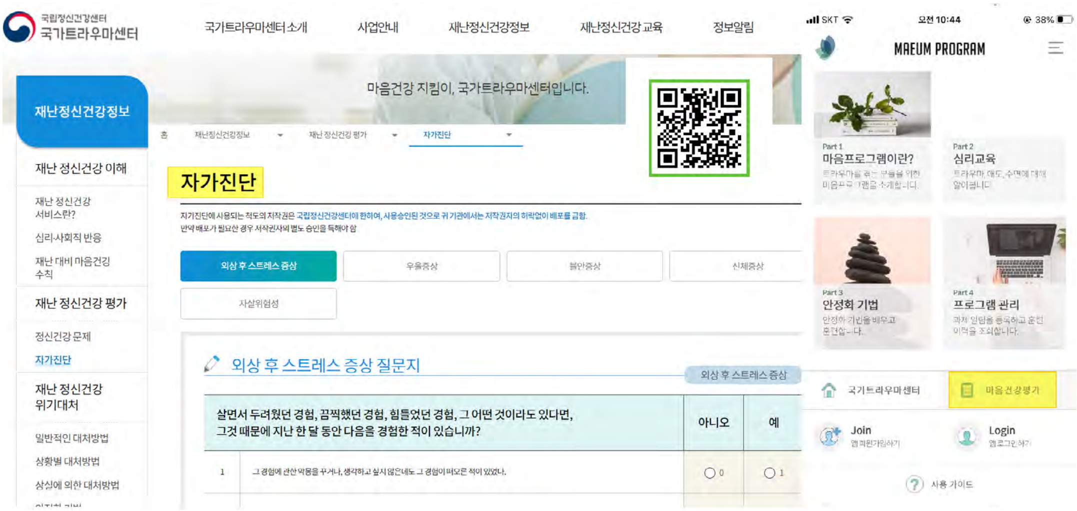

❶ 유의사항

- 각 척도별 지시문을 주의해서 읽고, 자기 증상과 가장 일치하는 한 문항만 선택하도록 한다.
- 모든 척도는 '검사 시점'으로부터 지시문에 표시된 기간 동안 경험한 증상을 체크하도록 한다. (예) 외상후 스트레스장애(the Primary Care PTSD Screen for DSM-5, PC-PTSD) 설문의 경우, '재난 경험 당시'가 아니라 '검사 시점'으로부터 지난 1달 동안 경험한 증상을 체크한다.
- 검사 종료 후, 누락 문항이 없는지, 역채점 문항(P4-4)에 잘 응답했는지 한 번 더 확인한다.

❷ 채점 방법

- 우울(The Patient Health Questionnaire-9, PHQ-9), 불안(Generalized Anxiety Disorder 7-item scale, GAD-7), 신체증상(The Patient Health Questionnaire-15, PHQ-15), 외상후스트레스 증상(PC-PTSD) 척도는 재난 경험자가 표시한 점수를 합산하여 채점한다.
- 자살(P4) 척도는 자살사고가 있는 경우에 한해, 1번 또는 2번 문항에 "있다"라고 답변한 경우, '자살위험성 낮음', 3번 문항에 '약간' 혹은 '매우 그렇다'라고 응답하거나 또는 4번 문항에 '없다'라고 답변한 경우, '자살 위험성 높음'으로 채점한다.

표 6 재난트라우마 평가설문(선별검사)

척도	정상	경도-중등도	고도
**외상후스트레스 증상 (PC-PTSD)**	0~1점	2점	≥3점
**우울 (PHQ-9)**	1~4점	5~9점	≥10점
**불안 (GAD-7)**	1~4점	5~14점	≥15점
**신체증상 (PHQ-15)**	0~4점	5~14점	≥15점
**자살 (P4)**	자살 과거력과<br>자살계획 없음	자살 과거력 또는<br>자살계획 있음	자살 가능성 있고<br>보호요인 없음

❸ 해석

선별검사 설문지의 심각도를 파악하고, 선별 요인 중 위험요인 항목(1.정신과적 과거력, 2.과거 트라우마 경험, 3.현재 지속되는 스트레스 사건, 4.취약한 지지체계)은 해당 요인이 있을 경우 '있음', 없을 경우 '없음', 확인되지 않은 경우 '모름'에 체크한 후 '있음'의 개수를 고려하여 고위험군, 관심군, 정상군을 구분한다.

표 7 고위험군 및 관심군 선별기준

구분	선별 기준
**고위험군**	다른 조건과 무관하게 자살척도(P4)가 '고도'인 경우<br>3개이상의 척도에서 '고도'인 경우
**관심군**	1개 척도라도 '경도' 이상인 경우<br>위험요인이 2개 이상인 경우
**정상군**	모든 척도가 정상인 경우

❹ 상세검사

보다 자세한 평가를 위해 다음 증상별로 아래와 같은 심리검사 도구를 활용할 수 있다[28).

표 8 증상별 상세 평가도구들

평가증상	평가 도구
**외상후스트레스장애**	외상후스트레스장애 체크리스트-5(Korean version of PTSD-5, PCL-5-K)
**우울**	벡우울 척도(Beck Depression Inventory, BDI)
**불안**	상태-특성 불안척도(State-Trait Anxiety Inventory, STAI), 화병증상 척도

28)박주언, 김원형, 노대영, 원성두, 김하경, 강석훈, 홍나래, 박성용, 김대호, 채정호. 재난정신건강평가 워크북. 대한불안의학회, 정신건강기술개발사업단. 2016.

**분노**	상태-특성 분노 표현 척도(State-Trait Anger Expression Inventory, STAXI)
**불면**	불면증 중증도 척도 (Insomnia Severity Index, ISI)
	피츠버그 수면의 질 지수(Pittsburgh Sleep Quality Index, PSQI)
**중독**	문제음주 선별검사<br>(the Alcohol Use Disorders Identification Test-Alcohol Consumption, AUDIT-C)
	파거스트롬 니코틴의존도 검사(Fagerstrom Test for Nicotine Dependence, FTND)
	영 인터넷중독 척도(Young Internet Addiction Scale, YIAS)
**애도**	복합 애도 척도(Inventory of Complicated Grief, ICG)

4) 주호소 증상의 분류

다음의 분류를 참고하여 재난 생존자가 가장 큰 고통을 호소하는 증상을 파악한다. 그리고 고통의 심각도가 어느 정도인지 주관적 고통평가척도(The Subjective Units of Distress Scale, SUDs)로 확인한다. 0에서 10 사이의 숫자로 스스로 평가하도록 한다.

표 9 주호소 증상의 분류

분류		
**심리증상**	**과긴장/불안**	재난 경험 후 일상에서 과도하게 긴장되어 있음. 급성기의 과긴장 상태가 만성화될 수 있음. 생각이 너무 많고 안절부절 못함.
	**공포/정서마비**	충격에 압도되어 심각한 불안 및 두려움을 호소하고 정서적으로 마비, 위축되고 얼어붙음. 충격에 대한 대처능력의 미비. 해리증상 발생가능.
	**우울/애도**	기분이 가라앉아있고 어떤 일도 즐겁지 않으며 무기력하고 죄책감에 시달림.
	**분노/과민**	과민해지고 폭력적이 되며 심하면 자해/타해의 위험성이 있음.
**신체증상**	**불면/악몽**	잠이 오질 않고 깊이 자지 못하며 악몽이 반복됨. 과긴장과 동반 고려.
	**식욕저하/소화불량**	입맛이 없고 가슴이 답답하며 소화가 안되고 불편함. 우울증과 동반 고려.
	**무기력/탈진**	피로해서 종일 누워있어야 하고 아무 것도 할 수가 없음. 만성기에 주로 나타남.<br>우울증과 동반 고려.
	**두통/어지럼증**	머리가 아프고 어지러움. 우울 및 분노와 동반 고려.
	**통증**	과긴장 및 재난 후 대피소의 불편한 생활 등으로 각종 근육통, 근육경련 등 발생 및 기존의 통증이 악화될 수 있음.

표 10 SUDs(The Subjective Units of Disturbance scale) 척도

**0점**	심리적 고통 없음
**1~2점**	약간의 심리적 고통이 있으나, 일상생활에 무리 없음
**3~6점**	심리적 고통이 집중을 어렵게 하거나, 정상적 활동을 방해
**7~8점**	심리적 고통이 상당하여, 활동에 제약
**9~10점**	표현할 수 있는 최대한의 심리적 고통

재난현장에서 한의사의 문진 및 치료과정을 정리하면 다음과 같다.

표 11 병력청취 및 문진 과정

**① 인구학적 조사**	성명, 성별, 생년월일, 연락처, 거주지 / 결혼 상태, 직업, 내소경로, 기존 심리지원 경험 여부
▼	
**② 기초 문진**	재난유형, 시기 단계, 재난 피해 수준별 경험자 구분, 활력징후
▼	
**③ 트라우마 선별검사**	단축형 마음건강 검사지: 외상후스트레스 증상(PC-PTSD), 우울(PHQ-9), 불안(GAD-7), 신체증상(PHQ-15), 자살(P4)
▼	
**④ 상세 문진**	발병일, 주소증, 현병력, 과거력, 가족력, 복약력, 음주 및 흡연력 등 파악
▼	
**⑤ 주호소 증상구분 및 평가**	심리증상이 뚜렷(과긴장/공포/우울/분노)<br>신체증상이 뚜렷(불면/식욕저하/무기력/두통·어지럼증/통증)
▼	
**⑥ 한의학적 진찰**	식욕·소화, 대소변, 수면, 구갈·구건, 오한·오열 및 주소증 관련 장부별 증상 계통문진(장부 변증) / 맥진, 설진, 복진, 망진
▼	
**⑦ 위험요인 문진**	정신과적 과거력, 과거 트라우마 경험, 현재 지속되는 스트레스 사건, 취약한 지지체계
▼	
**⑧ 평가 결과 설명 및 치료**	진찰 및 평가 결과를 설명.<br>고위험군은 심리지원센터나 정신건강의학과에 의뢰.<br>단계별(급성기/아급성기/만성기) 프로토콜, 증상별 프로토콜((심리증상(과긴장/공포/우울/분노), 신체증상(불면/식욕저하/무기력/두통·어지럼증/통증))을 고려하여 치료 시행.

3. 단계별 대처법 및 진료 프로토콜

재난 단계와 생존자들의 단계별 상태를 고려하여 대처하는 것이 바람직하다. 응급기, 급성기, 아급성기, 만성기와 같은 재난 후 시간경과에 따른 단계별 대처목표와 대처내용을 제시하였다.

표 12 재난 후 단계별 대처법

단계	상태	대처목표	대처방법
**응급기**<br>**재난 발생 직후 1~3일**	· 심리적 응급상태<br>· 충격, 불안, 공포, 두려움	· 현장 파악 및 진료 준비	· 재난현장의 정보 파악<br>· 의료지원팀 구성 및 현장 진료소 설치, 진료용품 준비<br>· 관련 기관에 한의진료 시행 홍보 및 의뢰 요청
**급성기**<br>**재난 발생 후 3일~1개월**	· 과긴장 혹은 심하면 공포상태<br>· 급성 스트레스 반응<br>· 충격, 불안, 분노, 절망<br>· 정서적 마비, 판단력 저하, 망연자실<br>· 일상생활 리듬 깨짐	· 과긴장/공포 상태의 안정<br>· 고위험군 파악 및 전문기관 연계	· 선별검사 : 생존자의 중증도 및 고위험군 파악하고 필요시 전문기관 연계<br>· 필요시 심리적 응급처치 우선 시행<br>· 침치료(이침 및 일반침) 시행 및 한약<br>· 호흡이완법 : 침치료시 병용<br>· 재난 후 반응에 대한 심리교육<br>· 감정자유기법(이하 EFT) 시행<br>· 자가 관리법 교육<br>· 치료빈도 : 주 3회
**아급성기**<br>**1~3개월**	· 아급성기<br>· 증상 및 고통이 뚜렷해지고 악화되는 시기<br>· 외상후스트레스장애, 우울증 발병 위험<br>· 취약층의 불안 및 분노 악화<br>· 신체 증상 발생<br>· 일상생활 리듬 깨짐	· 심리적 신체적 증상 및 고통 완화<br>· 고위험군 파악 및 전문기관 연계<br>· 신체 증상에 대한 한의학적 평가<br>· 외상후스트레스장애, 우울증 발병 예방	· 선별검사 : 외상후스트레스장애 및 심한 우울, 해리, 자살 의심시 전문치료기관 의뢰<br>· 침(이침 혹은 일반침) 및 한약치료 지속<br>· 안정화기법 : 호흡명상법, 그라운딩, 이정변기요법, 향아리요법 고려<br>· 감정자유기법(개인 혹은 단체)<br>· 자가 관리법 교육<br>· 치료빈도 : 주 2~3회
**만성기**<br>**3개월 이후**	· 심리/신체 증상의 복잡화 및 지속<br>· 고위험군의 외상후스트레스장애 및 우울증 발생<br>· 중독 등 의존 문제 발생<br>· 극단적 혼란이 지속되어 심신의 탈진상태	· 복합적 정서신체 증상의 적극적 치료 및 관리<br>· 고위험군 파악 및 전문기관 연계<br>· 재난 이전의 일상생활기능 회복 지원	· 지속되는 신체·심리증상에 대한 적극적 한약, 침치료 시행<br>· 안정화기법 : 이정변기요법, 향아리요법, 리소스 마인드풀니스 등 고려<br>· 감정자유기법 : 개인치료 및 4주 혹은 8주 단체 교육프로그램 시행<br>· 자가 관리법 교육<br>· 치료빈도 : 주 1~2회(최소 3개월 이상 지속적 치료 및 관리)<br>· 필요시 인근 한의원 및 한방병원 진료 연계

1) 급성기(재난 발생 후 3일~1개월)

❶ 대처목표

	급성스트레스로 인한 과긴장 상태의 안정
	이침치료 및 한약 등을 활용한 생존자의 증상과 고통 완화
	고위험군 파악 및 전문기관 연계

❷ 대처방법

**고위험군 선별**	트라우마 평가를 시행하여 생존자의 심각도를 파악하고 고위험군 및 기타 정신과적 응급상황이라면 전문기관에 의뢰한다.
**침치료**	손쉽게 활용 가능한 이침치료를 우선 고려한다. 생존자는 충격으로 혼란스러운 상태이므로 신체적 치료인 침치료는 상담이 어려운 생존자의 안정을 위해 우선적으로 활용가능하다. 이침은 최소한의 자극으로 통증이 적기 때문에 침자극에 공포를 가진 자에게도 시행할 수 있다. 호침을 활용하여 20~30분간 유침하되 치료공간을 확보하지 못해 유침이 어려운 경우 피내침을 활용한다. 자극을 지속시키기위해 피내침을 부착하고 지압법을 교육한다. 유침하는 동안 호흡이완법(예: 복식호흡)을 함께 시행할 수 있다. 단체로 시행할 수도 있다. 일반 침치료가 가능하다면 생존자의 증상에 따라 이침혈위 및 기타 증상별 프로토콜에 따른 혈위를 고려하여 침치료를 시행한다.
**호흡법**	간단한 호흡법을 교육하고 시행한다. 가능하면 침치료와 병용할 수 있다.
**한약**	과긴장 상태에 대한 처방을 기본적으로 고려하되, 생존자의 호소증상에 따라<br>한약(과립제/연조제)을 처방한다.
**심리교육**	재난 후 트라우마 반응에 대한 심리교육을 시행한다(치료편 / 안정화기법 중 ‘심리교육’ 참고).<br>가능하다면 효율적인 진행을 위해 단체교육을 시행한다.
**심리적 응급처치**	필요시 심리적 응급처치를 우선 시행한다.
**감정자유기법<br>(이하 EFT)**	생존자가 현재 호소하는 심리적 고통에 대해 EFT 시행을 고려한다. 급성기에는 생존자의 인지적 능력들이 떨어지기 때문에, EFT를 처음 적용하는 경우 수용확언 “나는 비록 __ 하지만, 이런 나 자신을 받아들인다”는 표현은 생존자가 받아들이기 어려울 수 있다. 생존자에게 수용확언의 개념들을 새롭게 설명, 인식시키기 어렵다면, 현 상황에 가장 필요한 “안전” 에 대한 표현들을 담은 “선택확언”을 적극적으로 활용하는 것이 생존자에게 도움이 될 수 있다.(예; 나는 지금 너무 불안하고 두렵지만, 지금 나는 안전한 마음을 느끼는 것을 선택합니다. 나는 지금 괜찮다는 마음을 온전히 받아들이기로 선택합니다. 불안하고 두려운 나를 받아들이고 다독여주는 것을 선택합니다.)
**자가 관리법 교육**	생존자에게 경혈지압 및 EFT를 교육하여 증상의 자가 관리를 돕는다. 자가지압은 이침혈위를 하루 5번(아침, 점심, 오후, 저녁, 자기전) 혈위당 20회씩 누르도록 교육한다.
**치료빈도**	주 3회 이상의 집중적 지원을 고려한다.

2) 아급성기(1~3개월)

❶ 대처목표

	다양한 심리적, 신체적 증상 및 고통이 뚜렷해지고 악화되는 시기이므로 집중적인 치료가 필요한 시기
	침, 한약 치료를 통한 증상의 적극적 관리
	신체증상의 평가 및 관리
	주기적 선별검사를 통한 외상후스트레스장애 및 우울증 발병 파악 및 전문기관 연계
	해리 증상 및 물질의존 발생에 대한 주의깊은 관찰

❷ 대처방법 및 진료프로토콜

**고위험군 선별 및 평가**	트라우마 평가(특히 PC-PTSD, PHQ-9)를 시행하여 고위험군의 외상후스트레스장애 및 우울증 발병을 선별하고 필요시 전문기관에 의뢰한다. 재진의 경우 2주에 1회 간격으로 선별검사를 시행한다.
**침치료**	불안, 공포, 우울, 분노 등 주호소증상에 따른 혈위에 침치료를 시행한다. 침치료에 적절한 공간이 없다면 이침치료를 활용할 수 있다. 유침이 어려운 경우 피내침을 활용하며 지압법을 교육한다. 또한 호소하는 개별 신체증상에 따라 개별 이침혈위를 추가로 고려할 수 있다.
**호흡법**	가능하다면 침치료와 병용한다. 복식호흡이 익숙해졌다면 호흡명상법을 시행한다. 지속시행할 수 있도록 교육한다. 지속 시행할 수 있도록 숙제를 활용하거나 호흡방법에 변화를 준다.
**안정화기법**	생존자가 호소하는 증상의 특징과 선호도를 고려하여 그라운드법, 이정변기요법, 항아리요법 등 안정화기법 중 적절한 방법을 선택하여 시행한다. 지속적으로 시행할 수 있도록 교육한다.
**EFT**	생존자가 현재 호소하는 심리적 고통에 대해 EFT를 고려한다. 선택확언 및 수용확언을 결합하여 사용하고 연속두드리기까지의 과정을 주로 활용한다. 주 1-2회 개인치료를 시행하고, 만약 생존자 자조모임을 조직할 수 있다면 주 1회는 그룹치료로 시행한다. 그룹치료는 공감하고 위로받는 느낌을 주고 생존자 간에 바로윙 베네핏(Borrowing Benefit, 빌려 쓰는 이득) 효과를 얻을 수 있다. 자가 치료법으로도 활용할 수 있도록 교육한다.
**자가 관리법 교육**	경혈지압 및 EFT를 활용한다. 필요시 이침혈위지압에 더하여 경혈지압(신문, 내관, 족삼리, 삼음교, 태충, 용천, 백회, 풍지 등)을 추가한다. 자가지압은 하루 3~5번(아침, 점심, 오후, 저녁, 자기전) 혈위당 20회씩 누르도록 교육한다.
**치료빈도**	주 2~3회의 지원을 고려한다.

3) 만성기(3개월 이후)

❶ 대처목표

	불안, 초조, 우울, 분노 등 복합적 정서 문제의 조절
	한약, 침치료 등을 통한 신체증상의 적극적 관리
	일상생활기능 회복을 위한 지원
	외상후스트레스장애 및 우울증, 물질의존 발병에 대한 주의깊은 관찰 및 전문기관 연계

❷ 대처방법 및 진료프로토콜

**고위험군 선별 및 평가**	재진 진찰 및 외상후스트레스장애, 우울증, 물질의존 평가(10분): 진료 시 관찰 및 관련 평가(PC-PTSD, PHQ-9, AUDIT)를 시행하여 관련 정신장애를 선별하여 필요시 전문기관에 의뢰한다.
**침치료**	일반침 및 이침치료를 지속 시행한다. 다양한 증상 및 생존자의 특징을 고려하여 치료혈위를 고려한다. 호흡법도 지속해서 시행한다.
**한약**	과긴장 상태에 대한 처방을 기본적으로 고려하되, 생존자의 호소증상에 따라 한약(과립제/과립제/연조제)을 처방한다.
**안정화기법**	신체/심리 증상을 모두 고려하여 한약 및 침치료를 시행한다.
**EFT**	생존자가 주로 호소하는 심리적 고통에 대해 EFT를 고려한다. 주 1회 개인치료를 시행하되, 가능하다면 주 1~2회 단체치료를 병용한다. 수용확언이 들어있는 기본과정과 선택확언을 함께 사용할 수 있다. 자가 치료법으로도 활용하도록 교육한다.
**자가 관리법 교육**	경혈지압 및 EFT를 활용한다. 필요시 이침혈위지압에 더하여 경혈지압(신문, 내관, 족삼리, 삼음교, 태충, 용천, 백회, 풍지 등)을 추가한다. 자가지압은 하루 3~5번(아침, 점심, 오후, 저녁, 자기전) 혈위당 20회씩 누르도록 교육한다. 이침혈위, 경혈지압 및 필요시 EFT를 자가시행하도록 지도한다. 도인운동법, 걷기명상법 등을 추가로 교육한다.
**치료빈도**	주 1~2회의 지속적 지원을 고려한다. 최소 3개월 이상의 지속적 관리 및 피드백이 필요하다.
**필요시 치료 연계**	적극적인 한의치료 및 한의정신요법 필요시 인근 한의원/한방병원/국공립병원/보건소에 연계한다.

그림 6 진료절차(초진, 재진) 및 예상소요시간

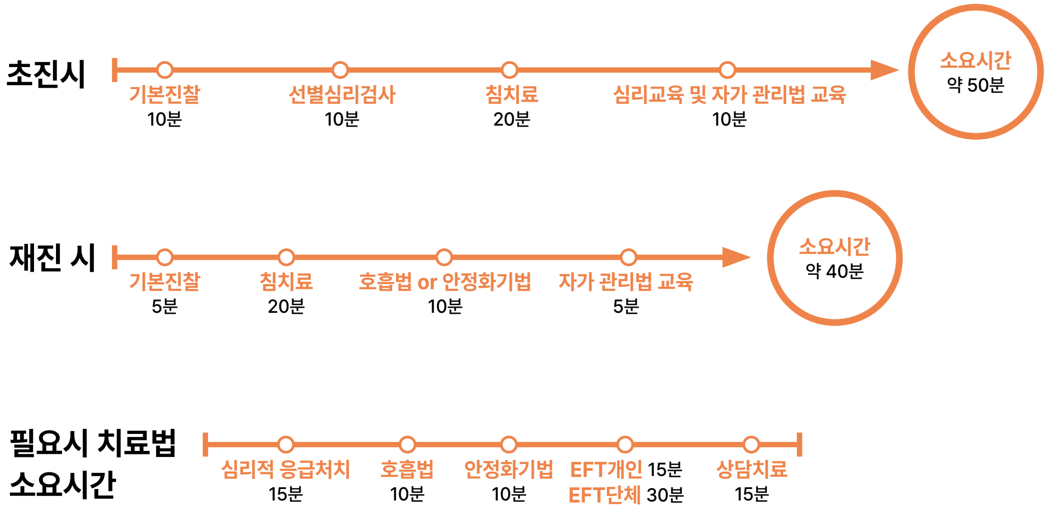

4) 평가

- 재진 시 재난 생존자가 동의한다면 SUDs 혹은 선별검사 도구를 활용하여 2주에 1회 심각도를 평가하는 것을 권고한다.
- 재진 시 평가 결과 생존자의 상태가 호전되지 않거나 악화된다면, 보다 적극적 치료를 위해 한방신경정신과 혹은 정신건강의학과 등 전문가 진료의뢰를 고려한다.

4. 증상별 진료 프로토콜

재난 생존자의 증상을 심리증상과 신체증상으로 구분하여 주호소 증상별로 중재 프로토콜(한약, 침, 지압혈위)과 증상별 대처/관리법을 제시하였다. 증상별 치료 프로토콜은 재난 현장에서 빠른 중재 선택과 개입에 도움을 줄 수 있다. 중재의 세부내용은 관련 증상의 한의임상진료지침 및 관련 근거를 바탕으로 개발팀의 토의 및 전문가 자문을 통해 제시하였다.

- 우선, 진료 및 침치료를 위한 적절한 치료 공간을 확보한다.
- 진료 현장에서는 기본적으로 증상별 진료 프로토콜을 참고하되, 생존자의 단계별 특징 및 치료목표를 고려한다.
- 한약, 침치료를 기본치료로서 고려하되, 심리적 증상을 주증상으로 호소할 때는 호흡법, 안정화기법, EFT 치료 및 상담치료를 병용할 수 있다.
- 증상이 복합적으로 나타날 때(예: 불면과 식욕부진이 함께 동반됨)는 개별 증상별 처방과 치료혈위를 함께 고려한다.
- 이침치료는 미국국립침해독협회(the National Acupuncture De-toxification Association; NADA)의 이침치료 프로토콜 기본혈위(신문, 교감, 간, 신, 폐)를 우선 고려하되[29], 재난 생존자별 신체 증상에 따라 개별 이침혈위를 가감할 수 있다(치료편. 이침치료. 증상별 혈위 참고).
- 피내침의 경우 부작용 발생 위험은 적으나, 가급적 프로그램이 진행되는 시간 동안 부착할 것을 권장한다. 부착 후 귀가시 부착 부위에 피부 이상(부종, 열감, 통증 등)이 발생하면 제거하도록 교육한다.
- 피내침 부착 부위의 통증이 지속될 때 피내침 대신 왕불류행이나 자석을 사용한다.
- 한약은 활용이 편리한 과립제(보험과립제 및 비보험과립제)를 우선 고려한다.
- **기타 치료 :** 진료현장에서 활용이 가능하다면 통증을 호소하는 생존자에게 약침치료, 전침치료, 부항, 전자뜸 및 단순추나의 적용을 고려한다.
- 경혈지압은 일 3-5회 각 혈위당 20회 이상 지압하도록 교육한다.
- **아시혈 :** 한의사는 지압을 위해 해당 근육의 혈위 및 압통점을 찾아주고, 해당부위를 자가지압할 수 있도록 교육한다.

29)미국 국립 침치료 해독협회(the National Acupuncture Detoxification Association; NADA)에서 개발한 이침 치료 프로토콜은 처음 헤로인 중독의 보조치료로 미국에서 개발된 치료법으로, 2001년 9.11 테러와 허리케인 카트리나 재난 이후 다양한 정신장애 및 재난 트라우마를 겪은 사람들에게 활용되고 있으며, NADA에서는 체계적인 훈련 프로그램을 통해 재난대응 인력들을 양성하고 있다

표 13 주증상별 진료 프로토콜 Ex*: 보험과립제, Ex: 비보험과립제, T: 탕약

심리증상					
증상 / 상황		과긴장 / 불안	공포 / 정서마비	우울 / 애도	분노 / 과민
고려할 수 있는 치료법		침(이침), 한약, 호흡법, 안정화기법, EFT			
개별치료	한약	Ex*: 시호소간산, 가미소요산 / Ex: 산조인탕, 시호가용골모려탕 / T: 온담탕, 소담해울방(소요산가감), 해울환(소요산+감맥대조탕), 황련아교탕	Ex: 계지가용골모려탕, 시호계지건강탕, 귀비탕, 천왕보심단, 우황청심원	Ex*: 시호소간탕, 가미소요산, 반하후박탕 / Ex: 귀비탕, 시호가용골모려탕, 계지복령환, 육군자탕	Ex*: 황련해독탕 / Ex: 억간산
	침	신문, 내관, 삼음교, 심수	노궁, 신문, 용천, 정혈자극(은백, 여태, 중충 등)	백회, 인당, 합곡, 태충, 족삼리	태충, 행간, 삼음교, 단중
자가 관리	지압	신문, 내관	노궁, 정혈	합곡, 백회	태충, 삼음교

신체증상						
증상 / 상황		불면 / 악몽	식욕저하 / 소화불량	무기력 / 탈진	두통 / 어지럼증	통증
고려할 수 있는 치료법		침(이침), 한약				
개별치료	한약	Ex: 산조인탕, 귀비탕, 천왕보심단, 온담탕	Ex*: 향사평위산, 반하사심탕, 불환금정기산, 삼출건비탕, 반하후박탕 / Ex: 대화중음, 내소화중탕, 육군자탕, 사역산	Ex*: 보중익기탕, 팔물탕, 가미소요산 / Ex: 귀비탕, 육미·팔미지황환/ T: 쌍화탕, 십전대보탕	두통- Ex*: 갈근탕, 청상견통탕, 반하백출천마탕 / Ex: 오령산 // 어지럼증- Ex*:반하백출천마탕 / Ex: 오령산, 당귀작약산 / T: 천마구등음가감, 영계출감탕, 자음건비탕	Ex*: 오적산, 구미강활탕, 청상견통탕 / Ex: 작약감초탕, 당귀수산 / T: 쌍화탕
	침	백회, 사신총, 안면, 신문, 삼음교, 용천, 신맥(사)조해(보)	중완, 천추, 합곡, 내관, 족삼리, 태충	태백, 족삼리, 관원, 용천, 은백	두통: 백회, 풍지, 합곡, 족임읍 어지럼증: 태충, 합곡, 현종, 백회, 풍지, 태양, 인당, 상성, 솔곡, 견정	통증부위별 혈위, 사암침 어혈방
자가 관리	지압	신문, 용천, 안면	중완, 족삼리, 천추	족삼리, 은백, 용천	태양, 풍지, 합곡, 족규음	아시혈

5. 심리적 응급처치

1) 개요

세계적으로 재난 생존자의 심리적 안정과 회복을 위하여 다양한 프로그램이 개발, 보급되어왔다. 재난에 노출된 개인에 대한 심리적 지원은 크게 재난 직후로부터 1달 이내에 이루어지는 급성기 개입, 재난 직후로부터 1~3개월 이내에 이루어지는 아급성기 개입, 심리적 문제가 3개월 이상 지속되는 경우 이루어지는 만성기 개입으로 구분할 수 있다. 이 중 급성기 재난 정신건강 지원을 위한 대표적인 프로그램인 '심리적 응급처치(psychological first aid, PFA)'는 '고통에 처해 도움을 필요로 하는 사람들에게 인도적인 도움을 주는 행위'로, 기존의 전문적인 심리 개입이 아니라 긴급 상황에서 필요한 현실적이고 직접적인 도움을 포함하는 심리·사회적 서비스를 뜻한다[30).

심리적 응급처치는 재난 직후로부터 수일까지를 뜻하는 급성기에 실질적인 도움을 제공하여 재난 생존자들의 트라우마 사건으로 인한 초기 충격을 줄이고 적응과 대처를 강화하는 것이 목적이다[31). 일반적인 응급처치가 부상자의 신체적 고통을 완화하며, 신체기능을 안정시켜 정상적으로 돌아오도록 도와주듯이, 심리적 응급처치를 통해서 생존자의 안정을 도모하며, 심리적으로 재난에 보다 잘 대처할 수 있도록 돕는다. 심리적 응급처치를 통해 재난 후 후유증을 최소화하고, 생존자들이 정상적으로 회복하는 것을 촉진한다. 심리적 응급처치의 대상은 아동과 청소년을 포함한 모든 연령과 성별을 아우르며, 문화적 차이나 재난의 다양한 종류에 상관없이 적용 가능하다[32). 심리적 응급처치는 간단한 기본 원칙으로 구성되어 있으므로 충분한 교육을 받았다면 일반인도 누구나 수행할 수 있다[33).

미국에서는 국립 외상후스트레스장애 센터(National center for PTSD)와 국가 아동 외상스트레스 네트워크(National Child Traumatic Stress Network)가 함께 현장 지침서를 개발하였다. 이 PFA 현장 지침서는 미국 허리케인 카트리나 발생 직후 초판이 나왔으며 정보와 지침이 추가된 2판이 2006년에 발간되었다. 근거 중심의 접근법을 활용하며, 테러나 천재지변과 같은 트라우마 사건 직후의 소아나, 청소년, 성인 및 그 가족들을 돕기 위해 만들어졌다. 재난과 관련된 다양한 정신건강 직종의 근무자가 활용할 수 있고, 각 연령층별로 단계적으로 구체적인 방법과 예시문을 제시하여 쉽게 활용될 수 있다. 세계보건기구가 2011년에 발행한 PFA 지침서 등 현재 전 세계적으로 약 5종의 PFA 지침서가 있다. 본 매뉴얼에서는 미국과 세계보건기구에서 발행한 PFA 지침서를 참고하여 기술하였다.

---

30)Park HI et al. Effect of Korean Version of Psychological First Aid Training Program on Training Disaster Mental Health Service Provider. J Korean Neuropsychiatr Assoc. 2020;59(2):123-135.

31)Brymer M, Jacobs A, Layne C, Pynoos R, Ruzek J, Steinberg A, Vernberg E, Watson P. Psychological first aid: field operations guide. 2nd ed. Los Angeles, CA: National Child Traumatic Stress Network and National Center for PTSD; 2006.

32)Pynoos RS, Nader K. Psychological first aid and treatment approach to children exposed to community violence: research implications. J Trauma Stress 1988;1(4):445-473

33)Dieltjens T, Moonens I, Van Praet K, De Buck E, Vandekerckhove P. A systematic literature search on psychological first aid: lack of evidence to develop guidelines. PLoS One. 2014;9:e114714

2) 심리적 응급처치의 기본 목표[34)]

① 상황과 심리적 상태를 판단하지 않고 공감적이며 인간적인 관계를 만든다.
② 최대한 빨리 안정감을 느낄 수 있도록 신체적, 정서적 지원을 제공한다.
③ 압도되어 있고 혼란스러운 상황에 있는 재난 생존자를 진정시킨다.
④ 재난 생존자에게 지금 필요한 것과 심리적 어려움을 정확하게 파악한다.
⑥ 가족, 친구 등 사회적 지원이 재난 생존자와 연결될 수 있도록 도와준다.
⑦ 긍정적인 대처 방법을 촉진하고 능동적으로 대처하도록 격려한다.
⑧ 재난의 심리적 충격에 대처할 수 있도록 심리교육을 제공한다.
⑨ 다른 지원체계와 지속적으로 소통하며 현장을 떠날 때에는 인수인계를 명확히 한다.

3) 심리적 응급처치의 대상자

심리적 응급처치는 심각한 위기 상황을 겪은 직후 고통스러워하는 사람들을 위한 것이며 아동과 어른 모두에게 제공될 수 있다. 종종 심리적 응급처치만으로는 해결할 수 없는 경우가 발생하기도 하는데 이런 경우 훨씬 더 고도화된 전문적인 수준의 지원이 요구된다. 이럴 때에는 전문가, 동료 등 다른 사람들이나 지역사회 주민 등에게 도움을 받아야 한다.
재난이나 테러 직후, 초기 고통을 줄이고 단기적 적응과 장기적 기능 회복을 돕기 위한 것으로 모든 생존자가 심한 정신적 문제를 겪게 되거나 회복에 장기적인 어려움을 보이는 것이 아니라는 가정 하에 시행하도록 한다.

4) 심리적 응급처치의 주요 구성요소

PFA의 5가지 주요 원칙은 안전감, 차분함, 연결성, 효과(대처할 수 있다는 능력과 신념), 그리고 낙관주의이다[35)]. 이런 원칙을 적용하는 것은 다른 치료와 통합될 수 있다. PFA는 신체적 안정감, 음식, 보호, 연결성, 생존과 같은 기본 욕구를 만족시키는 모든 영역과 연관된다. 생존자의 요구를 파악하고, 구호 및 회복 환경을 모니터링하고, 가정에 방문하고 정보를 전달하고 기술적 도움을 주고 상담하고 회복력을 키우고 중증도를 판단하고 조기치료를 시행하는 모든 영역을 포함한다[36)].

34)Brymer et al. Psychological first aid: field operations guide (2nc edition). National Child Traumatic Stress Network & National Center for PTSD; 2006.

35)Hobfoll SE, Watson P, Bell CC, Bryant RA, Brymer MJ, Friedman MJ, Friedman M, Gersons BP, De Jong JT, Layne CM, Maguen S. Five essential elements of immediate and mid–term mass trauma intervention: Empirical evidence. Psychiatry: Interpersonal and Biological Processes. 2007;70(4):283-315.

36)The National Child Traumatic Stress Network. PSYCHOLOGICAL FIRST AID (PFA) FIELD OPERATIONS GUIDE: 2ND EDITION. [Cited 21 Dec, 2020]. Available:https://www.nctsn.org/resources/psychological-first-aid-pfa-field-operations-guide-2nd-edition

5) 원활한 의사소통을 위해 해야 할 일과 하지 말아야 할 일[37)]

재난의료지원 시 의사소통하는 방식은 매우 중요하다. 재난 생존자들은 매우 화가 나거나 기분이 언짢을 수 있으며 자신을 탓하며 괴로워하는 경우도 있다. 자신들이 겪은 괴로운 상황에 대해 말하고 싶어 하는 사람들의 이야기를 들어주는 것은 매우 중요하다. 하지만 이야기를 강요해서는 안 된다. 너무 많은 말을 하지 말고 침묵을 허용하도록 한다. 어느 정도의 침묵은 재난 생존자들이 스스로 생각할 수 있는 여지를 마련해 준다.

표 14 재난의료지원 시 해야 할 일과 하지 말아야 할 일

해야 할 일	하지 말아야 할 일
• 가급적 조용한 장소를 찾아 대화한다.<br>• 재난 생존자의 사생활을 존중하고 비밀을 보장한다.<br>• 이야기에 고개를 끄덕이거나 호응을 하며 경청하고 있음을 표현한다.<br>• 인내심을 가지고 차분히 대화한다.<br>• 알고 있는 것과 모르는 것을 명확하게 한다<br>(예 : "그것은 제가 잘 모르는 부분이네요.<br>한번 알아보겠습니다.")<br>• 도움을 받는 사람이 이해할 수 있도록 정보를 제공하고 간결하게 말하도록 한다.<br>• 재난 생존자들의 충격과 슬픔을 잘 듣고 공감해 준다.<br>(예 : "정말 유감입니다.<br>얼마나 마음이 아프시겠어요?")<br>• 재난 생존자들의 강점과 스스로 잘 대처하고 있는 부분들을 찾아 인정하고 격려한다.<br>• 침묵을 받아주고 기다려준다.	• 이야기하라고 강요하면 안 된다<br>• 이야기 도중 끼어들거나, 재촉하면 안 된다.<br>(예 : 시계를 보거나, 빨리 말하기 등)<br>• 신체적인 접촉을 주의하도록 한다.<br>• 상대방이 한 일이나 하지 않은 일, 감정에 대해서 판단하면 안 된다. (예 : "그렇게 느끼시면 안 돼요.", "살아있는 것을 행운이라고 생각하세요.")<br>• 모르는 것을 이야기하지 않는다.<br>• 전문적인 단어를 사용하지 않는다.<br>• 다른 사람의 이야기를 하지 않는다.<br>• 지킬 수 없는 약속이나, 잘못된 확신을 하지 않는다.<br>• 재난 생존자가 처한 모든 문제를 해결해 주겠다고 생각하거나 행동하지 않는다.<br>• 재난 생존자들의 강점 및 스스로 대처할 능력과 기회를 빼앗지 않도록 한다.<br>• 재난 생존자들을 부정적으로 표현해서는 안된다.(예 : "미친 사람들", "정신 이상자" 등)

37)World Health Organization, War Trauma Foundation & World Vision International. Psychological first aid: Guide for field workers. World Health Organization; 2011

6) 심리적 응급처치의 핵심 활동[38)]

PFA의 구체적인 내용은 지침서마다 약간의 차이가 있다. 국제보건기구(WHO)의 PFA 지침에서는 ① 준비단계, ② 보고, 듣고, 연결하는 단계, ③ 마무리 단계로 나누어 기술하였다. 미국 국립외상후스트레스장애 센터의 PFA 지침은 ① 첫 접촉과 관계 형성, ② 안전과 지지, ③ 안정화, ④ 정보 수집 : 현재 요구와 고통 파악, ⑤ 실제적인 도움, ⑥ 사회 지지체계와의 연계, ⑦ 대처기술 제공, ⑧ 연계기관 안내로 구성되었다. 국내 매뉴얼들에서는 국제보건기구(WHO)나 미국 국립외상후스트레스장애 센터의 PFA 중 하나를 선택하여 기술하고 있는 편이다. 국가트라우마센터에서도 미국 PFA를 기본으로 하고 있으므로 본 매뉴얼에서는 미국 PFA 지침을 간략하게 기술하였다[39)].

❶ 첫 접촉과 관계 형성	이름, 소속, 역할, 방문 목적 등 자기소개를 하면서 공식적으로 활동한다는 것을 알려준다. 대화 시에는 미리 동의를 구하고, 당장 해결해야 할 문제나 즉각적 처치가 필요한 의학적 문제 등이 있는지 살핀다. 가능한 비밀을 보장하여야 하고, 자해·타해·학대·유기 등 법적 보고사안은 기관에 보고한다.
❷ 안전과 지지	신체적 안전을 확보하고 재난 대응 체계와 활동에 대한 정보를 제공한다. 신체적 안녕에 관심을 기울이고, 사회적 상호작용을 촉진한다. 보호자와 헤어진 아동이 있는지 파악하는 등 잃어버린 가족이 있는 재난 생존자를 지원해주며, 사별을 경험한 생존자에 특별히 주의한다. 재난 생존자가 트라우마를 떠올리는 상황에서 보호하도록 한다. 영성 이슈에 주목하고, 시신 확인이 필요한 생존자를 돕는다.
❸ 안정화	재난 생존자에게 진정과 심리적 안정을 유도한다. 재난 이후의 반응에 대한 이해를 바탕으로 재난 생존자에게 일어나는 반응을 관찰한다. 이완할 수 있도록 심호흡을 교육하여 이완을 유도한다. 지금 그리고 여기에 집중할 수 있도록 그라운딩 기법(치료편. '안정화기법' 참고)을 쓰기도 한다.
❹ 정보 수집	현재의 요구와 고통을 파악하는 것이 목적으로 각 개인에게 맞춤형 지원을 하기 위해서 생존자의 구체적인 요구와 심리적 어려움을 명확히 한다. 즉각적인 의뢰나 전문적 지원이 필요한 문제, 추가 상담 요구가 있는지에 대한 정보를 수집한다.
❺ 실제적인 도움	가장 급한 요구를 파악하고, 요구를 명료화한다. 행동 계획을 수립하고, 문제 해결을 위해 행동한다.

38)국가트라우마센터. 재난 정신건강 실무자를 위한 표준 매뉴얼. 서울: 국립정신건강센터; 2019. 58-63p.

39)The National Child Traumatic Stress Network. PSYCHOLOGICAL FIRST AID (PFA) FIELD OPERATIONS GUIDE: 2ND EDITION. [Cited 21 Dec, 2020]. Available:https://www.nctsn.org/resources/psychological-first-aid-pfa-field-operations-guide-2nd-edition

**❻ 사회 지지체계와의 연계**	가족, 친구, 지역사회 네트워크 등 사회적 지지체계와 연결해주며 다른 생존자, 지원 단체 등 가능한 지지체계를 활용한다. 소규모 그룹을 소개하여 관계를 형성한다.
**❼ 대처기술 제공**	스트레스 반응에 대한 정보를 제공한다. 이완기법이나 분노조절 방법을 알려주고 적응을 위한 긍정적 대처 방법과 부정적 감정 다루는 법을 소개한다.
**❽ 연계기관 안내**	시급한 의학적, 정신과적 문제가 있는 경우 혹은 정신건강 문제가 있는 경우, 자해·타해 위협이나 우려가 있는 경우, 알코올이나 약물 사용 문제가 있는 경우, 4주 이상 문제가 지속되는 경우, 본인이 의뢰를 요구한 경우 연계기관을 안내한다.

6. 기타 고려사항

1) 사후관리

사후관리가 재난현장에서 중요하다. 언제 다시 의료진의 서비스를 받을지 모르기 때문에 단계별 증상의 특징, 추후 진료 방법, 자가 관리법 교육, 내원 횟수, 타의료기관과의 연계 등에 대해 설명해주는 것이 필요하다.
지압을 시행하여 자기효능감을 증진하고, 안정화기법을 지속적으로 시행할 수 있도록 숙제를 내며, 자가 관리에 도움이 될 수 있는 인터넷 자료(국가트라우마센터 홈페이지 등[40]) 및 모바일 앱(국가트라우마센터앱 '마음프로그램')을 소개하고 활용하도록 안내한다.
아웃리치(방문진료) : 대피소 등으로 내원하지 못하거나 대피하지 못한 일부 피해 주민은 적절한 심리지원을 받지 못한다. 또한 재난 후 만성기 생존자들은 대피소를 떠나 가정으로 돌아가는데 노인, 장애인, 기타 취약계층의 경우 심리지원을 받기 위해 정신건강센터나 보건소 등을 정기적으로 방문하기가 쉽지 않다. 그러므로 아웃리치(outreach)나 방문진료가 필요하다. 단순 가정 방문보다는 전문화된 팀의 관리가 필요하며 해당 지역 공중보건한의사 및 지역 한의사회 연계를 통해 한의진료를 제공할 수 있다.

40) https://nct.go.kr/

2) 주의점

재난 경험에 대해 이야기하는 것은 재난 생존자에게 매우 고통스러운 과정이므로 또 다른 트라우마가 될 수 있음을 명심하여야 한다.
재난유형 : 재난 형태에 따라 지원 방법에 다소 차이가 있다. 진찰시 자연재난, 사회재난, 감염재난 등 재난 유형에 따른 생존자의 반응이 다를 수 있다는 것을 염두에 두고 접근해야할 것이다.
재난 생존자의 자살위기 가능성을 고려해야 한다.
생존자의 특성 고려 : 진료 시 생존자의 성별과 연령을 고려해서 중재법을 활용해야 한다.

3) 진료진의 대리트라우마/소진(번아웃) 예방

재난 생존자의 고통에 공감하고 이를 함께 나눌 수 있어야 하지만 지나치게 몰입하지 않도록 주의하여, 의료진이 소진되지 않도록 해야 한다. 또한, 재난 생존자가 의료진에 대해 지나치게 의존하지 않도록 주의한다.
현장에서 지원하는 한의사들의 대리 트라우마 및 소진(번아웃) 예방을 위한 전략이 진료 시스템의 일부로 포함되어야한다.

다음 대리 트라우마 및 소진 상황을 점검한다.

- 진료를 마친 후 생존자가 느꼈던 힘든 감정을 느낀다.
- 생존자의 끔찍한 충격이나 기억이 내게도 떠오른다.
- 생존자의 이야기에 집중하기 어렵다.
- 생존자에게 도움이 되지 못하는 것 같고 자신이 무능하게 느껴진다.

진료진의 자기관리

- 진료 중/후 적절한 휴식을 취하고 신체활동을 한다.
- 규칙적인 식사를 거르지 않는다.
- 장시간 연속으로 진료하지 않는다. 적절한 휴진일을 확보한다.
- 정기적으로(매일/격일/매주) 진료현장 한의사들과 함께 자신의 감정에 대해 이야기한다.

4) 재난현장 윤리지침

재난 생존자의 권익을 최우선으로 하고, 생존자에게 해가 되는 일을 삼간다.
생존자와의 진료 및 상담 내용을 동의 없이 제3자나 외부에 공개하지 않는다. 반드시 정확한 정보만 제공하도록 하고, 잘 모를 경우에는 모른다고 인정한다.
자신의 한계를 넘는 도움이 필요할 경우 해당 전문가를 찾아 자문을 구하거나 의뢰한다.

III

치료법 소개

01 이침치료

02 침치료

03 한약치료

04 감정자유기법

05 안정화기법

06 자가 관리법

III. 치료법 소개

1. 이침치료

1) 개요

오늘날 국제 임상진료지침에서는 재난트라우마에 약물치료는 권고하지 않으며, 이에 따라 현재 재난심리지원은 심리적 중재가 주로 활용되고 있다. 하지만 대규모로 생존자와 이재민이 발생하면 인적 및 물적 의료자원이 크게 소모되어 즉각적이고 효율적이며 지속적인 지원이 어렵다. 또한 장기간의 심리지원은 심리지원 제공자의 소진(번아웃)을 유발할 수 있다[41]. 그러므로 재난현장에서 활용 가능하며 효과적이고 안전한 비약물·비심리적인 중재법의 활용이 도움이 될 수 있다.

이침치료는 대표적인 한의학의 비약물요법으로 간단하게 시술할 수 있는 안전하고 효과적인 방법이며, 통증, 응급의학 분야뿐 아니라 정신장애에서도 그 유효성과 안전성이 보고되었다. 이침치료는 적용이 쉽고 빠르며, 신체 및 정신증상을 함께 개선할 수 있으며 저렴하기에 재난 진료 현장에서 활용하기에 적합하다. 해외에서는 이미 NADA 프로토콜이 전 세계의 다양한 재난현장에서 활용되고 있다[42].

표 15 재난 현장에서 이침치료 활용의 장점

재난구호 시 어려움	구체적인 문제들	이침치료의 강점
문화적 역량	언어장벽<br>원조하는 낯선 외부인에 대한 거부감<br>상담치료의 경우 지역사회 문화적 차이 및 언어장벽으로 적용이 어려울 수 있음.	비언어적 치료로 언어장벽이 없음<br>지압법 등을 지역민에 훈련하여 재난구호 제공가능
지속가능성	외부원조에 대한 의존<br>전문가의 지속적 상담은 고비용	비용이 저렴<br>지역민들이 안전하고 효과적으로 적용할 수 있도록 훈련가능<br>지역사회 회복탄력성을 북돋는 기능
서양 정신모델의 적용	DSM 진단적 접근이 적절치 않음<br>약물치료와 대화치료는 재난현장에서 적절치 않음	진단이 필요없음<br>비약물치료임<br>기존 치료에 보완, 추가 가능함

41)Mattei A, Fiasca F, Mazzei M, Necozione S, Bianchini V. Stress and Burnout in Health-Care Workers after the 2009 L'Aquila Earthquake: A Cross-Sectional Observational Study. Front Psychiatry. 2017 Jun 12;8:98.

42)Bemis R./NADA Literature Clearinghouse. Ear Acupuncture and Humanitarian Aid: History, application, and improvement of the NADA model. [Cited 10 August 2020] Available from: https://acudetox.com/ear-acupuncture-and-humanitarian-aid/

2) 근거 및 활용사례

브라질 연구진인 Corrêa 등은 최근 체계적 문헌고찰을 통해 성인과 노인의 스트레스, 불안, 우울에 대한 이침 치료의 효과를 분석하여 보고했다[43]. 총 11개의 전자 의료데이터베이스에서의 포괄적 검색을 통해 24편의 관련 임상 연구를 수집하였으며, 포함된 연구들 중 92% (22편)에서 이침치료가 스트레스, 불안, 우울에 긍정적인 효과를 보였다고 보고했다.

일차성 불면증에 대하여 이침치료의 효과를 보고하는 8편의 무작위 대조군 임상시험을 분석한 결과, 이침치료는 거짓 이침치료나 무처치군과 비교하여, 불면증 환자들의 수면의 질을 유의하게 개선시키고, 수면 잠복기를 단축시키며, 야간의 각성 횟수를 줄인 것으로 나타났다[44].

본 매뉴얼 개발 연구팀은 대규모 재난 생존자들에서 발생한 심리적 트라우마 관련 장애에 이침치료가 미치는 영향을 평가하기 위하여 체계적 문헌고찰을 시행하였다[45]. 포함된 총 10편의 임상 연구를 분석한 결과, 이침치료는 재난 후 외상후스트레스장애 환자에서 전반적인 외상후스트레스장애 관련 증상들뿐 아니라, 우울 증상과 불안 증상을 개선하는 것으로 보고되었다. 동서양을 막론하고 재난 생존자들에게 사용된 이침치료의 혈위는 유사했다. 향후 이침치료의 근거를 체계적으로 확립하기 위해서는 NADA 프로토콜과 같은 이미 표준화된 치료 전략이 사용될 수 있다.

대규모 재난 현장에서 난민이나 재난 생존자들에게 이침치료를 교육하고 해당 커뮤니티에서 광범위하게 이침치료를 실시한 사례도 있다. 침구사 Yarberry(2010)는 2007년 케냐 유혈사태로 인한 외상후스트레스장애를 겪고 있는 난민들에게 NADA 프로토콜 치료를 제공하였다[46]. 주민이 직접 이웃들에게 NADA 프로토콜 치료를 제공할 수 있도록 난민 21명을 교육하였으며 6개월 동안 18,000건의 치료를 제공하였다. 당시, 아이들이 심한 스트레스와 불안, 악몽, 야뇨증 등의 증상을 앓았는데 신문(神門)혈을 사용하거나 추가로 신(腎)혈을 사용하여 치료 후 야뇨증이 줄고 아이들이 싸우는 것도 줄었다. Yarberry(2011)는 50만 명의 사상자와 180만 명의 이재민을 발생하게 한 2010년 아이티(Haiti) 대지진으로 외상후스트레스장애를 겪고 있는 이재민들에게 NADA 프로토콜 치료 및 교육을 시행했다[47]. 24명의 주민들에게 NADA 프로토콜 치료를 교육하였으며 2,000명 이상에게 치료를 제공했다. 치료 후 이재민들은 전반적으로 기분이 나아지고 희망감이 생겼으며, 가슴 두근거림, 두통, 스트레스 등이 완화되었다.

43)Corrêa HP, Moura CC, Azevedo C, Bernardes MFVG, Mata LRFPD, Chianca TCM. Effects of auriculotherapy on stress, anxiety and depression in adults and older adults: a systematic review. Rev Esc Enferm USP. 2020 Oct 26;54:e03626.

44)Tan HJ, Lan Y, Wu FS, Zhang HD, Wu L, Wu X, Liang FR. Auricular acupuncture for primary insomnia: a systematic review based on GRADE system. Zhongguo Zhen Jiu. 2014 Jul;34(7):726-30.

45)Kwon CY, Lee B, Kim SH. Effectiveness and safety of ear acupuncture for trauma-related mental disorders after large-scale disasters: A PRISMA-compliant systematic review. Medicine (Baltimore). 2020 Feb;99(8):e19342.

46)Yarberry, M. The use of the NADA protocol for 외상후스트레스장애 in Kenya. German Journal of Acupuncture & Related Techniques. 2010 Oct 53(4):6-11.

47)Yarberry, M. NADA Training Provides 외상후스트레스장애 Relief in Haiti. German Journal of Acupuncture & Related Techniques. 2011 Jan 54(1):21-24.

뉴욕시 9·11 테러 후 많은 뉴욕시 병원들은 NADA 프로토콜을 제공하는 기존 중독치료센터에서 트라우마 생존자에게 치료를 제공했다[48]. 폭발이 발생했던 그라운드 제로에서 가장 가까이 있는 성 빈센트 병원은 NADA 프로토콜 활용 스트레스 클리닉을 열어 지역민들에게 4만 번이 넘는 치료를 제공했다. 다른 침치료 팀들은 테러 발생 직후 24시간 활동했으며 재난 처리를 담당했던 경찰관과 소방관들에게 치료를 제공했다. 침과 마사지 치료 제공팀인 CRREW (Community Rebuilding and Relief through Education and Wellness)는 9·11 테러를 계기로 결성되어 이후 뉴욕시 소방서와 구조팀에게 침치료를 제공했다. 2006년 뉴욕시 응급 의료대응 협의회(the New York City Regional Emergency Medical Response Council)는 위기 구호 경험이 있는 CRREW팀을 주요 사고발생 대응팀에 참여시켰다.

국경 없는 침구사회(Acupuncturists Without Borders; AWB)는 2005년 미국의 허리케인 카트리나 발생 직후 75명의 침구사가 모여 설립되었으며 NADA 프로토콜을 활용해 미국 및 전 세계 재난 현장에서 의료지원을 제공하는 단체이다[49]. AWB는 허리케인 카트리나 발생 당시 재난심리지원팀, 무료 진료실, 이재민 대피소, 소방관, 경찰, 군인 등과 협업하여 8천 명 이상의 사람들에게 이침치료 의료지원을 제공하였다. 이후 지진, 홍수, 총격 사건, 산불, 최근의 COVID-19까지 수백 건의 다양한 재난 사태에 치료를 제공해왔다.

국내에서도 재난트라우마에 NADA 프로토콜이 적용된 연구가 발표되었다. 대구한의대부속 포항한방병원 진료팀이 2017년 포항 지진 이후 장기간 임시보호시설에 거주하는 재난 후 생존자들에게 8주간 주2회 이침치료(피내침)를 시행한 결과 심리적 트라우마 및 우울 증상을 개선시켰다[50]. 또, 치료 후 4주 차 추적 관찰 평가에서도 개선 상태는 유지되었다. 이차 결과 지표인 수면의 질, 분노, 삶의 질에서도 8주 치료 후 일부 유의한 개선 결과가 나타났다. 치료기간 동안 이상반응은 발생되지 않았다.

---

48)Claudia Voyles, Rachel Toomim and Libby Stuyt. Acupuncture Detoxification Specialist Training Resource Manua(Fifth Edition) l: A handbook for individuals training in the National Acupuncture Detoxification Association's Five-needle Acudetox Protocol. National Acupuncture Detoxification Association;2017. 104-105p.

49)Acupuncture Without Borders. National(USA) Programs. Available from: https://acuwithoutborders.org/international-work/ [Cited 25 Jan 2021]

50)Kim SH, Kwon CY, Kim ST, Han SY. Ear acupuncture for posttraumatic symptoms among long-term evacuees following the 2017 Pohang earthquake: a retrospective case series study. Integr Med Res. 2020 Dec;9(4):100415.

3) 치료 메커니즘

생물학적으로 이침치료는 자율신경계에 영향을 미침으로써 치료 효과를 보일 수 있는 것으로 생각된다. 외이도를 자극할 때 미주신경에 의한 기침반사가 나타나는데(아놀드 반사(Arnold's Reflex)). 이 반사는 구심성 신경인 미주신경의 귓바퀴가지(auricular branches of the vagus nerve, ABVN)로 인해 발생하며, ABVN에 대한 자극은 미주신경과 유사한 반응을 유도할 수 있어 이침치료와 관련된다[51]. ABVN은 고립속계의 핵(nucleus of the solitary tract, NTS)으로 섬유를 전달하고, 뇌와 내장들 간의 NTS의 복잡한 연결을 기반으로 하여 ABVN 자극은 자율신경계를 조절할 수 있는 것으로 생각된다. 이침치료는 미주신경 활성을 높이고, 심혈관계 (심박수와 혈압 감소, 심박변이도 증가 등), 호흡기계 (후각 인식 개선, 폐활량 증가 등), 위장관계 (위장관계 운동성 조절, 복부 팽만 감소 등), 내분비계 (내분비 호르몬 조절을 통한 항상성 기여)를 조절할 수 있는 것으로 생각된다[52]. 또한 귀에는 삼차신경의 제3지인 하악신경의 분지 이개측두신경(auriculotemporal nerve)의 지배를 받는 곳이 있고, 이 경로를 통해 미주신경을 자극하거나, 뇌간 및 전뇌와 같은 특정 신경 구조물을 활성화시킬 수 있는 것으로 생각된다[53].

침치료의 범주에서 이침치료의 메커니즘을 살펴볼 수도 있다. 기존 발표된 동물실험들을 포괄적으로 수집하여 외상후스트레스장애에 대한 침치료의 효과와 메커니즘을 정리한 결과는 다음과 같다[54].

(1) 침치료는 외상후스트레스장애 동물 모델에 대하여 불안 증상과 공포 반응을 줄이고, 공포 조건화를 약화시켰다. 관련 메커니즘으로는 시상하부-뇌하수체-부신피질 축(hypothalamic-pituitary-adrenal axis)의 활동성 감소나 편도, 해마 등 뇌의 특정 부위의 가소성 증가가 보고되고 있었다.
(2) 침치료는 외상후스트레스장애 동물 모델에 대하여 우울 증상을 개선시킬 수 있었다. 관련 메커니즘으로는 해마에서 노화, 뇌 기능, 단백질 합성과 세포 성장 등 다양한 생체 기능과 관련이 있는 라파마이신 표적(mammalian target of rapamycin) 경로를 통한 가소성 증가가 보고되고 있었다.
(3) 침치료는 외상후스트레스장애 동물 모델에 대하여 수면구조에 영향을 미칠 수 있었다. 관련 메커니즘으로는 시상하부에서 사이토카인 수준의 조절 등이 보고되고 있었다.
(4) 침치료는 외상후스트레스장애 동물 모델에 대하여 공간학습과 기억의 장애를 경감시킬 수 있었다. 관련 메커니즘으로는 비정상적인 뇌파의 조절, 대뇌 피질의 혈중 산소 농도 조절 등이 보고되고 있었다.
(5) 침치료는 외상후스트레스장애 동물 모델에 대하여 과도한 스트레스 반응성을 감소시킬 수 있었다. 관련 메커니즘으로는 해마 영역의 뇌파 활성 조절, 청반(locus coeruleus)의 신경 산화질소 합성효소(neuronal NOS) 발현 감소 등이 보고되고 있었다.

51)Hou PW, Hsu HC, Lin YW, Tang NY, Cheng CY, Hsieh CL. The History, Mechanism, and Clinical Application of Auricular Therapy in Traditional Chinese Medicine. Evid Based Complement Alternat Med. 2015;2015:495684.

52)He W, Wang X, Shi H, Shang H, Li L, Jing X, Zhu B. Auricular acupuncture and vagal regulation. Evid Based Complement Alternat Med. 2012;2012:786839.

53)Mercante B, Deriu F, Rangon CM. Auricular Neuromodulation: The Emerging Concept beyond the Stimulation of Vagus and Trigeminal Nerves. Medicines (Basel). 2018 Jan 21;5(1):10.

54)Kwon C-Y, Lee B, Kim S-H. Efficacy and Underlying Mechanism of Acupuncture in the Treatment of Posttraumatic Stress Disorder: A Systematic Review of Animal Studies. Journal of Clinical Medicine. 2021; 10(8):1575.

4) 이침치료 프로토콜

NADA(National Acupuncture Detoxification Association) 프로토콜을 기반으로 하며, 본래 약물 남용의 치료를 위해 개발되었지만, 뉴욕의 9·11 테러 사건과 허리케인 카트리나 이후 심각한 트라우마를 경험한 자들에게도 도움이 된다는 것이 밝혀진 후 중독뿐 아니라 다양한 정신건강 문제에 적용되고 있다.

정신장애에 대한 NADA 프로토콜 임상 근거는 최근 10년 동안 다양한 정신장애 문제에 대해서 축적된 편인데, 중독과 관련된 연구가 가장 많고, 이 외에 불면증, 경계성 인격장애, 불안을 호소하는 화상환자, 중환자실의 의료진, 불안장애, 주요우울장애, 신경성 식욕부진증, 불안을 호소하는 제왕절개 후 산모, 지진 이재민 등 그 대상이 다양하다. 이침치료는 기존 치료와 병용되거나 단독으로 사용될 수 있으며, 연구에 따라 NADA 프로토콜에 더하여 일반침치료를 시행하거나, 혈위지압을 시행하는 경우가 있다[55].

그림 7 NADA 프로토콜: 교감, 신문, 신, 간, 폐

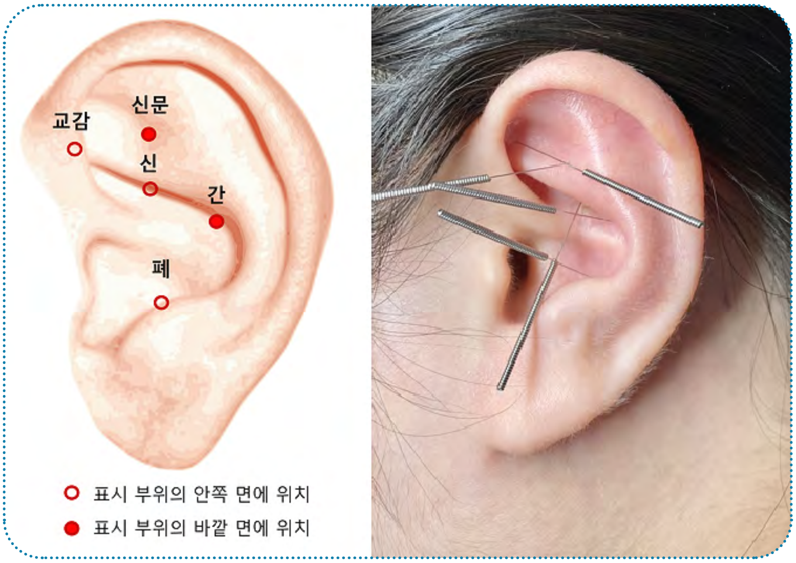

55)김다운, 김상호. 재난트라우마에 대한 이침의 활용 – NADA 프로토콜의 소개. 동의신경정신과학회지. 2020;31(3):157-168.

NADA 프로토콜의 각 혈위의 이해

'신'은 한의학에서 공포와 연관되며 특히 뇌수를 저장해 뇌기능의 원천이다. '간'은 기의 소통을 주관하여 소통이 정체되면 짜증이 나고 화가 잘 나게 된다. 간의 건강은 분노 조절과 전신 기능을 원활하게 유지하는데 중요하다. '폐'는 호흡과 면역력을 담당하며 특히 우울증과 연관된다. '신문'은 정신의 통로로 심장과 연관되며 불안/긴장을 다스리고, 차분함과 평안, 이완 효과를 나타낸다. '교감'은 교감신경/부교감신경의 균형을 조절해 주고, 강한 진통 효과 및 비/위와 연관되어 내장기관의 혈관확장으로 소화를 촉진하고, 간과 함께 짜증과 공격성을 감소시킨다. 이압요법(이침혈위 자극요법)을 교육할 때 위와 같은 혈위의 특성을 함께 설명해 준다면 환자는 더 적극적으로 활용하게 될 것으로 생각된다.

- **시술 절차 :** 시술자는 시술에 앞서 비누나 알코올 젤 등을 사용하여 손을 깨끗이 씻고 말린다. 이후 일회용 알코올 스왑을 사용하여 환자의 혈위 국소부위를 소독한다. 이후 깨끗이 소독된 핀셋 등을 사용하여, 일회용 무균 피내침을 환자의 편측 치료 혈위에 부착한다. 환자들에게는 하루 3~5번씩 (아침에 일어났을 때, 아침, 점심, 저녁 식사 후, 취침 전) 각 혈위에 부착된 피내침을 20회 정도 지압하도록 권고한다. 다음 치료일에는 시술자가 핀셋 등을 사용하여 기존에 부착된 피내침을 제거한 뒤, 국소 피부의 이상반응 여부를 확인하고 일회용 알코올 스왑을 사용하여 환자의 혈위 국소부위를 소독한다. 이후 반대 측 치료 혈위에 같은 방법을 사용하여 이침치료를 실시한다.

- 일반적으로 NADA 프로토콜 적용은 기존 이침치료와 다르지 않으며, 호침을 이용하여 0.3 cm 정도 자입하고, 30~45분의 유침기간을 거친다. 자침 외에 왕불류행 씨앗이나 구슬, 또는 피내침을 혈위에 붙여 지압할 수도 있다.

- **시술 빈도 :** 이침치료는 매주 2회 실시한다.

증상별 개별 혈위[56)]

이침혈위 선정시 생존자의 주호소 증상별로 다음의 혈위(공통혈위 및 개별혈위)를 고려할 수 있다.

표 16 증상별 개별 혈위

근골격계 통증			
공통혈위1	공통혈위2	부위	개별혈위
간, 신, 신문	대뇌피질(소뇌)	두통	대뇌피질(피질하)
		경항통	후두(침), 경추, 경
		요둔통	요천추
**내과 질환**			
간, 신, 신문	교감	소화기	위, 대장
		호흡기	폐
		비뇨생식기	방광
		순환기	심
	내분비	산부인과	내생식기(자궁)
		오관과	눈, 내이, 내비, 구, 인후
		피부과	폐
**기타**			
금주		금연	신문, 내분비, 폐, 인후, 내비
		후두, 대뇌피질, 액, 간양	

5) 안전한 시술 및 주의점

이침치료는 기본적으로 매우 안전한 치료로 생각된다. 하지만 안전한 시술 절차를 지키지 않는다면 일부 부작용이 발생할 수 있다. 2014년에 발표된 체계적 문헌고찰에서 이침치료와 관련된 부작용을 보고한 문헌들을 포괄적으로 수집하여 분석한 결과, 이침치료와 관련하여 가장 자주 보고된 이상반응은 침 자입 시 통증, 현기증, 국소 불편감, 경미한 출혈, 오심 등이었다. 또한, 바늘을 자입하지 않는 혈위지압도 국소 피부 자극 및 불편감, 경미한 압통 및 통증, 현기증 등의 이상반응이 흔히 보고되었다. 하지만 이러한 이상반응은 대부분 일시적이고 경미하며 견딜 수 있는 수준이었고, 심각한 부작용은 확인되지 않았다[57)].

장기간 피내침 부착의 경우에는 감염과 관련한 주의가 더 필요하다. 2020년에 발표된 문헌고찰에 따르면, 귀에 부착한 장기간 피내침으로 인해 감염, 연골막염, 연골염 등의 사례가 있었다.

56)대한침구의학회 교재편찬위원회. 침구의학. 서울(한미의학):2020.

57)Tan JY, Molassiotis A, Wang T, Suen LK. Adverse events of auricular therapy: a systematic review. Evid Based Complement Alternat Med. 2014;2014:506758.

그리고 그 주요 원인은 오염된 도구의 사용으로 추정되었다. 이에 저자들은 왕불류행(王不留行)과 같은 씨앗을 사용하는 것이 감염 위험을 줄이면서도 임상적 이점이 있음을 제안하고 있다[58). 그러므로 재난 현장에서 이침치료의 활용에는 안전한 시술 절차가 준수되어야 할 것이다[59).

이침치료 프로토콜의 안전성 절차

- 생존자의 혈압이 140/90 mmHg 이상의 고혈압인 경우 이첨방혈 등의 추가처치를 고려할 수 있으며, 180/120 mmHg 이상의 고혈압성 응급인 경우 진료의뢰 혹은 응급의료센터 후송이 필요하다. 또한 발열 또는 기침 등 호흡기계 감염 증상이 있는 경우에는 이침치료를 실시하지 않는다.
- 생존자의 귀에 손상이 있거나, 치유되지 않은 상처가 있거나, 흉터, 동상, 또는 기형 등이 있는 경우에는 이침치료를 실시하지 않는다.
- 피내침 부착 시 대상자들에게 30분 동안 휴식을 취하도록 권고하며, 휴식 후에도 어지럼증이나 오심이 있으면 피내침을 제거한다.
- 피내침 부착 후 생존자가 이로 인한 통증, 열감, 붓기를 경험할 때는 스스로 피내침을 제거하도록 하며, 생존자의 불편감이나 이상반응 발생 시 연락할 수 있는 연락수단을 마련한다.
- 시술자는 매 방문 시 치료 혈위의 감염이나 출혈 징후를 주의 깊게 살피며, 감염 징후가 있는 경우 보건소나 병원진료를 안내하여 진통소염제나 항생제 등 적절한 대증요법을 받도록 한다.

침구의학 교과서[60)의 이침치료 주의점

- 자세한 문진과 정확한 진단 후 시행한다.
- 최소의 혈위로 최대의 효과를 얻도록 고민한다. 보통 3~5개 혈위를 사용한다.
- 시술 전 환자에게 잘 설명하고 이해시켜 긴장하지 않도록 해서 자침 시 통증을 감소시키도록 노력한다.
- 귀를 잘 소독해야 한다. 소독이 불충분하면 피부감염이 발생할 수 있으며, 간혹 연골에까지 파급되면 예후가 불량해질 수 있다. 감염 발생 시 항염증 치료(항생제 등)를 시행한다.
- 귀에 염증이 있거나 흉터, 조직 변형이 있는 부위는 자침을 금한다.
- 유침 중 갑작스러운 다른 부위 통증, 시큰거리고 뻐근함같은 불쾌감 발생 시 침을 뺀다.
- 훈침 예방을 철저히 하고, 훈침 발생 시 적절히 처치와 안정을 시행한다.
- 이침과 체침을 함께 놓을 때 먼저 이침치료를 실시하고 체침치료를 실시한다. 체침으로 증상이 경감되면, 이침혈위의 압통점을 선별하기 어렵기 때문이다.
- 임신 초기(~14주) 임산부에서는 시술을 피한다. 임신 중기 이후에도 습관성 유산 경험이 있는 임산부는 이침 시술을 하지 않는다. 피내침 유지보다는 일정 시간 유침 후 발침한다. 피내침보다는 왕불류행 등 씨앗을 활용하고 유지기간을 1-2일 정도로 짧게 하고 지압시 약자극한다. 유산/조산을 예방하기 위해 내생식기, 내분비, 복, 골반강 등 혈위는 금기이다.
- 과도한 피로, 영양섭취 불량, 신체가 극히 허약한 사람, 심한 빈혈 환자는 금기이다. 만약 시행해야 한다면 편하게 누운 자세에서 훈침을 예방하고, 혈압이 불안정한 노인 환자는 자침 또는 이첨, 강압점 방혈 시 30분 정도 휴식 후 옆으로 누운 자세에서 자침한다. 자침 후 30분 정도 안정시키면서 관찰한다.

---

58) Nielsen A, Gereau S, Tick H. Risks and Safety of Extended Auricular Therapy: A Review of Reviews and Case Reports of Adverse Events. Pain Med. 2020 Jun 1;21(6):1276-1293.

59) Kim SH, Kwon CY, Kim ST, Han SY. Ear acupuncture for posttraumatic symptoms among long-term evacuees following the 2017 Pohang earthquake: a retrospective case series study. Integr Med Res. 2020 Dec;9(4):100415.

60) 대한침구의학회 교재편찬위원회. 침구의학. 서울(한미의학):2020;249-250p.

6) 타 치료와의 병용

재난 현장에서 이침치료는 다른 치료법과 병용하여 사용가능하다. 이침치료는 신체적 치료이므로 트라우마 치료에 흔히 사용되는 노출요법(exposure therapy)의 적용이 어려운 환자들에게 고려될 수 있다.
침치료는 심리적 증상과 통증 등 신체적 증상을 함께 관리할 수 있으며, 다른 치료와 병행 치료하기 편하다는 장점이 있다.

2. 침치료

한의학의 주요 치료법인 침치료를 재난트라우마에 활용한 다양한 연구 및 사례들이 있다. 침치료는 생존자의 다양한 심리적, 신체적 증상에 적용할 수 있으며, 다른 치료와 병용이 쉬우며 비약물치료이기에 안전하다. 일본에서는 대형 재난 발생 시 재난의료지원팀(the disaster medical assistant team), 일본의료지원팀(Japan Medical Assistant Team) 등이 서양의학 치료를 담당하는데, 일본 일차케어협회의 재난구호 프로젝트(the Japan Primary Care Association Disaster Relief Project)[61], 재난 침뜸마사지 구호 프로젝트팀(Disaster Acupuncture, Moxibustion and Massage Relief Project)[62], 아시아 의사협회(Association of Medical Doctors of Asia)[63]에서는 서양의학 치료와 함께 구호 활동에 침치료를 활용한다.

1) 근거 및 사례

중국 절강성의 동덕병원 심리구호팀은 2008년 5월 20일부터 31일까지 91명의 급성스트레스장애로 진단된 지진 트라우마 환자(평균연령 35.01±19.32세)를 인지행동치료군 24명과 인지행동과 전침 병용치료군 67명으로 나누어 전침치료를 노궁혈에 1주일간 매일 30분간 50hz로 치료하였다[64]. 노궁혈은 심포경락에 속하며 심장부위 통증, 울분, 구토, 의식저하 등에 사용하는 구급혈이다. 치료 결과 인지행동치료와 전침 병용치료가 트라우마를 떠올렸을 때 발생하는 정서적 반응과 외상후스트레스장애 증상을 유의하게 개선시켰고, 병용치료는 단독치료보다 효과적이었다. 전침 병용치료는 인지행동 단독치료군보다 치료효과가 빨리 나타났다. 인지행동치료에 전침 병용치료가 효과적인 재난트라우마 급성기 치료법으로 사용될 수 있음을 시사한다.

61)Japan Primary Case Association-Primary Care for All Team[Internet]. [cited 2020 Dec 17]. Available from https://primary-care.or.jp/jpca_eng/activities.html

62)Disaster Acupuncture and Massage Project[Internet]. [cited 2020 Dec 17]. Available from http://sinkyu-sos.jimdo.com

63)Association of Medical Doctors of Asia[Internet]. [cited 2020 Dec 17]. Available from http://amda.or.jp/

64)Zhang Y, Feng B, Xie JP, Xu FZ, Chen J. Clinical study on treatment of the earthquake-caused post-traumatic stress disorder by cognitive-behavior therapy and acupoint stimulation. J Tradit Chin Med. 2011 Mar;31(1):60-3.

일본 도호쿠대학 대학원 전통의학과 의료진은 동일본대지진 피해 이재민이 거주하는 미야기현과 후쿠시마현의 7곳의 대피소를 방문하여 의료지원(침과 마사지)을 시행하였다[65]. 도호쿠대학 의료팀과 전통의학과 의료진이 함께 의료지원을 시행했는데. 침과 마사지 병용치료에 대해 이재민뿐 아니라 의사들의 지원 요청도 많았다. 총 553명이 치료받았고 가장 흔한 증상은 견배부 강직이었다. 치료 만족도는 92.3%였고 대부분 신체뿐 아니라 정신적으로도 편안해졌다고 응답했다. 침과 마사지 병용치료가 시작됐을 때 수돗물이 충분히 대피소에 공급되지 않아 이재민들은 목욕은 물론 손을 제대로 씻을 수도 없었다. 이 기간에는 감염 위험 때문에 마사지 치료만 시행하고 침치료는 보류했다. 재난 시 침과 마사지 병용치료는 신체와 정신을 함께 안정시켰고, 다른 양의학적 치료와 함께 효과적으로 사용 가능했다.

일본 침뜸마사지 구호프로젝트 팀(Disaster Acupuncture, Moxibustion and Massage Relief Project)은 2011년 동일본 대지진 이재민(2011년 3월 27일~2011년 12월 5일)과 2015년 홍수 이재민들(2015년 9월 19일~2015년 10월 23일)에게 의료지원(침과 마사지 병용치료)을 시행했다[66]. 치료를 받은 동일본 대지진 이재민 1042명과 홍수 이재민 110명의 차트를 후향적으로 분석한 결과, 장기간 불편한 대피소 생활을 하다 보면 통증 관련 문제가 발생하기 쉽기 때문에 가장 많은 호소증상은 견배통, 슬통(지진 이재민에서 67.6%, 홍수 이재민의 80.9%)이었다. 침과 마사지 병용치료 시행 후 시각통증척도(VAS) 상 이재민과 자원봉사자들의 통증이 유의하게 감소되었다(치료 전 VAS 3.0 vs 치료 후 VAS 1.0, $p<0.001$). 또한 일본 침뜸마사지 구호프로젝트팀에서 개발한 침치료 재난의료지원 가이드라인(2016년 4월 25일 발표)이 소개되었는데 1) 자원봉사 기구 조직, 2) 피해지역 사람들과의 소통, 3) 자료관리, 4) 시술과정 설명, 5) 타 지원팀과의 협력, 6) 필요장비와 시설, 7) 침치료나 마사지 치료를 시행하면 안 되는 경우, 8) 심리상담법, 9) 시술 중 부작용 발생 대처에 대한 내용이 담겨있다.

2016년 8월 24일 진도 6의 강진이 이탈리아 중부를 강타하여 아마트리체와 여러 마을들을 파괴시켜 300명이 사망하고 3만 명의 이재민이 발생했다. 롬바르도 침구사협회(The Lombard Association of Medical Acupuncturists)와 세계 침치료협회(Acupuncture in the World) 구호팀은 지진 피해 이재민에게 2016년 9월부터 10월까지 5주간 침치료를 시행하였다. 이 중 41명에 대한 침치료 재난 구호활동 결과를 분석하여 생존자의 불안, 우울, 불면 같은 정신과적 증상과 통증에 대한 침치료 효과가 보고되었다[67]. 4일간 연속으로 총 4번의 치료를 시행했고 뜸치료도 병용했다. VAS를 사용하여 통증과 전반적 심리상태에 대해 평가한 결과, 3번의 치료 후 분석에 포함된 총 41명 중 54%에서 심리증상이, 60%에서 통증이 뚜렷하게 호전되었고 심각한 부작용은 없었다. 이런 결과는 재난 후 응급상황에서 침치료는 심리증상과 동반된 신체증상(통증)을 함께 치료할 수 있기에 대규모 재난 발생 초기에 효과적으로 활용가능한 치료법임을 보여준다.

---

65) Takayama S, Kamiya T, Watanabe M, Hirano A, Matsuda A, Monma Y, Numata T, Kusuyama H, Yaegashi N. Report on disaster medical operations with acupuncture/massage therapy after the great East Japan earthquake. Integr Med Insights. 2012;7:1-5.

66) Miwa M, Takayama S, Kaneko S. Medical support with acupuncture and massage therapies for disaster victims. J Gen Fam Med. 2017 Dec 16;19(1):15-19.

67) Moiraghi C, Poli P, Piscitelli A. An Observational Study on Acupuncture for Earthquake-Related Post-Traumatic Stress Disorder: The Experience of the Lombard Association of Medical Acupuncturists/Acupuncture in the World, in Amatrice, Central Italy. Med Acupunct. 2019 Apr 1;31(2):116-122.

외상후스트레스장애에 대한 16개 중의학 문헌 검토 결과(단일군 전후 비교 연구 4편, 대조군 임상 연구 12편), 외상후스트레스장애 환자에 한약뿐 아니라 일반침, 전침, 이침 및 침뜸 병용 치료, 심리치료와 침 병용 치료 등 다양한 중의학 치료가 활용되었으며 양약과 중의학 치료를 병용하는 경우가 많았다[68]. 전침, 체침, 이침, 완과침, 뜸 치료가 활용되었는데 전침이 가장 많이 사용되었다. 대부분의 연구에서 중의학 치료는 대조군보다 유의하게 좋은 효과를 보이며, 양약 치료군과의 비교결과 효과가 동등하거나 더 좋았다.

2018년 발표된 외상후스트레스장애에 대한 침치료 효과에 대한 체계적 문헌고찰에서는 포함된 7개의 연구(709명)에 대한 분석 결과, 침치료는 대조군 치료보다 치료 후 및 추적조사 시에도 외상후스트레스 증상에 효과적이었으며 우울증 개선에도 효과적이었다. 또한 침치료와 관련된 부작용은 거의 없었다[69].

외상후스트레스장애에 대한 한의학 치료 임상연구 동향 연구에서 국내 한의학 치료를 사용한 8편의 연구가 확인되었다[70]. 5편이 증례 및 증례군 보고였고, 단일군 전후 비교 연구가 2편, 준 무작위 대조군 연구가 1편이었고, 외상후스트레스장애를 유발했던 사건으로는 교통사고, 폭행, 성폭력, 가족 자살 목격 등이 있었다. 침, 한약, 뜸, 부항, 추나, 물리치료, 기공요법, 한의정신요법(M&L 트라우마 심리치료 프로그램) 등 다양한 치료법이 사용되었다.

2) 임상적 고려사항

대피소에서 물이 공급되지 않아 목욕이나 손을 씻을 수 없으며 손소독제 등도 사용할 수 없는 경우 침치료와 연관될 수 있는 감염을 주의한다. 대피소에서 전침기를 활용가능하다면 증상별 혈위에 전침치료 시행을 고려한다. 전침은 연속파로 생존자가 견딜 수 있는 강도로 시행한다.

❶ 과긴장/불안[71]	신문, 내관, 족삼리, 백회, 사신총, 신정, 풍지
❷ 공포[71]	노궁, 신문, 용천 / 필요시 십정혈(은백, 여태, 중충 등)을 자극한다.

68)최유진, 권찬영, 장재순, 정하영, 김윤나, 정선용. 외상후스트레스장애 치료에 대한 중의학 임상연구 동향. 동의신경정신과학회지. 2016;27(3):197-206.

69)Grant S, Colaiaco B, Motala A, Shanman R, Sorbero M, Hempel S. Acupuncture for the treatment of adults with posttraumatic stress disorder: A systematic review and meta-analysis. Journal of Trauma & Dissociation. 2018 Jan 1;19(1):39-58.

70)주성준, 권정은, 권찬영, 이보람, 김상호. 외상후스트레스장애 치료에 대한 한의학 임상연구 동향. 동의신경정신과학회지. 2019;30(3):251–63.

71)김근우. 불안장애 한의표준임상진료지침. 한의표준임상진료지침개발사업단. 대한한방신경정신과학회; 2017. 1-57p.

❸ 우울/애도[72]	백회, 인당, 합곡, 족삼리<br>* 간기울결- 태충, 합곡, 신문, 내관; 기울화화- 행간, 협계, 소부; * 담기울결- 풍륭, 양릉천, 중완; * 심비양허- 삼음교, 족삼리; * 간신음허- 태계, 삼음교; * 음허화왕- 태계, 조해
❹ 분노/과민[73]	태충, 행간, 삼음교
❺ 불면/악몽[74]	백회, 사신총, 신정, 안면(安眠:예풍과 풍지혈 사이 1/2), 신문, 내관, 신맥, 조해, 삼음교, 족삼리, 태충 등.<br>* 심비양허- 신문, 족삼리 ; * 심신불교- 조해, 노궁 ; * 간울화화- 태충, 협계
❻ 식욕저하/소화불량[75][76]	중완, 천추, 합곡, 내관, 족삼리, 태충, 공손
❼ 무기력/탈진[77]	태백, 족삼리, 관원
❽ 두통[78]	백회, 풍지, 합곡, 족임읍<br>* 근위 취혈 : 백회, 풍지, 사신총, 태양, 인당, 솔곡, 현로, 각손, 천주, 두유, 사죽공, 곡빈, 함염, 현리, 신정 및 아시혈 등<br>* 원위 취혈 : 합곡, 태충, 내정, 곤륜, 족임읍, 곡천, 행간, 열결, 풍륭, 양릉천, 구허, 외관, 중저, 태계 등
❾ 어지럼증[79]	백회, 사신총, 풍지, 태양, 인당, 상성, 솔곡, 견정, 합곡, 태충, 현종<br>* 풍담- 풍륭, 내관; * 음허양항- 태계, 간수; * 간화상염- 행간, 태충;<br>* 기혈휴허- 족삼리, 혈해
❿ 통증: 통증부위별 혈위	경항통, 견배통-견정, 풍지, 대추, 견외수, 천료, 곡원, 천종, 비노, 거골, 중부<br>요통[80]-요양관, 명문, 신수, 대장수, 상료, 차료, 위중, 곤륜, 양릉천, 삼음교<br>슬통-양릉천, 음릉천, 족삼리, 양구<br><br>사암침 어혈방(태백 태연 +, 곡지 외관 -)을 고려할 수 있다.

72)김종우. 우울증 한의임상진료지침. 한의학연구원. 한방신경정신과학회; 2016. 168p.
73)정선용. 화병 한의표준임상진료지침. 한의표준임상진료지침개발사업단. 대한한방신경정신과학회; 2019. 53p
74)김보경. 불면장애 한의표준임상진료지침. 한의표준임상진료지침개발사업단. 대한한방신경정신과학회; 2017. 1-50p.
75)김진성. 기능성소화불량 한의표준임상진료지침. 한의표준임상진료지침개발사업단. 대한한방내학회; 2020. 3-6p.
76)김진성. 기능성 소화불량 한의표준임상진료지침. 한의표준임상진료지침개발사업단. 대한한방내과학회; 2017. 1-53p.
77)Zhang Q, Gong J, Dong H, Xu S, Wang W, Huang G. Acupuncture for chronic fatigue syndrome: a systematic review and meta-analysis. Acupunct Med. 2019 Aug;37(4):211-222.
78)이상관. 편두통 한의표준임상진료지침. 한의표준임상진료지침개발사업단. 대한중풍순환신경학회; 2017. 1-28p.
79)이의주. 현훈(어지럼증) 한의표준임상진료지침. 한의표준임상진료지침개발사업단. 사상체질의학회; 2021. 68p.
80)남동우. 만성요통증후군 한의표준임상진료지침. 한의표준임상진료지침개발사업단. 대한침구의학회; 2020. 65p.

3. 한약치료

한의학의 주요 치료법인 한약을 재난트라우마에 활용한 다양한 연구 및 사례들이 있다. 한약치료는 다양한 증상에 대처할 수 있으며 부작용이 적고 의존 위험성이 없다. 따라서 한약치료는 정신과 약물치료가 적절하지 않은 생존자에게 대안적으로 활용할 수 있다. 또한 한약은 일반적 의학적 처치가 어려운 탈진, 식욕저하, 저체온증 등에도 활용가능하다. 특히 한약 과립제는 준비, 운반, 보관, 용량 조절 등이 용이해 재난현장에서 활용도가 높다.

1) 근거 및 사례

일본 도호쿠대학 한방의학과 연구진은 재난 후 한약사용에 대한 체계적 문헌고찰을 시행하였다[81]. 검토 결과 2개의 무작위 대조군 연구, 3개의 후향적 관찰연구, 7개의 증례 연구를 확인하였고, 포함된 연구들은 재난현장에서 한약의 활용가능성을 보여주었다. 한약은 외상후스트레스장애 및 기타 재난 후 일반 신체증상 및 다양한 심신의학적 증상(감기, 편도염, 체온 저하, 장염, 비염, 눈 가려움, 안구 건조, 변비, 전신소력, 복통, 하지부종, 하지저림, 어지럼증, 불면, 불안, 짜증)에 사용된 것으로 보고 있다.

일본 국립 센다이-니시타가 병원 연구진은 동일본 대지진 생존자 중 사건충격척도(Impact of Event Scale – Revised, IES-R) 25점 이상에 해당하는 2년 이상 경과한 만성 외상후스트레스장애 외래 환자 48명을 대상으로 시호계지건강탕을 시험군에게 2주간 1일 3회 투여하였으며, 대조군은 치료를 받지 않고 대기하였다[82]. 2주 치료 후 한약 복용군은 대조군에 비해 트라우마 척도의 불안 및 트라우마의 반복되는 회상(플래시백), 회피반응 등이 유의하게 개선됐으며, 부작용이나 혈액검사상 이상 소견도 없었다. 즉 시호계지건강탕은 재난 생존자의 만성 트라우마 증상 완화에 효과적이었고 안전했다. 이같은 근거를 바탕으로 시호계지건강탕은 국제트라우마스트레스 학회의 외상후스트레스장애 가이드라인(2018)에 비심리적 치료법으로도 소개되고 있다[83].

---

81)Takayama S, Kaneko S, Numata T, Kamiya T, Arita R, Saito N, Kikuchi A, Ohsawa M, Kohayagawa Y, Ishii T. Literature Review: Herbal Medicine Treatment after Large-Scale Disasters. Am J Chin Med. 2017;45(7):1345-1364.

82)Numata T, Gunfan S, Takayama S, Takahashi S, Monma Y, Kaneko S, Kuroda H, Tanaka J, Kanemura S, Nara M, Kagaya Y, Ishii T, Yaegashi N, Kohzuki M, Iwasaki K. Treatment of posttraumatic stress disorder using the traditional Japanese herbal medicine saikokeishikankyoto: a randomized, observer-blinded, controlled trial in survivors of the great East Japan earthquake and tsunami. Evid Based Complement Alternat Med. 2014;2014:683293.

83)ISTSS Guidelines Committee. Posttraumatic stress disorder prevention and treatment guidelines methodology and recommendations. International Society for Traumatic Stress Studies (ISTSS).[Google Scholar]. 2018:p28.

중국 상해장정병원에서는 2008년 규모 8.0의 쓰촨 대지진 발생 5개월 후 외상후스트레스장애 진단 생존자에게 한약 투여 무작위 대조군 이중맹검 연구를 시행했다[84]. 실험군(123명)은 소담해울방(消痰解郁方)[85]을 복용했고, 대조군(122명)은 플라시보 한약을 하루 2번 총 8주간 복용하였다. 소담해울방 복용군은 대조군에 비해 전반적인 심리상태를 보는 간이정신진단검사(Symptom Checklist-90-Revision, SCL-90-R) 척도와 치료반응률이 유의하게 개선되었으며 심각한 부작용 보고는 없었다. 특히 소담해울방 복용군은 불안뿐 아니라 수면상태도 개선되었다.

2014년 세월호 침몰 사건 후 3곳에서 대한한의사협회는 24시간 3교대로 한약을 이용해 의료지원을 시행했다[86]. 한의치료를 받은 총 1860명의 환자는 대부분 통증을 호소했지만 또한 소화불량, 감기, 두통, 어지럼증, 탈진, 불안, 불면 등 다양한 증상을 호소했다. 특히 환자들은 통증 다음으로 정신과적 증상과 탈진 때문에 한의진료를 선택했다. 의사들은 환자에 대한 응급치료를 담당했고, 한의사는 주로 구호요원들을 치료했다. 가장 많이 처방된 약은 쌍화탕, 소합향원, 오적산이었다. 맥문동탕, 소청룡탕이 콧물, 코막힘, 마른 기침을 호소하는 구호요원들에게 효과적이었다.

일본 도호쿠대학병원 한방내과 연구진은 동일본 대지진 발생 후 피해가 컸던 미야기현 오나가와정, 이시노마키시의 대피소에서 시행된 10주간 한약치료를 받았던 236명의 한방진료 차트를 후향적으로 조사, 분석했다. 지진 후 2주간 매우 추웠지만 난방시설이 부족하여 생존자들은 감기 증상과 저체온 등이 많아 감기 증상에 갈근탕과 계지탕을 사용하고, 저체온증에는 인삼탕과 당귀사역가오수유생강탕을 사용했다. 특히 기초대사가 저하된 저체온 노인에게 몸을 따뜻하게 하는 한약이 효과적이었다. 청결한 물의 확보가 어려워 손을 씻지 못하는 위생환경 때문에 위장염 증상이 나타나 정장제와 지사제로 개선되지 않는 증례에 오령산을 처방했다. 2주부터 6주까지는 쓰나미로 운반된 헤도로(하수와 공장 폐수 등이 해변가에 질척하게 굳어진 것)와 토사 등이 건조되어 공중에 떠올라 공기오염이 심해져서 심한 기침과 콧물, 안구 소양감 등 알레르기 및 호흡기 증상이 많아져서 소청룡탕과 맥문동탕 등을 처방했다.

84)Meng XZ, Wu F, Wei PK, Xiu LJ, Shi J, Pang B, Sun DZ, Qin ZF, Huang Y, Lao L. A chinese herbal formula to improve general psychological status in posttraumatic stress disorder: a randomized placebo-controlled trial on sichuan earthquakesurvivors. Evid Based Complement Alternat Med. 2012;2012:691258.

85)소요산가감방. 소요산(시호, 당귀, 복령, 백출, 백작약, 박하, 자감초, 생강)에서 생강을 빼고, 7개 약제(황련, 반하, 진피, 용골, 모려, 대황주증, 석창포)를 추가함.

86)Kim KH, Jang S, Lee JA, Jang BH, Go HY, Park S, Jo HG, Lee MS, Ko SG. Experiences Providing Medical Assistance during the Sewol Ferry Disaster Using Traditional Korean Medicine. Evid Based Complement Alternat Med. 2017;2017:3203768.

6주부터 10주까지 기간에는 긴 대피소 생활로 인한 스트레스로 불면, 초조, 불안 등 정신적 증상과 다양한 신체증상 호소가 증가하여 억간산, 가미귀비탕 등 안정작용이 있는 한약을 처방했다[87]. 노인에게 강한 수면유도제를 쓰면 몽롱해져서 여진 때 대피하기가 어렵고 낙상 위험성이 발생하는데, 한약은 가벼운 진정 작용으로 수면을 개선하면서 근긴장을 저하시키지 않는 장점을 가지고 있다. 재난 초기에 노인의 저체온 증례가 많았고, 6주부터는 특히 정신증상을 호소하는 사람들이 많았다. 많은 이재민들에게 이미 양약이 처방되고 있었지만 한약이 추가되어 증상 개선이 보이는 증례가 많았다.

대한한의사협회는 유행 초기 코로나19가 대규모로 발생한 대구에 '코로나19 한의진료 전화상담센터'를 운영하였다[88]. 전화상담센터의 자원봉사 한의사는 코로나19 확진 환자에게 처방된 청폐배독탕을 기본적으로 처방하였지만, 호흡기 증상이 경미한 환자에게는 동반 증상에 따라 다양한 한약들을 처방하였다. 비염에 처방한 소청룡탕과 불안 및 불면에 처방한 가미귀비탕이 대표적이었다. 호흡기 증상이 경미하였지만 많은 확진자가 질병 악화에 대한 불안과 격리 때문에 불안, 우울, 불면, 분노, 집중력저하 등 다양한 정서 문제를 호소하였다. 이런 환자에게 전화상담센터에서는 가미귀비탕과 명상 오디오파일(유튜브 링크 제공)을 제공하였다.

외상후스트레스장애에 대한 16개 중의학 문헌 검토 결과(단일군 전후 비교 연구 4편, 대조 임상 연구 12편), 외상후스트레스장애 환자에 한약뿐 아니라 일반침, 전침, 이침 및 침뜸 병용 치료, 심리치료와 침 병용 치료 등 다양한 중의학 치료가 활용되었으며 양약과 중의학 치료를 병용하는 경우가 많았다[89]. 사용된 한약 처방은 당울소(糖鬱疏), 백룡해울과립(白龍解鬱顆粒), 청심해울탕(清心解鬱湯)이었다.

87)Takayama, S., et al. The Role of Oriental Medicine in the Great East Japan Earthquake Disaster(東日本大震災における東洋医学による医療活動. Kampo medicine 2011;62(5):621-626.

88)Jang S, Kim D, Yi E, Choi G, Song M, Lee EK. Telemedicine and the Use of Korean Medicine for Patients With COVID-19 in South Korea: Observational Study. JMIR Public Health and Surveillance. 2021 Jan 19;7(1):e20236.

89)최유진, 권찬영, 장재순, 정하영, 김윤나, 정선용. 외상후스트레스장애 치료에 대한 중의학 임상연구 동향. 동의신경정신과학회지. 2016;27(3):197-206.

2) 임상적 고려사항

❶ 과긴장/불안[90)]

**가미소요산**	허증. 혈허. 갱년기 여성 고려.
**시호소간산**	실증. 간기울결. 가슴답답함.
**산조인탕**	허증. 심번 및 불면증 동반.
**시호가용골모려탕**	실증. 진정 효과. 우울증 동반.
**온담탕**	실증. 급성 불안.
**소담해울방(소요산가감)[91)]**	소요산에 청열약(황련, 대황주증), 거담안신약(반하, 진피, 석창포),<br>진정약(용골, 모려)을 추가함.
**해울환**	소요산과 감맥대조탕 합방.
**황련아교탕**	실열증, 가슴답답함 및 불면 동반.

❷ 공포[92)]

**계지가용골모려탕**	진정 효과. 시호가용골모려탕보다 허증. 냉증, 복통, 대소변불리 동반.
**시호계지건강탕**	계지가용골모려탕보다 허증. 구건, 오한, 냉증 동반.
**귀비탕**	허증. 심비양허. 불면, 우울, 피로, 식욕저하 동반.
**천왕보심단**	허증. 심혈허.
**우황청심원**	급성 고혈압, 정신혼미 동반 / 단기간 사용.

90)김근우. 불안장애 한의표준임상진료지침. 한의표준임상진료지침개발사업단. 대한한방신경정신과학회; 2017. 1-57p.

91)소요산(시호, 당귀, 복령, 백출, 백작약, 박하, 자감초, 생강)에서 생강을 빼고, 7개 약제(황련, 반하, 진피, 용골, 모려, 대황주증, 석창포)를 추가함.

92)공포로 얼어붙은 상태, 저각성 상태는 과각성이 극심하여 나타나는 상태로 파악함. 즉 충격에 대한 내적 에너지의 부족, 즉 과긴장상태에 비해 보다 허증으로 파악되어 불안장애진료지침 처방 중 허증 관련 처방을 제시함.

❸ 우울/애도[93)]

시호소간탕	실증. 간기울결. 가슴답답함 동반.
가미소요산	간울비허. 혈허. 갱년기 여성.
반하후박탕	실증. 기울. 매핵기 증상 주소증.
귀비탕	허증. 심비양허. 불면, 불안, 피로, 식욕저하 동반.
시호가용골모려탕	실증. 진정 효과. 우울증 동반.
계지복령환	실증, 어혈. 갱년기 여성, 상열감 및 안면홍조 동반.
육군자탕	허증, 식욕저하, 소화불량 동반.

❹ 분노/과민[94)]

억간산	실증. 두통 동반.
황련해독탕	실열증. 변비 동반.
분심기음가감	실증. 기울화화.
시호가용골모려탕	실증. 진정 효과. 우울증 동반.

❺ 불면/악몽[95)]

산조인탕	허증. 두근거림, 심번(心煩) 동반.
귀비탕	허증. 심비양허. 불안, 우울, 피로, 식욕저하 동반
온담탕	실증. 급성 불안
가미소요산	허증. 혈허. 갱년기 여성.
천왕보심단	허증. 심혈허.

93)김종우. 우울증 한의표준임상진료지침. 한의학연구원. 대한한방신경정신과학회; 2016. 1-10p.
94)정선용. 화병 한의표준임상진료지침. 한의표준임상진료지침개발사업단. 대한한방신경정신과학회; 2016. 49p
95)김보경. 불면장애 한의표준임상진료지침. 한의표준임상진료지침개발사업단. 대한한방신경정신과학회; 2017. 1-50p.

❻ 식욕저하/소화불량[96)]

**내소화중탕**	허실협잡. 잦은 식체.
**반하사심탕**	허실협잡. 심하비, 속쓰림, 심하부 작열감 동반.
**육군자탕**	허증. 비허습담. 담음정체. 전신무력, 심하비, 포만감, 잦은 트림 동반.
**소요산**	허실협잡. 간울비허. 우울, 불안, 불면 동반.
**시호소간산**	실증. 간위불화. 우울, 불안, 불면 동반.
**평위산**	실증. 습곤비위. 상복부 진수음, 오심, 어지럼증 동반.

❼ 무기력/탈진[97)]

**보중익기탕**	비기허. 중기부족.
**귀비탕**	심비양허. 불안, 우울, 피로, 식욕저하 동반.
**쌍화탕**	기혈양허.
**팔물탕**	기혈양허.
**십전대보탕**	기혈양허.
**육미/팔미지황환**	신음허/신양허. 노인.
**가미소요산**	간울비허. 혈허. 갱년기 여성.

96)김진성. 기능성소화불량 한의표준임상진료지침. 한의표준임상진료지침개발사업단. 대한한방내학회; 2020. 3-6p.
97)남동현. 만성피로 한의표준임상진료지침. 한의표준임상진료지침개발사업단. 대한한의진단학, 사상체질의학회; 2020. 3-6p.

❽ 두통[98)]

**청상견통탕가감**	실열증. 일체 두통에 활용.
**반하백출천마탕**	비허습담증. 소화기능이 좋지 않음. 어지럼증 동반.
**천궁다조산가감**	풍한증. 감기관련 두통. 열증 주의.

❾ 어지럼증[99)100)]

**반하백출천마탕**	비허습담증. 소화기능이 좋지 않음.
**천마구등음가감**	간양상항증. 번열감, 혈압상승.
**영계출감탕**	수기능심증(水氣凌心證). 위내정수(복진상 심하부 꿀렁거림), 두근거림, 두통.
**자음건비탕**	기혈양허+담음증. 만성경과.

❿ 통증[101)]

**오적산**	냉증. 소화불량 동반.
**당귀수산**	어혈. 급성 통증.
**쌍화탕**	허증. 만성통증. 근육긴장, 쥐내림, 피로감 동반.

98)이상관. 편두통 한의표준임상진료지침. 한의표준임상진료지침개발사업단. 대한중풍순환신경학회; 2017. 1-28p.
99)이의주. 현훈(어지럼증) 한의표준임상진료지침. 한의표준임상진료지침개발사업단. 사상체질의학회; 2021. 3-6p.
100)이재휘, 신현수, 김동현, 조창환, 임승민, 안정조, 조현경, 김윤식, 설인찬, 유호룡. 滋陰健脾湯加味方을 투여한 어지럼증 환자 70 례에 대한 분석 및 고찰. 동의생리병리학회지. 2010 Feb;24(1):171-6.
101)NEO 핸드북 편집위원회. NEO 인턴 핸드북 2nd Edition. 파주: 군자출판사; 2017. 661-75p.

4. 감정자유기법

1) 개요

감정자유기법(Emotional Freedom Techniques; EFT)은 경락과 경혈을 물리적으로 자극하여 에너지를 조절하는 정신의학적 접근법으로 2019년 외상후스트레스장애에 대한 한의 신의료기술로 등재되었다. EFT는 단기 노출(증상 선택과 평가)과 신체적 중재(경혈 두드리기), 인지적 요소(확언 등)가 결합된 치료법이다. Church 등(2017년)은 외상후스트레스장애 군인에게 EFT를 적용한 연구를 검토한 결과, EFT는 외상후스트레스장애 및 동반된 증상을 단시간 내에 개선시켰으며 EFT가 스트레스 호르몬과 변연계 기능을 조절하고 다양한 건강지표들을 개선하는 것으로 나타났다[102). 또한 이 연구에서는 EFT에 대해 (1) 치료 효과가 뚜렷하고 적응증이 폭넓으며, (2) 치료 기간이 짧고, (3) 부작용이 적으며, (4) 시술이 간단하여 배우기 쉽고 (5) 신체 및 심리증상 모두에 활용 가능하며, (6) 대규모 그룹 치료로서 활용 가능성과 치료효과가 밝혀졌으며, (7) 온라인이나 전화로 적용 가능하다는 점을 EFT의 장점으로 제시하였다.

EFT는 특히 트라우마 후 반응과 관련된 심리치료로 활용성이 높고 관련 임상연구에서도 통증, 불안, 우울, 외상후스트레스장애 척도 등이 유의하게 개선된 것으로 나타났다. EFT는 재난 생존자들에게 자기조절법으로 교육하여 증상에 대처하여 자기효능감을 회복하는 데 사용될 수 있다. EFT는 치료 효과가 높고, 비교적 치료 기간이 짧으며, 부작용 위험이 낮고, 필요한 훈련 시간이 적으며, 신체증상과 정신증상을 동시에 조절할 수 있다.

EFT의 이런 장점 때문에 재난 현장에서 안정화기법으로 활용할 수 있다[103). EFT를 개인적 자원봉사 혹은 지역 및 국제 비정부기구와 협력하여 여러 재난 현장(2004년 허리케인 카트리나, 태국 쓰나미, 르완다 인종 학살 생존자, 2008년 파키스탄 지진, 2010년 아이티 지진, 이라크 파병 참전용사의 트라우마, 멕시코 내전)에서 활용했다는 보고들이 있다[104).

102)Church D, Feinstein D. The manual stimulation of acupuncture points in the treatment of post-traumatic stress disorder: A review of clinical emotional freedom techniques. Medical acupuncture. 2017 Aug 1;29(4):194-205.

103)Feinstein D. Energy psychology in disaster relief. Traumatology. 2008 Mar;14(1):127-39.

104)감정자유기법 제4판. 대규모재난에 대처하기. 서울:대성의학사;2020. pp345-367.

2) 실시 전 고려사항

❶ 환자가 새로운 치료 방법에 가지는 거부감이 치료에 방해가 될 수 있으므로 알기 쉬운 용어로 간단히 설명한다. (예: "마음을 안정시켜주는 특정 혈자리가 있다. 그 부분을 두드리면 마음의 긴장이 더 잘 풀릴 것이다.", "마음이 아픈 것은 기운이 막혀서 생기는데 혈자리를 두드려서 막힌 기운을 푸는 방법을 사용해보겠다.")

❷ 치료와 함께 단계적으로 교육을 진행한다. 치료 과정에서는 전 과정을 모두 사용하고, 자가치료는 연속 두드리기를 먼저 알려주어 일상생활에서 사용하도록 한다. 치료 회기가 반복되면서 다른 과정을 순차적으로 교육한다.

❸ 준비작업 중 재난트라우마에 적합한 다양한 수용확언 예시를 아래에 제시하였다. 한의사는 생존자에게 적절한 것을 골라서 제시하고 생존자가 동의하는 예시들을 찾아서 EFT를 실시한다.

3) 기본 프로토콜 ※ 부록 10. 자가치료학습지 참고

❶ 문제의 확인

목표로 삼을 문제 혹은 증상, 불편감을 지정한다.

❷ 치료전 불편감 확인

0에서 10사이의 점수에 그 문제로 인한 불편한 정도 혹은 고통 점수를 정한다.

❸ 준비작업(SET-UP, 기운 바로잡기)

두드리기를 시작하는 과정이며, 두 손가락(주로 검지와 중지)으로 반대편 손날타점(후계혈)을 계속 두드리면서 아래의 간단한 어구를 정해서 세 번 반복해서 말한다. 어구는 그 문제를 인정하고, 그럼에도 불구하고 자신을 받아들인다는 내용이다.

'비록 나는 ____________ 이 있을지라도, 나는 이런 나 자신을 진심으로 완전히 받아들입니다.'

- 현재 상황을 대상으로 할 때는, '지금 불안하지만 / 불편하지만 / 회피하고 싶지만'으로 어구를 설정하며, 신체 증상을 대상으로 할 때는 '심장이 뛰지만 / 손에 식은땀이 나지만 / 두통이 있지만' 식으로 표현한다.
- 기운을 바로잡는 방법이라고 설명을 한다(극성역전 해소).

표 17 Affirmation(수용확언) 예시

과거	현재, 나 자신
비록 그 일만 생각하면 너무나 불안하고 무섭고 고통스럽지만<br>비록 너무나 갑작스러워 당황스럽고 불안하고 어떻게 해야 할지 모르겠지만<br>비록 하필이면 왜 나에게 이런 일이 일어났는지 당황스럽고 억울하고 화가 나지만,<br>비록 그 일만 생각하면 너무나 슬프고 속상하지만	비록 화가 치밀고 짜증이 나지만<br>비록 너무 외롭고 슬프게 느껴지지만<br>비록 죽을 만큼 힘들고 고통스럽지만<br>비록 너무 쉽게 깜짝깜짝 놀라게 되지만<br>비록 가슴이 두근거리고 무섭게 느껴지지만<br>비록 내 삶이 송두리째 흔들리고 없어지는 것처럼 느껴지지만<br>비록 내 삶이 돌이킬 수 없이 망가진 것처럼 느껴지지만<br>비록 우울하고 무기력해서 아무것도 하기 싫지만<br>비록 주체할 수없이 너무 많은 생각들이 떠올라서 고통스럽지만<br>비록 나쁜 생각(자살충동, 자해충동)이 자꾸 올라와서 무섭고 힘들지만
**현재, 외부 자극**	**현재, 신체증상**
비록 세상이 원망스럽고 사람들이 밉지만<br>비록 세상이 너무 낯설어서 당황스럽고 무섭지만<br>비록 모든 것이 위험하게 느껴져서 고통스럽지만<br>비록 남들의 말과 시선이 너무나 신경 쓰여서 고통스럽지만<br>비록 일상생활을 하고 사람들을 만나는 것이 너무 힘들지만<br>비록 세상과 사람들을 피하고만 싶어지지만	비록 너무나 긴장되고 생각이 많아져서 잠을 잘 수가 없지만<br>비록 너무 쉽게 긴장하고 머리가 아파지지만<br>(통증 부위가 아파지지만)
**미래**	**자아상**
비록 앞으로 어떻게 살아가야 할지 막막하고 불안하고 아득하지만<br>비록 앞으로 어떤 일이 일어나게 될지 무섭고 불안하지만<br>비록 당장 어떤 고통스러운 일이 벌어질 것 같아서 불안하지만<br>비록 평생 이러고 살아야 하나 하는 생각이 더 불안하게 만들지만	비록 이런 내 모습이 너무나 당황스럽고 불쌍하고 한심하게 느껴지지만<br>비록 이렇게 지내는 것이 죄스럽게 느껴지지만<br>비록 후회와 자책감이 몰려와서 고통스럽지만<br>비록 내가 재수 없는 사람처럼 느껴지지만

나는 이런 나 자신을 마음속 깊이 진심으로 받아들입니다.

표 18 단계별 EFT 수용확언 적용 예시

**1) 급성기 (선택확언)**	급성기에서 가장 핵심은 "안전감을 회복"하는 것이다. 급성기에는 생존자가 현재 경험하는 증상에 대해 다음과 같은 수용확언을 선택확언의 형식으로 만들어 적용한다. 급성기에는 "수용"보다는 "지금 내가 안전함을 인식하는 방향"으로 선택확언을 설정하는 것이 더 바람직하다. 이 때, 치료자는 재난 경험을 먼저 묻지 않고, 생존자가 현재 인식하고 표현하는 증상에 따라 아래의 형식을 이용하여 선택확언을 만든다.<br><br>**나는 멍하고 정신이 없지만, 나는 지금 내가 안전하다고 깨닫는 것을 선택합니다.**<br><br>예시)<br>나는 멍하고 정신이 없지만 / 나는 불안하고 두렵지만 / 너무 갑작스러운 일이 벌어졌지만 / 너무 충격적이고 놀랐지만 / 가슴이 두근거리고 무섭게 느껴지지만 / 내 삶이 송두리째 흔들리고 없어진 것처럼 느껴지지만 / 너무 혼란스럽고 뭐가 뭔지 모르겠지만 / 나도 모르게 자꾸 그 순간이 떠오르지만
**2) 아급성기 (수용확언 +선택확언)**	아급성기에 발생하는 ①과각성 ②우울/애도반응/죄책감 ③불신/고립감을 중심으로 EFT를 적용할 수 있다. 1개월 이상 지난 상태이므로, EFT 수용확언 또는 선택확언을 결합해서 생존자가 편안함을 느끼기 쉬운 수용확언을 선택, 적용할 수 있다.<br><br>**나는 (비록) ______ 하지만, 나는 지금 내가 안전하다고 깨닫는 것을 선택합니다. 지금의 나는 안전합니다. 나는 이런 나 자신을 진심으로 완전히 받아들입니다.**<br><br>예시)<br>① 과각성 수용확언의 예<br>나도 모르게 그 때가 떠올라서 너무 무섭고 두렵지만 / 작은 자극에도 깜짝깜짝 놀라지만 / 긴장과 불안감 때문에 목도 긴장되고 몸에도 힘이 들어가지만 / 불안감에 심장이 자꾸 빨리 뛰지만 / 숨이 차고 답답하고 불안하지만 / 악몽을 꿀까봐 무섭고 두렵지만<br>② 우울/애도장애/죄책감 수용확언의 예<br>가슴이 먹먹하고 눈물 밖에 안나오지만 / 슬프고 무력하지만 / 사랑하는 그 (대상)에게 일어난 일을 도저히 받아들일 수가 없지만 / 그 때 내가 조금만 다르게 행동했었더라면 그 사람도 안전했을텐데 / 후회되고 죄책감이 느껴지지만/ 이제 다시는 그 때로 돌아갈 수 없다는 것이 믿겨지지가 않지만<br>▶ 단, 자기 비하, 자책감이 심한 경우에는 반드시 전문가의 도움이 필요<br>③ 불신/고립감 수용확언의 예<br>그 누구도 내 마음을 / 내 상황을 진심으로 이해할 수 없지만 /<br>나를 동정하는 것 같아 불쾌하고 화가 나지만 /<br>나는 너무나 외롭고 나 혼자 덩그러니 버려진 것만 같지만 /<br>또 다시 이런 일이 벌어질까봐 두렵지만 /<br>세상은 너무 위험한 곳인 것만 같아 불안하고 두렵지만
**3)만성기**	불안한 마음이 사라지질 않지만 / 왜 이 증상들이 좋아지지 않지? 초조하고 불안하지만 / 도대체 언제까지 이렇게 지내야하는 거지? 답답하고 화도 나지만 / 도대체 언제쯤 나도, 이 상황도 좋아지는 걸까? 막막하고 답답하지만 / 이제는 예전으로 돌아갈 수 없는 걸까? 너무 두렵고 절망적이지만 / 왜 하필 나한테 이런 일이 일어난 건지, 억울하고 화도 나지만<br><br>**나는 이런 나 자신을 마음속 깊이 진심으로 받아들입니다.**

❹ 연속두드리기(Sequence, 14경락 기운열기)

다음은 신체의 각 부위를 7번 정도 두드리면서 연상어구라고 하는 말을 반복한다. 연상어구는 해결하고자 하는 문제에 집중하기 위한 목적으로 사용하며, 그 상황을 떠올리게 하는 단어로 간단하게 정한다. 대개 생존자가 호소하고 있는 증상 자체를 사용한다. 부위 순서는 정수리, 눈썹시작 부위, 눈 옆, 눈 밑, 코 밑, 턱, 쇄골 시작부위, 겨드랑이 밑인데, 연상어구는 짧은 단어로 그 문제를 단순히 확인하기 위해 간단하게 정한다. 연상어구는 문제에 따라 다른데, 예를 들어 '이 어깨 통증', '그때 사고', '악몽' 등이 될 수 있다.
적극적으로 마음의 기운을 소통시키는 방법이라고 설명한다.

그림 8 연속두드리기 혈위

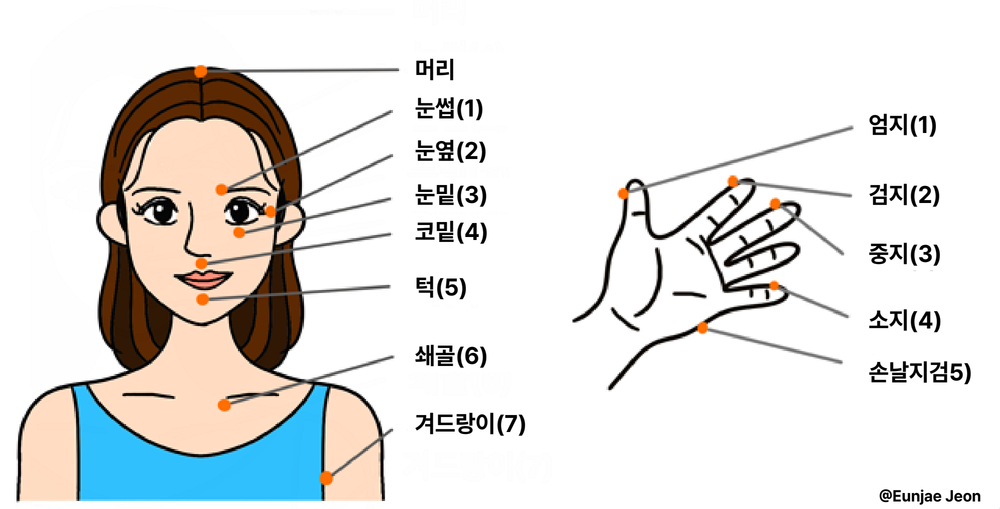

표 19 Reminder Phrase(연상어구) 예시

걱정되는, 암담한, 신경 쓰이는, 무서운, 겁나는, 두려운, 불안한, 긴장한, 안절부절못한, 조마조마한, 초조한, 불편한, 괴로운, 답답한, 숨 막히는, 슬픈, 가슴이 찢어지는, 그리운, 눈물겨운, 목이 메는, 서글픈, 서러운, 쓰라린, 애끓는, 울적한, 참담한, 처참한, 안타까운, 한스러운, 마음이 아픈, 비참한, 서운한, 야속한, 낙담한, 외로운, 공허한, 허탈한, 막막한, 우울한, 무기력한, 침울한, 피곤한, 따분한, 맥 빠진, 지긋지긋한, 귀찮은, 지겨운, 지친, 절망스러운, 실망스러운, 질려버린, 혼란스러운, 창피한, 놀란, 당혹스러운, 수치스러운, 화가 나는, 끓어오르는, 속상한, 분한, 울화가 치미는, 억울한, 화가 치밀어 오르는,
심장이 두근대는, 가슴에서 천불이 올라오는, 머리가 아프고 지끈대는, 어깨와 목이 (통증 부위가) 뻐근하고 쑤시는, 온몸이 뭉치고 뻐근한, 온몸이 천근만근, 명치가 꽉 막힌, 잠을 잘 수 없는, 몸에 힘이 하나도 없는, 어쩔 수 없이 몸이 긴장되고 힘이 들어가는,

❺ 불편감 재평가

1~4 단계를 한 뒤, 2단계에서 측정한 불편 점수가 감소하지 않았으면, 감소하거나 마음이 안정될 때까지 반복한다. 다시 반복할 때는 변화된 측면을 반영하여 수용확언과 연상어구를 다소 수정한다. 예를 들어, "비록 아직도 약간___, 남아 있는 ___ " 등으로 표현할 수 있다.

EFT는 인지적 노출과 언어처리, 경혈자극이 결합된 안정화 기법이다. EFT는 트라우마 회상 시 재경험으로 인한 불안이나 회피가 심한 경우에 활용할 수 있으며, 신체증상에도 활용가능하다. 일반적 트라우마 심리치료는 트라우마 기억을 구체적으로 다뤄 치료 중 트라우마 재경험이나 해리 등이 발생할 수 있지만, EFT는 반대로 전반적인 불편함을 먼저 다루어서 부작용을 최소화하며 안정을 도모한다.

4) 선택확언(Choice Method) - 추가 프로토콜

어떤 상태가 되기를 원하는지 치료의 방향을 명확하게 하기 위한 것이라고 설명한다.

표 20 선택확언(Choice Method) 예시

모든 마음의 고통이 사라지면 어떤 느낌이 들까?
모든 문제가 해결이 되었다면 어떤 느낌이 들까?

나는 ________해지는 것을 선택합니다.

**1) 급성기 선택확언의 예**
나는 이제 내가 안전하다는 것을 압니다. / 나는 안전합니다. / 나는 편안해지는 것을 선택합니다. / 내 몸과 마음이 건강하게 회복되는 것을 선택합니다. / 나는 회복되고 편안해지는 것을 선택합니다. / 내 몸과 마음이 건강하게 회복되는 것을 선택합니다. / 나는 건강하고 편안하고 행복해지는 것을 선택합니다. / 나는 가슴이 시원해지는 것을 선택합니다. / 나는 가슴이 편안해지는 것을 선택합니다. / 나는 깊게 단잠을 자게 되는 것을 선택합니다.
**2) 아급성기 & 만성기 선택확언의 예**
나는 내 삶의 주인공입니다. / 나는 뿌리 깊은 나무입니다. / 나는 행복하고 편안해질 자격이 있습니다. / 나는 나 자신을 비난하거나 미워하지 않습니다. / 나는 나 자신을 진심으로 이해하고 사랑합니다. / 나는 나 자신을 진심으로 이해하고 사랑하는 삶을 살아갑니다. / 나의 몸과 마음은 점점 회복하고 있습니다. / 내 안에는 내 감정을 조절할 수 있는 충분한 지혜와 힘이 있습니다. / 지금 일어난 일은 내 삶의 일부일 뿐입니다. / 나는 나의 길을 갈 수 있습니다. / 과거는 이미 지나갔고 미래는 아직 오지 않았습니다./ 나는 지금 여기에서 최선을 다하는 삶을 살아갑니다. / 내가 어쩔 수 없는 과거는 받아들이고, 내가 어쩔 수 있는 현재와 미래에 최선을 다합니다. / 조금 모자라도 조금 넘쳐도 내가 나의 길을 가고 있으니 그것으로 괜찮습니다. / 지금 일어난 일은 내 삶의 일부일 뿐입니다. 나는 나의 길을 갈 수 있습니다. / 과거는 이미 지나갔고 미래는 아직 오지 않았습니다. / 나는 지금 여기에서 최선을 다하는 삶을 살아갑니다. / 내가 어쩔 수 없는 과거는 받아들이고, 내가 어쩔 수 있는 현재와 미래에 최선을 다합니다.

5) 그룹치료 ※ 부록 9. 그룹치료 예시 참고

그룹치료는 공감하고 위로받는 느낌도 들고 바로윙 베네핏(Borrowing Benefit, 빌려 쓰는 이득) 효과를 얻을 수 있다.
주1회 4주간, 회당 소요시간 1시간으로 계획할 수 있는데 상황에 따라 유연하게 설정할 수 있다. 자가 학습 음성녹음파일과 자가 실습지를 제공하여 개별적으로도 매일 시행할 수 있도록 한다.

6) 주의할 점

EFT는 환자 스스로 경혈점을 두드리는 비침습적인 방법으로 대상자에게 직접적인 위해를 가하지 않으므로, 시술 수행에 따른 안전성에 문제가 없으며, 기존 연구에서도 부작용이 보고되지 않았다[105). 다만 노출요법의 요소가 포함되므로 초기 재난생존자에서는 트라우마의 기억을 직접적으로 떠올리기보다는 생존자가 호소하고 있는 증상들 위주로 EFT를 적용하되, 심각한 트라우마 증상을 호소하는 환자 또는 치료에 반응을 하지 않을 때는 다른 치료법을 고려하거나 전문가에게 의뢰해야한다.

그림 9 감정자유기법 활용을 돕는 동영상

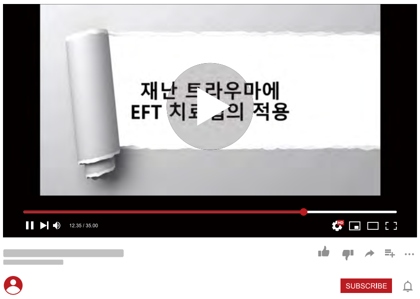

105)Flint, GA, Lammers W, Mitnick DG. Emotional Freedom Techniques: A safe treatment intervention for many trauma based issues. Journal of aggression, maltreatment & trauma. 2006;12(1-2):125-150.

5. 안정화기법

안정화기법은 재난 급성기 트라우마 치료에 가장 기본적이고 필수적인 치료 방법이다. 재난을 경험하여 편도체를 비롯한 변연계가 과잉활성화되고 전전두 피질의 기능이 저하되어 있는 상태에서 트라우마 생존자는 안정화기법을 통해 감정을 관찰하고 집중하는 법을 배워 전전두엽 기능을 활성화하여 트라우마와 관련된 신체 감각, 정서, 인지장애를 조절할 수 있도록 돕는다.

다양한 자연재난을 겪은 동남아시아에서 인도주의 단체인 '트라우마 에이드 독일팀'이 현지 치료사를 훈련시켜 재난으로 충격 받은 사람들을 대상으로 지원하였다. 이에 대한 연구 결과, 안정화기법이 외상후스트레스장애의 높은 완화율을 보이며 증상을 감소시키는데 매우 효과적이었으며, 유연하고 안전하고 독립적인 치료법이었다[106]. 여기에서 사용된 안정화 기법은 정신교육, 거리 두기 기술, 플래시백 다루기, 감정 조절 증진시키기, 리소스 활성화, 컨트롤 능력 향상, 대처기술 강화, 안전감 증진이었다. 또한 전쟁 트라우마를 겪은 난민들을 대상으로 디지털 오디오파일의 이미지 가이드를 활용한 연구에서 난민들에게 도움이 되고 잘 받아들여졌으며, 특히 "내면의 안전한 장소"기법이 큰 호평을 받았다[107].

이미지 안정화 기법[108]은 세 단계로 구성되어 있는데, 첫 번째 단계는 참가자들이 긴장을 풀고, 현재에 집중하며, 자기 자신과 연결될 수 있는 상태(self-engagement)에 들어갈 수 있도록 하는 의식 호흡으로 구성되었다. 두 번째 단계인 바디스캔은 원래 Jon Kabat-Zinn[109]이 개발하고 Louise Reddemann[110]이 설명한 것으로 참가자들에게 자기인식을 가르치고, 다른 신체 부위에 정신을 집중함으로써 자신의 몸을 경험할 수 있도록 하는 것을 목표로 한다. 세 번째 단계에서는 "내부 안전한 장소" 또는 "나무 운동" 중 하나를 사용한다[111]. "내부 안전한 장소"라는 기술은 개인들로 하여금 편안하고 안전하게 느껴지는 실제 또는 꾸며낸 장소에 있는 자신을 상상하도록 장려한다. "나무 운동" 동안, 개인들은 나무가 내면의 안정, 편안함, 그리고 에너지의 느낌을 불러일으키는 힘과 영향의 상징으로 상상한다. 이는 리소스 마인드풀니스와 같은 맥락에 해당된다.

106)Mattheß C, Farrell D, Mattheß M, Bumke P, Sodemann U, Mattheß H. The therapeutic value of trauma stabilisation in the treatment of post-traumatic stress disorder–a Southeast Asian study. Asian journal of psychiatry. 2019;41:45-49.

107)Zehetmair C, Nagy E, Leetz C, Cranz A, Kindermann D, Reddemann L, Nikendei C. Self-Practice of Stabilizing and Guided Imagery Techniques for Traumatized Refugees via Digital Audio Files: Qualitative Study. Journal of medical Internet research. 2020;22(9):e17906.

108)Zehetmair C, Kaufmann C, Tegeler I, Kindermann, D, Junne F, Zipfel S, Herpertz SC, Herzog W, Nikendei C. Psychotherapeutic Group Intervention for Traumatized Male Refugees Using Imaginative Stabilization Techniques—A Pilot Study in a German Reception Center. Frontiers in psychiatry. 2018;9:533.

109)Kabat-Zinn J, Hanh TN. Full catastrophe living: Using the wisdom of your body and mind to face stress, pain, and illness. NY; A Division of Random House, Inc.: 2009.

110)Reddemann, L. Imagination als heilsame Kraft. Klett-Cotta, Stuttgart; 2005.

111)Reddemann, L. Imagination als heilsame Kraft. Klett-Cotta, Stuttgart; 2005.

Pierre Janet의 근거기반 트라우마 접근 모델(1989)에서는 3단계 트라우마 치료 접근법을 제시했다[112). 1단계는 증상 완화, 안정화 능력을 키우는 작업(자원확대), 2단계는 반응(방어, 정향 등) 및 트라우마기억 처리 작업(리뷰 및 재평가), 3단계는 개인성 재통합, 관계와 친밀감 공고화, 재활 단계로 구성된다. 즉, 안정화 단계는 트라우마 치료의 가장 기본이 된다.

1) 안정화의 구성요소[113)114)]

치료적 동맹을 맺고 안전감을 증진시키며, 환자 경험을 정상화하고 유효화한다. 트라우마후스트레스와 치료에 대한 교육을 진행하고, 희망을 주고 강점을 강조한다. 공동작업을 통해 치료 목표를 생성하고 증상을 대처하며 증상을 조절하는 기술을 교육한다.

❶ 치료 동맹

치료자-환자 관계에서 관계성의 확립은 모든 치료에 있어서 기본 요소라 할 수 있다. 긍정적 치료관계는 성공적인 트라우마 치료의 가장 중요한 요소 중 하나이며, 트라우마 생존자에게 큰 위안과 지지를 제공할 수 있다.

❷ 안전감

트라우마치료와 관련된 안전은 먼저 주위 환경으로부터 최대한 안전하게 격리되어야 하며, 자기 파괴적인 사고나 행동의 개선, 그리고 자기 돌봄 기술의 개발과 습득을 통한 자신과 관련된 위험의 해결이다[115)].

❸ 정상화와 유효화

치료자는 재난 생존자들의 증상이 일종의 대처 방식이며 정상적인 반응임을 주지시켜야 한다. 환자가 경험하고 있는 증상은 부정적인 것이 아니라, 건강한 적응 방식이나 반응, 혹은 자기 보호의 의미가 있음을 교육한다.

112)Van der Hart O, Brown P, Van der Kolk BA. Pierre Janet's treatment of post-traumatic stress. Journal of traumatic stress. 1989 Oct 1;2(4):379-95.

113)재난정신건강위원회. 재난과 정신건강. 서울: 학지사; 2015. 268-271p.

114)Haskell L. First stage trauma treatment: A guide for mental health professionals working with women. Toronto, ON: Centre for Addiction and Mental Health; 2003. 45p.

115)Baranowsky A, Gentry JE. Trauma practice: Tools for stabilization and recovery. Boston, Massachusetts: Hogrefe Publishing; 2014.

❹ 심리교육[116)]

심리교육은 트라우마의 특성과 영향에 대해 올바른 정보를 제공하고, 이런 새로운 정보를 재난 생존자의 지식체계에 통합할 수 있도록 해야 한다. 자신의 겪는 증상이 재난을 경험한 후에 일어날 수 있는 자연스러운 반응임을 이해할 때, 수치심과 죄책감이 덜어지고 트라우마 반응을 완화시켜줄 수 있다.

❺ 희망주기와 강점

대규모 재난의 경우 그전에 자신이 가지고 있던 이 세상이 안전하다고 생각했던 세계관이 붕괴하며, 미래는 희망이 없다고 경험한다. 이때 긍정적인 미래나 결과가 가능하다는 기대감과 같은 희망 갖기와 긍정적 자원을 발굴하는 것은 중요한 치유 요소이다.

❻ 치료 목표 형성

성취할 수 있는 치료 목표를 설정하고 공유하며, 단순한 증상이나 불편한 감소보다는 전반적인 일상생활과 기능적 회복에 중점을 둔다.

❼ 증상 대처 및 조절 기술 교육

기술교육의 목표는 그들이 자주 경험하고 일상생활에 지장을 주는 강력한 감정이나 충동적 행동, 자기 비하적 생각이나 행동을 조절할 수 있는 방법을 교육하고 조절할 수 있는 방법을 습득해 스스로 이러한 증상에 대처하는 것이다.

❽ 한의학적 이해와 적용

치료동맹은 환자와 치료자와의 관계적 측면을 말한다. 한의학에서는 환자와 치료자의 관계에서 치료자 자신을 수양하고 마음을 살펴야함을 이도요법(**以道療法**), 허심합도(**虛心合道**)라고 하였다. 재난 현장에서 공감과 수용의 자세로 재난 피해자를 유기체적인 치유의 힘을 가진 존재 자체로써 바라보면 관계성이 구축되어 안전, 안심의 장을 형성할 수 있다. 한의학적으로 각 칠정에 따라 나타나는 증상으로 설명하고, 그것에 대한 의미를 설명해 줄 수 있다. 심리교육은 한의학 정신요법 중 오지상승요법의 사승공(**思勝恐**) 요법에 해당한다고 볼 수 있다. 트라우마에 대한 반응을 알고, 이러한 것들이 정상적인 반응임을 안다면, 공포의 감정을 다스릴 수 있다. 희망주기와 강점 강조는 한의정신요법 중 지언고론법과 유사하며, 부정거사의 방법으로 정기를 북돋아 사기를 몰아내는 한의학 치료원칙에 해당된다.

116)Fisher J. The work of stabilization in trauma treatment. Boston, Massachusetts: Trauma Center Lecture Series; 1999.

2) 안정화 기법

❶ 심리교육

심리교육이란 재난 경험후 심리적 과정 및 변화를 재난 생존자들에게 설명하여 이해를 돕는 작업이다. 심리교육은 모든 트라우마 관련 치료에서 근거기반의 핵심 요소이다. 교육은 기본적으로 재난 생존자와 기본적인 관계 형성이 된 후에 실시한다. 재난 생존자들이 불편해하는 부정적 증상을 건강한 반응이나 적응, 대처 방법, 자기보호적인 관점에서 바라볼 수 있도록 인식의 변화를 돕는다. 생존자가 자신의 반응이 자연스러우며 예측 가능하며 많은 사람들이 경험하는 것이라는 것을 알게 되면 불안감과 수치심, 자책감이 줄어든다.[117)118)].

2011년 동일본 대지진 피해 지역에서 재난 직후 짧은 심리교육으로도 아이들의 심리적 충격을 효과적으로 줄일 수 있었다[119)]. 또한 리비아에서 41명의 재난 생존자 대상 심리교육은 생존자들의 외상후스트레스 장애 증상을 줄일 수 있었다[120)].

예상치 못한 충격을 받은 재난 생존자는 매우 긴장하여 이해력이나 판단력이 평소보다 많이 저하된다. 그러므로 조심스럽게 접근하고 신뢰관계를 잘 만들어나가는 것이 중요하다. 생존자가 잘 이해할 수 있도록 쉬운 언어나 그림이나 소도구를 활용하는 것이 좋다.

- **목표**
  트라우마의 개념을 설명하고 내담자의 반응을 정상화하고 자기 반응을 이해할 수 있도록 돕는다.

- **고려점**
  - 대상자가 대화가 가능하다면 진찰결과 및 진료계획을 설명하고 심리교육을 시행한다.
  - 이완을 위해 먼저 복식호흡을 교육할 수 있다.
  - 가능한 경우 그룹으로 진행할 수 있다.
  - 대면이 어렵다면 온라인으로도 가능하다.
  - 복습 및 반복학습을 위해 심리교육 내용을 담은 교육자료를 제공하거나 인터넷 홈페이지(예: 국립트라우마센터의 재난정신건강정보, 정보알림의 공지사항)를 활용한다.
  - 과각성을 분산시킬 수 있는 방법을 함께 활용하는 것이 효과적인 교육에 도움이 된다. 교육 전에 한방차, 핫팩 혹은 아이스팩 등을 제공한다.

117)Hisli SN, Yilmaz B, Batigun A. Psychoeducation for children and adults after the Marmara earthquake: an evaluation study. Traumatology. 2011;17(1):41-49.

118)Fisher J. The work of stabilization in trauma treatment. Boston, Massachusetts: Trauma Center Lecture Series; 1999.

119)Fukuchi N. Psychoeducation for children in a psychiatric ward in the immediate aftermath of the 2011 earthquake and tsunami in Japan. Intervention. 2020;18(1):85.

120)Mughairbi FA, Abdulaziz AA, Hamid A. Effects of psychoeducation and stress coping techniques on posttraumatic stress disorder symptoms. Psychological reports. 2020;123(3):710-724.

표 21 심리교육(예시)

**트라우마란?**	트라우마란, 수많은 스트레스 경험 중에서 가장 충격적인 경험입니다. 내 몸과 마음이 감당할 수 없는 범위를 넘어서는 사건과 충격입니다.
**트라우마반응과 오해**	그러므로 너무나 무섭고 자꾸 충격이 떠올라 제 정신이 아닌 것 같고, 사람도 만나기 싫고 삶이 완전히 망가졌다고 느끼는 것은 너무 자연스럽고 당연한 반응입니다. 금방 이겨내고 힘을 내지 못하는 것은 다른 사람들도 마찬가지입니다. 다른 사람들은 다 잘 이겨내고 있는데 하필 내게만 나타나는 특별하고 독특한 반응이 아니랍니다.
**트라우마반응의 확인**	재난 경험후 ______와 같은 다양한 정서적, 신체적, 인지적, 행동적, 영적 반응과 증상이 나타난답니다. 이런 스트레스 반응은 매우 일반적인 적응, 보호 과정이랍니다
**트라우마반응의 이해**	첫째, 몸과 마음이 너무 과도하게 준비하는 것입니다. 내 자신을 지키기 위해 나타나는 반응입니다. 앞으로 조심하고 잘 대처하기 위해 충격적 기억들이 자꾸 생각나는 것입니다. 또한 지나치게 긴장하다보니 불안하고 예민해지고 화를 내기도 합니다.<br>둘째, 망연자실해서 기억력이 떨어지거나 다스리기 어려운 압도적인 감정들을 차단하려고 감정을 느끼지 못하거나 충격적 기억과 떠올리게 하는 것을 피하게 됩니다. 심한 경우 해리 증상이 나타날 수도 있는데 멍하거나 넋이 나간 듯 주변을 인식하기 어렵고 현실 세계가 실제같이 느껴지지 않고 중요한 기억이 나지 않거나 몸과 마음이 분리된 것처럼 느껴집니다.
**정리**	이런 증상들은 충격 직후 누구나 경험할 수 있는 반응입니다. 견디기 어려운 충격으로부터 몸과 마음을 보호하기 위해 최선을 다하는 대처반응인 것입니다. 시간이 지나면서 점점 나아질 수 있으나, 증상이 지나치거나 지속된다면 적절한 도움을 받으시는 것이 중요합니다. ___님께서 이 사실을 잘 이해하고 치료하고 관리해가면 좋겠습니다.

- **한의 심리교육**
  - 한의학의 칠정과 관련 신체 증상의 연관성에 대해 교육한다면 재난 생존자들은 본인들의 증상을 더욱 잘 이해할 수 있다. 칠정(七情)은 희(喜)·노(怒)·우(憂)·사(思)·비(悲)·공(恐)·경(驚) 7종의 감정 변화로서 정신활동의 구체적인 표현이며, 외부 스트레스에 대한 반응과 변화이다[121]. 스트레스 상황에서 칠정(七情)이 지나치면 정상 생리변화에 영향을 주고 질병을 발생시킬 수 있다[122].
  - 스트레스에 대한 반응은 주로 노(怒)·우(憂)·사(思)·비(悲)·공(恐)·경(驚)과 관련된 증상들이며, 칠정과 신체적 증상과의 관련성에 대해서는 다음과 같이 심리교육을 할 수 있다[123][124].

121)전국한의과대학 신경정신과 교과서편찬위원회 편. 한의신경정신과학. 서울:집문당;2014. 33-7p.
122)이경우 역. 황제내경소문上. 서울:여강출판사;2007. 589-92p.
123)김하나, 김경옥. 칠정(七情)에 의해 유발되는 신체적 증상에 대한 통계적 고찰. 동의신경정신과학회지. 2015;26(1):11-22.
124)이고은, 유영수, 강형원. 동의보감(東醫寶鑑)에 나타난 칠정(七情)의 대한 연구-병기(病機)를 중심으로. 동의신경정신과학회지. 2014;25(1):85-108.

표 22 칠정과 신체증상

노 (怒)	"怒則氣上, 怒則氣逆" "怒傷肝"	화 났을 때를 한번 떠올리시면 몸에서 뭐가 느껴지세요? 숨이 가빠지고, 열이 오르고, 목뒤가 뻐근하고 몸에 긴장이 되지요? "노즉기상, 노즉기역"이라는 말은 화가 날 때 우리 몸의 기운은 위로 치밀어 오른다는 뜻입니다. 그래서 화가 과도한 경우에 열 오름, 숨가쁨, 긴장감, 눈 뻑뻑함, 두통 같은 증상이 나타날 수 있습니다. 또한, 분노가 너무 과도해지면 간을 상하게 할 뿐만 아니라 정에 영향을 주어 기억력도 저하시킬 수 있습니다.	일상에서 과민하고 흥분된 상태. 분노(怒氣)가 과도한 경우 신체 증상 발현에 직접 관여한다는 것을 여러 가지 예시를 들어 설명함으로써 증상에 대한 이해를 도움.
우사 (憂思)	"愁則氣沈, 思則氣結" "憂思傷心" "思傷脾"	근심에 찬 사람들을 보면 어떠세요? 고개가 숙여지고, 가슴이 굽어있고 한숨을 푹 내쉬지요? "우수자 기폐색이불행"이라고 하여 근심이 있으면 기운이 막혀 운행하지 못한다 했습니다. 그래서 한숨을 내쉬게 되고, 대소변도 잘 안나오고, 근심과 고민이 지나쳐 비(脾)를 상하게 하면 식욕이 떨어지거나, 소화가 잘 안되거나 배가 부르는 등 소화기계 증상이 나타나고, 팔다리가 나른해 움직이기 힘들기도 합니다. 우울증의 증상들과도 유사합니다.	지나치게 생각을 많이 하면 심(心)을 상하게 하여 가슴 두근거림, 불면 증상들도 나타날 수 있음을 추가 설명할 수 있음.
비 (悲)	"悲則氣消." "悲傷肺"	슬퍼서 울고 나면 어떠세요? 뭔가 좀 시원한 느낌이 드시기도 하시죠? 눈물을 흘림으로써 기를 소산시키면 카타르시스가 되기도 합니다만, 너무 지나치게 되면 기운을 소모하게 됩니다. 한의학에서 슬픔이 지나치면 기운을 소모한다 하였고, 과도한 슬픔은 폐를 상하게 한다고 하였습니다.	일상의 상심, 번뇌로 생기는 슬픔으로 의기소침하며 가라앉는 상태를 설명.
공 (恐) 경 (驚)	"恐則氣下, 驚則氣亂." "恐傷腎"	간 떨어질뻔 했네. 놀라서 뱃속이 출렁했네. 말문이 막힌다. 이런 말 들어보셨죠.? 너무 놀라면 아무 생각도 안나고, 정신이 없잖아요? '恐則氣下,驚則氣亂'이 이를 뜻합니다. 놀람과 두려움은 기를 어지럽혀, 놀라고 잠 못자고, 불안하며 눈앞이 아찔하고 몸이 떨리기도 하는 등 다방면으로 큰 영향을 미친다고 알려져 있습니다. 그래서 놀란 기운을 안정시키는 게 트라우마 치료에서 중요한 것입니다.	일상에서 긴장(緊張)과 혼란(混亂) 상태로 관찰될 수 있음. 기란(氣亂)하여 심신(心身)이 안정되지 못하기 때문에 신체에 다방면으로 영향을 줄 수 있음을 인식하게 됨으로써 트라우마 반응이 정상적인 것임을 이해할 수 있게 되고, 안정화 기법을 교육할 수 있음.

❷ 호흡이완법

호흡이완법은 기본적인 정신건강 관리와 개선뿐 아니라, 재난 생존자들의 정신증상을 개선하기 위한 목적으로도 활용될 수 있다. 과거 재난의 충격에 빠져 있거나 미래에 닥치지 않은 재난을 걱정하는 것이 재난 생존자의 특징이다. 호흡을 활용한 명상은 과거의 후회나 미래의 걱정에서 벗어나 현재에 머무르고 행동하는데 도움을 줄 수 있다. 이와 관련하여 한의사가 사용할 수 있는 '한의사 마음 건강법 지도 매뉴얼'은 출판된 논문을 통해 확인하여 활용될 수 있다[125).]

125)Kwon CY, Kwak HY, Kim JW. Using Mind-Body Modalities via Telemedicine during the COVID-19 Crisis: Cases in the Republic of Korea. Int J Environ Res Public Health. 2020 Jun 22;17(12):4477.

한의사 마음 건강법 지도 매뉴얼

이 매뉴얼은 2020년 코로나19 한의진료 전화상담센터에서 사용되었다. 코로나19 관련 스트레스로 인해 환자에게 발생하는 증상을 지속적인 증상(과긴장, 공포, 불안, 무기력/우울) 또는 일시적 증상(불면, 소화불량, 통증, 분노/짜증)으로 분류하고, 각 증상별 심리교육과 함께 증상을 조절할 수 있는 심신요법 방법을 매뉴얼과 영상을 제공하였다.

기본적 심신요법으로는 '호흡훈련, 마음챙김 호흡, 걷기명상'이 포함되고, 이는 아침과 저녁에 1번씩 일상에서 정기적으로 시행하는 것이 권고된다. 개별 방법으로 1) 과긴장 상태에 점진적 근육이완법과 자율훈련법을, 2) 공포 상태에 수식관(산란한 마음을 집중시키기 위해 들숨과 날숨의 숫자를 헤아리는 수행법)을, 3) 불안 상태에 정좌명상을, 4) 무기력/우울 상태에 자애명상과 먹기명상을, 5) 불면증에 바디스캔을, 6) 소화불량에 먹기명상을, 7) 통증에 기와 함께하는 15분 명상을, 8) 분노/짜증에 정좌명상을 권고한다.

이 개별 방법은 증상이 발생하거나 악화될 때마다 시행하는 것이 권고된다.
대한한의사협회 유튜브 채널(AKOM_TV) 또는 강동경희대병원 한방신경정신과 유튜브 채널에서 제공하는 영상을 통해 시행 및 교육이 가능하다.

그림 10 호흡명상 유튜브 링크

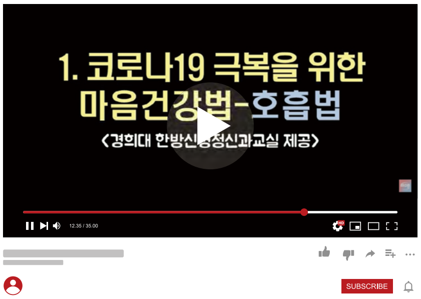

뇌의 해마는 트라우마 기억을 담당하고 편도체는 위협지각과 연관된다. 뇌영상검사상 규칙적인 명상을 한 사람은 해마의 크기가 컸으며[126], 우측 편도체의 크기가 작았다[127]. 이는 명상 수련자가 트라우마 기억을 잘 처리하여 트라우마에서 보다 빨리 회복할 수 있다는 것을 뜻한다. 명상은 마음챙김을 키울 수 있도록 도와준다. 마음챙김은 현재 일어나고 있는 무언가를 수용하고 느끼는 것이다. 마음챙김은 재난으로 인한 공포반응을 알아차리고 어떻게 대처해야할지 생각할 수 있도록 도와준다.

126)Luders E, Toga AW, Lepore N, Gaser C. The underlying anatomical correlates of long-term meditation: larger hippocampal and frontal volumes of gray matter. Neuroimage. 2009 Apr 15;45(3):672-8.

127)Gotink RA, Vernooij MW, Ikram MA, Niessen WJ, Krestin GP, Hofman A, Tiemeier H, Hunink MM. Meditation and yoga practice are associated with smaller right amygdala volume: the Rotterdam study. Brain imaging and behavior. 2018 Dec 1;12(6):1631-9.

명상은 여러 재난 상황에서 활용되었다. 2018년 태국 유소년축구팀이 폭우로 물이 불어난 동굴에 갇혔다가 17일 만에 전원 구조된 사건 당시, 엑까뽄 축구팀 코치는 전직 승려로 소년들에게 명상으로 마음의 안정을 찾고, 체내에 에너지를 비축해두는 방법을 가르쳤다고 한다[128]. 재난대응요원은 재난에 대한 노출로 불안, 우울, 외상후스트레스장애를 호소하는 경우가 많다. 사전에 마음챙김 기반 명상(Mindfulness Based Stress Reduction)을 교육한 경우 재난대응요원(군인)의 스트레스반응이 감소하였다[129]. 2011년 동일본 대지진 생존자 171명에 대한 초월명상 시행은 정상인 326명과 비교했을 때 스트레스관련 심리적, 신체적 증상을 뚜렷하게 감소시켰다[130]. 후쿠시마 제1 원자력발전소 사고로 인한 방사선 불안 및 건강 불안과 심리적 스트레스같은 재난 후유증에 마음챙김이 어떻게 영향을 주는지 알아보기 위해 사고 발생 7년 후 416명의 설문지 결과를 분석한 결과, 마음챙김은 건강불안과 심리적 스트레스 완화에 기여하였다[131].

기존 연구들에 따르면, 호흡을 느리게 조절하는 것만으로도 자율신경계와 중추신경계에 대한 진정 효과가 있으며, 뇌파 측정상 알파파가 증가하고 세타파가 감소하였고, fMRI 검사상 감정조절과 연관되는 일부 뇌 영역들을 활성화하여 편안함, 이완감, 행복감, 활력감을 증진하고, 각성, 불안, 우울, 분노, 혼란 등을 감소시킨다[132]. 또한 호흡에 집중하는 호흡명상은 기능적 인지적 예비능(functional cognitive reserves)과 기타 생리학적 시스템을 복원하는 보상 메커니즘을 활성화하여 급·만성 스트레스 기간 동안 수행능력을 유지하는 데 도움이 될 수 있다[133].

---

128)조선일보. 태국 암흑의 동굴서 '기적 생존기' 만든 4가지 요인. [Cited: March 29, 2021] Available:https://www.chosun.com/site/data/html_dir/2018/07/13/2018071301090.html

129)Atti SK, Alfalasi RB, Mahon SE, Sarin RR, Voskanyan A, Molloy MS, Ciottone GR. Mindfulness in Disaster Response. Prehospital and disaster medicine. 2017 Apr;32(S1):S181.

130)Yoshimura M, Kurokawa E, Noda T, Tanaka Y, Hineno K, Kawai Y, Dillbeck MC. Disaster relief for the Japanese earthquake–tsunami of 2011: Stress reduction through the transcendental meditation® technique. Psychological reports. 2015 Aug;117(1):206-16.

131)Kashiwazaki Y, Takebayashi Y, Murakami M. Relationships between radiation risk perception and health anxiety, and contribution of mindfulness to alleviating psychological distress after the Fukushima accident: Cross-sectional study using a path model. PloS one. 2020 Jul 6;15(7):e0235517.

132)Zaccaro A, Piarulli A, Laurino M, Garbella E, Menicucci D, Neri B, Gemignani A. How Breath-Control Can Change Your Life: A Systematic Review on Psycho-Physiological Correlates of Slow Breathing. Front Hum Neurosci. 2018 Sep 7;12:353.

133)Carter KS, Carter R 3rd. Breath-based meditation: A mechanism to restore the physiological and cognitive reserves for optimal human performance. World J Clin Cases. 2016 Apr 16;4(4):99-102.

재난현장에서 간단하게 활용할 수 있는 복식호흡법과 호흡명상을 소개한다. 호흡과 함께 생존자 스스로 자신의 맥박(손목 혹은 목)을 재는 것을 통해 자신이 살아있다는 것을 스스로 확인하여 안정감 형성에 도움이 될 수 있다.

복식호흡의 횡격막 호흡법 외에 다양한 호흡법을 활용할 수 있다. 호흡의 길이를 조절하거나, 코로만 하는 호흡, 입으로만 하는 호흡, 코와 입으로 하는 호흡, 호흡의 중간을 멈추는 호흡 등이 포함된다.
실제 재난 현장에서는 쉬운 호흡부터 시작한다. 시간이 지나면 호흡이 익숙해져 주의가 다른 곳으로 분산되는 경우 새로운 호흡으로 전환하도록 한다. 새로운 호흡을 연습하는 과정 속에서 상당한 안정감을 느낄 수 있다.

그러므로 쉬운 호흡(복식호흡)과 함께 2~3단계의 호흡 방식을 함께 활용할 수 있다. 또한 호흡의 난이도, 지속성 등에 따라 호흡 방법을 다양하게 활용할 수 있다.

(1) 복식호흡

스트레스 상황에서 심호흡을 하는 것은 교감신경계를 진정시키는 효과가 있다. 서로 마주 본 상황에서 함께 복식호흡을 시행할 수도 있다.

- 편안한 자세로 앉습니다.
- 공기가 코로 들어왔다가 나가는 것에 집중하면서 코를 통해 호흡을 시작합니다(5~6회 시행).
- 코를 통해 공기가 목과 폐를 지나 배까지 내려가는지 관찰해봅니다.
- 배를 이용해 좀 더 깊이 숨을 쉬어봅니다. 한 손을 아랫배 위에 얹어 놓고, 숨이 들이쉬고 나아가는 것을 느껴본 후, 익숙해지면 손을 떼고 하셔도 됩니다.
- 배 안에 풍선이 있다고 상상하시면서, 매번 숨을 들이쉴 때 공기가 흘러들어 배와 폐를 채우는 것을 상상해보십시오. 같은 방식으로 숨을 내쉴 때 공기가 폐에서 먼저 나와서 그다음에 배에서 나오게 됩니다.
- 호흡의 속도를 천천히 하기 위해 숨을 들이쉬고 내쉴 때마다 천천히 셋(들숨)/넷(날숨)을 세도록 합니다(5~6회 시행).
- 매일 집에서 5~10분간 이 과정을 연습해서 일상생활의 한 부분으로 만들도록 합니다

(2) 호흡명상 ※ 그림 10. 호흡명상 유튜브 링크 참고

조용한 장소를 찾아 편안하게 앉습니다.
- 조용한 장소를 찾을 수 없는 경우에는
- 귀에 이어폰이나 헤드폰을 끼고,
- 규칙적이고 부드러운 선율의 음악을 작게 켜놓아도 좋습니다.

자, 이제 호흡명상을 시작하겠습니다.
- 부드럽게 눈을 감습니다.
- 그리고 심호흡을 크게 3번 해보겠습니다.

숨을 부드럽게 들이마시고, 길게 내쉽니다.
- 숨을 부드럽게 들이마시고, 길게 내쉽니다.
- 숨을 부드럽게 들이마시고, 길게 내쉽니다.

자, 이제는 편안하게 호흡을 이어나가봅니다.
- 숨이 규칙적이고, 부드러우며, 중간에 끊어지지 않도록 합니다.
- 호흡하는 자신을 바라보며, 자신의 고유한 리듬도 확인해봅니다.

자, 이제는 숨이 들어오고 나가는 것을 바라보겠습니다.
- 어떠한 선입관이나 판단도 없이,
- 지금 이 순간 내 몸에서 일어나고 있는 호흡을
- 그저 바라봅니다.

코끝으로부터 공기가 내 몸으로 들어오는 느낌.
- 코끝, 입, 목구멍, 가슴.
- 이때 몸속으로 들어온 공기로 인해
- 몸이 부푸는 느낌도 확인해봅니다.

그리고 몸속으로 들어왔던 공기가 다시 몸 밖으로 나가는 느낌.
- 가슴, 목구멍, 입, 입술.
- 이때 몸 밖으로 공기가 나가며
- 부풀었던 몸이 다시 줄어드는 느낌도 확인해봅니다.

호흡을 계속 이어나가면서
- 이처럼 자신의 호흡을 계속해서 관찰해봅니다.
- 지금 이 순간에도, 끊임없이
- 숨이 들어오고 나가고 있습니다.

중간에 다른 생각이 떠오르는 것은 자연스러운 일입니다.
- 만약 중간에 잡념이 떠오른다면,
- 잡념이 떠올랐다는 것을 알아차리고
- 다시 호흡에 집중합니다.

어떠한 선입관이나 판단도 없이,
- 지금 이 순간 내 몸에서 일어나고 있는 호흡을
- 그저 바라봅니다.

이와 같이 호흡을 5분에서 10분 정도 계속해서 바라봅니다.

충분히 마음이 진정되었다고 생각이 되면,
- ‘이제 호흡명상을 그만하겠다’는 마음을 먼저 정한 뒤에
- 천천히 눈을 뜨며 명상을 마무리합니다.

❸ 그라운딩(Grounding)

재난 기억에 압도되어 공황 상태, 플래시백, 침습적인 부정적 사고, 해리 등이 일어날 때, 그라운딩은 재난 생존자가 현재의 외적 환경에 대한 지남력을 갖고, 치료자-생존자 간의 연결에 다시 초점을 맞추도록 하면서, 현재의 안전감을 느끼고 내적 각성 조절을 할 수 있게 도와준다.

그라운딩이라고 하는 것은 지면(ground)에 닿아있는 발뿐만 아니라 마음이 닿아질 수 있는 능력을 말한다. 그라운딩을 할 때에는 촉각, 시각, 청각, 후각 등 여러 가지 감각을 활용하는 것이 도움이 된다[134].

- 이제 저와 함께 배워볼 방법은 그라운딩이라고 하는 안정화기법입니다.
- 우리는 이 연습을 통해 지금, 여기에 머무를 수 있습니다.
  - 먼저 심호흡을 천천히 해봅니다.
  - 지금 당신이 머무는 이곳을 한번 찬찬히 돌아봅니다. 지금 어디에 있나요? 지금 몇 시죠?
    이곳을 묘사해 볼 수 있을까요?
    서서히 고개를 돌려 둘러보면서 뭔가 마음에 드는 것이 있으면 잠시 바라보며 그 느낌을 느껴보세요.
  - 자 이제는 양 발바닥이 바닥에 닿는 느낌과 등과 엉덩이를 받쳐주는 느낌, 팔걸이의 감촉을 느껴보세요.
  - 엉덩이가 닿아져 있는 느낌에 머물러보세요. 잠시 그곳에 머물고 있으면 어떤 느낌인가요?
  - 발바닥을 바닥에 붙이고 무게를 실어보세요. 발바닥이 바닥에 닿는 느낌이 어떻습니까?
  - 의자 팔걸이나 옆 부분을 만져보세요. 팔이 닿아져 있는 부분의 느낌은 어떤가요?
  - 이 중에서 가장 편안하거나 잘 느껴지는 부분이 어디인지 알아차려 보세요.
    그리고 그 부분의 느낌에 좀 더 머물러 봅니다.

❹ 이정변기요법 (정위반응 Orienting response)

불편한 정동이나 침습적 기억에 대처하기 위해 간단한 활동을 시행하는 경우이다. 재난 생존자와 상의해서 불편한 상태에서 주의를 다른 곳으로 돌릴 수 있는 활동을 찾아내고, 연습하게 도와준다. 예를 들어, 음악듣기, 산책하기, 재미있는 프로그램이나 동영상 시청하기, 그림 그리기, 손으로 만드는 작업 등이 활용될 수 있다.

이정변기란 정(精)을 움직여서 기분을 바꾼다는 것으로 기분을 바꾼다는 것은 정신을 바뀌게 해주는 것이다[135]. 이정변기요법은 매몰되어 있던 감정과 기억에서 벗어나고 고통스러운 기억과 체험을 직면하기 위해 신뢰할 수 있는 안전하고 지지적인 환경을 제공하기 위한 요법이다. 이정변기요법에는 음악, 미술, 시, 춤, 식물, 사람, 창문, 차(茶) 등을 이용한 정신전이법(精神轉移法)과 요가, 명상, 태극권 등을 이용한 정서도인법(情緖導引法)이 있다.

134) Fisher J. The work of stabilization in trauma treatment. Boston, Massachusetts: Trauma Center Lecture Series; 1999.
135) Yang YK. Hwangjaenaekyung Haeseok (Somoon). Seoul:Seongbosa;1980. 35, 108-12, 304, 664p.

최근 트라우마 치료에 있어서 정위반응(orienting response)을 이용한 기법이 응용되고 있는데[136), 정위반응은 인간을 포함한 동물들이 새로운 자극이 주어질 때 그쪽을 바라보거나 몸을 틀게 되는 것으로, 자극이 어느 쪽에서 올지에 따라 다가올 위험을 감지하고 예방하기 위한 것으로 일종의 생존 반응과 매우 연관이 깊다.

재난을 경험한 생존자들은 사소한 자극에도 필요이상으로 과각성하거나, 또는 저각성되어 꼼짝도 안하게 되는데, 정위반응을 이용하여 매몰되어있던 감정과 기억에서 벗어나도록 감각을 통한 반응을 키우는 훈련을 하게 한다. 정위반응은 고개를 천천히 돌려 주변을 살펴보게 한 후에 그 순간 가장 눈길이 가는 곳에 머물게 하여 신체 감각을 탐색하고 지금 여기 이 순간으로 다시 돌아오게 한다. 정위반응은 한방정신요법인 이정변기요법에 현대적 이론을 접목하여 활용한 심리치료법이다[137).

이정변기요법 (= 정위반응)

- **이정변기란 '정(精)을 움직여서(移) 기분(氣)을 바꾼다(變)'는 뜻입니다.**
  현재의 감정과 고통스런 기억에서 벗어나 안전하고 지지적인 환경을 체험하는 방법입니다.
  지금부터 저와 같이 해보겠습니다.

- **먼저 천천히 심호흡을 3번 정도 해봅니다.**
  지금 이 순간 고개를 천천히 그리고 충분히 상하좌우로 움직이면서 주변을 살펴봅니다.
  천천히 살펴보면서 눈길이 가는 것 3가지를 알아차려보세요.
  그것이 어떤 것인지 좀 더 자세히 말씀해주세요.

- **그 중에서도 뭔가 기분이 좋아지거나 편안하게 해주는 것이 있으신가요?**
  있다면 그것을 잠시 바라보면서 내 안에서 어떤 느낌인지 드는지 가만히 살펴봅니다.
  그 느낌을 충분히 느끼시고 천천히 돌아오시면 됩니다.

- **(기분이 좋아지거나 편안해지는 것이 없다고 할 경우)**
  괜찮습니다. 다시 천천히 고개를 돌려서 눈길이 가는 것을 다시 한번 찾아봅니다.
  그리고 그것이 어떤 것인지 말씀해보면서 어떤 느낌이 드는지 말씀해주세요.

136)Pat O. Kekuni M, Clare P. Trauma and the Body: A sensorimotor approach to psychotherapy. New York: WW Norton & Company; 2006. 27,69p.

137)박종민, 이고은, 서주희, 배달빛, 최성열, 강형원. 마음챙김 명상과 이정변기요법을 이용한 외상후스트레스장애 환자 치험례. 동의신경정신과학회지. 2014;25(1):73-84.

❺ 항아리요법 (봉쇄기법)[138)]

마음챙김 상태에서 심상을 통해서 재난 생존자가 힘들어하는 침습적인 기억, 이미지, 불편한 생리반응을 효과적으로 조절할 수 있도록 하는 방법이다. 봉인(containment)은 항아리나, 상자, 금고, 자루 등과 같이 담아두는 장치를 상상해서 현재 재난 생존자가 경험하는 불편한 요소를 담아 통제하는 능력을 가르치는 것이다.

불편한 증상을 형상화하여 통제하면 안도감을 준다. 불편한 증상이 봉인되면, 통제가능성이 증가하여 안도감을 느끼게 되기 때문이다. 이 요법은 충분한 안전의 장을 확보한 후에 실시한다. 불편한 증상이 봉인된 후 긍정적 감각과 정서(正氣)를 강화시켜 봉인된 증상(邪氣)을 처리할 수도 있다.

- 의식을 내면으로 가져가서 괴로웠거나 싫었던 체험을 떠올립니다.
- 그 체험이 떠오르면 그 상황, 내용을 이미지화해서 형태를 만들어 갈 수 있도록 합니다.
  구체적으로 내용을 듣지 말고, 고통스러운 장면이나 느낌의 경계를 정해 형태화합니다.
- 형태화한 이미지를 본인이 원하는 항아리에 넣어서 단단히 봉인합니다.
  그리고 열리지 않도록 열쇠로 잠그거나 끈으로 묶습니다.
- 단단하게 봉인한 이미지를 자기가 원하는 곳에 둡니다
  (예: 땅에 묻거나, 방 안 구석 어딘가에 두거나, 금고 안에 넣어두거나, 우주로 날려 보낼 수 있습니다).
- 원하면 언제든 다시 항아리를 저장해놓은 곳에 가서 그것을 볼 수 있습니다.
  굳이 찾지 않아도, 보지 않아도 괜찮습니다. 힘이 생기면, 언제든 다룰 수 있습니다.

138)한국M&L 심리치료연구원. 전문가를 위한 M&L 심리치료 프로스킬 트레이닝 코스 자료집.

❻ 리소스 마인드풀니스 (안전지대 심상화기법)[139)]

다양한 리소스 중 편안하거나 안심이 되는 공간(안전지대)을 선택하여 이를 심상화하도록 유도한다. 일부 불안정한 생존자에서는 안전한 곳을 찾을 수 없거나 부정적 기억이 리소스 장소(안전지대)와 연결이 될 경우 불안함이 오히려 악화될 수 있으므로 주의한다.

재난 현장에서는 짧게 시행하는 게 좋으며, 시행 도중 불안이 심해지거나 악화되면 바로 중지하고 정위반응이나 그라운딩을 통해 안정화시킨다. 안정이 불가능하다면 한방신경정신과 전문의 혹은 정신건강의학 전문의 및 심리치료 전문가에게 의뢰해야한다.

- **편안한 자세를 취하고 조용히 눈을 감아봅니다.**
- **그리고 천천히 세 번 정도 심호흡을 해봅니다.**
- **이번에는 그냥 조용히 자신의 마음의 눈으로 내면을 바라봅니다.**
- **이제 자신에게 있어서 가장 편안하고 안심이 되는 곳이 있으면 그대로 떠올려 봅니다.**
  - 장소를 발견하면 어떤 곳인지 천천히 얘기해 주실 수 있나요?
  (‘만약 장소가 떠오르지 않는다면, 그래도 괜찮습니다. 잔잔한 호수나 넓은 백사장이 있는 바닷가나, 파란 잔디밭에 누워 본 파란 하늘일 수도 있고, 숲속 오솔길 있습니다. 등’ 재난상황과 연관이 되지 않는 장소를 예로 든다.)
- **보이는 주변 풍경은 어떤가요? 뭔가 들려오나요?**
- **지금 몸으로 어떤 감각이 느껴지나요?**
- **얼굴에 닿는 공기가 느껴지세요? (오감을 동원하여 천천히 느끼게 한다)**
- **그리고 지금 거기에 있는 당신의 마음은 어떤가요?**
  - (지금 느끼고 있는 이 편안함, 안심의 느낌을 충분히 느끼도록 여유를 줍니다. 그리고 잠시 후)
- **이 느낌을 내 몸 어딘가에 저장하고 싶은 곳이 있는지 천천히 한번 찾아보시겠어요?**
- **(시간을 주고) 저장하고 싶은 곳을 찾으셨어요?**
- **그러면 그곳에 그 느낌을 그대로 저장해봅니다.**
  - 저장한 그곳의 느낌을 그대로 다시 한번 느껴봅니다.
  저장된 이 느낌은 당신이 필요하다면 언제든지 다시 느낄 수 있습니다.
- **이제 충분히 느끼셨다면 충분히 눈을 뜨고 돌아오셔도 됩니다.**

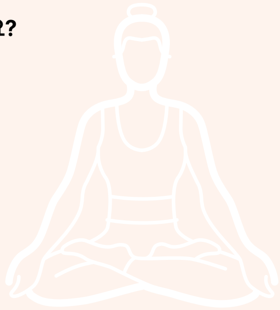

139)이희정, 강다현, 임명아, 김두리, & 서주희. 북한이탈주민의 심신증상에 대한 M&L 심리치료와 한의치료 치험 2 례 보고. 동의신경정신과학회지. 2016:27(3), 185-196.

3) 안정화기법 특징 및 주의점

기법	특징 및 적응증	주의점
심리교육	• 인식의 변화<br>• 자신의 반응이 자연스러우며 예측 가능하며 많은 사람들이 경험하는 것이라는 것을 알게 되면 불안감과 수치심, 자책감이 감소됨	• 시행전 기본 신뢰관계 형성이 필요.<br>• 그룹으로 시행하여 효율적 진행.<br>• 그림, 소도구 및 과각성을 분산시킬 수 있는 방법(한방차, 핫팩 혹은 얼음팩) 함께 활용
호흡이완법	• 자율신경계 진정을 통한 과긴장의 안정<br>• 손쉽게 적용가능	• 저각성 상태시 시행 주의, 단체로 활용가능
그라운딩	• 과각성, 저각성 모두 활용가능<br>• 생존자의 상태가 내성 영역안에 들어올 수 있도록 함<br>• 현재 감각을 자극하여 충격으로 압도된 사고, 감정, 기억에서 빠져나오게 함.	• 한의사는 상황 및 생존자의 상태에 따라 적절한 감각을 활용<br>• 현재 지지적 환경과 관계(예. 단단한 지면이나 나를 도와주는 사람들과 같이 있다 등의 안전요소)에 연결시킴
이정변기요법·정위반응	• 과각성 및 저각성 상태 모두 활용가능<br>• 주의를 전환시키는 활동	• 한의사는 상황 및 생존자의 상태에 따라 적절한 방법을 선택
항아리요법	• 침습적 기억, 플래시백이 뚜렷할 때<br>• 불편한 기억, 감정을 통제하는 능력을 획득	• 충분한 리소스와 안전의 장을 전제로 실시<br>• 불편한 기억을 처리하여 긍정 신체감각과 감정을 강화
리소스 마인드풀니스	• 노출치료를 위한 준비 과정으로 활용가능<br>• 치료를 마무리할 때 사용 가능.	• 시행전 호흡을 통해 충분히 이완상태가 되도록 함<br>• 안전한 곳을 찾을 수 없거나 부정적 기억이 리소스 장소와 연결이 될 경우 불안함이 오히려 악화될 수 있으므로 주의

- 심리교육이나 안정화기법을 단체로 교육할 때, 생존자들이 단체로 시행하는 방법이 사회적 연결성을 통해 개인 트라우마 증상의 완화뿐 아니라 재난 현장에 치유적 분위기를 조성하여 시너지 효과를 낼 수 있다. 애도와 치유, 연대를 통한 치유적 환경 조성이 트라우마 증상의 빠른 안정에 중요하다.

6. 자가 관리법

대규모 재난은 단시간에 회복하기 어려운 심각한 피해를 넓은 지역에 발생시킨다. 대규모 재난이 발생되어 전기, 가스, 수도 등 라이프 라인(life line)이 파괴되면 작은 병원은 이용하기 어렵고 한꺼번에 대량의 이재민이 몰려 매우 혼란스러우며 현지 의료인의 숫자도 부족하기 때문에 근처 병원의 응급의료구호소나 대피소에서 제대로 의료지원을 받기 어렵다. 재난의 충격과 피해는 단시간에 해결할 수 없어 이재민들은 주로 대피소에서 불편한 생활을 하게 된다.

큰 부상을 당하지 않더라도 이재민들은 재난트라우마를 겪어 이미 심리적, 신체적으로 취약한 상태인데 대피소에서 장기간 생활하면 많은 건강 문제가 발생할 수 있다. 이재민들은 정신적 고통뿐 아니라 감기 같은 감염성 질환, 소화기 증상, 통증, 불면, 심부정맥혈전증, 기존의 만성질환 악화를 경험하며 심지어 조기사망과 연관될 수 있다[140)141)142)143)144)145)146)]. 재난 발생 초기에는 대피소가 만원이기에 입소할 수 없어 자동차에서 피난 생활을 하다가 심부정맥혈전증이 발생하여 사망할 수도 있다. 2016년 구마모토 지진 시 피난 생활 관련 사망자는 가옥 붕괴 등으로 인한 직접적 사망보다 4배나 컸다[147)]. 특히 노인, 소아, 여성, 장애인, 저소득층 및 기존 정신장애 병력이 있는 취약한 계층은 더욱 큰 고통을 겪을 수 있다.

이를 정리하면, 1) 재난 발생 초기 대규모 생존자가 발생한 혼란스러운 상황에서는 제대로 의료지원을 받기 어렵고, 2) 장기간의 대피소 생활로 인해 다양한 증상이 발생하며, 3) 이에 대한 지속적이고 충분한 의료지원이 어렵고, 4) 기존의 의료지원이나 심리지원 등은 비용 및 시간, 인력 등이 많이 필요하므로 장기간 지속적으로 제공하기가 어렵다. 그러므로 이재민 스스로 건강을 관리할 수 있는 자가 관리법이 효과적으로 활용될 수 있다.

---

140)Kunii Y, Suzuki Y, Shiga T, et al. Severe Psychological Distress of Evacuees in Evacuation Zone Caused by the Fukushima Daiichi Nuclear Power Plant Accident: The Fukushima Health Management Survey. PLoS One. 2016;11(7):e0158821

141)Uemura M, Ohira T, Yasumura S, Otsuru A, Maeda M, Harigane M, Horikoshi N, Suzuki Y, Yabe H, Takahashi H, Nagai M, Nakano H, Zhang W, Hirosaki M, Abe M; Fukushima Health Management Survey Group. Association between psychological distress and dietary intake among evacuees after the Great East Japan Earthquake in a cross-sectional study: the Fukushima Health Management Survey. BMJ Open. 2016 Jul 5;6(7):e011534.

142)Takahashi T, Goto M, Yoshida H, Sumino H, Matsui H. Infectious Diseases after the 2011 Great East Japan Earthquake. J Exp Clin Med. 2012;4(1):20-23.

143)Inoue T, Nakao A, Kuboyama K, Hashimoto A, Masutani M, Ueda T, Kotani J. Gastrointestinal symptoms and food/nutrition concerns after the great East Japan earthquake in March 2011: survey of evacuees in a temporary shelter. Prehosp Disaster Med. 2014 Jun;29(3):303-6.

144)Yabuki S, Ouchi K, Kikuchi S, Konno S. Pain, quality of life and activity in aged evacuees living in temporary housing after the Great East Japan earthquake of 11 March 2011: a cross-sectional study in Minamisoma City, Fukushima prefecture. BMC Musculoskelet Disord. 2015;16:246.

145)Ueda S, Hanzawa K, Shibata M. One-year overview of deep vein thrombosis prevalence in the ishinomaki area since the great East Japan earthquake. Ann Vasc Dis. 2014;7(4):365-368.

146)Sawano T, Nishikawa Y, Ozaki A, et al. Premature death associated with long-term evacuation among a vulnerable population after the Fukushima nuclear disaster: A case report. Medicine (Baltimore). 2019;98(27):e16162.

147)재난 시 의료는 어떻게 될까? 현장에서 침구는 무엇을 할 수 있는지 : 미와 正敬 선생님 인터뷰. 건강 · 미용 정보 미디어 "케아쿠루" . 2018년5월 2일[Cited: Jan 7, 2021] Available: https://media.carecle.com/articles/n5lvb#i31602

자가 관리법은 다음과 같은 장점이 있다. 1) 외부적 도움에만 의존하지 않고 이재민 스스로 자신의 불편감을 조절하는데 적극적으로 참여한다면 자기효능감과 리질리언스(회복탄력성)를 증진할 수 있다. 2) 자가 관리법들은 기존 치료와 함께 활용되어 시너지 효과를 나타내거나 기존 치료의 부작용을 완화할 수도 있다. 3) 스스로 증상을 관리하면 불필요한 병원 방문이나 의료이용을 최소화하여 의료비를 절감할 수 있다.

먼저 재난현장에서 적용할 수 있는 1) 증상별 대처법을 소개하고, 2) 경혈지압, 3) 도인운동, 4) 걷기명상와 같은 구체적인 방법을 소개한다.

1) 증상별 대처법

❶ 과긴장/불안

**증상의 이해**	긴장은 재난의 충격 후 발생하는 스트레스를 이겨내기 위한 자연스러운 반응이다. 위협을 느끼면 과각성되고 근육은 긴장된다. 이러한 긴장은 미래에 다시 발생할지도 모를 충격을 대비하는 것이다. 지속적이고 과도한 긴장은 자율신경계 반응을 항진시켜 두근거림, 불면, 소화불량, 두통 등 다양한 증상과 회피행동을 발생시킨다. 긴장이 나를 보호하기 위한 반응이란 것과, 과도한 긴장은 오히려 대처능력을 떨어뜨린다는 것을 이해하는 것이 필요하다.
**호흡법**	호흡은 몸과 마음을 잇는 연결자가 된다. 내쉬는 숨을 길게 하여 교감신경계를 안정시키는 호흡법이 도움이 된다. 내쉬는 숨을 충분히 길고 천천히 한다. 불안이 안정되면 자연스러운 호흡으로 돌아온다. 적극적인 이완훈련을 위해 점진적 근육이완법과 자율훈련법을 추가 적용한다.
**생각 조절**	어떤 걱정이 있는지 파악한다. 나의 걱정이 타당한 것인지 주위 사람과 대화를 통해 확인하거나 직접 적어보는 것이 필요하다. 대화를 통해 나를 긴장시키는 생각이 명확해진다면 불안반응을 조절하기가 수월해진다.
**심상법**	편안하게 호흡하며 안전하고 편안한 장소를 떠올려본다.
**신체감각에 집중**	과도한 걱정과 불안을 조절하기 어렵다면 신체 감각에 집중하는 것이 좋다. 가볍게 걷거나, 마사지나 스트레칭을 하거나, 공을 주고받거나, 음악을 듣거나, 노래를 부르거나, 컬러링북을 색칠하거나, 족욕이나 반신욕을 하는 등의 활동이 도움이 될 수 있다. 가족이나 가까운 지인이 함께 활동한다면 자율신경계 안정에 더욱 도움이 된다.
**마음챙김하기**	억지로 생각을 바꾸려고 애쓰기보다는 생각을 내려놓고 긴장과 걱정들을 있는 그대로 관찰하고 수용하는 것이 더욱 도움이 된다. 이를 위해 호흡이완법이나 마음챙김 명상이 활용될 수 있다.

❷ 공포/정서마비

**증상의 이해**	공포는 극도의 과긴장과 불안으로 공황상태에 빠진 것이다. 재난의 충격 후 과도한 스트레스 반응을 처리할 수 없을 때 우리 몸과 마음은 얼어붙은 상태가 된다. 교감신경계 항진 증상뿐만 아니라 멍해지거나, 기억력저하, 정서적 마비, 해리 증상이 나타날 수 있다. 과긴장이 미래에 대한 불안이라면, 공포는 과거 충격의 재현이다. 하지만 이런 공포반응도 위협에서 자신을 보호하고 위협을 차단하기 위한 반응이라는 것을 이해한다.
**호흡법**	평소 호흡이완법을 통해 불안발작에 대처한다.
**그라운딩(착지 기법)**	주변에서 보이는 물건 세 가지를 찬찬히 살펴보거나, 편안한 의자에 기대어 닿은 부위를 느껴보거나, 서있다면 발이 바닥에 닿아있는 느낌을 확인해본다.
**노출 연습(경자평지요법)**	과도한 회피가 오히려 불안을 가중시킨다는 것을 이해한다. 회피하는 상황을 관찰하고 점진적으로 상황에 대한 노출을 연습해본다. 경자평지요법(체계적 탈감작요법)을 적용해볼 수 있다. 우선 이완되고 안정된 상태를 연습하여 안정감을 확보하고, 이를 바탕으로 트라우마 반응을 유발하는 자극에 대한 점진적인 노출을 시도한다. 다만, 노출 전 치료적 관계 형성이 꼭 필요하며, 성급한 노출을 주의해야하며 생존자의 상태가 악화된다면 이 방법은 사용하지 않고 전문가에게 의뢰한다.
**매스컴이용 자제**	언론매체의 재난 보도를 너무 자주 접하는 것은 오히려 공포를 악화시킬 수 있으므로 주의한다.
**전문가 의뢰**	과도한 차단으로 기억장애, 착란 등 해리증상이 발생하거나 자해 및 타해 위험이 있는 경우 전문가에게 의뢰한다.

❸ 우울/애도

**증상의 이해**	지나친 긴장이 풀어지지 않으면 심신의 에너지가 소모되어 무기력하고 탈진 상태가 된다. 재난에 잘 대처하지 못했다는 죄책감과 자책, 그리고 재난피해가 잘 복구되지 않고 트라우마 증상이 지속될 때 우울감이 발생한다. 재난의 충격으로 깨진 일상생활 리듬은 우울감을 가중시킨다.
**자살위험성 확인**	우선 자살위험성에 주의한다. 주기적인 평가와 관찰을 통해 자살 위험성을 확인하고 필요시 전문가에게 의뢰한다. 특히 과거 우울증 등 정신과적 병력이 있다면 더욱 유의한다.
**생활리듬 만들기**	재난의 충격으로 평상적인 일상생활을 할 수 없고, 대피소의 생활이 오래 지속될 수도 있으므로 새로운 규칙적인 생활 리듬을 만들어야 한다. 규칙적인 기상, 식사, 활동 및 수면시간을 정하고 실천하도록 한다.
**활동량 늘리기**	몸을 움직이고 활동량을 늘리는 것이 필요하다. 걷기 명상 및 스트레칭이나 도인운동법을 통해 제한된 공간에서 활동을 지속한다.
**수면 관리**	일상생활 리듬의 저하와 지속되는 불안으로 흔히 불면이 동반된다. 적극적인 수면 관리가 필요하다.

**생각의 변화**	우울감을 극복하기 위한 긍정적인 생각과 마음이 필요하다. 재난의 의미를 찾아보는 것, 과거가 아니라 미래를 계획하는 것, 그리고 변화된 자신의 모습을 수용하는 자애와 다른 사람들이 행복하기를 바라는 자비의 마음을 통해 밝은 에너지를 얻을 수 있다.
**사회적 관계**	같은 재난을 겪은 생존자와의 모임과 교류에 참여한다. 사회적 활동은 자율신경계(복측미주신경)를 활성화하여 사회적 관여체계를 자극하고 기분을 전환시킨다. 자조모임에서 다른 생존자들의 이야기를 듣고 돕는 활동 등이 우울감 극복에 도움이 될 수 있다.
**집착의 차단**	부정적인 생각의 악순환에 사로잡혀 에너지는 소모되고 우울함에서 벗어나지 못한다. 완벽한 행동이나 과도한 준비에 대한 집착을 내려놓도록 돕는다. 지나치게 많은 생각보다 아주 작은 행동 하나부터 시작해보도록 격려한다.
**애도 문제**	가족이나 지인의 죽음 등으로 인한 애도, 사별 문제가 있을 경우 이를 위한 구체적인 도움과 상담이 필요하다.

❹ 분노/과민

**증상의 이해**	분노, 짜증, 과민, 폭력적 행동까지 다양하게 나타날 수 있다. 상열감, 두통, 가슴두근거림, 답답함 같은 신체증상이 나타날 수 있으며, 주변 사람에게 발산하여 대인관계 문제가 발생할 수 있다. 재난 발생과 관련된 책임자가 있다면 이에 대한 분노는 자연스런 반응이다. 우선 분노가 상황에 대한 자연스런 반응임을 이해하도록 한다. 자신의 분노가 당황스러워 이를 부인하고 억압하면 또 다른 문제로 악화될 수 있다. 처리하기 어려운 감정에 대한 인식과 수용이 필요하다.
**분노 표출**	다만, 분노의 표출은 자해 및 타해, 사회적 관계 문제, 행동적 문제 등 다양한 문제를 야기하며 또다른 분노를 발생시킬 수 있다. 분노는 문제 해결에 도움이 되지 않는다는 것을 이해한다.
**호흡법**[148)]	들이쉬는 숨의 길이를 3으로 잡는다면 내쉬는 숨의 길이를 5 정도로 잡아서 호흡한다. 들이쉴 때 하나, 둘, 셋 하고 숫자를 세어본다. 내쉴 때는 다섯까지 센다. 갑자기 화가 났을 때 이렇게 들이쉬고 내쉬는 숨을 5회 정도 하면 약 1분이 지난다. 이렇게 세 번 반복하여 3분 정도 이런 패턴의 심호흡을 유지한다. 이렇게 시간이 지나면 교감신경의 흥분이 가라앉고 이후 일상적인 호흡이 유지된다. 평소의 호흡법이 10분 정도 유지되었다면 몸은 분노상태에서 벗어나 안정감을 느낄 수 있다. 이런 호흡법을 평소에 연습해두면 순간적으로 분노가 발생했을 때 효과적으로 대처할 수 있다.
**기타 생활관리**[149)]	음식(담백한 음식, 쓴맛 나는 채소), 차(페퍼민트(박하), 국화, 구기자, 귤피차), 산책, 글쓰기와 그림그리기, 주변 사람들과의 대화(감정표현과 대인관계 회복)를 고려한다.

148)대한한방신경정신과학회 화병연구센터. 화병100문100답. 서울:집문당;2013. p.257-8.
149)대한한방신경정신과학회 화병연구센터. 화병100문100답. 서울:집문당;2013. p.259-60, 261-3, 274-5.

❺ 불면

**증상의 이해**	초기에 재난의 충격 후 과긴장, 불안, 우울함이 발생하여 잠이 오지 않는 것은 자연스런 반응이다. 일상생활 리듬이 깨진 것이 원인이다. 활동량을 늘리며 새로운 리듬을 만들어내는 것이 중요하다. 1개월 이상 지속되는지 적절히 평가하고 관찰한다.
**적절한 각성상태**	햇빛을 충분히 쐬고 낮에 충분히 활동하여 적절한 각성상태를 유지한다. 재난 전 리듬대로 정해진 시간에 일어나 30분 이상 창가나 야외에서 햇빛을 쬐며 가볍게 걷는 게 도움이 된다. 제한된 일상생활 속에서 활동량을 늘리기 위해 규칙적인 활동을 계획하고 실천한다.
**낮잠 제한**	밤에 못 자서 피로감으로 낮에 누워서 휴식을 취하거나 낮잠으로 수면을 보충하는 경우가 많다. 밤낮이 바뀌어 생체리듬을 저하시키므로 낮잠은 제한하거나 최소화한다.
**이완법**	피곤함에도 긴장, 각성이 지속되어 잠들지 못한다. 자기 전 이완법을 시행한다. 호흡법, 목욕, 가벼운 산책, 음악 감상, 일기쓰기, 가족과의 대화 등 자신만의 이완법을 새롭게 만들고 실천하도록 한다.
**수면관리법**	- 잠을 자려고 애쓰고 노력하면 몸은 오히려 각성되어 잠들지 못한다. 수면제한법과 같은 역설적인 방법이 도움이 될 수 있다. 수면시간이 부족하다고 걱정하기보다 오늘 못 자면 내일 더 푹 잘 수 있다고 편안하게 마음을 먹는 것이 좋다.<br>- 30분 이상 잠이 오지 않을 때는 침대를 벗어나 다른 활동을 한다. 이때 할 수 있는 편안한 활동들을 준비한다. 그리고 다시 졸음이 찾아올 때 자도록 시도해본다.<br>- 잠을 한숨도 못 잤더라도 정해진 아침 기상시간을 지키는 것이 생체리듬을 만들어내는데 중요하다.<br>- 적절한 운동이 수면에 도움이 된다.
**피로감 관리**	한약, 침치료 등을 통해 낮동안 피로감을 적절히 관리할 수 있다.
**지압**	불면에 도움이 되는 경혈지압(이침혈위(신문) 및 일반경혈(신문, 내관, 삼음교, 용천 등))을 자가 관리법으로 활용할 수 있다.
**수면제복용 관리**	수면제 복용시 정확한 복용법을 지도하여 수면제 의존과 남용에 주의한다.<br>한약, 침, 수면교육, 인지행동치료, 이완법 등의 포괄적 수면관리를 통해 수면제 의존을 막고 적극적으로 수면을 관리한다.

❻ 식욕저하/소화불량

**증상의 이해**	우울감은 식욕을 저하시키고 불안과 긴장은 소화불량을 일으킨다. 식욕저하, 소화불량이 지속되면 면역력 저하와 우울감과 무기력으로 악화될 수 있다.
**천천히 먹기**	음식을 천천히 오래 씹어 그 맛을 느껴본다. 혀로 잇몸 구석구석을 양치질하듯이 마사지하여 침샘분비를 자극한다.
**심상법**	평소 좋아하는 맛있는 음식을 찾고, 그 기억과 맛을 상상하고 음미하도록 한다. 가족 및 친지, 친구와 함께 맛있게 먹었던 기억을 찾아보고, 최대한 구체적으로 떠올려본다.
**생체리듬 조절**	식욕과 위장활동은 생체리듬의 하나이다. 규칙적인 식사 및 활동량 증가를 통해 식욕을 촉진시킨다.
**식후관리**	식사 후 가벼운 산책을 통해 위장관 운동을 도와 소화를 촉진한다.

**지압**	적절한 지압(합곡, 내관 등)을 통하여 소화기능을 개선하도록 교육한다.
**이완**	과도한 긴장과 불안은 위장관 운동을 저해한다. 이완을 통해 편안한 마음을 갖는다.
**섭식장애**	과도한 섭식장애(거식증/폭식증)가 동반된다면 이의 평가와 전문가 의뢰를 고려한다.

❼ 무기력/탈진

**증상의 이해**	우울, 과긴장의 지속으로 발생할 수 있다. 재난 충격은 쉽게 해결되지 않고 장기간의 대피소생활이 지속되면서 무기력해질 수 있다. 일상생활 리듬의 깨짐과 연관된다. 규칙적인 기상, 식사, 활동, 수면을 갖도록 한다. 우울과 불면이 피로감으로 이어지고, 피로감은 또한 우울과 불면을 악화시킬 수 있다. 따라서 우울증 및 수면 관리가 통합적으로 이뤄져야한다.
**활동량 증가**	활동량을 증가시켜야한다. 가벼운 스트레칭과 운동, 걷기 및 걷기 명상, 가벼운 집안일 등을 통해 일상생활 속에서 활동량을 증가시킨다. 활동량 증가는 우울과 불면 개선에도 도움이 된다.
**지압**	적절한 지압(족삼리, 용천 등)을 통하여 피로감을 개선하도록 교육한다.
**사회적 활동**	피로감으로 활동량이 떨어지고 고립되면 우울증으로 악화될 수 있다. 자조 모임 등을 통해 사회적 활동을 시도하는 것이 피로와 우울감 개선에 도움이 될 수 있다.

❽ 두통/어지럼증

**증상의 이해**	재난 충격 후 과긴장과 우울, 활동량 저하, 분노, 과민 등으로 인한 목, 어깨의 근육경직과 관련되어 두통, 어지럼증이 발생할 수 있다. 눈앞이 캄캄해지는 어지럼증은 몸이 허약하고 저혈압 상태로 머리쪽으로 순환이 안되는 경우가 많으므로 충분한 영양공급과 적절한 운동이 필요하다.
**바른 자세**	자세가 중요하다. 한 가지 자세를 오래 취하면 특히 목, 어깨 근육이 긴장되기 쉽다. 컴퓨터나 스마트폰 과사용을 주의하고 바른 자세를 취하는 것이 필요하다.
**스트레스 관리**	불안, 긴장, 우울은 몸을 긴장시켜 두통, 어지럼증과 연관되거나 악화시킬 수 있다. 호흡법, 걷기, 간단한 기분전환 활동 등을 통해 심리적 스트레스를 적절히 관리해야한다.
**지압 및 스트레칭**	목 주변의 경혈점(풍지, 풍부, 천주혈 등)을 지압하여 뭉친 기운과 근육을 풀면 머리로의 혈액 순환이 개선되어 머리가 맑아진다. 특히 승모근과 흉쇄유돌근의 긴장이 밀접한 관련이 있다. 이 근육의 스트레칭과 침치료가 도움이 된다.
**찜질법**	목덜미, 뒤통수 쪽에 뜨거운 찜질팩을 베고 누우면 목, 어깨 긴장 이완에 도움이 된다.
**예기불안 대처**	어지럼증이 심하여 실신하거나 공황발작이 동반된다면 예기불안이 발생하고 어지럼증을 유발하는 요인에 대한 회피가 발생한다. 이런 활동제한은 불안 긴장과 우울을 악화시킬 수 있으므로 호흡법 등 이완법을 활용하여 예기불안과 긴장에 대처한다.
**진료의뢰**	두통, 어지럼증이 종일 지속되거나, 두개내압항진 증상, 복시, 상하지소력 등 기타 신경학적 증상이 나타난다면 뇌정밀검사를 위해 전문의 진료의뢰를 고려한다.

❾ 통증

**증상의 이해**	통증은 외상후스트레스장애에서 흔히 동반되는 신체증상이다. 근육의 과긴장은 통증을 유발한다. 호흡법 등을 통한 과긴장의 이완이 필요하다. 일상생활 리듬이 깨져 활동량이 저하된 경우 우울함이 유발되어 통증에 대한 민감도가 높아진다. 적극적인 통증관리를 위해 활동량의 개선이 필요하다.
**주의력 분산**	통증으로 그 부위에 신경이 쓰이면 통증은 더욱 악화된다. 관심을 다른 곳으로 돌리는 연습이 필요하다. 가벼운 집안일이나 산책, 사람들과의 대화, 음악 듣기 등 주의전환이 필요하다.
**마음챙김**	통증은 위협과 신체적 트라우마에서 나를 지키기 위한 감각이다. 통증을 위협적이고 부정적으로 바라보는 시선을 바꿔보도록 시도한다. 통증은 있는 그대로 느끼고 바라보고 시간에 따라 어떻게 변화하는지 관찰하려는 시도가 필요하다. 마음챙김 명상이 도움이 될 수 있다.
**지압**	해당 통증 부위와 연관되는 경혈을 지압한다.

2) 경혈지압

(1) 배경

경혈지압은 증상을 개선하고 건강을 유지하기 위해 체표의 특정 부위(경혈)에 손가락이나 도구를 통해 누르고 자극하는 치료 및 관리법이다. 경혈은 경락에 위치하며, 경락(12경맥과 15낙맥)은 내장에서 근육, 피부까지 인체 모든 부위를 연결하는 거대한 망(네트워크)이다. 경혈은 경락을 운행하는 기(생명 에너지)의 생리 반응점이자, 질병 발생과 연관되는 병리/진단 반응점이자, 또한 지압을 하는 치료 자극점이다. 경혈에 유효한 자극을 주면 생체반응이 나타나 질병을 예방하고 치료할 수 있다.

경혈지압은 간단해서 누구나 쉽게 할 수 있으며 장소와 시간에 제약 없이 혼자서나 가족이나 동료에게 해줄 수 있다는 장점이 있다. 경혈지압은 비약물, 비침습적인 방법이므로 통증 없이 안전하게 적용하여 환자 스스로 자신의 증상을 조절하고 건강을 증진시켜 삶의 질을 개선할 수 있다. 재난 발생 초기 생활기반 시설이 파괴되어 수돗물이 충분히 대피소에 공급되지 않아 이재민들은 목욕은 물론 손을 제대로 씻을 수도 없다. 침치료는 감염발생 위험을 줄이기 위해 적절한 치료부위 소독을 시행하므로 소독에 필요한 깨끗한 물, 소독액, 알코올 솜 등 추가 자원을 필요로 하지만, 경혈지압이나 마사지는 비침습적이기에 별도로 소독을 위한 자원이 필요없고 감염 위험이 없어 간편하고 안전하게 활용할 수 있다[150]. 또한 침치료에 대한 공포가 있는 생존자나 어린이들에게 부담 없이 적용할 수 있다.

150) Takayama S, Kamiya T, Watanabe M, Hirano A, Matsuda A, Monma Y, Numata T, Kusuyama H, Yaegashi N. Report on disaster medical operations with acupuncture/massage therapy after the great East Japan earthquake. Integr Med Insights. 2012;7:1-5.

재난 후 이재민들은 정신적 충격뿐 아니라 좁고 사생활도 확보할 수 없는 불편한 대피소 생활로 인해 어깨와 허리 및 관절의 통증, 전신 피로, 붓기와 저림, 식욕저하와 변비 등 다양한 증상을 호소한다. 경락 및 경혈은 전신에 분포하며 오장육부와 연결되므로 각 증상에 연관되는 경락 및 경혈을 선택하여 지압하면 다양한 증상에 대처할 수 있다.

경혈지압 및 이침혈위 지압(auricular acupressure)은 불안, 불면 같은 정신과 증상뿐 아니라 통증, 호흡기, 소화기, 피부질환, 여성질환, 수술후 부작용 등 다양한 증상과 질환에 적용되었으며 그 효과에 대한 많은 근거들이 있다. 2015년 시행된 체계적 문헌고찰에서 자가 지압은 알레르기 질환, 암환자의 오심 및 구토, 호흡기질환, 월경통, 건강인의 스트레스와 피로, 불면에 유의한 효과가 있었다[151). 불안[152)], 불면[153)154)155)156)], 통증[157)158)], 경항통(낙침)[159)], 분만통[160)], 수술후 통증[161)], 통증과 동반된 불안[162)], 월경통[163)], 월경전증후군[164)], 항암

---

151)Song HJ, Seo HJ, Lee H, Son H, Choi SM, Lee S. Effect of self-acupressure for symptom management: a systematic review. Complementary therapies in medicine. 2015 Feb 1;23(1):68-78.

152)Kwon CY, Lee B. Acupuncture or acupressure on yintang (EX-HN 3) for anxiety: a preliminary review. Medical acupuncture. 2018 Apr 1;30(2):73-9.

153)Waits A, Tang YR, Cheng HM, Tai CJ, Chien LY. Acupressure effect on sleep quality: a systematic review and meta-analysis. Sleep medicine reviews. 2018 Feb 1;37:24-34.

154)Zhao ZH, Zhou Y, Li WH, Tang ZH, Xia TW. Auricular acupressure in patients with hypertension and insomnia: a systematic review and meta-analysis. Evidence-Based Complementary and Alternative Medicine. 2020 Jun 17;2020.

155)Jang M, Park H. The Effects of Auricular Acupressure on Sleep Disorder in The Elderly: A Systematic Review and Meta-analysis. Journal of the Korea Academia-Industrial cooperation Society. 2020;21(6):116-26.

156)Gong Z, Yu W. Effectiveness and Characteristics of Acupressure for Elderly with Insomnia: A Systematical Review. Global Journal of Intellectual & Developmental Disabilities. 2017;2(2):58-66.

157)You E, Kim D, Harris R, D'Alonzo K. Effects of auricular acupressure on pain management: A systematic review. Pain Management Nursing. 2019 Feb 1;20(1):17-24.

158)Liu M, Tong Y, Chai L, Chen S, Xue Z, Chen Y, Li X. Effects of auricular point acupressure on pain relief: a systematic review. Pain Management Nursing. 2020 Sep 17.

159)Kwon CY, Lee B. Clinical effects of acupressure on neck pain syndrome (nakchim): a systematic review. Integrative medicine research. 2018 Sep 1;7(3):219-30.

160)Chen Y, Xiang XY, Chin KH, Gao J, Wu J, Lao L, Chen H. Acupressure for labor pain management: a systematic review and meta-analysis of randomized controlled trials. Acupuncture in Medicine. 2020 Aug 18:0964528420946044.

161)Zhong Q, Wang D, Bai YM, Du SZ, Song YL, Zhu J. Effectiveness of auricular acupressure for acute postoperative pain after surgery: a systematic review and meta-analysis. Chinese journal of integrative medicine. 2019 Mar 1;25(3):225-32.

162)Monson E, Arney D, Benham B, Bird R, Elias E, Linden K, McCord K, Miller C, Miller T, Ritter L, Waggy D. Beyond pills: Acupressure impact on self-rated pain and anxiety scores. The Journal of Alternative and Complementary Medicine. 2019 May 1;25(5):517-21.

163)Ghiasi A, Keramat A, Mollaahmadi L, Hashemzadeah M, Bagheri L. The effect of acupressure at the Sanyinjiao (SP6) point on relief of primary dysmenorrhea: a systematic review of clinical trials. The Iranian Journal of Obstetrics, Gynecology and Infertility. 2017;19(40):55-68.

164)Armour M, Ee CC, Hao J, Wilson TM, Yao SS, Smith CA. Acupuncture and acupressure for premenstrual syndrome. Cochrane Database of Systematic Reviews. 2018(8).

치료 유발 구역·구토[165], 비만[166] 근시[167], 수술 후 장폐색[168], 변비[169)170)171], 가려움증[172], 알레르기성 호흡기 증상[173], 인지 기능, 치매의 행동심리증상[174], 치매간병인의 스트레스[175], 노인의 불면·우울,[176] 노인의 삶의 질 개선[177]에 대한 많은 근거들을 확인할 수 있다.

외상후스트레스장애나 이재민들에게 직접 경혈지압을 사용한 연구는 없으나 동일본 대지진 장기 이재민에게 침과 마사지 병용치료를 시행해 통증을 완화시켰다는 보고가 있다[178)179]. 또한 외상후스트레스장애 및 다양한 심리 및 신체증상에 활용되는 감정자유기법(EFT)은 인지 및 노출요법과 함께 특정 경혈점을 두드리는 지압과 유사한 과정이 결합되어 있다[180].

165)Miao J, Liu X, Wu C, Kong H, Xie W, Liu K. Effects of acupressure on chemotherapy-induced nausea and vomiting-a systematic review with meta-analyses and trial sequential analysis of randomized controlled trials. International journal of nursing studies. 2017 May 1;70:27-37.

166)Huang CF, Guo SE, Chou FH. Auricular acupressure for overweight and obese individuals: a systematic review and meta-analysis. Medicine. 2019 Jun;98(26).

167)Gao H, Zhang L, Liu J. Auricular acupressure for myopia in children and adolescents: A systematic review. Complementary therapies in clinical practice. 2020 Feb 1;38:101067.

168)Liu Y, Tang WP, Gong S, Chan CW. A systematic review and meta-analysis of acupressure for postoperative gastrointestinal symptoms among abdominal surgery patients. The American Journal of Chinese Medicine. 2017 Aug 22;45(06):1127-45.

169)Zheng SH, Yan M, Field T, Xu X. Beneficial effects of auricular acupressure on preventing constipation in breast cancer patients undergoing chemotherapy: evidence from systematic review and meta-analysis. Frontiers of Nursing. 2018 Oct 25;5(3):227-34.

170)Sahan S. The Effect of Acupressure on Constipation: A Systematical Review. Int J Clin Med Info. 2020;3(1):28-34.

171)Wang PM, Hsu CW, Liu CT, Lai TY, Tzeng FL, Huang CF. Effect of acupressure on constipation in patients with advanced cancer. Supportive Care in Cancer. 2019 Sep 1;27(9):3473-8.

172)Aval SB, Ravanshad Y, Azarfar A, Mehrad-Majd H, Torabi S, Ravanshad S. A systematic review and meta-analysis of using acupuncture and acupressure for uremic pruritus. Iranian journal of kidney diseases. 2018 Mar 1;12(2):78.

173)Liang Y, Lenon GB, Yang AW. Acupressure for respiratory allergic diseases: a systematic review of randomised controlled trials. Acupuncture in Medicine. 2017 Dec;35(6):413-20.

174)Harris ML, Titler MG, Struble LM. Acupuncture and acupressure for dementia behavioral and psychological symptoms: a scoping review. Western journal of nursing research. 2020 Oct;42(10):867-80.

175)Almeida Sr JR, Almeida Sr MC, Cunha Sr ME, Silva Sr AG. The effectiveness of a self-administered acupressure intervention in caregivers of Alzheimer's disease patients: A systematic review: Psychosocial factors and environmental design/Living with dementia and quality of life. Alzheimer's & Dementia. 2020 Dec;16:e037536.

176)Hmwe NT, Browne G, Mollart L, Allanson V, Chan SW. An integrative review of acupressure interventions for older people: A focus on sleep quality, depression, anxiety, and agitation. International Journal of Geriatric Psychiatry. 2019 Mar;34(3):381-96.

177)Chen MC, Yang LY, Chen KM, Hsu HF. Systematic review and meta-analysis on using acupressure to promote the health of older adults. Journal of Applied Gerontology. 2020 Oct;39(10):1144-52.

178)Takayama S, Kamiya T, Watanabe M, Hirano A, Matsuda A, Monma Y, Numata T, Kusuyama H, Yaegashi N. Report on disaster medical operations with acupuncture/massage therapy after the great East Japan earthquake. Integr Med Insights. 2012;7:1-5.

179)Takayama S, Kamiya T, Watanabe M, Hirano A, Matsuda A, Monma Y, Numata T, Kusuyama H, Yaegashi N. Report on disaster medical operations with acupuncture/massage therapy after the great East Japan earthquake. Integr Med Insights. 2012;7:1-5.

180)Church D, Stapleton P, Yang A, Gallo F. Is tapping on acupuncture points an active ingredient in Emotional Freedom Techniques? A systematic review and meta-analysis of comparative studies. The Journal of nervous and mental disease. 2018 Oct 1;206(10):783-93.

경혈지압은 대체로 안전한 자가 관리법이다. 하지만 경혈지압이 원인을 근본적으로 해결하는 치료법은 아니기에 모든 증상을 해결해 줄 수는 없다. 그러므로 경혈지압을 지속해도 증상이 개선되지 않는다면 반드시 한의사와의 상담이 필요하다.

(2) 유의사항

쉽고 간단하여 누구나 시행할 수 있지만 지압하는 경혈의 위치가 정확하다면 더 좋은 효과가 나타날 수 있다. 본 매뉴얼의 사용자들은 한의약융합연구정보센터에서 제공하는 표준경혈DB(그림13)[181]를 참고하여 정확한 위치를 찾을 수 있으며 담당 한의사는 혈위에 대해 적절히 교육하고 피드백해야 할 것이다.

먼저 시술 전 안전하고 위생적인 시술을 위해 소독액 및 깨끗한 물을 활용하여 손을 소독하거나 씻는 것이 바람직하다.
손가락 끝마디의 지문 부위를 해당 경혈에 대고 천천히 누르고 뗀다. 자극 시간은 3초 정도이다. 경혈을 누르면 가벼운 압통이 느껴지는데 주위를 눌러 압통점을 찾는다.
힘을 주기 쉬운 엄지를 가장 많이 사용한다. 가볍게 누를 때는 검지나 중지를 사용한다. 자극에 민감한 부위(예: 얼굴)는 검지나 중지로 가볍게 누른다. 넓은 부위를 자극할 때는 여러 손가락을 동시에 사용하고 복부는 양 손가락을 겹쳐서 가볍게 누른다.
매 경혈당 지압 횟수는 5~10회 정도로 하고 시간은 약 3분 정도 시행한다. 하루 지압 횟수는 1~5회 정도로 상황과 개인의 상태에 맞추어 적절히 시행한다[182]. 필요시 적절한 자극 횟수와 시간에 대해 한의사와 상담한다.
경혈을 지압하면 시큰하고, 얼얼하고, 무겁고, 부은 듯한 느낌(산마중창酸麻重脹)이 나타나는데 이를 득기(得氣)라고 하며 제대로 된 유효한 자극을 판단하는 기준이 된다.
넓은 범위를 자극할 때 해당 경혈 부위와 근육을 손으로 문지르거나 쓰다듬거나(경찰법輕擦法), 잡고 압을 가하며 주무르거나(유날법 揉捏法), 주먹으로 두드리는(고타법叩打法) 다양한 마사지 방법을 활용할 수 있다(그림11). 이런 방법들은 추나 치료에서도 사용된다. 지압에 비해 넓은 범위를 자극하므로 힘의 강도는 약하다. 손등으로 문지르면 더 강한 자극을 줄 수 있다. 마사지는 혈액순환을 개선하여 손발이 붓거나 차거나 과긴장했을 때 효과적이다.

181)한의약융합연구정보센터. 표준경혈DB.[Cited: Jan 12, 2021] Available:https://www.kmcric.com/database/acupoint
182)다케노우치 미쓰시. 그림으로 쉽게 배우는 지압 비타민100. 중앙생활사(서울):2008. pp31.

그림 11 마사지법

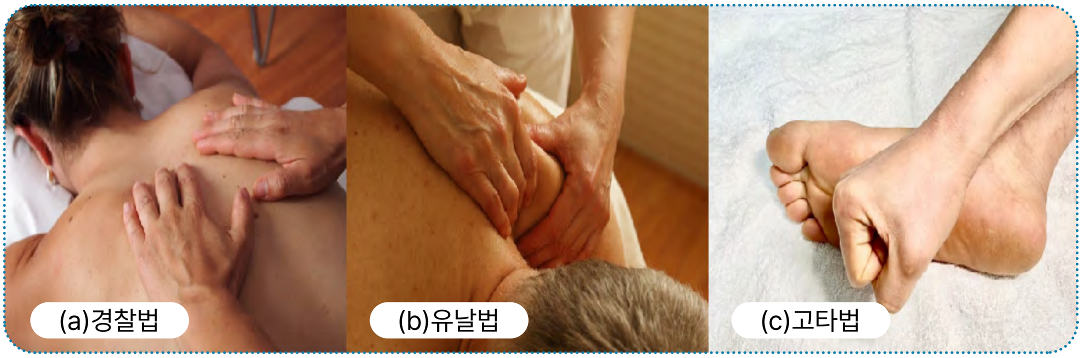

· 손가락보다 강한 자극을 주거나, 손이 닿기 어려운 부위나 넓은 부위를 자극하거나, 손가락 힘이 약해 자극을 지속하지 못하는 경우도 있다. 이때는 손가락 외에도 다양한 일상의 도구들을 활용할 수 있다. 머리핀이나 볼펜 혹은 이쑤시개를 여러 개 묶어 머리를 자극하거나, 빨래집게로 손가락이나 발가락에 강한 자극을 주거나, 골프공을 굴려 발바닥을 자극하거나, 나무 방망이로 어깨를 두드리거나, 머리빗으로 머리를 두드려 자극할 수 있다. 또한 핫팩이나 수건, 헤어드라이기를 활용하여 손쉽게 넓은 부위에 온열 자극을 줄 수 있다. 이런 도구들은 근처에서 손쉽게 구할 수 있으므로 재난 초기 의료지원이 제한적인 상황에서 활용가치가 높다. 그러므로 재난 생존자들에게 생필품 외에 다양한 지압 도구들을 지원해 준다면 생존자들의 자가 건강관리에 도움이 될 수 있을 것이다.

· 격렬한 운동, 음주, 목욕, 식사 직후, 고열, 출혈, 혈압이 지나치게 높을 때는 지압에 주의한다. 임신 중 과도한 경혈 자극은 유산이나 조산의 위험이 있으므로 한의사와 상의 후 주의해서 시행한다.

그림 12 한의약융합연구정보센터 홈페이지 링크(표준경혈DB)

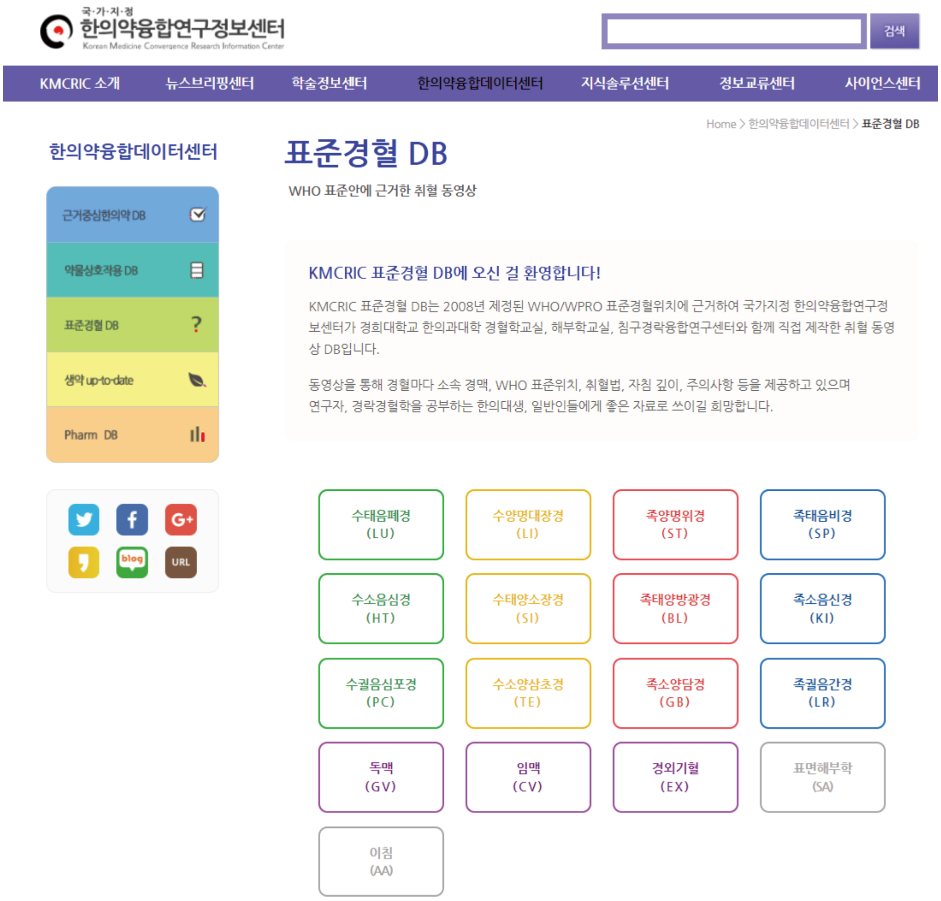

(3) 증상별 혈위[183)]

❶ 기본 11개 혈위 및 고려점

**심신을 안정시키는 혈위**	신문, 내관, 인당, 삼음교, 용천
**기혈을 순환시키는 혈위**	합곡, 풍지, 견정, 백회, 지실, 족삼리
**온열자극**	핫팩, 뜨거운 물수건 등을 활용할 수 있으며,<br>전기사용이 가능하다면 헤어드라이기를 활용할 수도 있다.

❷ 심리적 증상

**과긴장/불안**	신문, 내관
**불면**	신문, 용천, 안면, 삼음교
**우울**	백회, 합곡, 태충, 족삼리
**집중력 저하/건망증**	백회, 풍지, 은백
**분노/과민**	태충, 삼음교

183) 대한침구의학회 교재편찬위원회 . 침구의학. 제2장 각론편. 서울(한미의학);2020.

❸ 신체적 증상

**근골격계 통증**	재난 충격으로 인한 과긴장과 불편한 대피소 생활로 인해 근육이 경직되어 다양한 통증이 발생할 수 있다. 외상후스트레스장애에서도 통증이 흔히 동반된다.<br>- 목어깨 통증 : 견정, 풍지, 후계, 수침(手鍼)의 경항점, 견점<br>- 허리 통증 : 지실, 중봉, 금문, 수침(手鍼)의 제1,2요퇴점<br>- 무릎 통증 : 슬안, 족삼리, 양릉천<br>- 근육경련(쥐내림) : 족삼리, 양릉천
**두통**	교감신경계 흥분으로 인한 긴장과 재난 초기의 혼란스러운 상황으로 두통을 호소할 수 있다.<br>- 합곡, 태양, 풍지 : 강하게 자극한다.<br>- 손가락의 근위지관절 : 수침(手鍼)의 전두점(검지), 두정점(중지), 편두점(약지), 후두점(소지)

그림 13 수침 혈위

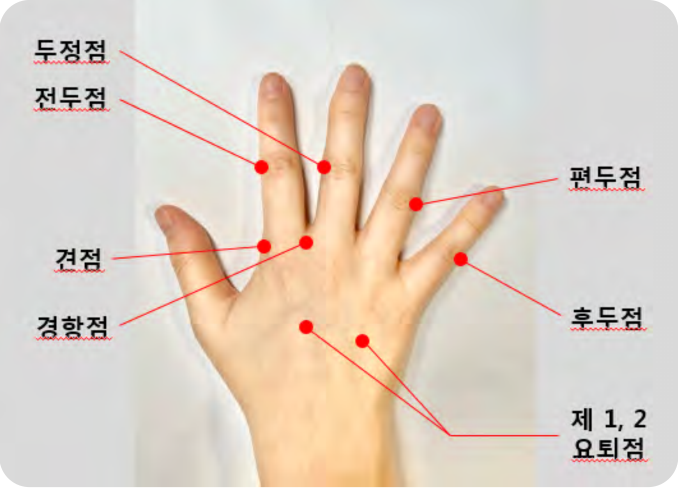

**어지럼증**	땅이 흔들리는 지진 같은 자연재난 경험 시 어지럼증이 흔히 발생할 수 있다.<br>- 족규음, 태양, 풍지
**눈의 피로/시력감퇴**	재난 충격과 혼란스러운 환경으로 감각 조절 기능이 저하되어 시야가 좁아지거나 눈이 침침해질 수 있다.<br>- 찬죽, 어요, 정명, 승읍<br>- 정명 : 엄지와 검지로 좌우 경혈을 잡고 감싸듯 동시에 누른다.

**감기 증상**	좁은 공간에 밀집도가 높으며 위생상태가 열악한 대피소 단체 생활은 다양한 상기도 감염 증상이 발생하기 쉽다. 특히 겨울철 대피소는 난방을 위해 보일러가 아닌 온풍기를 사용하므로 매우 건조하다.<br>- 콧물, 재채기 : 영향, 태연(온열 자극), 합곡<br>- 코막힘 : 영향, 대추, 아문<br>- 오한, 발열 : 풍문, 천주, 풍지(온열 자극)<br>- 기침 : 천돌, 공최<br>- 가슴 두근거림/숨 가쁨 : 내관, 단중
**식욕부진, 위통**	- 중완, 족삼리, 신궐, 천추(온열 자극)
**배변**	대피소 생활로 인한 생활리듬의 변화, 식습관의 불균형, 섭취량의 저하, 운동 부족, 스트레스로 인해 변비가 발생할 수 있다.<br>- 설사, 복통: 족삼리 / 신궐, 천추(온열 자극)<br>- 변비: 은백, 여태, 대돈[184]
**피로/나른함**	불편한 대피소 생활로 섭취량 저하, 운동 부족, 스트레스, 우울증으로 인해 피로, 나른함을 호소할 수 있다.<br>- 족삼리, 은백, 여태, 용천(주먹으로 50~100회 두드린다.)
**(여성) 월경통**	삼음교
**(소아) 도한**	밤에 자주 깨서 울고 짜증이 심하고 밤에 땀이 너무 많다<br>- 신주, 명문(등 부위를 전체적으로 부드럽게 쓰다듬는다.)

184)대한침구의학회 교재편찬위원회. 침구의학. 서울(한미의학):2020. p715

표 23 증상별 혈위

증상명			혈위
심리적 증상	불안/초조		신문, 내관
	불면		신문, 용천, 안면, 삼음교
	우울		백회, 합곡, 족삼리
	집중력 저하/건망증		백회, 풍지, 은백
	분노/과민		태충, 삼음교
신체적 증상	근골격계 통증	목어깨	견정, 풍지, 후계, 수지침의 제1,2경항점
		허리	지실, 중봉, 금문, 제1,2요퇴점
		무릎	슬안, 족삼리, 양릉천
		근육경련(쥐 내림)	족삼리, 양릉천 / 손가락의 근위지관절: 전두점(검지), 두정점(중지), 편두점(약지), 후두점(소지)
	두통		합곡, 태양, 풍지
	어지럼증		족규음, 태양, 풍지
	눈의 피로/시력감퇴		찬죽, 어요, 정명, 승읍
	감기 증상	콧물, 재채기	영향, 태연, 합곡
		코막힘	영향, 대추, 아문
		오한, 발열	풍문, 천주, 풍지
		기침	천돌, 공최
	가슴 두근거림/숨 가쁨		내관, 단중
	식욕부진, 위통		중완, 족삼리 / 신궐, 천추
	배변	설사, 복통	족삼리 / 신궐, 천추
		변비	은백, 여태, 대돈
	피로/나른함		족삼리, 은백, 여태, 용천
	(여성) 월경통		삼음교
	(소아) 밤에 자주 깨서 울고 짜증이 심하고 땀이 너무 많다.		신주, 명문

3) 도인운동

한의학 전통치료법인 도인운동법은 영추·병전편(靈樞·病傳篇)에 기록된 '도인안교'에서 그 기원을 찾을 수 있다. 도인운동법은 질병 치료 및 양생건강법으로 사용되는 치료적 운동으로 호흡과 함께 적극적으로 몸을 움직인다[185). 도인은 호흡과 동작을 동시에 시행하여 인체의 기혈순환을 촉진시키고 체내의 사기를 몸 밖으로 배설시키며[186), 오장육부의 기능을 조절하고, 정신을 안정시키며, 경락을 소통시키고, 근육과 뼈를 튼튼히 하는 작용이 있다[187).

본래 도인에서 도(導)는 호흡을 통해 대기의 기를 안으로 끌어들인다는 뜻이고 인(引)은 잡아 늘인다는 말로 인체의 굴신작용을 의미한다. 즉 도인은 인체 내로 끌어온 기를 이용하여 인체 각부의 기능을 활발히 하는 것으로 단순한 신체 단련이 아닌 정신수련의 의미를 가지고 있다는 점에서 서양의 운동요법과 차이가 있다[188). 도인기공체조가 성인여성의 견비통[189)과 퇴행성 슬관절염[190)에 효과가 있고, 요통환자의 통증과 기능개선 및 삶의 질 향상[191)에도 유효성이 있었다는 연구들이 발표된 바 있다.

도인운동은 사지의 움직임을 통해 척추를 움직이고, 척추의 움직임을 통해 내장을 흔들어 주어 이를 통해 근골격계의 구조를 바르게 하고 내부 장기에 원활한 기혈 순환을 촉진한다[192)193)194). 특히 공간을 많이 차지하지 않으므로 재난현장에서 장기간 대피소 생활로 인하여 활동이 제약되고 신체적 통증과 기능저하 등이 발생한 상황에서 활용성이 높다. 자가훈련법의 일종으로 혹은 그룹치료의 형태로 활용하여 재난 생존자들의 통증과 기능을 개선하여 삶의 질을 향상하는데 도움을 줄 수 있다.

---

185)The society of korean medicine rehabilitation. Oriental rehabilitation medicine. 3rd ed. Seoul: Koonja publishing company; 2011. p.306.

186)Hong WS, Jing-Jiao-Hung-Di-Nei-Jing, Seoul:Research of Oriental Medicine. 1985:198.

187)Kim HT. A Documentational Study of Doinqigong in The Oriental Medicine Classics. Journal of Korean Medical classics. 2009:22(3):9-18.

188)Lee EJ, Park IS, Oh MS. Effects of the Daoyin and Corrective Exercise Program on Musculoskeletal Pain -Based on the Acute Sprain Patients due to Traffic Accidents-. Journal of Korean Medicine Rehabilitation. 2011;21(4):205-17.

189)Youn HM, Kim MY, Kim YS, Lim JS. Effects of the Doin Gigong Exercise on the Shoulder-Arm Pain in Women. The Journal of Korean Acupuncture & Moxibustion Society, 2005;22(1):243-8.

190)Lee JW. Effect of exercise treatment through Dong Bang Hwal Bup and dao in yang seng gong in degenerative knee arthritis. Official Journal of the Korean Association of Certified Exercise Professionals. 2006;8(2):129-36.

191)Choi BS, Lee EJ, Li YC, Lee JM, Kim ES, Song GC, Jung IC, Oh MS. Effect of The Daoyin Exercise Therapy Combined with Complex Korean Medicine Treatment on Pain and Function Improvement of Low Back Pain Patients : A Retrospective Observational Study. J Physiol & Pathol Korean Med 2018;32(1):88-97.

192)황의형, 양창섭. 의료기공학. 부산시, 부산대학교 출판부, 2013, pp21-99,

193)구대근, 박종학, 한창희, 엄기영, 강수원. 고식화타오금희. 서울시, 도서출판 밝은빛, 2010, pp 12-20.

194)Hwang EH, Kwon YK, Heo KH, Cho HW, Lee HY, Sung WY. Comparative Review of Qigong and Daoyin as a Therapeutic Exercise of Traditional Korean Medicine. Korean J. Oriental Physiology & Pathology 2013;27(5):594-601,

(1) 동의보감 [안마도인]

그룹에서 다 같이 따라하도록 교육하거나 동영상을 활용하여 개인이 일상에서 꾸준히 수행하도록 독려한다. 스트레스를 해소하는 동작을 강조하며, 전신을 이완시키고, 자세 교정에 도움이 되는 동작을 위주로 구성되어 있다.

제시한 유튜브의 동영상을 활용하거나 한의사가 동작을 숙지하여 직접 시연하며 진행한다.

그림 14 한국건강증진개발원 유튜브 링크(동의보감 안마도인)

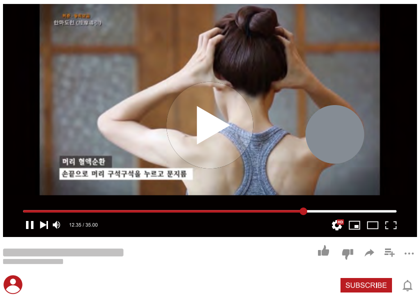

표 24 안마도인

연번	부위	방법	효능
1	머리	손끝으로 머리 구석구석을 누르고 문지름	머리 혈액순환
2	이마	손끝으로 머리털 난 곳에서 아래로 10번씩 쓸어주기, 관자놀이 문지르기	얼굴 윤택, 심
3	눈	손바닥 비벼 뜨겁게 하여 양 눈을 10번 비벼주기,<br>눈 돌리기 5번	눈밝음, 간
4	코	코의 좌우를 가운데 손가락으로 위아래로 10번 문지르기, 숨쉬기 5번	코, 폐 강화
5	귀	오른손으로 머리 위를 거쳐서 왼쪽 귀를 5번 잡아당김, 반대로 5번.<br>양 손으로 귀바퀴 10번 문지르기	총명, 난청 예방,<br>신 강화. 미주신경 자극
6	이, 혀	이를 5번 부딪치고, 잇몸 손끝으로 문지름<br>혀를 돌려 침이 입안에 나오게 하여 삼킴	잇몸, 비위강화
7	목	오른손으로 왼쪽 목 근육(흉쇄유돌근,사각근)을 10번 잡아당김, 반대로 10번	목 혈액순환
8	뒷목	고개 들어 머리를 좌우로 돌림 10번, 손은 허리에	뒷목 이완
9	어깨	양 어깨를 위로 힘차게 으쓱거림 10번	어깨긴장 이완,<br>견갑거근, 승모근
10	가슴	오른손을 갈퀴처럼 하여 쇄골부터 갈비뼈까지 바깥에서 안쪽으로 쓸어줌 10번, 반대로 10번 실시	가슴, 심폐 혈액순환
11	흉골	양손을 모아서 위에서 아래로 쓸어내림 10번(코로 크게 숨쉬면서) 실시	스트레스 해소
12	배	양손을 모아서 시계방향 원을 그리며 쓸어줌 10번(장운동 방향) 실시	내장 순환, 소화
13	허리	등배운동 뒤로 5번, 손은 허리에	허리
14	엉덩이	양손으로 허리 엉치 따뜻하게 비벼주기 10번	엉치 강화
15	항문조이기	항문 조이기 5번	생식기
16	허리돌리기	양손 깍지 끼고 허리를 좌우로 돌림 10번	허리
17	코숨달리기	코로 숨쉬며 제자리 달리기1분,<br>팔치기를 뒤로 힘차게 함	어깨, 전신이완
18	마무리 호흡	호흡 10회	전신이완

(2) 도인운동체조[195]

뇌와 전신을 자극하여 기혈순행을 촉진하여 심신을 안정시키고 몸을 튼튼히 하는 방법으로 도인(導引), 또는 교인(撟引)이라고도 하며, 정신을 집중하는 법, 침을 삼키는 법, 숨을 조절하는 법, 힘을 쓰는 법, 운동하는 법, 손으로 만지고 비비는 등 여러 가지 방법이 사용된다.
도인법은 몸의 동작보다는 호흡과 의념에 중점을 두는 것이 특색이며, 기를 모으고 확장하는 기공 동작이 포함되어 기운의 축적에도 도움이 될 수 있다. 기혈 순환을 촉진하고 근육과 뼈를 튼튼하게 하며 피로를 해소하며 심신을 강건하여 질병 예방에 도움이 될 수 있다.

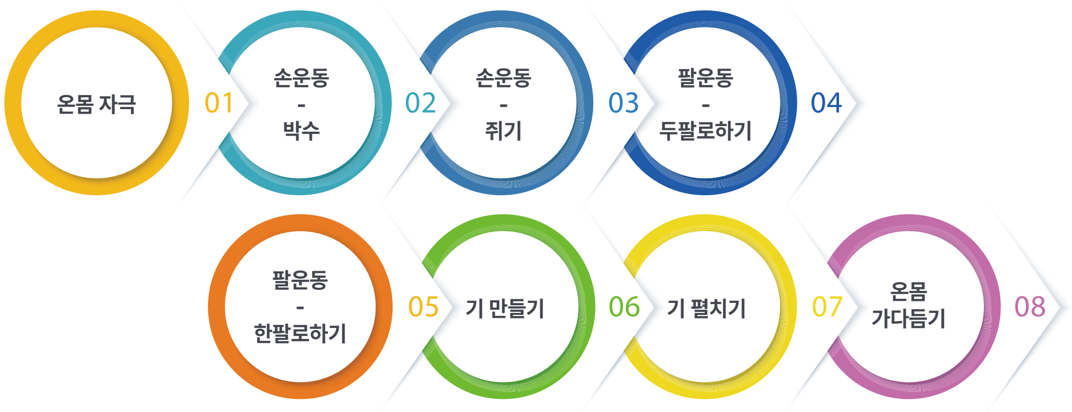

❶ 온몸 자극 : 어깨 회전 범위 확대, 상체 혈액순환 촉진 및 뇌 자극
- 머리박수 : 손가락 끝을 세워 머리를 경쾌하게 두드려줌[196]
- 어깨박수 : 양손으로 어깨를 두드림
- 엉덩이박수 : 양손으로 엉덩이를 두드림
- 세로박수 : 양손을 세로로 세워 박수를 침

❷ 손운동 - 박수 : 말초신경 자극, 혈액순환 촉진
- 주먹박수 4회 + 세로박수 4회
- 손끝박수 4회 + 세로박수 4회
- 손바닥박수 4회 + 세로박수 4회
- 손목박수 4회 + 세로박수 4회

195)중앙치매센터. 2018 나에게 힘이 되는 치매가이드북. 중앙치매센터. 보건복지부. 2018:19-31p
196)제병원후론 양생방도인법. 두면풍후(頭面風候) 令髮不白. 以手復捋頭五, 通脈也와 유사한 동작

❸ 손운동 - 쥐기 : 운동능력 향상
- 세로박수 : 양손을 맞대어 강하게 박수
- 가로박수 : 양손을 수평이 되도록 눕혀 박수
- 가로 쥐기 : 양손을 수평으로 맞댄 상태에서 손을 꼭 쥐어줌
- 깍지 끼기 : 양손을 서로 마주 놓고 힘껏 깍지

❹ 팔운동 - 두팔로 하기 : 상체 혈액순환 촉진, 운동능력 향상
- 두 팔 앞으로 밀기[197)]
- 두 팔 위로 밀기[198)]
- 두 팔 옆으로 밀기
- 두 팔 교차하여 밀기

❺ 팔운동 - 한팔로 하기 : 상체 혈액순환 촉진, 운동능력 향상
- 한 팔씩 번갈아 밀기 : 오른손을 시작으로 앞, 위, 옆, 사선으로 한 팔씩 밀고 돌아오기를 반복

❻ 기 만들기 : 후두엽, 두정엽 및 전두엽의 활성화
- 기운 모으기
  ① 가슴 아래쪽에 양손으로 위 아래로 위치시키고 손가락을 둥글게 말아 쥠
  ② 왼손이 위로 향하도록 돌려줌
  ③ 왼손이 위쪽에 위치하면 다시 오른손이 위로 향하도록 천천히 돌려줌

그림 15 기운 모으기

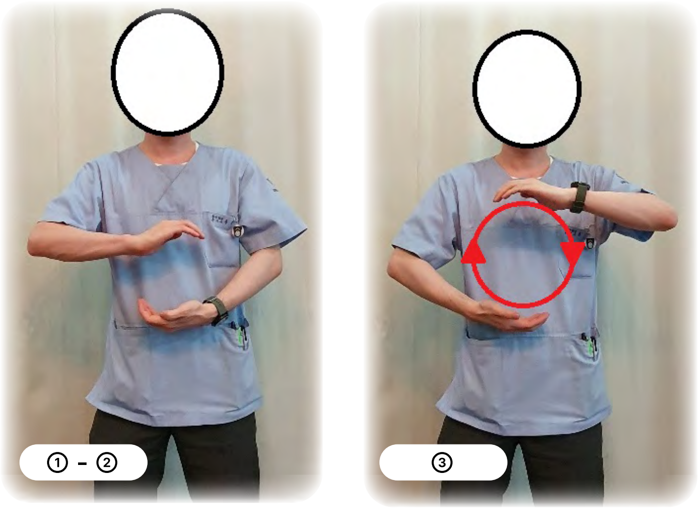

197)제병원후론 양생방도인법. 허로후(虛勞候) 兩手抱兩乳, 急努, 前後振搖, 極勢二七와 유사한 동작
198)제병원후론 양생방도인법. 허로후(虛勞候) 兩手長舒, 掌相向腦項之後와 유사한 동작

- 기운 키우기
  양손을 자신의 몸통 크기로 넓혀주어 같은 방법으로 천천히 돌려줌
- 기운 크게 키우기
  양 팔을 위아래로 길게 뻗어

그림 16 기운 키우기 및 기운 크게 키우기

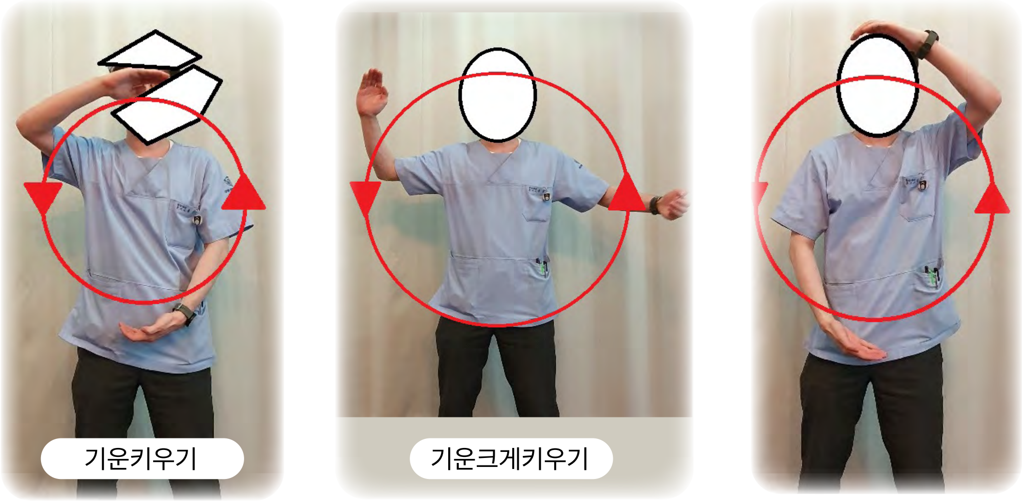

- 기운 펼치기
  양 팔을 위아래로 길게 뻗어 손바닥이 밖을 향하도록 하여 천천히 돌려줌

그림 17 기운 펼치기

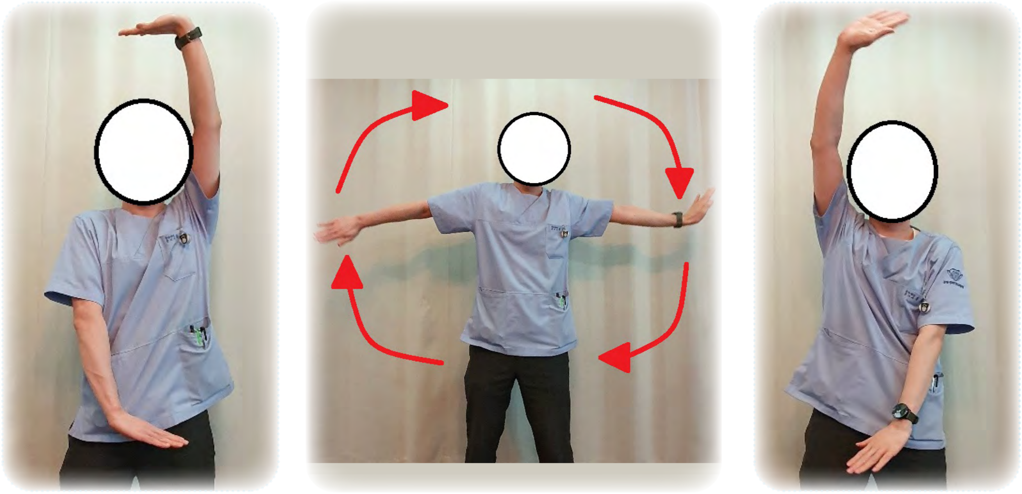

❼ 기 펼치기 : 후두엽, 두정엽 및 전두엽의 활성화

- 밑 면 동그라미 그리기
  ① 양손은 볼펜을 쥐듯이 가볍게 모아 허리에 위치
  ② 허리를 기준으로 밑면에 그림을 그리듯 동그라미를 그려줌
  ③ 오른손과 왼손을 번갈아가면서 동그라미를 그려줌

그림 18-1 밑 면 동그라미 그리기(위)

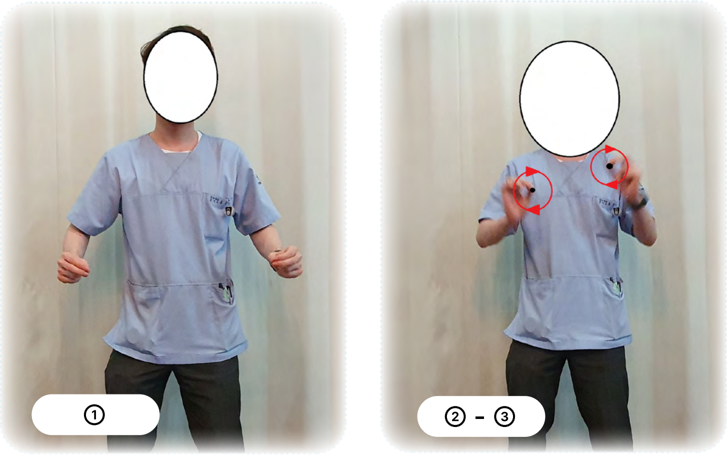

- 앞 면 동그라미 그리기
  ① 양손은 볼펜을 쥐듯이 가볍게 모아 허리에 위치
  ② 허리를 기준으로 앞면에 그림을 그리듯 동그라미를 그려줌
  ③ 오른손과 왼손을 번갈아가면서 동그라미를 그려줌

그림 18-2 앞 면 동그라미 그리기(아래)

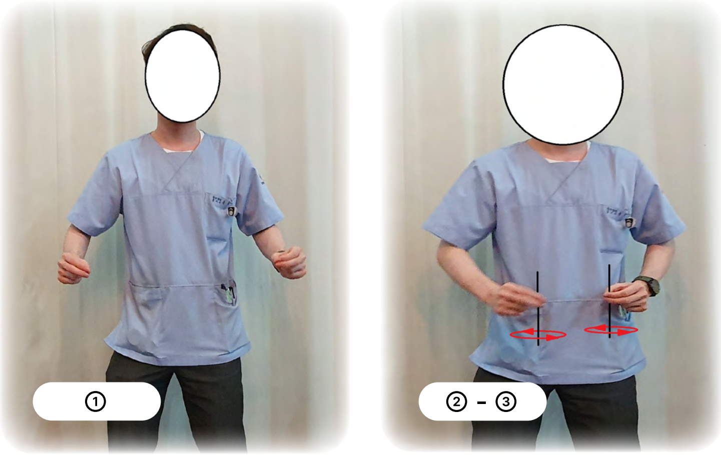

- 앞과 옆면에 동그라미 그리기
  ① 양손은 볼펜을 쥐듯이 가볍게 모아 왼손은 옆면에 오른손은 앞면에 위치
  ② 양손으로 동시에 동그라미를 그려줌
  ③ 양손은 볼펜을 쥐듯이 가볍게 모아 왼손은 앞면에 오른손은 옆면에 위치
  ④ 양손으로 동시에 동그라미를 그려줌

그림 19 앞과 옆면에 동그라미 그리기

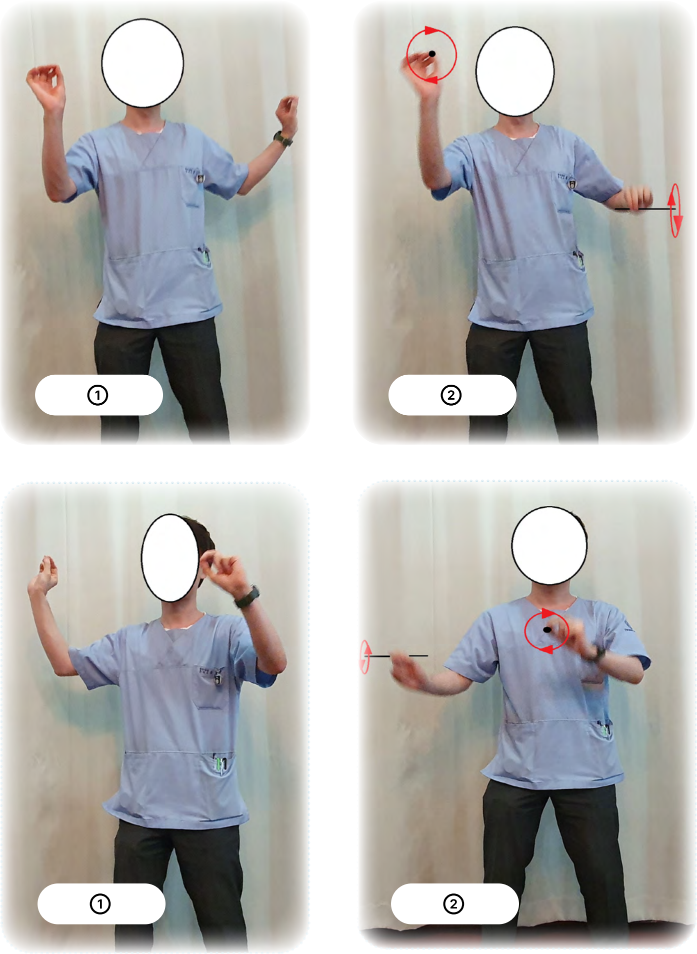

❽ 온몸 가다듬기 : 어깨 및 가슴근육 이완

- 크게 숨 들이마시기
  가슴을 넓게 펴고 팔을 위로 올려 숨을 들이마시기
- 크게 숨 내쉬기
  팔을 아랫배 위로 내리면서 숨을 내쉼
- 숨 들이마시기
  손바닥이 위로 오게 하여 손끝을 마주 보게 가슴 쪽으로 올리며 숨을 들이마시기
- 숨 내쉬기
  마주한 손을 내리며 숨을 내쉼

그림 20 온몸 가다듬기

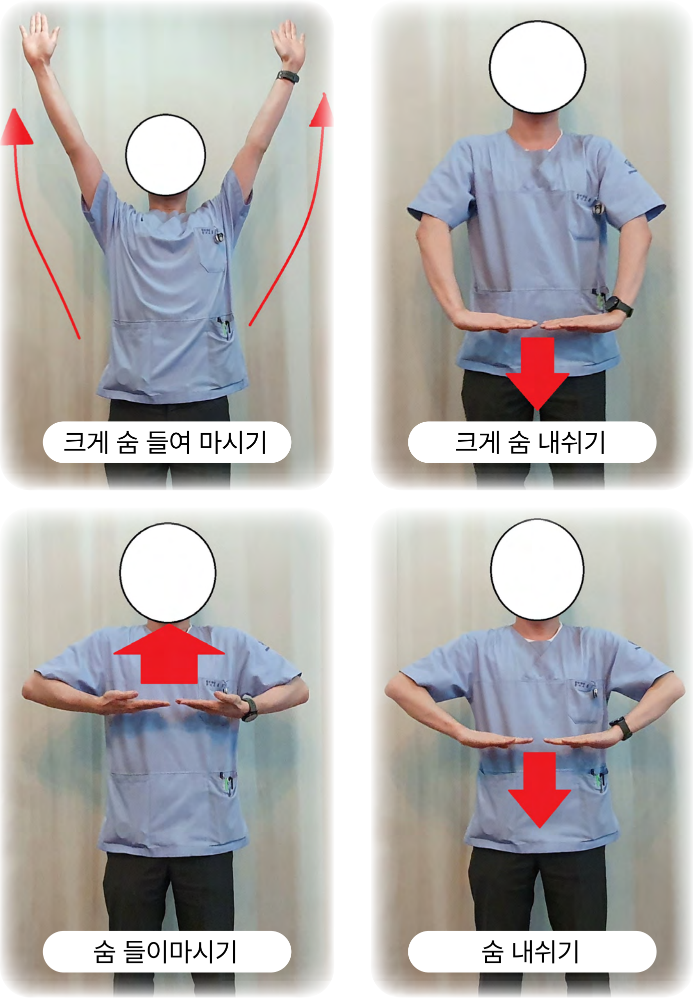

4) 걷기명상법

걷기 명상은 걸으면서 느껴지는 모든 감각에 주의를 기울이고, 안정감을 느낄 수 있는 동적 명상으로, 우울 증상을 경감시키고, 건강 기능 상태와 혈관 반응성 개선에 효과적이라고 보고되어[199], 재난 지원 현장에서 유용하게 활용될 수 있다. 특히, 이 명상 방법은 고강도의 신체 활동을 필요로 하지 않기 때문에, 신체 질환이 있거나 노인 환자라도 일정 수준 이상의 자가 보행능력이 있다면 안전하게 시행할 수 있다.

재난 지원 현장에서 한의사가 환자들과 함께 시행하거나 교육하여 활용할 수 있는 간단한 걷기 명상(걸으면서 하는 명상)을 소개한다. 대한한의사협회 유튜브 채널(AKOM_TV) 또는 강동경희대병원 한방신경정신과 유튜브 채널에서 제공하는 영상을 참고한다. 호흡명상은 한의상담치료 중 안정화기법 편을 참고한다.

그림 21 걷기명상 유튜브 링크

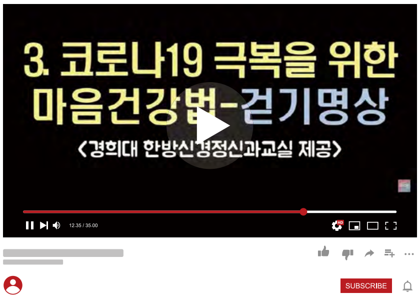

199) Prakhinkit S, Suppapitiporn S, Tanaka H, Suksom D. Effects of Buddhism walking meditation on depression, functional fitness, and endothelium-dependent vasodilation in depressed elderly. J Altern Complement Med. 2014 May;20(5):411-6.

걷기 명상

자, 이제 걷기 명상을 시작하겠습니다.
걷기 명상을 하기 좋은 공간은 걸을 수 있는 실내의 조용한 공간, 또는 야외도 좋습니다.
다만 주위에 부딪힐 만한 물건이 있거나, 자동차, 오토바이, 자전거가 다닐 수 있는 공간은 피합니다.

자, 이제 두 발을 땅에 대고 바로 섭니다.
두 발은 안정감을 느낄 수 있을 정도로 한 뼘이나 두 뼘 정도 떨어뜨려 섭니다.
양 팔은 편하게 몸 양쪽으로 늘어뜨립니다.

눈을 가만히 감습니다.
하지만 균형을 잡기 어렵거나 어지러우신 분들은
눈을 뜨고 있어도 좋습니다.

천천히 호흡을 3번 합니다.

두 발바닥 면이 땅바닥에 붙어있는 느낌을 확인합니다.
땅바닥이 딱딱한지, 물컹한지, 평평한지, 울퉁불퉁 한지, 차가운지, 따뜻한지.
그 땅바닥에 발바닥이 마치 뿌리처럼 붙어
안정적으로 서있는 자기 자신을 느껴봅니다.

천천히 호흡을 하면서 자신의 움직임도 확인합니다.
마치 바람이 불어 살랑살랑 움직이는 나무처럼
자신의 몸도 조금씩 움직이며 균형을 잡고 있는 것을 확인합니다.
땅바닥과 닿아있는 발바닥 면에서
균형을 잡기 위해 압력이 느껴지는 부위가 조금씩 달라지는 것을 확인합니다.

발뒤꿈치, 발바닥 중앙, 발바닥 바깥 면, 발바닥 앞쪽, 발가락
엄지발가락부터 새끼발가락까지
발바닥의 어떤 부분에서 압력이 느껴지는지 확인합니다.

그렇게 발바닥을 땅바닥에 뿌리내려
안정적으로 서 있는 자기 자신을 관찰합니다.

이번에는 천천히 걸어볼 것입니다.
곧바로 동작으로 옮기지는 않습니다.
머릿속으로 이제 걸음을 걸어야겠다는 생각을 먼저 하고,
그 생각을 행동으로 옮깁니다.

천천히 한쪽 발을 듭니다.
발과 다리의 감각이 시시각각 변화하는 것을 관찰해보세요.
땅바닥에 붙어있는 발에서 압력이 늘어나는 것을 관찰합니다.
발목이 흔들거릴 수도 있습니다.
정강이나 종아리에 힘이 들어가지는 않는지 확인합니다.

들었던 한쪽 발을 서서히 앞으로 내밉니다.
몸 전체적으로 흔들거리는 것을 관찰합니다.
빨리 발을 땅에 딛고 싶은 생각은 없는지,
몸이 흔들거려서 불안한 생각은 없는지,
자신의 내면에서 올라오는 생각도 관찰합니다.

앞쪽으로 내민 발을 발뒤꿈치부터 서서히 땅에 댑니다.
발뒤꿈치, 발바닥 중앙, 발바닥 바깥 면, 발바닥 앞쪽, 발가락
서서히 땅바닥에 발이 닿는 느낌을 관찰합니다.
흔들리던 내 몸도 안정되어 있음을 확인합니다.
무게 중심이 양쪽 다리로 분산되는 것을 확인합니다.
지금 발과 발목, 정강이, 종아리, 허벅지에서 느껴지는 감각은 어떤가요?

같은 방법으로 이번에는 반대쪽 발을 천천히 듭니다.
발과 다리의 감각이 시시각각 변화하는 것을 관찰해보세요.
땅바닥에 붙어있는 발에서 압력이 늘어나는 것을 관찰합니다.
발목이 흔들거릴 수도 있습니다.
정강이나 종아리에 힘이 들어가지는 않는지 확인합니다.

들었던 한쪽 발을 서서히 앞으로 내밉니다.
몸 전체적으로 흔들거리는 것을 관찰합니다.
빨리 발을 땅에 딛고 싶은 생각은 없는지,
몸이 흔들거려서 불안한 생각은 없는지,
자신의 내면에서 올라오는 생각도 관찰합니다.

앞쪽으로 내민 발을 발뒤꿈치부터 서서히 땅에 댑니다.
발뒤꿈치, 발바닥 중앙, 발바닥 바깥 면, 발바닥 앞쪽, 발가락
서서히 땅바닥에 발이 닿는 느낌을 관찰합니다.
흔들리던 내 몸도 안정되어 있음을 확인합니다.
무게 중심이 양쪽 다리로 분산되는 것을 확인합니다.
지금 발과 발목, 정강이, 종아리, 허벅지에서 느껴지는 감각은 어떤가요?

지금과 같은 방법으로 천천히 5분에서 10분 정도 걸어봅니다.
동그랗게 원을 그리면서 돌아도 괜찮고,
한쪽 방향으로만 걸어가도 괜찮습니다.
또 방향을 바꾸어 걸어가도 괜찮습니다.

걸으면서 느껴지는 모든 감각들에 주의를 기울이면서
걷기를 해보십시오.

...

(마무리) 자 이제 동작을 멈추겠다는 생각을 떠올린 뒤,
그 생각을 행동으로 옮깁니다.

처음 시작했던 것처럼 두 발을 디디고 바로 섭니다.
두 발은 안정감을 느낄 수 있을 정도로 한 뼘이나 두 뼘 정도 떨어뜨려 섭니다.
양 팔은 편하게 몸 양쪽으로 늘어뜨리고 있습니다.

두 발바닥 면이 땅바닥에 붙어있는 느낌을 확인합니다.
안정적으로 서있는 자기 자신도 느껴봅니다.

천천히 호흡을 하면서 느껴지는 자신의 움직임.
발과 다리의 무게중심이 옮겨가며
자신의 몸이 균형을 잡고 있는 것을 확인합니다.
잠시 동안 충분히 그 안정감을 느껴봅니다.

이제 천천히 호흡을 3번 하고 걷기 명상을 마무리하겠습니다.
수고하셨습니다.

IV

부록

01 외상후스트레스장애 진단기준
(DSM-5)

02 진료기록부
(예시)

03 개인정보 수집 및 이용 동의서
(예시)

04 외상후스트레스장애 선별검사
(The Korean Version of the Primary Care PTSD Screen for DSM-5, K-PC-PTSD-5)

05 우울증 선별검사
(Patient Health Questionnaire, PHQ-9)

06 불안 설문지
(Generalized Anxiety Disorder 7-item scale, GAD-7)

07 건강질문지-15
(Patient Health Questionnaire, PHQ-15)

08 자살위험성 질문지

09 그룹 치료 예시

10 감정자유기법 자가 치료 학습지

IV. 부록

부록1. 외상후스트레스장애 진단기준 (DSM-5)

외상후스트레스장애 진단기준 (DSM-5)

자세한 진단 기준은 시대별로 다르고, 미국 정신의학회와 세계보건기구의 기준도 다름
그러나 일반적으로 다음과 같은 내용을 포함하고 1개월 이상 증상이 지속되면 진단될 수 있음

A. 실제 죽음이나 죽음에 대한 위협, 심각한 부상 또는 성폭력에 노출됨

1) 트라우마성 사건을 직접 경험
2) 타인에게 일어난 사건을 직접 목격
3) 가까운 가족 혹은 가까운 친구에게 사건이 일어난 것을 알게 됨(가족이나 친구가 실제로 사망하거나 이에 준하는 상황을 경험한 경우, 사건은 반드시 폭력적이거나 갑작스럽게 일어난 일이어야 함)
4) 트라우마성 사건의 혐오스러운 세세한 내용에 대한 반복적이거나 심한 정도로 노출을 경험함 (예: 사체를 수습하는 긴급 구조 대원들, 아동 학대의 자세한 내용에 반복적으로 노출된 경찰관)

주의 : 4) 항목은 그 노출이 직업과 관련되지 않으며 전자 매체, 텔레비전, 영화 혹은 사진을 통한 노출에는 적용되지 않음.

B. 트라우마성 사건 관련 침습적 증상들이 한 가지 이상 나타남

1) 반복적, 불수의적 그리고 침습적이고 괴로운 트라우마 기억
2) 사건과 관련된 반복적인 괴로운 꿈
3) 사건이 다시 일어나는 듯이 느끼거나 행동하는 해리 반응(예: 플래시백)이 나타남(이런 반응은 다양하게 나타나며 심한 경우에는 현재 상황을 전혀 인식 못 할 수 있음)
4) 사건의 일부와 유사하거나 상징하는 자극에 노출되었을 때 나타나는 강력하거나 지속적인 심리적 고통
5) 사건의 일부와 유사하거나 상징하는 자극에 노출되었을 때 나타나는 현저한 생리적 작용

C. 관련된 자극에 대한 지속적인 회피가 시작되고, 다음의 한 가지 혹은 두 가지 증상이 나타남

1) 트라우마성 사건과 관련된 고통스러운 기억, 생각 혹은 감정을 회피하거나 회피하려고 노력함
2) 트라우마성 사건과 고통스러운 기억, 생각 혹은 감정을 회상시키는 외부 요인들(예 : 사람, 장소, 대화, 활동, 물건, 상황)을 회피하거나 회피하려고 노력함

D. 트라우마성 사건이 일어난 뒤에 시작되거나 악화되는 사건과 관련된 인지와 기분의 부정적인 변화가 다음 중 두 가지 이상 나타남

1) 트라우마성 사건의 중요한 측면을 기억할 수 없음
   - 전형적으로 해리성 기억상실 때문임
   - 두부 손상, 알코올 혹은 약물과 같은 다른 요소들은 제외
2) 자신과 타인 또는 세상에 대한 지속적이고 지나친 부정적 믿음이나 기대
   - 예) “나는 나쁘다”
   - “누구도 믿을 수 없다.”
   - “세상은 모든 것이 위험하다.”
   - “나의 모든 신경계가 완전히 망가졌다.”
3) 트라우마성 사건의 원인이나 결과에 대한 자신이나 타인을 비난하게 되는 지속적이고 왜곡된 인지
4) 지속적인 부정적 감정(예: 두려움, 공포, 분노, 죄책감 혹은 수치심)
5) 의미 있는 활동에 흥미를 갖거나 참여하는 것이 심하게 감소함
6) 타인들로부터 고립되었거나 소외된 느낌
7) 긍정적인 감정을 지속적으로 경험할 수 없음 (예: 행복, 만족감 혹은 사랑하는 감정을 경험할 수 없음)

E. 트라우마성 사건과 관련된 각성과 반응의 심한 변화가 다음에서 두 가지 이상 사건이 일어난 뒤에 시작되거나 악화됨

1) 전형적으로 타인이나 물체에 대한 언어적 또는 신체적 공격으로 표현되는 과민한 행동과 분노 폭발(유발 인자가 거의 혹은 전혀 없음)
2) 무모하거나 자기 파괴적인 행동
3) 과각성
4) 과도한 놀람 반응
5) 집중의 어려움
6) 수면장애(예: 잠들기 어렵거나 잠을 계속 자기 어렵거나 편히 잠을 못 잠)

※ 급성 스트레스장애

- 외상 후 스트레스장애와 증상은 같으나 외상경험 후 4주 이내에 증상이 나타나고 증상 지속기간이 4주이내인 상태.

부록2. 진료기록부(예시)

<table>
<tr><th colspan="6">인구학적 조사 및 기초 문진</th></tr>
<tr><td>성명</td><td colspan="2"></td><td>성별</td><td colspan="2">☐ 남 ☐ 여</td></tr>
<tr><td>나이</td><td colspan="2"></td><td>생년월일</td><td colspan="2">년 월 일</td></tr>
<tr><td>결혼 상태</td><td colspan="2">☐기혼 ☐미혼 ☐별거 ☐사별 ☐기타</td><td>키/몸무게</td><td colspan="2">cm kg</td></tr>
<tr><td>직업</td><td colspan="2"></td><td>발병일</td><td colspan="2">년 월 일</td></tr>
<tr><td>특이사항</td><td colspan="2">☐소아 ☐노인 ☐신체장애인
☐정신장애인 ☐임산부
☐기타( )</td><td>트라우마 종류</td><td colspan="2">☐생명위협 ☐신체부상 ☐목격(사망/신체부상) ☐인지(가족/친구의 사망)</td></tr>
<tr><td>활력 징후</td><td colspan="5">수축기/이완기 혈압(좌위): / mmHg 맥박(좌위): /min
체온: . °C 호흡수: /min</td></tr>
<tr><td>주소증</td><td colspan="5">(SUDs: __점)</td></tr>
<tr><td rowspan="3">현증상</td><td>신체적</td><td>정서적</td><td>인지적</td><td colspan="2">행동적</td></tr>
<tr><td>☐ 두통
☐ 어지럼증
☐ 불면
☐ 식욕저하
☐ 소화불량
☐ 가슴답답함
☐ 가슴두근거림
☐ 피로
☐ 통증</td><td>☐ 불안
☐ 공포
☐ 슬픔
☐ 우울
☐ 절망
☐ 분노
☐ 수치심
☐ 죄책감</td><td>☐ 집중력저하
☐ 기억력저하
☐ 악몽
☐ 끔찍한 기억이 자꾸 떠오름
☐ 가까운 사람의 죽음을 받아들이지 못함
☐ 죽음에 대한 몰두</td><td colspan="2">☐ 혼미
☐ 회피/부인
☐ 대인기피
☐ 자해
☐ 퇴행
☐ 폭력/폭언
☐ 과도한 음주/흡연
☐ 대처능력 저하</td></tr>
<tr><td colspan="5">기타 증상:</td></tr>
<tr><td>정신과 치료 경험</td><td colspan="5">☐ 있음: 진단명( ) 복용 약물( ) ☐ 없음</td></tr>
<tr><td>복용약물</td><td colspan="5"></td></tr>
<tr><td>흡연</td><td colspan="2">__갑/일 * __년</td><td>음주</td><td colspan="2">주_회 * 주량_____ *_ 년</td></tr>
<tr><td>상병명</td><td colspan="5"></td></tr>
<tr><td>선별검사</td><td>외상후스트레스(PC-PTSD-5): __점</td><td>우울(PHQ-9): __점</td><td>불안(GAD-7): __ 점</td><td>신체증상(PHQ-15): __점</td><td>자살(P4): __점</td></tr>
<tr><td>위험요인</td><td>정신과적 과거력 ( )</td><td>과거 트라우마 경험 ( )</td><td>현재 스트레스 사건 ( )</td><td colspan="2">취약한 지지체계 ( )</td></tr>
<tr><td colspan="6">한의사 성명: (서명/인)
작성일: 년 월 일</td></tr>
</table>

부록3. 개인정보 수집 및 이용 동의서(예시)

한의 진료를 위해 아래와 같이 개인정보를 수집하여 이용하고자 합니다. 내용을 자세히 읽으신 후 동의 여부를 결정하여 주십시오.

『개인정보』·『민감 정보 및 고유 식별정보』는 '정보의 수집 및 이용목적'을 위한 한의 진료 서비스 제공기간에 한하여 보유 및 이용되며, 서비스 제공을 위하여 수집된 정보는 개인정보 보호법, 의료법, 정신건강증진 및 정신질환자 복지서비스 지원에 관한 법률의 규정에 따라 보유하고 있습니다. 다만, 수집 목적 및 제공받은 목적이 달성된 경우에도 다른 법령 등에 의하여 보유의 필요성이 있는 경우에는 개인정보를 보유할 수 있습니다.

□ 개인정보 수집 · 이용 내역

수집·이용항목	수집·이용 목적	보유기간	보유근거
· 성명<br>· 성별<br>· 생년월일<br>· 연락처<br>· 거주지	· 재난 경험자의 회복을 위한 한의사 진료에 필요한 자료<br>· 고지사항 전달 등을 위한 원활한 의사소통 경로로 이용(전화, SMS)<br>· 교육, 연구에 필요한 분석자료	5년	『표준 개인정보 보호지침』 제60조, [별표 1호] 개인정보파일 보유기간 책정기준표

☞ 위와 같이 개인정보를 수집하는데 동의하십니까? 동의 □ 미동의 □
☞ 위와 같이 개인정보를 이용하는데 동의하십니까? 동의 □ 미동의 □

□ 민감 정보 수집 · 이용 내역

수집·이용항목	수집·이용 목적	보유기간	보유근거
재난 경험으로 인한 심리적·신체적 증상, 병력 및 서비스 제공자가 필요하다고 판단되는 개인 건강정보	· 재난 경험자에 대한 한의 진료 제공 및 조사, 연구 등에 필요한 자료	5년	『표준 개인정보 보호지침』 제60조, [별표 1호] 개인정보파일 보유기간 책정기준표

☞ 위와 같이 민감정보를 수집하는데 동의하십니까? 동의 □ 미동의 □
☞ 위와 같이 민감정보를 이용하는데 동의하십니까? 동의 □ 미동의 □

□ 개인정보 및 민감정보 제3자 제공 내역

제공받는 기관	제공목적	제공하는 항목	보유기간
재난 심리지원 관련 기관 및 의료기관	재난 경험자의 회복에 필요한 기관 또는 치료기관 연계	성명, 성별, 생년월일, 연락처, 거주지, 재난 경험관련 건강정보	5년

☞ 위와 같이 개인정보 및 민감정보를 제3자에 제공하는데 동의하십니까? 동의 □ 미동의 □

☞ 동의 거부에 따른 안내

※ 수집·이용하는 개인정보 및 민감정보는 목적 이외의 용도로 이용되지 않습니다. 귀하께서는 개인정보 제공 및 이용에 거부할 권리가 있으며, 이 경우 한의 진료 서비스 이용에 제한을 받으실 수 있습니다.

20 년 월 일

본 인 : ______________________(서명)

법정대리인 :____________________(서명)

부록4. 한국어판 외상후스트레스장애 선별검사

(The Korean Version of the Primary Care PTSD Screen for DSM-5, K-PC-PTSD-5)[200)201)]

때로는 평소와 달리 매우 두렵거나 끔찍하거나 충격적인 일들이 일어날 수도 있습니다.

예를 들면:

- 심각한 사고 또는 화재
- 신체폭력 또는 성폭력, 신체학대 또는 성학대
- 지진 또는 홍수
- 전쟁
- 다른 누군가가 죽거나 심각하게 다치는 장면을 목격함
- 사랑했던 사람이 살인이나 자살에 의해 죽게 됨

**지금까지 한번이라도 이런 종류의 사건을 경험한 적이 있습니까?**	☐ 예 ☐ 아니요

살면서 두려웠던 경험, 끔찍했던 경험, 힘들었던 경험, 그 어떤 것이라도 있다면, 그것 때문에 지난 1달 동안 다음을 경험하신 적이 있습니까?

**1. 그 경험에 관한 악몽을 꾸거나, 생각조차 하기 싫은데도 그 사건이 떠오른 적이 있었다.**	0) 아니오 1) 예
**2. 그 경험을 생각하지 않으려고 애쓰거나, 그 경험이 생각날만한 상황을 피하기 위해 특별히 노력하였다.**	0) 아니오 1) 예
**3. 계속 경계하거나, 긴장을 풀지 못하거나, 쉽게 놀라는 경우가 있었다.**	0) 아니오 1) 예
**4. 다른 사람, 일상 활동, 또는 주변에 대해 별다른 느낌이 없거나 혼자 동떨어진 느낌을 가진 적이 있었다.**	0) 아니오 1) 예
**5. 그 사건이나 그 사건으로 인해 생긴 문제에 대해 죄책감을 느끼거나, 자기 자신이나 다른 사람에 대한 원망을 멈출 수가 없었다.**	0) 아니오 1) 예

200) Prins A., Bovin MJ., Kimerling R., Kaloupek DG., Marx BP., Pless Kaiser A., Schnurr PP. The Primary Care PTSD Screen for DSM-5 (PC-PTSD-5). National Center for PTSD. 2015.(Available from https://www.ptsd.va.gov)

201) Jung YE, Kim D, Kim WH, Roh D, Chae JH, Park JE. A Brief Screening Tool for PTSD: Validation of the Korean Version of the Primary Care PTSD Screen for DSM-5(K-PC-PTSD-5). Journal of Korean Medical Science 2018;33(52):e338.

부록5. 우울증 선별검사

(Patient Health Questionnaire, PHQ-9)[202)203)]

지난 2주일 동안, 당신은 다음의 문제들로 인해서 얼마나 자주 방해를 받았는지 해당 번호에 표시(V)해 주세요.

문제	전혀 아니다	여러 날 동안	일주일 이상	거의 매일
1) 일 또는 여가 활동을 하는 데 흥미나 즐거움을 느끼지못함	0	1	2	3
2) 기분이 가라앉거나, 우울하거나, 희망이 없음	0	1	2	3
3) 잠이 들거나 계속 잠을 자는 것이 어려움, 또는 잠을 너무 많이 잠	0	1	2	3
4) 피곤하다고 느끼거나 기운이 거의 없음	0	1	2	3
5) 입맛이 없거나 과식을 함	0	1	2	3
6) 자신을 부정적으로 봄. 혹은 자신이 실패자라고 느끼거나 자신 또는 가족을 실망시킴	0	1	2	3
7) 신문을 읽거나 텔레비전 보는 것과 같은 일에 집중하는 것이 어려움	0	1	2	3
8) 다른 사람들이 주목할 정도로 너무 느리게 움직이거나말을 함. 또는 반대로 평상시보다 많이 움직여서, 너무 안절부절못하거나 들떠 있음	0	1	2	3
9) 자신이 죽는 것이 더 낫다고 생각하거나 어떤 식으로든 자신을 해칠 것이라고 생각함	0	1	2	3

한 개 증상이라도 1점 이상으로 체크를 하셨다면 해당 증상 때문에 직장이나 집안일을 하거나 주변 사람들과의 관계에서 얼마나 어려움을 겪으셨습니까?
① 전혀 없었다 ② 조금 어려웠다 ③ 많이 어려웠다 ④ 아주 어려웠다

202) Spitzer RL., Kroenke K., Williams JBW. Validation and utility of a self-report version of PRIME-MD: the PHQ primary care study. The Journal of the American Medical Association. 1999:282(18);1737-1744.

203) 박주언, 김원형, 노대영, 원성두, 김하경, 강석훈, 홍나래, 박성용, 김대호, 채정호. 재난정신건강평가 워크북. 2016.

부록6. 불안 설문지

Generalized Anxiety Disorder 7-item scale, GAD-7[204)]

지난 2주일 동안, 당신은 다음의 문제들로 인해서 얼마나 자주 방해를 받았습니까?

문제	전혀 아니다	여러 날 동안	일주일 이상	거의 매일
1) 초조하거나 불안하거나 조마조마하게 느낀다.	0	1	2	3
2) 걱정하는 것을 멈추거나 조절할 수가 없다.	0	1	2	3
3) 여러 가지 것들에 대해 걱정을 너무 많이 한다.	0	1	2	3
4) 편하게 있기가 어렵다.	0	1	2	3
5) 쉽게 짜증이 나거나 쉽게 성을 내게 된다.	0	1	2	3
6) 너무 안절부절못해서 가만히 있기가 힘들다.	0	1	2	3
7) 마치 끔찍한 일이 생길 것처럼 두렵게 느껴진다.	0	1	2	3

204)Spitzer RL., Kroenke K., Williams JB., Lowe B. A brief measure for assessing generalized anxiety disorder: the GAD-7. Archives of internal medicine. 2006;166(10):1092-1097.

부록7. 건강질문지-15

Patient Health Questionnaire, PHQ-15[205)206)]

지난 4주일 동안, 다음 나열되는 증상들에 얼마나 자주 시달렸습니까?

문제	전혀 시달리지 않음	약간 시달림	대단히 시달림
위통	0	1	2
허리 통증	0	1	2
팔, 다리, 관절(무릎, 고관절 등)의 통증	0	1	2
생리 기간 동안 생리통 등의 문제 [여성만 해당]	0	1	2
두통	0	1	2
가슴 통증, 흉통	0	1	2
어지러움	0	1	2
기절할 것 같음	0	1	2
심장이 빨리 뜀	0	1	2
숨이 참	0	1	2
성교 중 통증 등의 문제	0	1	2
변비, 묽은 변이나 설사	0	1	2
메스꺼움, 방귀, 소화불량	0	1	2
피로감, 기운 없음	0	1	2
수면의 어려움	0	1	2

205)Kroenke K., Spitzer RL., Williams JB. The PHQ–15: validity of a new measure for evaluating the severity of somatic symptoms. Psychosomatic Medicine. 2002;64:258–266.

206)Han C, Pae CU, Patkar AA, Masand PS, Kim KW, Joe SH, Jung IK. Psychometric properties of the Patient Health Questionnaire-15(PHQ-15) for measuring the somatic symptoms of psychiatric outpatients. Psychosomatics. 2009;50(6):580-585.

부록8. 자살위험성 질문지[207)208)]

당신 자신을 정말 해치겠다는 생각을 했던 적이 있습니까?			있다	없다
'있다'에 체크한 경우에만 아래 문항 1번 ~ 4-1번 문항까지에 답변하세요.<br>'없다'에 체크한 분은 답변하지 않으셔도 됩니다.			있다	없다
1	이전에 당신을 위험에 빠뜨리는 행동을 한 적이 있습니까?		있다	없다
2	당신 자신을 정말 해칠 방법에 대해 지금도 생각을 하고 있습니까?		있다	없다
2-1	있다면, 어떤 방법을 생각했습니까? (			)
3	생각하는 것과 생각을 행동에 옮기는 것은 큰 차이가 있습니다. 앞으로 한 달 내에는 어느 때라도 당신 자신을 해치거나 당신의 삶을 끝내겠다는 그 생각을 행동으로 옮길 것 같습니까?	전혀 아니다	약간 그렇다	매우 그렇다
4	당신 자신을 해치려는 당신의 행동을 멈추게 하거나 하지 못하게 막는 것이 있습니까?		있다	없다
4-1	있다면, 무엇입니까? (			)

207)Dube P, Kurt K, Bair MJ, Theobald D, Williams LS. The p4 screener: evaluation of a brief measure for assessing potential suicide risk in 2 randomized effectiveness trials of primary care and oncology patients. Primary care companion to the Journal of clinical psychiatry. 2010;12(6).

208)박주언, 김원형, 노대영, 원성두, 김하경, 강석훈, 홍나래, 박성용, 김대호, 채정호. 재난정신건강평가 워크북. 2016.

부록9. 그룹 치료 예시

회기	내용	비고
1	불안에 대한 개요<br>EFT 소개<br>임상 EFT 중 준비작업과 연속 두드리기 학습 및 실습	√ 불안 증상에 대한 개요<br>√ 스트레스가 건강과 생활에 미치는 영향<br>√ 임상 EFT 중에서 준비작업 학습 및 실습<br>√ 실습 주제는 일반적 불안 증상과 불안을 일으키는 일반적 부정적 신념에 초점을 맞추어 실습한다.<br>√ EFT 음성녹음파일 및 실습확인표를 배포 (매일 1회 이상 실습)<br>√ 일상생활에서 불안해지는 상황에 대한 자가 실습지를 과제로 제공 및 SUD(주관적 고통지수) 변화 체크
2	1회차 복습<br>임상 EFT 중 확언기법 학습 및 실습	√ 임상 EFT 중에서 준비작업과 연결하여 상황 기억 신념에 대해서 생각 바꾸기로 사용<br>√ 실습 주제는 일반적 불안 증상과 불안을 일으키는 일반적 부정적 신념에 초점을 맞추어 실습한다.<br>√ EFT 음성녹음파일 및 실습확인표를 배포 (매일 1회 이상 실습)<br>√ 일상생활에서 불안해지는 상황에 대한 자가 실습지를 과제로 제공 및 SUD 변화 체크
3	1~2회차 복습<br>EFT Basic recipe 중에서 뇌조율과정 학습 및 실습<br>피험자 개인의 상황에 대한 단체 실습	√ EFT 중에서 뇌조율과정 학습 및 실습<br>√ 각자의 상황에 맞추어 자가치료를 할 수 있는 능력을 배양하기 위한 실습<br>√ EFT 음성녹음파일 및 실습확인표를 배포 (매일 1회 이상 실습)<br>√ 일상생활에서 불안해지는 상황에 대한 자가 실습지를 과제로 제공 및 SUDs 변화 체크
4	1~3회차 복습<br>피험자 개인의 상황에 대한 단체 실습	√ 임상 EFT의 준비작업, 연속두드리기, 선택확언까지 완료<br>√ 각자의 상황에 맞추어 자가 치료를 할 수 있는 능력을 배양하기 위한 실습<br>√ EFT 음성녹음파일 및 실습확인표를 배포 (매일 1회 이상 실습)<br>√ 일상생활에서 불안해지는 상황에 대한 자가 실습지를 과제로 제공 및 SUD 변화 체크

부록10. 감정자유기법(EFT) 자가 치료 학습지

1. 심호흡을 크게 한번 합니다.

2. 지금 이 순간, 자신에게 불편한 감정을 느끼게 하는 과거/현재/미래의 상황이나 사람을 선택합니다. 어떤 일이 있었나요?

3. 그런 생각을 하면 어떤 감정이나 증상 생기나요?

(예시) 화난다, 짜증 난다, 의욕 없다, 귀찮다, 불안하다, 긴장된다, 서럽다, 억울하다, 서운하다, 서글프다, 한스럽다, 한심하다, 머리가 아프다, 어지럽다, 소화가 안된다, 답답하다, 심장이 쿵쾅거린다, 가슴에 열불이 난다.

4. 그 감정은 0에서 10 중에서 어느 정도라고 느껴집니까?
(*내가 적은 감정들 옆 칸에 적어보세요.)

편안함 **조금 힘듦** **힘들지만 견딜 수는 있음** **매우 힘듦** **견딜 수 없음**

0 1 2 3 4 5 6 7 8 9 10

❶ 준비작업(Set-up)

시기별로 자신에 맞는 확언을 적용합니다.
____ 에는 해결하려는 상황·감정을 그대로 넣고, 해당 문장을 3회 반복해서 말합니다.

시기별	수용확언과 선택확언
**급성기 (~1개월)**	나는 비록 _____하지만, 나는 지금 내가 안전하다고 깨닫는 것을 선택합니다.
**아급성기 (1~3개월)**	나는 비록 _____하지만, 지금의 나는 안전합니다. 나는 이런 나 자신을 진심으로 완전히 받아들입니다.
**만성기 (3개월~)**	비록 나는 ______ 이 있을지라도, 나는 이런 나 자신을 마음속 깊이 진심으로 받아들입니다.
**급성기 예시**	나는 비록 불안하지만, 나는 지금 내가 안전하다고 깨닫는 것을 선택합니다.

❷ 연속 두드리기(Sequence)

*불편한 감정 증상을 반복하면서 두드림 : 예시) 불안하다, 심장이 쿵쾅거린다.

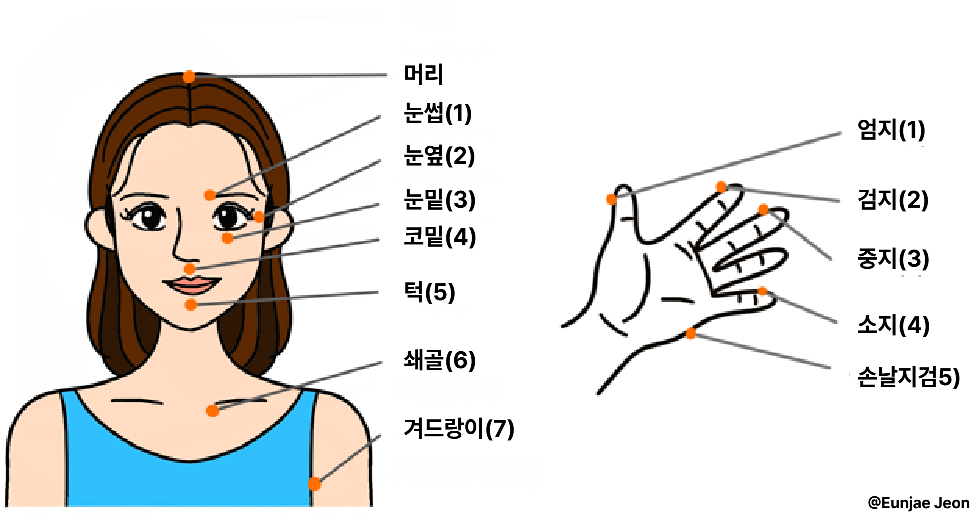

❸ 선택확언(Choice method)

나쁜 기억과 불안한 미래와 나를 힘들게 하는 현재 상황에도 불구하고,
나는 나를 상처 주지 않겠습니다.
나는 나를 비난하지 않겠습니다.
나는 나를 얕잡아보지 않겠습니다.
나는 내 삶의 주인공으로 살아갑니다.
나는 나 자신을 진심으로 이해하고 받아들이고 사랑합니다.
나는 나 자신을 점점 더 이해하고 사랑하는 삶을 살아갑니다.
나는 점점 더 편안해집니다.
나는 점점 더 건강해집니다.
나는 점점 더 유쾌해집니다.
나는 점점 더 행복해집니다.

재난 트라우마의 한의사 진료 매뉴얼 학회인증서

대한한방신경정신과학회 인증평가위원회에서는 대구한의대학교 한의과대학 김상호 교수 연구팀에서 개발한 '재난 트라우마의 한의사 진료 매뉴얼'을 검토하였으며, 그 내용에 대해 학회 인증을 합니다.

2021년 09월 03일

대한한방신경정신과학회장

본 매뉴얼은 정부(과학기술정보통신부)의 재원으로 한국연구재단의 지원을 받아 수행된 연구 성과임
(No.2019R1G1A100591513).

재난트라우마의 한의사 진료 매뉴얼(안)

2022년 4월 20일 1판 1쇄

저자 | 김상호, 권찬영, 서주희
발행인 | 임동규
발행처 | **(주)집문당**
등록 | 1971. 3. 23. 제2012-000069호
주소 | 03134 서울시 종로구 돈화문로 82, 5층
전화 | +82-1811-7567
이메일 | sale@jipmoon.com
홈페이지 | www.jipmoon.com

ISBN 978-89-303-1928-7 95510
비매품

편집 스튜디오 디자인밤(pbr1202@hanmail.net)

(주)집문당 이순신돋움체B (저작권자 아산시, 무료글꼴)
